# Effect of remote ischaemic conditioning on clinical outcomes in patients with acute myocardial infarction (CONDI-2/ERIC-PPCI): a single-blind randomised controlled trial

**DOI:** 10.1016/S0140-6736(19)32039-2

**Published:** 2019-10-19

**Authors:** Derek J Hausenloy, Rajesh K Kharbanda, Ulla Kristine Møller, Manish Ramlall, Jens Aarøe, Robert Butler, Heerajnarain Bulluck, Tim Clayton, Ali Dana, Matthew Dodd, Thomas Engstrom, Richard Evans, Jens Flensted Lassen, Erika Frischknecht Christensen, José Manuel Garcia-Ruiz, Diana A Gorog, Jakob Hjort, Richard F Houghton, Borja Ibanez, Rosemary Knight, Freddy K Lippert, Jacob T Lønborg, Michael Maeng, Dejan Milasinovic, Ranjit More, Jennifer M Nicholas, Lisette Okkels Jensen, Alexander Perkins, Nebojsa Radovanovic, Roby D Rakhit, Jan Ravkilde, Alisdair D Ryding, Michael R Schmidt, Ingunn Skogstad Riddervold, Henrik Toft Sørensen, Goran Stankovic, Madhusudhan Varma, Ian Webb, Christian Juhl Terkelsen, John P Greenwood, Derek M Yellon, Hans Erik Bøtker, CONDI-2/ERIC-PPCI Investigators, Anders Junker, Anne Kaltoft, Morten Madsen, Evald Høj Christiansen, Lars Jakobsen, Steen Carstensen, Steen Dalby Kristensen, Troels Thim, Karin Møller Pedersen, Mette Tidemand Korsgaard, Allan Iversen, Erik Jørgensen, Francis Joshi, Frants Pedersen, Hans Henrik Tilsted, Karam Alzuhairi, Kari Saunamäki, Lene Holmvang, Ole Ahlehof, Rikke Sørensen, Steffen Helqvist, Bettina Løjmand Mark, Anton Boel Villadsen, Bent Raungaard, Leif Thuesen, Martin Kirk Christiansen, Philip Freeman, Svend Eggert Jensen, Charlotte Schmidt Skov, Ahmed Aziz, Henrik Steen Hansen, Julia Ellert, Karsten Veien, Knud Erik Pedersen, Knud Nørregård Hansen, Ole Ahlehoff, Helle Cappelen, Daniel Wittrock, Poul Anders Hansen, Jens Peter Ankersen, Kim Witting Hedegaard, John Kempel, Henning Kaus, Dennis Erntgaard, Danny Mejsner Pedersen, Matthias Giebner, Troels Martin Hansen Hansen, Mina Radosavljevic-Radovanovic, Maja Prodanovic, Lidija Savic, Marijana Pejic, Dragan Matic, Ana Uscumlic, Ida Subotic, Ratko Lasica, Vladan Vukcevic, Alfonso Suárez, Beatriz Samaniego, César Morís, Eduardo Segovia, Ernesto Hernández, Iñigo Lozano, Isaac Pascual, Jose M. Vegas-Valle, José Rozado, Juan Rondán, Pablo Avanzas, Raquel del Valle, Remigio Padrón, Alfonso García-Castro, Amalia Arango, Ana B. Medina-Cameán, Ana I. Fente, Ana Muriel-Velasco, Ángeles Pomar-Amillo, César L. Roza, César M. Martínez-Fernández, Covadonga Buelga-Díaz, David Fernández-Gonzalo, Elena Fernández, Eloy Díaz-González, Eugenio Martinez-González, Fernando Iglesias-Llaca, Fernando M. Viribay, Francisco J. Fernández-Mallo, Francisco J. Hermosa, Ginés Martínez-Bastida, Javier Goitia-Martín, José L. Vega-Fernández, Jose M. Tresguerres, Juan A. Rodil-Díaz, Lara Villar-Fernández, Lucía Alberdi, Luis Abella-Ovalle, Manuel de la Roz, Marcos Fernández-Carral Fernández-Carral, María C. Naves, María C. Peláez, María D. Fuentes, María García-Alonso, María J. Villanueva, María S. Vinagrero, María Vázquez-Suárez, Marta Martínez-Valle, Marta Nonide, Mónica Pozo-López, Pablo Bernardo-Alba, Pablo Galván-Núñez, Polácido J. Martínez-Pérez, Rafael Castro, Raquel Suárez-Coto, Raquel Suárez-Noriega, Rocío Guinea, Rosa B. Quintana, Sara de Cima, Segundo A. Hedrera, Sonia I. Laca, Susana Llorente-Álvarez, Susana Pascual, Teodorna Cimas, Anthony Mathur, Eleanor McFarlane-Henry, Gerry Leonard, Jessry Veerapen, Mark Westwood, Martina Colicchia, Mary Prossora, Mervyn Andiapen, Saidi Mohiddin, Valentina Lenzi, Jun Chong, Rohin Francis, Amy Pine, Caroline Jamieson-Leadbitter, Debbie Neal, J. Din, Jane McLeod, Josh Roberts, Karin Polokova, Kristel Longman, Lucy Penney, Nicki Lakeman, Nicki Wells, Oliver Hopper, Paul Coward, Peter O'Kane, Ruth Harkins, Samantha Guyatt, Sarah Kennard, Sarah Orr, Stephanie Horler, Steve Morris, Tom Walvin, Tom Snow, Michael Cunnington, Amanda Burd, Anne Gowing, Arvindra Krishnamurthy, Charlotte Harland, Derek Norfolk, Donna Johnstone, Hannah Newman, Helen Reed, James O'Neill, John Greenwood, Josephine Cuxton, Julie Corrigan, Kathryn Somers, Michelle Anderson, Natalie Burtonwood, Petra Bijsterveld, Richard Brogan, Tony Ryan, Vivek Kodoth, Arif Khan, Deepti Sebastian, Diana Gorog, Georgina Boyle, Lucy Shepherd, Mahmood Hamid, Mohamed Farag, Nicholas Spinthakis, Paulina Waitrak, Phillipa De Sousa, Rishma Bhatti, Victoria Oliver, Siobhan Walshe, Toral Odedra, Ying Gue, Rahim Kanji, Alisdair Ryding, Amanda Ratcliffe, Angela Merrick, Carol Horwood, Charlotte Sarti, Clint Maart, Donna Moore, Francesca Dockerty, Karen Baucutt, Louise Pitcher, Mary Ilsley, Millie Clarke, Rachel Germon, Sara Gomes, Thomas Clare, Sunil Nair, Jocasta Staines, Susan Nicholson, Oliver Watkinson, Ian Gallagher, Faye Nelthorpe, Janine Musselwhite, Konrad Grosser, Leah Stimson, Michelle Eaton, Richard Heppell, Sharon Turney, Victoria Horner, Natasha Schumacher, Angela Moon, Paula Mota, Joshua O'Donnell, Abeesh Sadasiva Panicker, Anntoniette Musa, Luke Tapp, Suresh Krishnamoorthy, Valerie Ansell, Danish Ali, Samantha Hyndman, Prithwish Banerjee, Martin Been, Ailie Mackenzie, Andrew McGregor, David Hildick-Smith, Felicity Champney, Fiona Ingoldby, Kirstie Keate, Lorraine Bennett, Nicola Skipper, Sally Gregory, Scott Harfield, Alexandra Mudd, Christopher Wragg, David Barmby, Ever Grech, Ian Hall, Janet Middle, Joann Barker, Joyce Fofie, Julian Gunn, Kay Housley, Laura Cockayne, Louise Weatherlley, Nana Theodorou, Nigel Wheeldon, Pene Fati, Robert F. Storey, James Richardson, Javid Iqbal,, Zul Adam, Sarah Brett, Michael Agyemang, Cecilia Tawiah, Kai Hogrefe, Prashanth Raju, Christine Braybrook, Jay Gracey, Molly Waldron, Rachael Holloway, Senem Burunsuzoglu, Sian Sidgwick, Simon Hetherington, Charmaine Beirnes, Olga Fernandez, Nicoleta Lazar, Abigail Knighton, Amrit Rai, Amy Hoare, Ian Webb, Jonathan Breeze, Katherine Martin, Michelle Andrews, Sheetal Patale, Amy Bennett, Andrew Smallwood, Elizabeth Radford, James Cotton, Joe Martins, Lauren Wallace, Sarah Milgate, Shahzad Munir, Stella Metherell, Victoria Cottam, Ian Massey, Jane Copestick, Jane Delaney, Jill Wain, Kully Sandhu, Lisa Emery, Robert Butler, Charlotte Hall, Chiara Bucciarelli-Ducci, Rissa Besana, Jodie Hussein, Sheila Bell, Abby Gill, Emily Bales, Gary Polwarth, Clare East, Ian Smith, Joana Oliveira, Saji Victor, Sarah Woods, Stephen Hoole, Angelo Ramos, Annaliza Sevillano, Anne Nicholson, Ashley Solieri, Emily Redman, Jean Byrne, Joan Joyce, Joanne Riches, John Davies, Kezia Allen, Louie Saclot, Madelaine Ocampo, Mark Vertue, Natasha Christmas, Raiji Koothoor, Reto Gamma, Wilson Alvares, Stacey Pepper, Barbara Kobson, Christy Reeve, Iqbal Malik, Emma Chester, Heidi Saunders, Idah Mojela, Joanna Smee, Justin Davies, Nina Davies, Piers Clifford, Priyanthi Dias, Ramandeep Kaur, Silvia Moreira, Yousif Ahmad, Lucy Tomlinson, Clare Pengelley, Amanda Bidle, Sharon Spence, Rasha Al-Lamee, Urmila Phuyal, Hakam Abbass, Tuhina Bose, Rebecca Elliott, Aboo Foundun, Alan Chung, Beth Freestone, Dr Kaeng Lee, Dr Mohamed Elshiekh, George Pulikal, Gurbir Bhatre, James Douglas, Lee Kaeng, Mike Pitt, Richard Watkins, Simrat Gill, Amy Hartley, Andrew Lucking, Berni Moreby, Damaris Darby, Ellie Corps, Georgina Parsons, Gianluigi De Mance, Gregor Fahrai, Jenny Turner, Jeremy Langrish, Lisa Gaughran, Mathias Wolyrum, Mohammed Azkhalil, Rachel Bates, Rachel Given, Rajesh Kharbanda, Rebecca Douthwaite, Steph Lloyd, Stephen Neubauer, Deborah Barker, Ali Dana, Anne Suttling, Charlotte Turner, Clare Smith, Colin Longbottom, David Ross, Denise Cunliffe, Emily Cox, Helena Whitehead, Karen Hudson, Leslie Jones, Martin Drew, Nicholas Chant, Peter Haworth, Robert Capel, Rosalynn Austin, Serena Howe, Trevor Smith, Alex Hobson, Philip Strike, Huw Griffiths, Brijesh Anantharam, Pearse Jack, Emma Thornton, Adrian Hodgson, Alan Jennison, Anna McSkeane, Bethany Smith, Caroline Shaw, Chris Leathers, Elissa Armstrong, Gayle Carruthers, Holly Simpson, Jan Smith, Jeremy Hodierne, Julie Kelly, Justin Barclay, Kerry Scott, Lisa Gregson, Louise Buchanan, Louise McCormick, Madhusudhan Varma, Nicci Kelsall, Rachel Mcarthy, Rebecca Taylor, Rebecca Thompson, Rhidian Shelton, Roger Moore, Sharon Tomlinson, Sunil Thambi, Theresa Cooper, Trevor Oakes, Zakhira Deen, Chris Relph, Scott prentice, Lorna Hall, Angela Dillon, Deborah Meadows, Emma Frank, Helene Markham-Jones, Isobel Thomas, Joanne Gale, Joanne Denman, Joanne Gale, John O'Connor, Julia Hindle, Karen Jackson-Lawrence, Karen Warner, Kelvin Lee, Robert Upton, Ruth Elston, Sandra Lee, Vinod Venugopal, Amanda Finch, Catherine Fleming, Charlene Whiteside, Chris Pemberton, Conor Wilkinson, Deepa Sebastian, Ella Riedel, Gaia Giuffrida, Gillian Burnett, Helen Spickett, James Glen, Janette Brown, Lauren Thornborough, Lauren Pedley, Maureen Morgan, Natalia Waddington, Oliver Brennan, Ranjit More, Rebecca Brady, Stephen Preston, Chris Loder, Ionela Vlad, Julia Laurence, Angelique Smit, Kirsty Dimond, Michelle Hayes, Loveth Paddy, Jacolene Crause, Nadifa Amed, Priya Kaur-Babooa, Roby Rakhit, Tushar Kotecha, Hossam Fayed, Rohin Francis, Antonis Pavlidis, Bernard Prendergast, Brian Clapp, Divaka Perara, Emma Atkinson, Howard Ellis, Karen Wilson, Kirsty Gibson, Megan Smith, Muhammed Zeeshan Khawaja, Ruth Sanchez-Vidal, Simon Redwood, Sophie Jones, Aoife Tipping, Anu Oommen, Cara Hendry, DR Fazin Fath-Orboubadi, Hannah Phillips, Laurel Kolakaluri, Martin Sherwood, Sarah Mackie, Shilpa Aleti, Thabitha Charles, Liby Roy, Rob Henderson, Rod Stables, Simon Redwood, Michael Marber, Alan Berry, Andrew Redington, Kristian Thygesen, Henning Rud Andersen, Colin Berry, Andrew Copas, Tom Meade, Henning Kelbæk, Hector Bueno, Paul von Weitzel-Mudersbach, Grethe Andersen, Andrew Ludman, Nick Cruden, Dragan Topic, Zlatko Mehmedbegovic, Jesus Maria de la Hera Galarza, Steven Robertson, Laura Van Dyck, Rebecca Chu, Josenir Astarci, Zahra Jamal, Daniel Hetherington, Lucy Collier

**Affiliations:** a The Hatter Cardiovascular Institute, University College London, London, UK; b Royal Free Hospital London and Institute of Cardiovascular Science, University College London, London, UK; c National Institute of Health Research Biomedical Research Centre at University College London Hospitals, Research & Development, London, UK; d Cardiovascular & Metabolic Disorders Program, Duke-National University of Singapore Medical School, Singapore; e National Heart Research Institute Singapore, National Heart Centre, Singapore; f Yong Loo Lin School of Medicine, National University Singapore, Singapore; g Centro de Biotecnologia-FEMSA, Tecnologico de Monterrey, Monterrey, Mexico; h Oxford Heart Centre, Oxford University Hospitals National Health Service Trust, Oxford, UK; i Department of Cardiovascular Medicine, University of Oxford, Oxford, UK; j Department of Cardiology, Aarhus University Hospital, Aarhus, Denmark; k Department of Clinical Epidemiology, Aarhus University Hospital, Aarhus, Denmark; l University Hospital Southampton National Health Service Foundation Trust, Southampton, UK; m Department of Cardiology, Aalborg University Hospital, Aalborg, Denmark; n Department of Cardiology, University Hospitals of North Midlands, Royal Stoke University Hospital, Stoke-on-Trent, UK; o Department of Cardiology, Norfolk and Norwich University Hospital, Norwich, UK; p Clinical Trials Unit and Department of Medical Statistics, London School of Hygiene & Tropical Medicine, London, UK; q Portsmouth Hospitals National Health Service Trust, Portsmouth, UK; r Department of Cardiology, Rigshospitalet, University of Copenhagen, Copenhagen, Denmark; s Prehospital Emergency Medical Services, Central Denmark Region, Aarhus, Denmark; t Instituto de Investigación Sanitaria del Principado de Asturias, Hospital Universitario de Cabueñes, Oviedo, Spain; u Centro Nacional de Investigaciones Cardiovasculares, Madrid, Spain; v Centro de Investigación Biomédica en Red de Enfermedades Cardiovasculares, Madrid, Spain; w Department of Cardiology, Lister Hospital, East and North Hertfordshire National Health Service Trust, Stevenage, UK; x National Heart and Lung Institute, Imperial College London, London, UK; y Department of Clinical Medicine, Aarhus University, Aarhus, Denmark; z Servicio de Atención Médica de Urgencia—Asturias, Oviedo, Spain; aa Centro de Investigacion Biomedica En Red Cardiovascular, Madrid, Spain; ab IIS-Fundación Jiménez Díaz University Hospital, Madrid, Spain; ac Prehospital Emergency Medical Services, Capital Region of Denmark, Denmark; ad Department of Cardiology, Clinical Centre of Serbia, Faculty of Medicine, University of Belgrade, Belgrade, Serbia; ae Cardiology Clinic, Clinical Centre of Serbia, Faculty of Medicine, University of Belgrade, Belgrade, Serbia; af Emergency Centre, Clinical Centre of Serbia, Faculty of Medicine, University of Belgrade, Belgrade, Serbia; ag Department for Diagnostic and Catheterization Laboratories, Clinical Centre of Serbia, Faculty of Medicine, University of Belgrade, Belgrade, Serbia; ah Lancashire Cardiac Centre, Blackpool Teaching Hospitals National Health Service Foundation Trust, Blackpool, UK; ai Department of Cardiology, Odense University Hospital, Odense, Denmark; aj The Heart Centre, North Cumbria University Hospitals National Health Service Trust, Carlisle, UK; ak King's College Hospital, King's Health Partnership, London, UK; al Leeds Institute of Cardiovascular and Metabolic Medicine, University of Leeds, Leeds, UK; am Leeds Teaching Hospitals National Health Service Trust, Leeds, UK

## Abstract

**Background:**

Remote ischaemic conditioning with transient ischaemia and reperfusion applied to the arm has been shown to reduce myocardial infarct size in patients with ST-elevation myocardial infarction (STEMI) undergoing primary percutaneous coronary intervention (PPCI). We investigated whether remote ischaemic conditioning could reduce the incidence of cardiac death and hospitalisation for heart failure at 12 months.

**Methods:**

We did an international investigator-initiated, prospective, single-blind, randomised controlled trial (CONDI-2/ERIC-PPCI) at 33 centres across the UK, Denmark, Spain, and Serbia. Patients (age >18 years) with suspected STEMI and who were eligible for PPCI were randomly allocated (1:1, stratified by centre with a permuted block method) to receive standard treatment (including a sham simulated remote ischaemic conditioning intervention at UK sites only) or remote ischaemic conditioning treatment (intermittent ischaemia and reperfusion applied to the arm through four cycles of 5-min inflation and 5-min deflation of an automated cuff device) before PPCI. Investigators responsible for data collection and outcome assessment were masked to treatment allocation. The primary combined endpoint was cardiac death or hospitalisation for heart failure at 12 months in the intention-to-treat population. This trial is registered with ClinicalTrials.gov (NCT02342522) and is completed.

**Findings:**

Between Nov 6, 2013, and March 31, 2018, 5401 patients were randomly allocated to either the control group (n=2701) or the remote ischaemic conditioning group (n=2700). After exclusion of patients upon hospital arrival or loss to follow-up, 2569 patients in the control group and 2546 in the intervention group were included in the intention-to-treat analysis. At 12 months post-PPCI, the Kaplan-Meier-estimated frequencies of cardiac death or hospitalisation for heart failure (the primary endpoint) were 220 (8·6%) patients in the control group and 239 (9·4%) in the remote ischaemic conditioning group (hazard ratio 1·10 [95% CI 0·91–1·32], p=0·32 for intervention versus control). No important unexpected adverse events or side effects of remote ischaemic conditioning were observed.

**Interpretation:**

Remote ischaemic conditioning does not improve clinical outcomes (cardiac death or hospitalisation for heart failure) at 12 months in patients with STEMI undergoing PPCI.

**Funding:**

British Heart Foundation, University College London Hospitals/University College London Biomedical Research Centre, Danish Innovation Foundation, Novo Nordisk Foundation, TrygFonden.

## Introduction

Despite timely reperfusion with primary percutaneous coronary intervention (PPCI), morbidity and mortality following acute ST-elevation myocardial infarction (STEMI) remain substantial, and improvements in mortality and survival free of heart failure have plateaued.^1^, ^2^ New treatments are needed to reduce myocardial infarct size and preserve cardiac function to reduce risk of death and prevent onset of heart failure. In this regard, remote ischaemic conditioning, in which brief cycles of ischaemia and reperfusion are applied to an organ or tissue (including a limb) away from the heart, has been shown to reduce myocardial infarct size in animal models.^2^, ^3^

In the clinical setting, the cardioprotective remote ischaemic conditioning stimulus can be applied using serial inflations and deflations of a pneumatic cuff placed on the upper arm or thigh to induce brief cycles of ischaemia and reperfusion.^4^ In most clinical studies in patients with STEMI, remote ischaemic conditioning has increased myocardial salvage and reduced myocardial infarct size by 20–30% when applied before or during reperfusion.^5^, ^6^, ^7^, ^8^, ^9^, ^10^, ^11^, ^12^ In addition, two follow-up studies and a prospective single-centre study have suggested that remote ischaemic conditioning might also improve clinical outcomes in patients with STEMI undergoing PPCI.^13^, ^14^, ^15^ However, a large, sufficiently powered, prospectively designed multicentre clinical outcome study has not yet been done. Therefore, in the CONDI-2/ERIC-PPCI trial, we investigated the effect of remote ischaemic conditioning applied as an adjunct to PPCI on rates of cardiac death or hospitalisation for heart failure at 12 months in patients with STEMI. In this study, we combined the CONDI-2 and ERIC-PPCI trials and harmonised the protocols to increase sample size and ensure the study was sufficiently powered to detect an effect of remote ischaemic conditioning on clinical outcomes in patients with STEMI treated by PPCI.

**Research in context**

**Evidence before this study**

To improve clinical outcomes in patients with ST-elevation myocardial infarction (STEMI) treated by primary percutaneous coronary intervention (PPCI), new treatments are needed to protect the heart against acute ischaemia–reperfusion injury and thus reduce myocardial infarct size and prevent heart failure. Remote ischaemic conditioning with transient ischaemia and reperfusion of the arm or leg as an adjunct to PPCI has been shown in several small randomised controlled trials to reduce myocardial infarct size and increase myocardial salvage in patients with STEMI when compared with use of PPCI alone. However, whether remote ischaemic conditioning can improve clinical outcomes such as mortality and heart failure has not been confirmed. A PubMed search undertaken up to the start date of the trial (July 14, 2013) using the terms “remote ischemic conditioning; acute myocardial infarction; mortality; and heart failure” revealed no randomised controlled trials investigating the effect of remote ischaemic conditioning on clinical outcomes in patients with STEMI treated by PPCI.

**Added value of this study**

In this large, sufficiently powered, multicentre, randomised controlled trial of 5401 patients with STEMI, we investigated whether remote ischaemic conditioning applied as an adjunct to PPCI could reduce the incidence of cardiac death or hospitalisation for heart failure within 12 months post-PPCI, when compared with PPCI alone. Our findings showed that remote ischaemic conditioning did not improve clinical outcomes in terms of cardiac death or hospitalisation for heart failure at this timepoint. Furthermore, no improvements in any of the secondary endpoints were found, including major adverse cardiovascular and cerebral events and myocardial infarct size evaluated by troponin T release. Furthermore, no effects of the intervention versus the control were seen among prespecified subgroup analyses by age, presence of diabetes, pre-PPCI coronary flow, ischaemia time, or infarct location.

**Implications of all the available evidence**

The CONDI-2/ERIC-PPCI trial has provided definitive evidence that remote ischaemic conditioning offers no benefits on either myocardial infarct size or clinical outcomes at 12 months in patients with STEMI treated with PPCI. Two follow-up randomised controlled trials and a prospective single-centre randomised controlled trial, published since the commencement of our trial, had suggested that remote ischaemic conditioning might improve clinical outcomes in patients with STEMI undergoing PPCI. The two follow-up trials were not prospectively designed or powered to detect a difference in clinical outcomes with remote ischaemic conditioning. The prospective single-centre trial might not have been sufficiently powered, and it had an extended follow-up period. Until now, remote ischaemic conditioning had been the most promising potential cardioprotective strategy for improving clinical outcomes following STEMI. Therefore, identification of novel cardioprotective targets and discovery of innovative approaches to cardioprotection are needed to improve clinical outcomes in patients with STEMI treated by PPCI. Such approaches might include combination multitarget therapy. Remote ischaemic conditioning might still be beneficial in other clinical settings of acute ischaemia–reperfusion injury, including renal transplantation, acute kidney injury, and stroke.

## Methods

### Study design

We did an international, multicentre, single-blind, randomised controlled trial at 26 centres in the UK (the ERIC-PPCI component study), and at four hospitals in Denmark, two hospitals in Spain, and one hospital in Serbia (the CONDI-2 component study). The CONDI-2/ERIC-PPCI study received ethical approval from regional and national health service research ethics committees and was conducted in accordance with the principles of good clinical practice. In the CONDI-2 component study, all participants provided written informed consent before randomisation. In the ERIC-PPCI component study, all patients provided initial verbal assent before randomisation, which was followed by written informed consent. The London School of Hygiene & Tropical Medicine Clinical Trials Unit (London, UK) coordinated the trial in collaboration with the Cardiology Trial Unit and Department of Clinical Epidemiology of Aarhus University Hospital (Aarhus, Denmark). Details of the trial design have been reported previously,^16^ and a copy of the protocol is available in appendix 2.

### Participants

Patients with chest pain and suspected ST-segment elevation on electrocardiogram (ECG) were screened for possible inclusion. Patients were included if they were older than 18 years of age, had ST-segment elevation on ECG, and were eligible for PPCI. Exclusion criteria were previous coronary artery bypass graft surgery, myocardial infarct within the previous 30 days, left bundle branch block on ECG, treatment with therapeutic hypothermia, conditions precluding use of remote ischaemic conditioning (paresis of upper limb, or presence of an arteriovenous shunt), and life expectancy of less than 1 year due to a non-cardiac pathology.

### Randomisation and masking

In both trials, patients were randomly allocated (1:1) to a remote ischaemic conditioning group or a control group by a designated study team member who was unmasked to treatment allocation. For ERIC-PPCI, randomisation was done via a secure website, Sealed Envelope (London, UK), and was stratified by centre with use of random permuted blocks (block sizes of 4 or 6). For CONDI-2, randomisation was done through a web-based clinical trial support system accessible 24 h a day (TrialPartner, Aarhus, Denmark) and stratified by centre using random permuted blocks (block sizes of 4, 6, or 8). In both trials, access to the randomisation website and list were strictly controlled at each site and limited to unmasked study team members. Study team members collecting the data and assessing outcomes were masked to the treatment allocation.

### Procedures

An automated AutoRIC cuff device (CellAegis Devices, Toronto, ON, Canada) was used to deliver the remote ischaemic conditioning protocol, which comprised four alternating cycles of cuff inflation for 5 min to 200 mm Hg and deflation for 5 min. At the UK sites, the control group received a sham simulated remote ischaemic conditioning intervention. At the sites in Denmark, Spain, and Serbia, the control group received standard therapy. The remote ischaemic conditioning and control protocols were implemented before PPCI and did not delay the onset of PPCI. Where ambulance transit times allowed, remote ischaemic conditioning was administered in the ambulance (in Denmark and Spain). Where ambulance transit times were shorter, remote ischaemic conditioning was delivered immediately upon arrival at the PPCI centre (in Serbia and the UK).

Before PPCI, patients were treated with 300 mg aspirin orally or intravenously, 600 mg clopidogrel or 180 mg ticagrelor orally, and standard weight-adjusted heparin intravenously. The PPCI procedure followed guideline recommendations;^17^ ad hoc thrombectomy and administration of glycoprotein IIb/IIIa inhibitors, bivalirudin, or cangrelor were per operator discretion.

### Outcomes

The primary combined endpoint was cardiac death or hospitalisation for heart failure at 12 months post-randomisation in the intention-to-treat population. Hospitalisation for heart failure included heart failure both during the index hospitalisation and during rehospitalisation for heart failure. Hospitalisation was defined as treatment occurring during a hospital admission. Heart failure was judged to be present on the basis of at least one of the following symptoms and signs: new or worsening dyspnoea, orthopnoea, or paroxysmal nocturnal dyspnoea; increasing fatigue or worsening exercise tolerance; ejection fraction less than 45% on echocardiography; new pulmonary oedema seen on chest x-ray in the absence of a non-cardiac cause; crepitations believed to be due to pulmonary oedema; and use of loop diuretics to treat presumed pulmonary congestion.^18^ A blinded independent event validation committee reviewed all events according to standard operational procedures. Hospitalisation for heart failure was defined as an event that met the standard criteria^18^ (appendix 1 p 20).

Prespecified secondary endpoints, analysed in the intention-to-treat population, included combined cardiac death or hospitalisation for heart failure at 30 days; cardiac death and hospitalisation for heart failure (as individual endpoints) at 30 days and 12 months; major cardiovascular and cerebral adverse events (comprising all-cause death, reinfarction, repeat coronary revascularisation, and stroke; appendix 1 p 20) at 30 days and 12 months; incidence of implantable cardioverter-defibrillator insertion within 12 months; repeated episodes of hospitalisation for heart failure within 12 months; and myocardial infarct size (quantified by area under the curve [AUC] for high-sensitivity troponin T measured at 0–48 h after PPCI) in a subset of patients.

### Statistical analysis

The primary outcome event rate was originally predicted to be 11·0% at 12 months. To show a 25% relative reduction in the remote ischaemic conditioning group (from 11·0% to 8·25%) with 80% power and 5% significance would have required 4300 patients (2000 patients in ERIC-PPCI and 2300 in CONDI-2), allowing for 15% attrition. A blinded review of accruing data revealed that the estimated event rate was lower than anticipated and the sample size was, therefore, increased to 5400 patients (2800 patients in ERIC-PPCI and 2600 in CONDI-2).

The primary and secondary time-to-event outcomes were compared between the remote ischaemic conditioning and control groups with use of a Cox regression model stratified by the two component studies on an intention-to-treat basis, and presented with Kaplan-Meier curves. To account for recurrent hospitalisation for heart failure and competing mortality risk, a negative binomial model was used to compare the total number of outcomes experienced by the two groups up to 12 months after randomisation. The proportion of patients with an implantable cardioverter-defibrillator, biventricular pacemaker, or single-chamber or dual-chamber pacemaker at 12 months was compared using a generalised linear model for a binary outcome with a log link.

Prespecified analyses by subgroup (age [<55 years, 55 to <65 years, 65 to <75 years, or ≥75 years], diabetic status, infarct location [left anterior descending artery or other], pre-angioplasty TIMI flow grade [0–1 or 2–3], and time elapsed between first medical contact and PPCI [<90 min, 90 to <120 min, or 120 to 720 min]) were done on the primary outcome. The subgroups were analysed by including an interaction term between treatment group and subgroup in the Cox regression model.

Troponin T values were converted into a 48-h AUC summary measure for analysis, using multiple imputation by chained equations to account for missing data. As the distribution of the outcome variable was highly skewed, a log transformation was done. Differences were analysed by use of a linear regression model with the treatment effect presented as a ratio of the geometric means.

We also did a per-protocol analysis of all primary and secondary outcomes limited to patients who had a confirmed STEMI, received the full intervention as specified in the protocol, and had a completed PPCI.

Results were considered statistically significant if the two-sided p value was less than 0·05. Full details of the statistical methods are provided in the protocol and statistical analysis plan, which are available in appendix 2.

The CONDI-2/ERIC-PPCI trial is registered at ClinicalTrials.gov (number NCT02342522) and is a planned prespecified collaboration between the CONDI-2 and ERIC-PPCI studies (for further details see appendix 1 pp 20–21).

### Role of the funding source

The sponsor had no role in the study design, data collection, data analysis, data interpretation, or writing of the report. The co-corresponding authors had full access to all the data in the study and had final responsibility for the decision to submit for publication.

## Results

**Figure 1:**
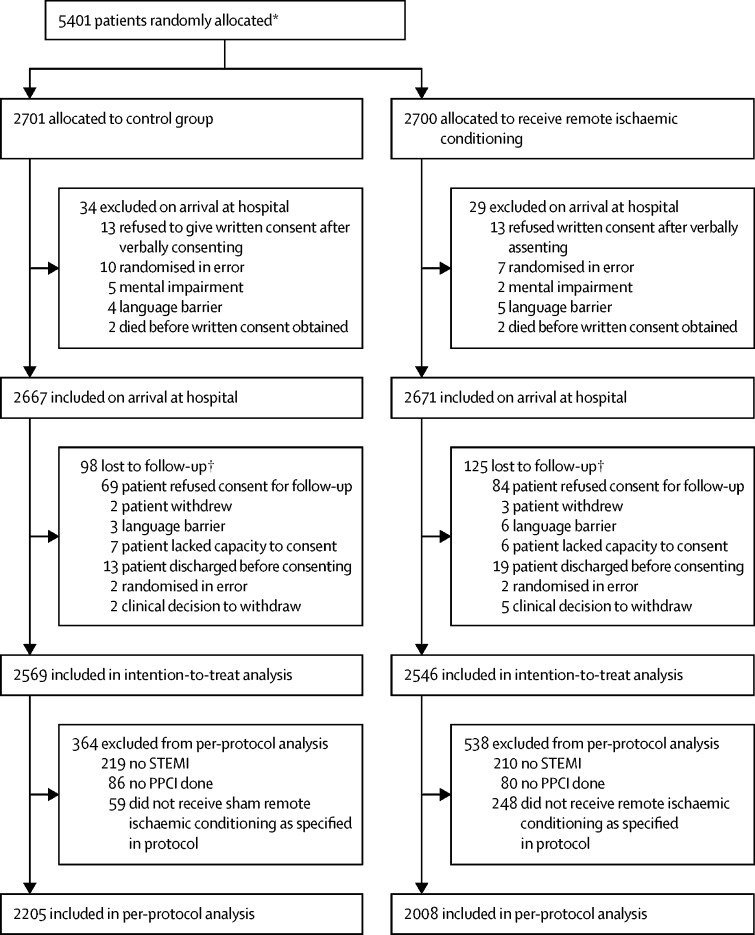
Trial profile PPCI=primary percutaneous coronary intervention. STEMI=ST-elevation myocardial infarction. *Full screening log data were not available as not all sites were able to collect screening logs given the emergency context of patient enrolment and randomisation. †ERIC-PPCI had approval from the UK Confidentiality Group to collect data on patients who died before consent could be obtained (20 patients in the control group and 22 patients in the remote ischaemic conditioning group); thus, these patients were included in subsequent analyses.

Between Nov 6, 2013, and March 31, 2018, 5401 patients were randomly allocated: 2701 to the control group and 2700 to the remote ischaemic conditioning group. 5115 patients (2569 in the control group and 2546 in the remote ischaemic conditioning group) were included in the intention-to-treat analysis (figure 1). The treatment groups were well balanced with respect to both patient baseline characteristics and PPCI details for the intention-to-treat analysis (table 1). The intervention was completed according to protocol for 2205 participants in the control group and 2008 in the remote ischaemic conditioning group, and the results were included in the per-protocol analysis. Reasons for incomplete interventions are provided in figure 1.

**Table 1:** Baseline characteristics and procedural details for the intention-to-treat population

		**Control group (n=2569)**	**Remote ischaemic conditioning group (n=2546)**
**Baseline characteristics**			
Age, years		63·1 (12·2)	63·9 (12·1)
Sex			
	Female	576/2569 (22·4%)	611/2546 (24·0%)
	Male	1993/2569 (77·6%)	1935/2546 (76·0%)
Body-mass index (kg/m^2^)		27·5 (4·7)	27·5 (4·9)
Current smoker		1015/2483 (40·9%)	993/2449 (40·5%)
Comorbidities			
	Hypertension	1020/2544 (40·1%)	1100/2518 (43·7%)
	Previous acute myocardial infarction	253/2553 (9·9%)	265/2530 (10·5%)
	Hypercholesterolaemia	682/2503 (27·2%)	696/2490 (28%)
	Medically treated diabetes	265/2557 (10·4%)	302/2534 (11·9%)
	Family history of ischaemic heart disease	819/2387 (34·3%)	853/2337 (36·5%)
Medications at admission for PPCI			
	Sulfonylurea	62/2519 (2·5%)	61/2496 (2·4%)
	Metformin	196/2518 (7·8%)	221/2499 (8·8%)
	Insulin	74/2548 (2·9%)	81/2533 (3·2%)
	Clopidogrel	85/2548 (3·3%)	98/2528 (3·9%)
	Ticagrelor	52/2549 (2·0%)	62/2527 (2·5%)
	Prasugrel	4/2546 (0·2%)	3/2528 (0·1%)
	Statins	652/2547 (25·6%)	665/2526 (26·3%)
	Aspirin	456/2548 (17·9%)	485/2528 (19·2%)
	β blockers	384/2539 (15·1%)	407/2525 (16·1%)
	Angiotensin-converting enzyme inhibitors or angiotensin receptor blockers	456/2541 (17·9%)	466/2525 (18·5%)
Blood pressure at randomisation, mm Hg			
	Systolic	131 (24)	131 (24)
	Diastolic	77 (15)	76 (15)
Killip class at randomisation			
	I	2462/2568 (95·9%)	2435/2546 (95·6%)
	II	75/2568 (2·9%)	75/2546 (2·9%)
	III	14/2568 (0·5%)	11/2546 (0·4%)
	IV (including cardiogenic shock)	17/2568 (0·7%)	25/2546 (1·0%)
**Procedural details**			
Number of complete cycles of remote ischaemic conditioning administered			
	1	NA	37/2246 (1·6%)
	2	NA	40/2246 (1·8%)
	3	NA	142/2246 (6·3%)
	4	NA	2027/2246 (90·2%)
TIMI flow grade at admission			
	0	1513/2227 (67·9%)	1478/2203 (67·1%)
	1	154/2227 (6·9%)	145/2203 (6·6%)
	2	218/2227 (9·8%)	225/2203 (10·2%)
	3	342/2227 (15·4%)	355/2203 (16·1%)
Symptom to balloon time, min		177 (128–279)	178 (130–278)
First medical contact to balloon time, min		102 (82–126)	103 (83–128)
Infarct-related coronary artery			
	Left anterior descending	974/2258 (43·1%)	911/2226 (40·9%)
	Circumflex	297/2258 (13·2%)	298/2226 (13·4%)
	Right coronary	985/2258 (43·6%)	1014/2226 (45·6%)
	Other	2/2258 (0·1%)	3/2226 (0·1%)
Number of vessels with clinically significant disease			
	0	1/2258 (<0·1%)	2/2227 (0·1%)
	1	1314/2258 (58·2%)	1274/2227 (57·2%)
	2	659/2258 (29·2%)	641/2227 (28·8%)
	3	284/2258 (12·6%)	310/2227 (13·9%)
Stenting of culprit lesion by PPCI		2104/2258 (93·2%)	2080/2226 (93·4%)
Aspiration thrombectomy		560/2242 (24·8%)	553/2209 (24·8%)
Supplementary staged PPCI		291/2258 (12·9%)	261/2227 (11·7%)
Supplementary staged coronary artery bypass grafting		52/2258 (2·3%)	62/2227 (2·8%)
TIMI flow grade after procedure			
	0	27/2223 (1·2%)	26/2200 (1·2%)
	1	20/2223 (0·9%)	9/2200 (0·4%)
	2	112/2223 (5·0%)	86/2200 (3·9%)
	3	2064/2223 (92·8%)	2079/2200 (94·5%)
Medications given in relation to PPCI			
	Opioids*	549/1054 (52·1%)	522/1040 (50·2%)
	Heparin	1904/2255 (84·4%)	1893/2224 (85·1%)
	Aspirin	2147/2255 (95·2%)	2129/2221 (95·9%)
	Clopidogrel	610/2256 (27·0%)	573/2226 (25·7%)
	Ticagrelor	1551/2257 (68·7%)	1554/2227 (69·8%)
	Prasugrel	103/2256 (4·6%)	97/2226 (4·4%)
	Nitrates	1758/2222 (79·1%)	1693/2185 (77·5%)
	Glycoprotein IIb/IIIa inhibitor	450/2250 (20·0%)	404/2223 (18·2%)
	Bivalirudin	505/2250 (22·4%)	489/2224 (22·0%)
	Cangrelor*	134/1088 (12·3%)	127/1085 (11·7%)

Data are n/N (%), mean (SD), or median (IQR). Numbers of missing data are provided in appendix 1 (pp 37–39). PPCI=primary percutaneous coronary intervention. NA=not applicable.

* Data collected only in the CONDI-2 trial.

**Figure 2:**
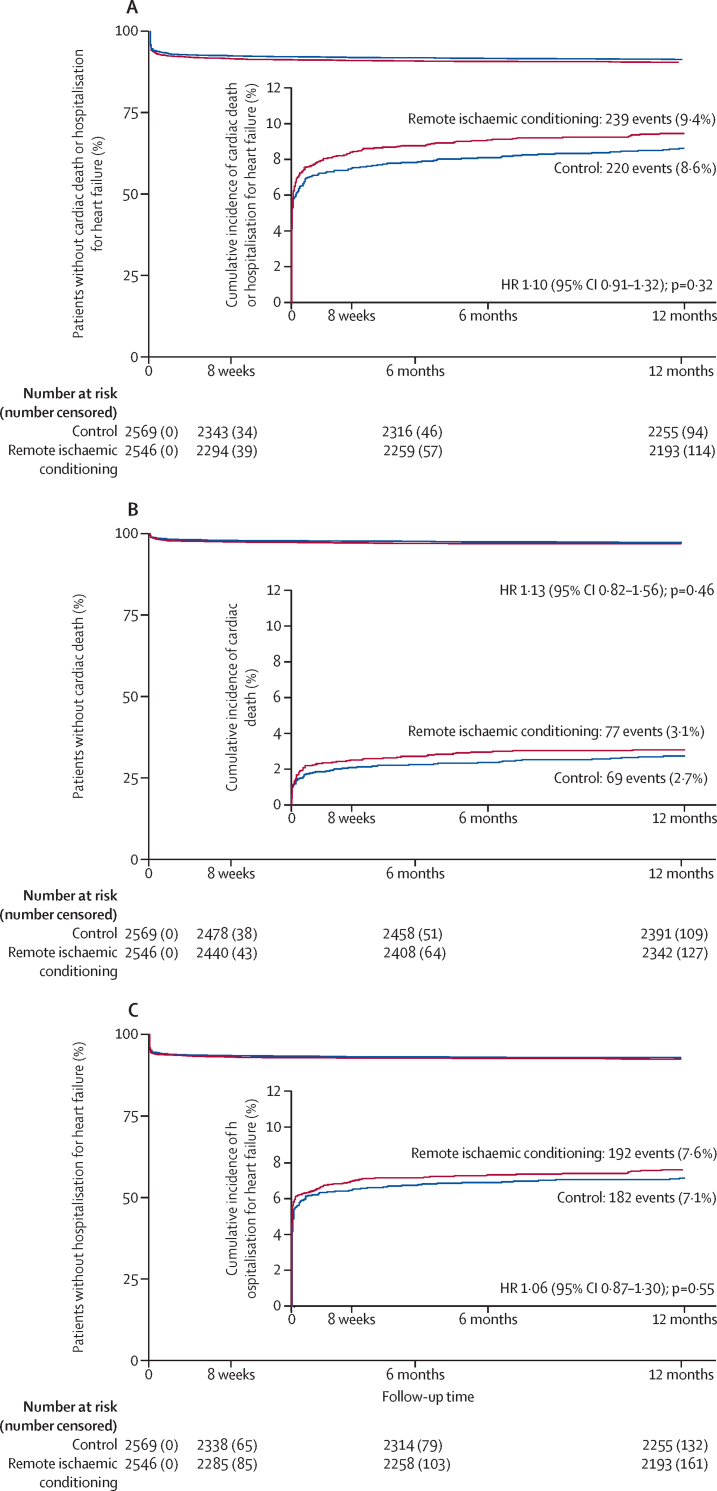
12-month cumulative incidence of combined cardiac death or hospitalisation for heart failure (A), cardiac death (B), and hospitalisation for heart failure (C) in the intention-to-treat population HR=hazard ratio.

There was no evidence of difference between the control group (8·6% [n=220]) and the remote ischaemic conditioning group (9·4% [n=239]) with respect to the combined primary end point of cardiac death or hospitalisation for heart failure at 12 months (hazard ratio [HR] 1·10; 95% CI 0·91–1·32; p=0·32; table 2, figure 2). Similarly, the control and remote ischaemic conditioning groups did not differ with regard to the individual components of cardiac death or hospitalisation for heart failure at 12 months (table 2, figure 2). The primary and secondary outcomes in CONDI-2 and ERIC-PPCI were similar, with no effect of remote ischaemic conditioning seen in either study (appendix 1 pp 25–30, 32–37).

**Table 2:** Primary and secondary outcomes in the intention-to-treat population

		Outcomes*		**Treatment effect**†**(95% CI)**	**p value**
		Control group (n=2569)	Remote ischaemic conditioning group (n=2546)		
**Time-to-event outcomes**					
Combined cardiac death or hospitalisation for heart failure within 12 months (primary outcome)		220 (8·6%)	239 (9·4%)	1·10 (0·91–1·32)	0·32
	Cardiac death within 12 months	69 (2·7%)	77 (3·1%)	1·13 (0·82–1·56)	0·46
	Hospitalisation for heart failure within 12 months	182 (7·1%)	192 (7·6%)	1·06 (0·87–1·30)	0·55
Major cardiovascular and cerebral adverse events‡ within 12 months		197 (7·8%)	212 (8·4%)	1·09 (0·90–1·32)	0·38
	All-cause death within 12 months	100 (3·9%)	122 (4·8%)	1·24 (0·95–1·61)	0·11
	Reinfarction within 12 months	43 (1·7%)	38 (1·5%)	0·89 (0·58–1·38)	0·61
	Unplanned revascularisation within 12 months	61 (2·4%)	61 (2·5%)	1·02 (0·71–1·45)	0·93
	Stroke within 12 months	21 (0·8%)	23 (0·9%)	1·11 (0·61–2·00)	0·73
Combined hospitalisation for heart failure or cardiac death within 30 days		185 (7·2%)	204 (8·0%)	1·11 (0·91–1·36)	0·29
	Cardiac death within 30 days	47 (1·8%)	59 (2·3%)	1·27 (0·87–1·86)	0·22
	Hospitalisation for heart failure within 30 days	162 (6·3%)	171 (6·8%)	1·06 (0·86–1·32)	0·57
Major cardiovascular and cerebral adverse events‡ within 30 days		97 (3·8%)	109 (4·3%)	1·14 (0·87–1·50)	0·35
	All-cause death within 30 days	52 (2·0%)	63 (2·5%)	1·23 (0·85–1·77)	0·27
	Reinfarction within 30 days	21 (0·8%)	19 (0·8%)	0·92 (0·49–1·70)	0·78
	Unplanned revascularisation within 30 days	29 (1·1%)	29 (1·2%)	1·02 (0·61–1·70)	0·95
	Stroke within 30 days	7 (0·3%)	10 (0·4%)	1·44 (0·55–3·80)	0·46
Implantable cardioverter-defibrillator implantation within 12 months		28 (1·1%)	37 (1·5%)	1·34 (0·82–2·18)	0·24
**Event frequency outcomes**					
Number of repeat episodes of hospitalisation for heart failure within 12 months		0·09 (0·36)	0·09 (0·34)	1·00 (0·81–1·24)	1·00
Number of repeat episodes of hospitalisation for heart failure or cardiac death within 12 months		0·11 (0·43)	0·12 (0·41)	1·03 (0·85–1·25)	0·77

* Data are Kaplan-Meier estimates of the n (%) of patients with the outcome at the specified timepoint (for time-to-event outcomes), or mean (SD; for event frequency outcomes).

† Data are hazard ratios (for time-to-event outcomes) or ratio of means (for event frequency outcomes), for treatment group versus control group.

‡ Composite of all-cause death, reinfarction, unplanned revascularisation, and stroke.

**Figure 3:**
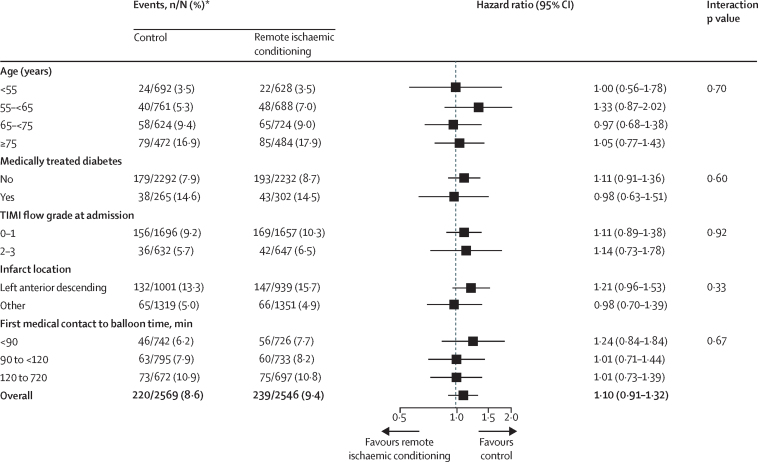
Forest plot for prespecified subgroup analyses of the primary endpoint in the intention-to-treat population *Kaplan-Meier estimates of the number of patients with hospitalisation for heart failure or cardiac death within 12 months.

Prespecified subgroup analyses showed no difference in the effect of remote ischaemic conditioning on the primary outcome by age, presence of diabetes, pre-PPCI TIMI flow grade, time from first medical contact to PPCI, or infarct location (figure 3). The results of the per-protocol analysis were very similar to those of the intention-to-treat analysis for the primary outcome (appendix 1 p 24), with the primary combined endpoint within 12 months occurring in 179 (9·0%) participants in the remote ischaemic conditioning group versus 178 (8·1%) in the control group (HR 1·11 [95% CI 0·90–1·36], p=0·35).

With respect to major cardiovascular and cerebral adverse events within 12 months of follow-up, no difference was observed between the remote ischaemic conditioning group and the control group, for either the combined outcome or the individual components (table 2).

No differences between the control and remote ischaemic conditions group were observed with regard to cardiac death or hospitalisation for heart failure (both combined and individually) within 30 days, major cardiovascular and cerebral adverse events (or the individual components of this outcome) after 30 days, or incidence of implantable cardioverter-defibrillator implantation within 12 months (table 2). Numbers of repeat episodes of hospitalisation for heart failure, or repeat episodes of hospitalisation for heart failure plus cardiac death were also similar between groups (table 2). Secondary endpoints were very similar between the CONDI-2 and ERIC-PPCI trials (appendix 1 pp 45, 57).

The AUC for high-sensitivity troponin T did not differ between the control group (median 91·9 ng × h/mL [IQR 37·6–185·6], n=1330) and the remote ischaemic conditioning group (110·3 ng × h/mL [48·0–218·5], n=1332; ratio of means 1·05 [95% CI 0·92–1·18], p=0·48; appendix 1 p 66).

No unexpected adverse events related to the trial treatment were reported. Skin petechiae and transient pain or paraesthesia were considered expected adverse events of remote ischaemic conditioning. In the remote ischaemic conditioning group, skin petechiae were reported for 72 (2·8%) patients and transient pain or paraesthesia was reported for 147 (5·8%) patients. There were no withdrawals due to adverse events.

## Discussion

In this large, appropriately powered, prospective, international, multicentre trial, we found no clinically meaningful beneficial effects of remote ischaemic conditioning administered as an adjunct to PPCI on clinical outcomes (cardiac death or hospitalisation for heart failure) at 12 months in patients with STEMI when compared with PPCI alone. Furthermore, remote ischaemic conditioning had no effect on major secondary endpoints, including myocardial infarct size assessed by cardiac biomarkers.

These findings are in direct contrast to previously published clinical studies of remote ischaemic conditioning in patients with STEMI that reported increased myocardial salvage (on nuclear myocardial perfusion imaging and cardiac MRI) and reductions in myocardial infarct size (assessed by cardiac biomarkers and cardiac imaging),^5^, ^6^, ^7^, ^8^, ^9^, ^10^, ^11^, ^12^ although not all studies have been positive for these endpoints.^12^ In our initial proof-of-concept CONDI-1 trial, no statistically significant reduction in myocardial infarct size (assessed by cardiac biomarkers) was observed despite an increase in myocardial salvage.^5^ Measurements of cardiac biomarkers might not be a sufficiently sensitive marker for detecting reductions in myocardial infarct size with cardioprotective therapies, and myocardial salvage might be a more sensitive marker to assess cardioprotection. In our cardiac biomarker analysis of a subset of 2662 patients, we found no effect of remote ischaemic conditioning on myocardial infarct size assessed by troponin T release, confirming no biological effects of remote ischaemic conditioning, a finding which is consistent with the observed absence of effect on clinical outcomes at 12 months. The lack of effect observed in our study raises the possibility that previously published, smaller studies, which used similar remote ischaemic conditioning protocols, were subject to type 1 errors.^5^, ^6^, ^7^, ^8^, ^9^, ^10^, ^11^, ^12^

Although myocardial infarct size is a known independent determinant of clinical outcomes post-PPCI in patients with STEMI, it is unclear whether a reduction in myocardial infarct size by a cardioprotective intervention applied as an adjunct to PPCI can be translated into improved clinical outcomes. The RIC-STEMI trial by Gaspar and colleagues^15^ also showed no reduction in myocardial infarct size (based on 48 h troponin I AUC), but still found improved clinical outcomes with remote ischaemic conditioning in terms of fewer cardiac deaths or hospitalisations for heart failure after a median follow-up time of 2·1 years (HR 0·35 [95% CI 0·15–0·78]). The unexpected and discordant effects of remote ischaemic conditioning on myocardial infarct size and clinical outcomes in the RIC-STEMI trial^15^ might have been due to a type 1 error, as only 516 patients with STEMI were randomly allocated, and the numbers of cardiac mortality events (three [1%] in the remote ischaemic conditioning group *vs* 11 [5%] in the control group) and hospitalisation for heart failure events (eight [3%] in the remote ischaemic conditioning group *vs* 17 [8%] in the control group) were small. An alternative explanation could be that the primary effect of remote ischaemic conditioning was on post-STEMI left ventricular remodelling rather than acute myocardial infarct size—although this mechanism would contradict experimental animal studies, which have clearly shown a beneficial effect of remote ischaemic conditioning on reducing acute myocardial infarct size. Notably, a clinical study investigating the cardioprotective effects of ischaemic postconditioning in patients with STEMI treated by PPCI showed no effect on myocardial infarct size, but still found reduced severity of adverse left ventricular remodelling at 1 year of follow-up, especially in patients with microvascular obstruction.^19^

Follow-up (median 3·8 years) of participants in the initial CONDI-1 trial^5^ (n=333 patients with STEMI) showed that increased myocardial salvage with remote ischaemic conditioning was associated with reduced frequencies of major cardiovascular and cerebral adverse events (17 [13·5%] patients) compared with the control group (32 [25·6%] patients).^13^ The LIPSIA CONDITIONING trial^10^ (n=696 patients with STEMI) showed improved myocardial salvage in patients who received PPCI combined with remote ischaemic conditioning and ischaemic postconditioning compared with either the control group (patients who received PPCI alone) or patients receiving ischaemic postconditioning with PPCI. Follow-up of LIPSIA CONDITIONING trial participants at a median of 3·6 years after the index event also revealed that major adverse cardiac events (cardiac death, reinfarction, and new congestive heart failure) were reduced in the group that received combined remote ischaemic conditioning and ischaemic postconditioning (23 [10·2%] patients) compared with the control group (37 [16·9%] patients)—an effect that was mainly driven by a reduced rate of heart failure.^14^ However, these studies were not prospectively designed or powered to assess clinical outcomes of remote ischaemic conditioning following STEMI. The present study is almost ten-times larger than any previous study and was adequately powered to address the efficacy of remote ischaemic conditioning on clinical outcomes.

The reasons for the failure of remote ischaemic conditioning to reduce myocardial infarct size and improve clinical outcomes in our study are not clear. One potential reason could relate to the remote ischaemic conditioning protocol itself. Although four 5-min cycles of upper arm cuff inflations and deflations applied before PPCI has been shown to increase the myocardial salvage index^5^ and reduce myocardial infarct size in previous clinical studies,^11^ whether this protocol is the most effective cardioprotective intervention in terms of cycle numbers and duration is not known. One experimental study in mice suggested that two cycles of remote ischaemic conditioning were ineffective, whereas four cycles of remote ischaemic conditioning reduced myocardial infarct size to a similar extent to that achieved with six or eight cycles.^20^ However, such phase 2 dose-response studies in humans are absent. The RIC-STEMI study,^15^ which showed no reduction in myocardial infarct size despite improved clinical outcomes, used a remote ischaemic conditioning stimulus comprising three cycles of inflation and deflation of a pneumatic cuff placed on the thigh, in contrast to our remote ischaemic conditioning stimulus that consisted of four cycles of inflation and deflation of a pneumatic cuff placed on the upper arm. The stimulus used in the RIC-STEMI trial might be expected to be more efficacious because of the greater ischaemic tissue burden, but this explanation would not account for the absence of effect on myocardial infarct size in the RIC-STEMI trial. Furthermore, a previous experimental study in mice showed that bilateral hindlimb remote ischaemic conditioning was no more efficacious at reducing myocardial infarct size than was unilateral limb remote ischaemic conditioning.^20^

The timing of the remote ischaemic conditioning protocol in relation to reperfusion by PPCI might also be important. Previous clinical studies have shown that remote ischaemic conditioning is effective at reducing myocardial infarct size when administered before PPCI (either in transit to the PPCI centre^5^ or on arrival at the hospital^11^), during reperfusion by PPCI, and even at onset of reperfusion.^7^ In our study, clinical outcomes did not differ on the basis of whether the remote ischaemic conditioning protocol was completed during transportation to the PPCI centre or on admission to hospital, where the remote ischaemic conditioning protocol was continued during reperfusion in most patients (appendix 1 pp 40–41). Furthermore, no differences in clinical outcomes were observed whether the full four cycles of the remote ischaemic conditioning protocol was completed before reperfusion or not (appendix 1 pp 40–41).

Comedications and comorbidities might affect the cardioprotective efficacy of remote ischaemic conditioning. In experimental studies, P2Y_12_ receptor antagonists confounded the cardioprotective effects of ischaemic conditioning,^21^, ^22^ although no evidence for remote ischaemic conditioning is available. In our trial, the P2Y_12_ receptor antagonist ticagrelor was administered to 69·8% of patients receiving remote ischaemic conditioning. We found no difference in clinical outcomes between the control group and the remote ischaemic conditioning group with respect to ticagrelor administration (appendix 1 p 41).

Experimental data have shown that age and presence of comorbidities, including diabetes, might attenuate the cardioprotective effects of ischaemic preconditioning and postconditioning.^23^ Whether these factors affected the cardioprotective efficacy of remote ischaemic conditioning in our study remains unclear; our prespecified subgroup analyses showed no differences in clinical outcomes between the control group and the remote ischaemic conditioning group with increasing age or presence of diabetes (figure 3).

The efficacy of cardioprotective interventions applied at reperfusion in patients with STEMI is closely related to myocardial infarct size and pre-PPCI TIMI flow, with the greatest benefit reported for patients with complete occlusion (pre-PPCI TIMI flow ≤1) due to infarcts in the left anterior descending artery.^5^ However, our prespecified subgroup analyses showed no differences in clinical outcomes according to pre-PPCI TIMI flow and infarct location (figure 3).

Remote ischaemic conditioning has shown mixed effects on outcomes in other clinical settings including cardiac surgery. Three large, prospective, multicentre, randomised controlled trials failed to find any beneficial effects of remote ischaemic conditioning on post-surgical clinical outcomes,^24^, ^25^, ^26^ possibly because of the confounding effects of propofol anaesthesia. Smaller studies of remote ischaemic conditioning in patients with stroke, renal transplantation, or undergoing elective PCI have suggested potential benefits.^27^, ^28^, ^29^ A clinical study in patients with STEMI treated by PPCI investigated the effects of remote ischaemic conditioning applied daily for 1 month (termed chronic remote ischaemic conditioning).^30^ This intervention showed no beneficial effects on post-infarct left ventricular remodelling, although it was only started on day 3 post-PPCI in that study, which might be too late to observe any beneficial effects.

A potential limitation of our study is the short follow-up time of 12 months post-STEMI for the primary outcome of cardiac death and hospitalisation for heart failure, which might have been too short to observe any effect of remote ischaemic conditioning on clinical outcomes.

In summary, the findings from our trial provide conclusive evidence that remote ischaemic conditioning offers no benefits on either myocardial infarct size or clinical outcomes at 12 months in patients with STEMI treated by PPCI. Unfortunately, remote ischaemic conditioning had been the most promising cardioprotective strategy for improving clinical outcomes following STEMI, and few other prospective options exist. As such, further studies are needed to identify novel cardioprotective targets and innovative approaches to cardioprotection such as combination multitarget therapy.^31^

## Data sharing

Requests for data collected for the study can be made to the co-corresponding authors and will be considered on an individual basis. Additional, related documents are immediately available (eg, study protocol, statistical analysis plan, informed consent form) and can be requested from the co-corresponding authors.
