# Supplementary material for: Effect of remote ischaemic conditioning on clinical outcomes in patients with acute myocardial infarction (CONDI-2/ERIC-PPCI): a single-blind randomised controlled trial
Source: Lancet. 2019 Oct 19;394(10207):1415–24. doi: 10.1016/S0140-6736(19)32039-2 (PMC6891239; doi:10.1016/S0140-6736(19)32039-2)
Supplement: Supplementary appendix 1 [file mmc1.pdf]

THE LANCET

Supplementary appendix

This appendix formed part of the original submission and has been peer reviewed. We post it as supplied by the authors.

Supplement to: Hausenloy DJ, Kharbanda RK, Møller UK, et al. Effect of remote ischaemic conditioning on clinical outcomes in patients with acute myocardial infarction (CONDI-2/ERIC-PPCI): a single-blind randomised controlled trial. *Lancet* 2019; published online September 6. http://dx.doi.org/10.1016/S0140-6736(19)32039-2.

Effect of remote ischaemic conditioning on clinical outcomes in patients with acute myocardial infarction (CONDI-2/ERIC-PPCI): a single-blind randomised controlled trial

Supplementary material

Table of Contents

**Page**	**2**	**Consort Checklist for trial**
**Page**	**4**	**Consort Checklist for abstract**
**Page**	**5**	**Consort Checklist for Harms**
**Page**	**6**	**Recruitment numbers by site**
**Page**	**7**	**Committee Members**
**Page**	**9**	**List of Investigators**
**Page**	**20**	**Supplementary Methods**
**Page**	**22**	**Supplementary Figures**
**Page**	**37**	**Supplementary Tables**
**Page**	**67**	**IDMC Charter for CONDI-2**
**Page**	**84**	**IDMC Charter for ERIC-PPCI**
**Page**	**105**	**Data handling procedures**
**Page**	**106**	**References**

CONSORT 2010 checklist of information to include when reporting a randomised trial

Section/Topic	Item No	Checklist item	Reported on page No
**Title and abstract**			
	1a	Identification as a randomised trial in the title	1
	1b	Structured summary of trial design, methods, results, and conclusions (for specific guidance see CONSORT for abstracts)	3
**Introduction**			
Background and objectives	2a	Scientific background and explanation of rationale	4
	2b	Specific objectives or hypotheses	4
**Methods**			
Trial design	3a	Description of trial design (such as parallel, factorial) including allocation ratio	4
	3b	Important changes to methods after trial commencement (such as eligibility criteria), with reasons	NA
Participants	4a	Eligibility criteria for participants	5
	4b	Settings and locations where the data were collected	4
Interventions	5	The interventions for each group with sufficient details to allow replication, including how and when they were actually administered	5
Outcomes	6a	Completely defined pre-specified primary and secondary outcome measures, including how and when they were assessed	6
	6b	Any changes to trial outcomes after the trial commenced, with reasons	NA
Sample size	7a	How sample size was determined	6
	7b	When applicable, explanation of any interim analyses and stopping guidelines	NA
Randomisation:			
Sequence generation	8a	Method used to generate the random allocation sequence	5
	8b	Type of randomisation; details of any restriction (such as blocking and block size)	5
Allocation concealment mechanism	9	Mechanism used to implement the random allocation sequence (such as sequentially numbered containers), describing any steps taken to conceal the sequence until interventions were assigned	5
Implementation	10	Who generated the random allocation sequence, who enrolled participants, and who assigned participants to interventions	5
Blinding	11a	If done, who was blinded after assignment to interventions (for example, participants, care providers, those assessing outcomes) and how	5

	11b	If relevant, description of the similarity of interventions	NA
Statistical methods	12a	Statistical methods used to compare groups for primary and secondary outcomes	6
	12b	Methods for additional analyses, such as subgroup analyses and adjusted analyses	6
**Results**			
Participant flow (a diagram is strongly recommended)	13a	For each group, the numbers of participants who were randomly assigned, received intended treatment, and were analysed for the primary outcome	19
	13b	For each group, losses and exclusions after randomisation, together with reasons	19
Recruitment	14a	Dates defining the periods of recruitment and follow-up	7
	14b	Why the trial ended or was stopped	NA
Baseline data	15	A table showing baseline demographic and clinical characteristics for each group	15
Numbers analysed	16	For each group, number of participants (denominator) included in each analysis and whether the analysis was by original assigned groups	17
Outcomes and estimation	17a	For each primary and secondary outcome, results for each group, and the estimated effect size and its precision (such as 95% confidence interval)	17
	17b	For binary outcomes, presentation of both absolute and relative effect sizes is recommended	17
Ancillary analyses	18	Results of any other analyses performed, including subgroup analyses and adjusted analyses, distinguishing pre-specified from exploratory	24
Harms	19	All important harms or unintended effects in each group (for specific guidance see CONSORT for harms)	8
**Discussion**			
Limitations	20	Trial limitations, addressing sources of potential bias, imprecision, and, if relevant, multiplicity of analyses	11
Generalisability	21	Generalisability (external validity, applicability) of the trial findings	9
Interpretation	22	Interpretation consistent with results, balancing benefits and harms, and considering other relevant evidence	9
**Other information**			
Registration	23	Registration number and name of trial registry	3
Protocol	24	Where the full trial protocol can be accessed, if available	5
Funding	25	Sources of funding and other support (such as supply of drugs), role of funders	7

CONSORT 2010 checklist for abstract of information to include

Item	Description	Reported on line number
Title	Identification of the study as randomized	1
Authors *	Contact details for the corresponding author	NA
Trial design	Description of the trial design (e.g. parallel, cluster, non-inferiority)	3
Methods		
Participants	Eligibility criteria for participants and the settings where the data were collected	3
Interventions	Interventions intended for each group	3
Objective	Specific objective or hypothesis	3
Outcome	Clearly defined primary outcome for this report	3
Randomization	How participants were allocated to interventions	3
Blinding (masking)	Whether or not participants, care givers, and those assessing the outcomes were blinded to group assignment	3
Results		
Numbers randomized	Number of participants randomized to each group	3
Recruitment	Trial status	3
Numbers analysed	Number of participants analysed in each group	3
Outcome	For the primary outcome, a result for each group and the estimated effect size and its precision	3
Harms	Important adverse events or side effects	3
Conclusions	General interpretation of the results	3
Trial registration	Registration number and name of trial register	3
Funding	Source of funding	3

**this item is specific to conference abstracts*

CONSORT checklist for Harms

Table 2. **Checklist of Items To Include When Reporting Harms in Randomized, Controlled Trials***

Standard CONSORT Checklist: Paper Section and Topic	Standard CONSORT Checklist: Item Number	Descriptor	Reported on page
Title and abstract	1	If the study collected data on harms and benefits, the title or abstract should so state.	1
Introduction			
Background	2	If the trial addresses both harms and benefits, the introduction should so state.	NA
Methods			
Participants	3		
Interventions	4		
Objectives	5		
Outcomes	6	List addressed adverse events with definitions for each (with attention, when relevant, to grading, expected vs. unexpected events, reference to standardized and validated definitions, and description of new definitions). Clarify how harms-related information was collected (mode of data collection, timing, attribution methods, intensity of ascertainment, and harms-related monitoring and stopping rules, if pertinent).	Protocol page 23
Sample size	7		
Randomization			
Sequence generation	8		
Allocation concealment	9		
Implementation	10		
Blinding (masking)	11		
Statistical methods	12	Describe plans for presenting and analyzing information on harms (including coding, handling of recurrent events, specification of timing issues, handling of continuous measures, and any statistical analyses).	Protocol page 23
Results			
Participant flow	13	Describe for each arm the participant withdrawals that are due to harms and their experiences with the allocated treatment.	*9*
Recruitment	14		
Baseline data	15		
Numbers analyzed	16	Provide the denominators for analyses on harms.	
Outcomes and estimation	17	Present the absolute risk per arm and per adverse event type, grade, and seriousness, and present appropriate metrics for recurrent events, continuous variables, and scale variables, whenever pertinent.†	9
Ancillary analyses	18	Describe any subgroup analyses and exploratory analyses for harms.†	
Adverse events	19		
Discussion			
Interpretation	20	Provide a balanced discussion of benefits and harms with emphasis on study limitations, generalizability, and other sources of information on harms.‡	
Generalizability	21		
Overall evidence	22		NA

* This proposed extension for harms includes 10 recommendations that correspond to the original CONSORT checklist.
† Descriptors refer to items 17, 18, and 19.

Recruitment by site in CONDI-2 trial

Inclusion site	Number of patients included
Aarhus University Hospital	1354
Rigshospitalet, Copenhagen	287
Odense University Hospital	104
Aalborg University Hospital	301
Clinical Centre of Serbia, Belgrade	206
Hospital Universitario de Cabueñes, Asturias	307

Recruitment by site in ERIC-PPCI trial

Inclusion site	Number of patients included
Cumberland Infirmary, Carlisle	128
Barts Heart Centre	68
Basildon University Hospital	60
John Radcliffe Hospital	146
Northern General Hospital	63
Leeds General Infirmary	420
King's College Hospital	132
Manchester Royal Infirmary	97
Norfolk and Norwich	105
New Cross, Wolverhampton	79
Royal Free Hospital	149
St Thomas' Hospital	50
Lister Hospital, Stevenage	130
Queen Alexandra, Portsmouth	384
Lincoln County	71
Papworth Hospital	50
Victoria Hospital, Blackpool	118
Royal Bournemouth	93
Royal Sussex, Brighton	93
Coventry University Hospital	30
Royal Stoke University Hospital	107
Birmingham Heartlands	56
Kettering General	66
Hammersmith Hospital	22
Bristol Royal Infirmary	43
William Harvey Hospital	30

COMMITTEE MEMBERS

ERIC-PPCI Trial Steering Committee

- Rob Henderson (Chair, Nottingham University Hospitals NHS Trust, UK)
- Derek J Hausenloy (Chief Investigator, ERIC-PPCI Bart's Heart Center, UK)
- Tim Clayton (Co-Principal Investigator/Senior Medical Statistician, London School of Hygiene and Tropical Medicine Clinical Trials Unit)
- Derek Yellon (Co-Principal Investigator, University College London, UK)
- Rajesh Kharbanda (Co-Principal Investigator, John Radcliffe Hospital, UK)
- Hans Erik Bøtker (Chief Investigator, Danish CONDI-2 Trial, Aarhus University, Denmark)
- Rod Stables (Liverpool Heart and Chest Hospital, UK)
- Simon Redwood (St Thomas' Hospital, UK)
- Michael Marber (St Thomas' Hospital, UK)
- Rosemary Knight (Senior Trials Manager, London School of Hygiene and Tropical Medicine Clinical Trials Unit)
- Alexander Perkins (Trials Manager, London School of Hygiene and Tropical Medicine Clinical Trials Unit)
- Richard Evans (Senior Trials Manager, London School of Hygiene and Tropical Medicine Clinical Trials Unit)
- Matthew Dodd (Medical Statistician, London School of Hygiene and Tropical Medicine Clinical Trials Unit)
- Paul Hambley (Previous PPCI patient)
- Alan Berry (Previous PPCI patient)

CONDI-2 Trial Steering Committee

- Andrew Redington (Chair, Cincinnati Children's Hospital Medical Center, Cincinnati, Ohio, US)
- Hans Erik Bøtker (Chief Investigator, CONDI-2 Trial, Aarhus University Hospital, Denmark)
- Derek J Hausenloy (Chief Investigator, ERIC-PPCI Trial, Bart's Heart Center, UK)
- Michael Rahbek Schmidt (Aarhus University Hospital, Denmark)
- Thomas Engstrøm (Rigshospitalet, University of Copenhagen, Denmark)
- Lisette Okkels Jensen (Odense University Hospital, Denmark)
- Jens Aarøe (Aalborg University Hospital, Denmark)
- Ulla Kristine Møller (Senior Trial Manager, Aarhus University Hospital, Denmark)
- Borja Ibanez (Centro Nacional de Investigaciones Cardiovasculares (CNIC), Madrid, Spain)
- José Manuel García Ruiz (Hospital Universitario de Cabueñes Gijón, Hospital Universitario Central de Asturias, Spain)
- Goran Stankovic (Clinical Center of Serbia, Belgrade, Serbia)
- Dejan Milasinovic (Clinical Center of Serbia, Belgrade, Serbia)

ERIC-PPCI Data Monitoring Committee

- Colin Berry (Chair, University of Glasgow, UK)
- Andrew Copas (Reader in Statistics, MRC Clinical Trials Unit, UK)
- Tom Meade (Professor Emeritus of Epidemiology, London School of Hygiene & Tropical Medicine, UK)
- Jenny Nicholas (Associate Professor of Medical Statistics, London School of Hygiene and Tropical Medicine, UK).

CONDI-2 Data Monitoring Committee

- Henning Kelbæk (Chair, Zealand University Hospital, Roskilde, Denmark)
- Hector Bueno (Spanish National Centre for Cardiovascular Research, Spain)
- Morten Madsen (Aarhus University Hospital, Denmark)

Joint CONDI-2/ERIC-PPCI Event Validation Committee

- Kristian Thygesen (Chair, Aarhus University Hospital, Denmark)
- Henning Rud Andersen (Aarhus University Hospital, Denmark)

- Paul von Weitzel-Mudersbach (Aarhus University Hospital, Denmark)
- Grethe Andersen (Aarhus University Hospital, Denmark)
- Andrew Ludman (Royal Devon and Exeter NHS Foundation Trust, UK)
- Nick Cruden (Royal Infirmary Edinburgh, UK)
- Dragan Topic (Institute for Cardiovascular Diseases Dedinje, Serbia)
- Zlatko Mehmedbegovic (Clinical Center of Serbia, Belgrade, Serbia)
- José Miguel Vegas Valles (Hospital Universitario de Cabueñes Gijón, Spain)
- Jesus María de la Hera Galarza (Hospital Universitario Central de Asturias, Spain)

London School Hygiene and Tropical Medicine Clinical Trials Unit, Other Staff

- Steven Robertson
- Laura Van Dyck
- Rebecca Chu
- Josenir Astarci
- Zahra Jamal
- Daniel Hetherington
- Lucy Collier

LIST OF INVESTIGATORS

CONDI-2

Aarhus University Hospital, Department of Cardiology, Aarhus N, Denmark
- Anders Junker
- Anne Kaltoft
- Christian Juhl Terkelsen
- Evald Høj Christiansen
- Lars Jakobsen
- Steen Carstensen
- Steen Dalby Kristensen
- Troels Thim
- Karin Møller Pedersen
- Mette Tidemand Korsgaard

Rigshospitalet, Department of Cardiology, University of Copenhagen, Copenhagen, Denmark
- Allan Iversen
- Erik Jørgensen
- Francis Joshi
- Frants Pedersen
- Hans Henrik Tilsted
- Karam Alzuhairi
- Kari Saunamäki
- Lene Holmvang
- Ole Ahlehof
- Rikke Sørensen
- Steffen Helqvist
- Bettina Løjmand Mark

Aalborg University Hospital, Department of Cardiology, Aalborg, Denmark
- Anton Boel Villadsen
- Bent Raungaard
- Leif Thuesen
- Martin Kirk Christiansen
- Philip Freeman
- Svend Eggert Jensen
- Charlotte Schmidt Skov

Odense University Hospital, Department of Cardiology Odense, Denmark
- Ahmed Aziz
- Henrik Steen Hansen
- Julia Ellert
- Karsten Veien
- Knud Erik Pedersen
- Knud Nørregård Hansen
- Ole Ahlehoff
- Helle Cappelen

Prehospital Emergency Medical Services, Denmark
- Daniel Wittrock
- Poul Anders Hansen
- Jens Peter Ankersen
- Kim Witting Hedegaard
- John Kempel
- Henning Kaus
- Dennis Erntgaard

- Danny Mejsner Pedersen
- Matthias Giebner

National prehospital Emergency Helicopter Services, Denmark
- Troels Martin Hansen

Clinical Center of Serbia, Department for Diagnostic and Catheterization Laboratories, Clinic for Cardiology, Faculty of Medicine, University of Belgrade, Belgrade, Serbia
- Mina Radosavljevic-Radovanovic
- Maja Prodanovic
- Lidija Savic
- Marijana Pejic
- Dragan Matic
- Ana Uscumlic
- Ida Subotic
- Ratko Lasica
- Vladan Vukcevic

Hospital Universitario de Cabueñes Gijón and Hospital Universitario Central de Asturias, Oviedo, Spain
- Alfonso Suárez
- Beatriz Samaniego
- César Morís
- Eduardo Segovia
- Ernesto Hernández
- Iñigo Lozano
- Isaac Pascual
- Jose M. Vegas-Valle
- José Rozado
- Juan Rondán
- Pablo Avanzas
- Raquel del Valle
- Remigio Padrón

Emergency Medical Services, SAMU, Asturias, Spain
- Alfonso García-Castro
- Amalia Arango
- Ana B. Medina-Cameán
- Ana I. Fente
- Ana Muriel-Velasco
- Ángeles Pomar-Amillo
- César L. Roza
- César M. Martínez-Fernández
- Covadonga Buelga-Díaz
- David Fernández-Gonzalo
- Elena Fernández
- Eloy Díaz-González
- Eugenio Martinez-González
- Fernando Iglesias-Llaca
- Fernando M. Viribay
- Francisco J. Fernández-Mallo
- Francisco J. Hermosa
- Ginés Martínez-Bastida
- Javier Goitia-Martín
- José L.Vega-Fernández
- Jose M. Tresguerres
- Juan A. Rodil-Díaz
- Lara Villar-Fernández

- Lucía Alberdi
- Luis Abella-Ovalle
- Manuel de la Roz
- Marcos Fernández-Carral
- María C. Naves
- María C. Peláez
- María D. Fuentes
- María García-Alonso
- María J. Villanueva
- María S. Vinagrero
- María Vázquez-Suárez
- Marta Martínez-Valle
- Marta Nonide
- Mónica Pozo-López
- Pablo Bernardo-Alba
- Pablo Galván-Núñez
- Polácido J. Martínez-Pérez
- Rafael Castro
- Raquel Suárez-Coto
- Raquel Suárez-Noriega
- Rocío Guinea
- Rosa B. Quintana
- Sara de Cima
- Segundo A. Hedrera
- Sonia I. Laca
- Susana Llorente-Álvarez
- Susana Pascual
- Teodorna Cimas

ERIC-PPCI

St Bartholomew’s Hospital, London, UK

- Anthony Mathur
- Eleanor McFarlane-Henry
- Gerry Leonard
- Jessry Veerapen
- Mark Westwood
- Martina Colicchia
- Mary Prossora
- Mervyn Andiapen
- Saidi Mohiddin
- Valentina Lenzi
- Jun Chong
- Rohin Francis

Royal Bournemouth Hospital, Bournemouth, UK

- Amy Pine
- Caroline Jamieson-Leadbitter
- Debbie Neal
- J.Din
- Jane McLeod
- Josh Roberts
- Karin Polokova
- Kristel Longman
- Lucy Penney
- Nicki Lakeman
- Nicki Wells
- Oliver Hopper
- Paul Coward
- Peter O'Kane
- Ruth Harkins
- Samantha Guyatt
- Sarah Kennard
- Sarah Orr
- Stephanie Horler
- Steve Morris
- Tom Walvin
- Tom Snow

Leeds General Infirmary, Leeds, UK

- Michael Cunnington
- Amanda Burd
- Anne Gowing
- Arvindra Krishnamurthy
- Charlotte Harland
- Derek Norfolk
- Donna Johnstone
- Hannah Newman
- Helen Reed
- James O'Neill
- John Greenwood
- Josephine Cuxton
- Julie Corrigan
- Kathryn Somers
- Michelle Anderson
- Natalie Burtonwood

- Petra Bijsterveld
- Richard Brogan
- Tony Ryan
- Vivek Kodoth

Lister Hospital, Stevenage, UK
- Arif Khan
- Deepti Sebastian
- Diana Gorog
- Georgina Boyle
- Lucy Shepherd
- Mahmood Hamid
- Mohamed Farag
- Nicholas Spinthakis
- Paulina Waitrak
- Phillipa De Sousa
- Rishma Bhatti
- Victoria Oliver
- Siobhan Walshe
- Toral Odedra
- Ying Gue
- Rahim Kanji

Norfolk and Norwich University Hospital, Norwich, UK
- Alisdair Ryding
- Amanda Ratcliffe
- Angela Merrick
- Carol Horwood
- Charlotte Sarti
- Clint Maart
- Donna Moore
- Francesca Dockerty
- Karen Baucutt
- Louise Pitcher
- Mary Ilsley
- Millie Clarke
- Rachel Germon
- Sara Gomes
- Thomas Clare
- Sunil Nair
- Jocasta Staines
- Susan Nicholson
- Oliver Watkinson
- Ian Gallagher
- Faye Nelthorpe

William Harvey Hospital, Ashford, UK
- Janine Musselwhite
- Konrad Grosser
- Leah Stimson
- Michelle Eaton
- Richard Heppell
- Sharon Turney
- Victoria Horner
- Natasha Schumacher
- Angela Moon
- Paula Mota

- Joshua O’Donnell

University Hospitals Coventry & Warwickshire, Coventry, UK
- Abeesh Sadasiva Panicker
- Anntoniette Musa
- Luke Tapp
- Suresh Krishnamoorthy
- Valerie Ansell
- Danish Ali
- Samantha Hyndman
- Prithwish Banerjee
- Martin Been

Royal Sussex County Hospital, Brighton, UK
- Ailie Mackenzie
- Andrew McGregor
- David Hildick-Smith
- Felicity Champney
- Fiona Ingoldby
- Kirstie Keate
- Lorraine Bennett
- Nicola Skipper
- Sally Gregory
- Scott Harfield

Northern General Hospital, Sheffield, UK
- Alexandra Mudd
- Christopher Wragg
- David Barmby
- Ever Grech
- Ian Hall
- Janet Middle
- Joann Barker
- Joyce Fofie
- Julian Gunn
- Kay Housley
- Laura Cockayne
- Louise Weatherlley
- Nana Theodorou
- Nigel Wheeldon
- Pene Fati
- Robert F.Storey
- James Richardson
- Javid Iqbal,
- Zul Adam
- Sarah Brett
- Michael Agyemang
- Cecilia Tawiah

Kettering General Hospital, Kettering, UK
- Kai Hogrefe
- Prashanth Raju
- Christine Braybrook
- Jay Gracey
- Molly Waldron
- Rachael Holloway
- Senem Burunsuzoglu

- Sian Sidgwick
- Simon Hetherington
- Charmaine Beirnes
- Olga Fernandez
- Nicoleta Lazar

King's College Hospital, London, UK

- Abigail Knighton
- Amrit Rai
- Amy Hoare
- Ian Webb
- Jonathan Breeze
- Katherine Martin
- Michelle Andrews
- Sheetal Patale

New Cross Hospital, Wolverhampton, UK

- Amy Bennett
- Andrew Smallwood
- Elizabeth Radford
- James Cotton
- Joe Martins
- Lauren Wallace
- Sarah Milgate
- Shahzad Munir
- Stella Metherell
- Victoria Cottam

Royal Stoke University Hospital, Stoke-on-Trent, UK

- Ian Massey
- Jane Copestick
- Jane Delaney
- Jill Wain
- Kully Sandhu
- Lisa Emery
- Robert Butler
- Charlotte Hall

Bristol Royal Infirmary, Bristol, UK

- Chiara Bucciarelli-Ducci
- Rissa Besana
- Jodie Hussein
- Sheila Bell
- Abby Gill

Royal Papworth Hospital, Cambridge, UK

- Emily Bales
- Gary Polwarth
- Clare East
- Ian Smith
- Joana Oliveira
- Saji Victor
- Sarah Woods
- Stephen Hoole

Basildon University Hospital, Basildon, UK

- Angelo Ramos

- Annaliza Sevillano
- Anne Nicholson
- Ashley Solieri
- Emily Redman
- Jean Byrne
- Joan Joyce
- Joanne Riches
- John Davies
- Kezia Allen
- Louie Saclot
- Madelaine Ocampo
- Mark Vertue
- Natasha Christmas
- Raiji Koothoor
- Reto Gamma
- Wilson Alvares
- Stacey Pepper

Hammersmith Hospital, London, UK

- Barbara Kobson
- Christy Reeve
- Iqbal Malik
- Emma Chester
- Heidi Saunders
- Idah Mojela
- Joanna Smee
- Justin Davies
- Nina Davies
- Piers Clifford
- Priyanthi Dias
- Ramandeep Kaur
- Silvia Moreira
- Yousif Ahmad
- Lucy Tomlinson
- Clare Pengelley
- Amanda Bidle
- Sharon Spence
- Rasha Al-Lamee
- Urmila Phuyal
- Hakam Abbass
- Tuhina Bose
- Rebecca Elliott

Birmingham Heartlands Hospital, Birmingham, UK

- Aboo Foundun
- Alan Chung
- Beth Freestone
- Dr Kaeng Lee
- Dr Mohamed Elshiekh
- George Pulikal
- Gurbir Bhatre
- James Douglas
- Lee Kaeng
- Mike Pitt
- Richard Watkins
- Simrat Gill

John Radcliffe Hospital, Oxford, UK

- Amy Hartley
- Andrew Lucking
- Berni Moreby
- Damaris Darby
- Ellie Corps
- Georgina Parsons
- Gianluigi De Mance
- Gregor Fahrai
- Jenny Turner
- Jeremy Langrish
- Lisa Gaughran
- Mathias Wolyrum
- Mohammed Azkhalil
- Rachel Bates
- Rachel Given
- Rajesh Kharbanda
- Rebecca Douthwaite
- Steph Lloyd
- Stephen Neubauer
- Deborah Barker

Queen Alexandra Hospital, Portsmouth, UK

- Ali Dana
- Anne Suttling
- Charlotte Turner
- Clare Smith
- Colin Longbottom
- David Ross
- Denise Cunliffe
- Emily Cox
- Helena Whitehead
- Karen Hudson
- Leslie Jones
- Martin Drew
- Nicholas Chant
- Peter Haworth
- Robert Capel
- Rosalynn Austin
- Serena Howe
- Trevor Smith
- Alex Hobson
- Philip Strike
- Huw Griffiths
- Brijesh Anantharam
- Pearse Jack
- Emma Thornton

Cumberland Infirmary, Carlisle, UK

- Adrian Hodgson
- Alan Jennison
- Anna McSkeane
- Bethany Smith
- Caroline Shaw
- Chris Leathers
- Elissa Armstrong
- Gayle Carruthers

- Holly Simpson
- Jan Smith
- Jeremy Hodierne
- Julie Kelly
- Justin Barclay
- Kerry Scott
- Lisa Gregson
- Louise Buchanan
- Louise McCormick
- Madhusudhan Varma
- Nicci Kelsall
- Rachel Mcarthy
- Rebecca Taylor
- Rebecca Thompson
- Rhidian Shelton
- Roger Moore
- Sharon Tomlinson
- Sunil Thambi
- Theresa Cooper
- Trevor Oakes
- Zakhira Deen
- Chris Relph
- Scott prentice
- Lorna Hall

Lincoln County Hospital, Lincoln, UK

- Angela Dillon
- Deborah Meadows
- Emma Frank
- Helene Markham-Jones
- Isobel Thomas
- Joanne Gale
- Joanne Denman
- Joanne Gale
- John O'Connor
- Julia Hindle
- Karen Jackson-Lawrence
- Karen Warner
- Kelvin Lee
- Robert Upton
- Ruth Elston
- Sandra Lee
- Vinod Venugopal

Blackpool Victoria Hospital, Blackpool, UK

- Amanda Finch
- Catherine Fleming
- Charlene Whiteside
- Chris Pemberton
- Conor Wilkinson
- Deepa Sebastian
- Ella Riedel
- Gaia Giuffrida
- Gillian Burnett
- Helen Spickett
- James Glen
- Janette Brown

- Lauren Thornborough
- Lauren Pedley
- Maureen Morgan
- Natalia Waddington
- Oliver Brennan
- Ranjit More
- Rebecca Brady
- Stephen Preston

Royal Free Hospital, London, UK
- Chris Loder
- Ionela Vlad
- Julia Laurence
- Angelique Smit
- Kirsty Dimond
- Michelle Hayes
- Loveth Paddy
- Jacolene Crause
- Nadifa Amed
- Priya Kaur-Babooa
- Roby Rakhit
- Tushar Kotecha
- Hossam Fayed
- Rohin Francis

St Thomas' Hospitalm London, UK
- Antonis Pavlidis
- Bernard Prendergast
- Brian Clapp
- Divaka Perara
- Emma Atkinson
- Howard Ellis
- Karen Wilson
- Kirsty Gibson
- Megan Smith
- Muhammed Zeeshan Khawaja
- Ruth Sanchez-Vidal
- Simon Redwood
- Sophie Jones
- Aoife Tipping

Manchester Royal Infirmary, Manchester, UK
- Anu Oommen
- Dr Cara Hendry
- DR Fazin Fath-Orboubadi
- Hannah Phillips
- Laurel Kolakaluri
- Martin Sherwood
- Sarah Mackie
- Shilpa Aleti
- Thabitha Charles
- Liby Roy

SUPPLEMENTARY METHODS

Definition of cardiac death: All deaths for which there was no clinical or post-mortem evidence of non-cardiac etiology.

Definition of hospitalization for heart failure: Hospitalization for heart failure included heart failure both during the index hospitalization and re-hospitalization for heart failure. Hospitalization was defined as treatment occurring during a hospital admission. Heart failure was judged to be present based on at least one of the following symptoms and signs: new or worsening dyspnea, orthopnea, paroxysmal nocturnal dyspnea, increasing fatigue/worsening exercise tolerance, echocardiography (EF<45%), new pulmonary oedema seen on chest X-ray in the absence of a non-cardiac cause, crepitations believed to be due to pulmonary edema, and use of loop diuretics to treat presumed pulmonary congestion[1].

Definition of stroke: Stroke was defined as a focal central neurological deficit lasting >24 hours that resulted in irreversible brain damage or body impairment. Stroke was classified as follows: infarction, haemorrhagic stroke, and transient ischaemic attack, verified by CT or MRI.

Definition of reinfarction: Reinfarction was defined as an acute MI that occurred within the 1-year follow-up period according to the universal definition of myocardial infarction[2]. Reinfarction within the first 48 hours following the index STEMI was considered procedure-related and not classified as part of the secondary end point.

Definition of coronary revascularisation: Revascularisation was defined as symptom-driven coronary revascularisation by PCI or CABG following the index PPCI, excluding staged PPCI.

Clinicaltrials.gov registration

The CONDI-2/ERIC-PPCI trial is a planned pre-specified collaboration between CONDI-2 (recruiting patients in Denmark, Spain and Serbia) and ERIC-PPCI (recruiting patients in UK) and is registered at clinicaltrials.gov NCT02342522. The CONDI-2 trial was set-up first and recruited their first patient on 6th Nov 2013, having registered their clinicaltrials.gov entry on 18th May, 2013 (NCT01857414). The ERIC-PPCI trial clinicaltrials.gov entry was set-up 21st January 2015 (NCT02342522) and the first ERIC-PPCI patient was

recruited on 15th December 2015. We then merged the 2 clinicaltrials.gov entries to the ERIC-PPCI entry NCT02342522 as advised by the clinicaltrials.gov team. This explains why the date of registration shows as 21st Jan 2015 and date first patient recruited shows 6th Nov 2013.

Troponin T Substudy

MI size was quantified as the 48 hour AUC serum level of high-sensitive Troponin-T (hsTropT). From each patient of the ERIC-PPCI component study, a single blood sample was taken at each of the 5 time-points (0, 6, 12, 24 and 48 hours following PPCI procedure). From each patient of the CONDI-2 component study, a single blood sample was taken at each of the 4 time-points (0, 6-8, 24 and 48 hours following PPCI procedure). Quantitative serum hsTropT measurement was performed using a one-step immunoassay based on electrochemiluminescence technology (Elecsys 2010, Roche, Switzerland). The reference range will be ≤14 ng/L (14 ng/L is the 99th centile of reference population with cardiovascular risk of <10%).

SUPPLEMENTARY FIGURES

Figure S1. Consort Diagram of the CONDI-2 Trial.

CONSORT Diagram, CONDI2

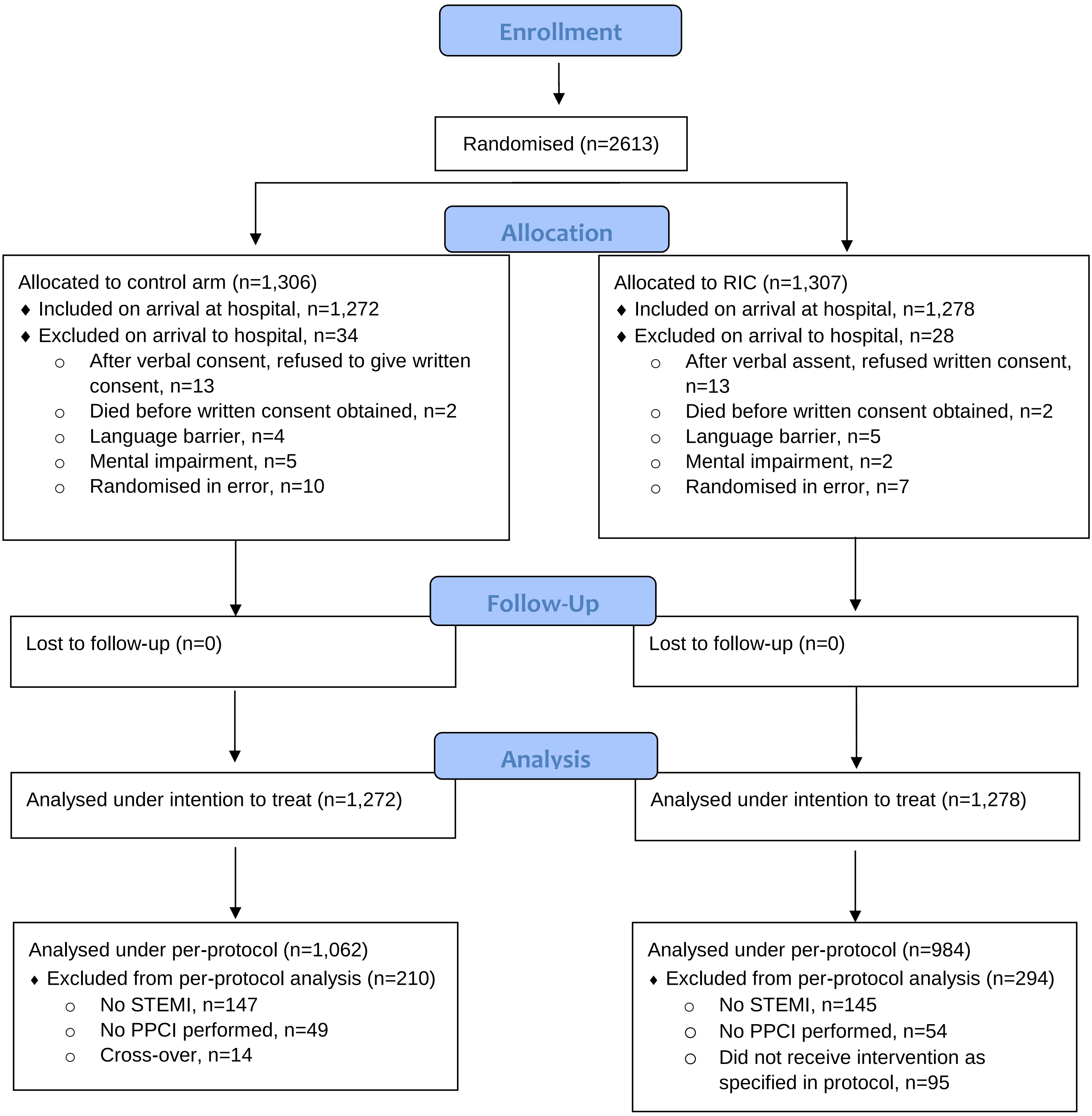

Figure S2

(a) Cumulative Incidence of Cardiac death or HHF in the CONDI-2/ERIC-PPCI Trial at 12 months (PP Analysis).

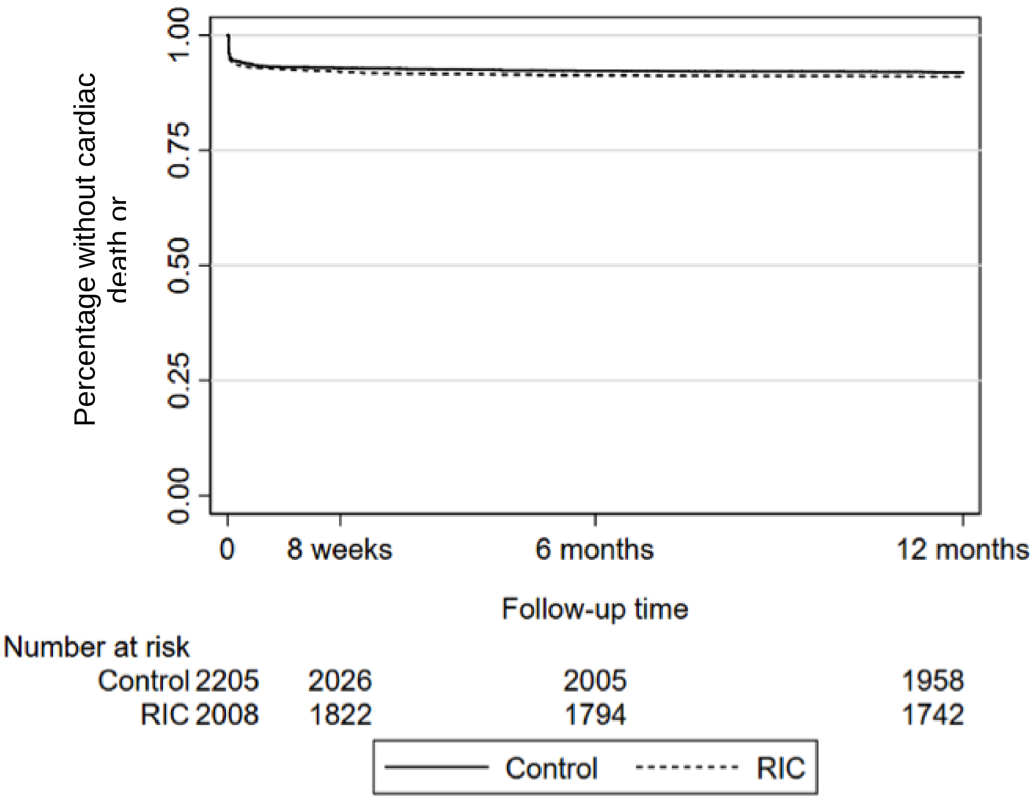

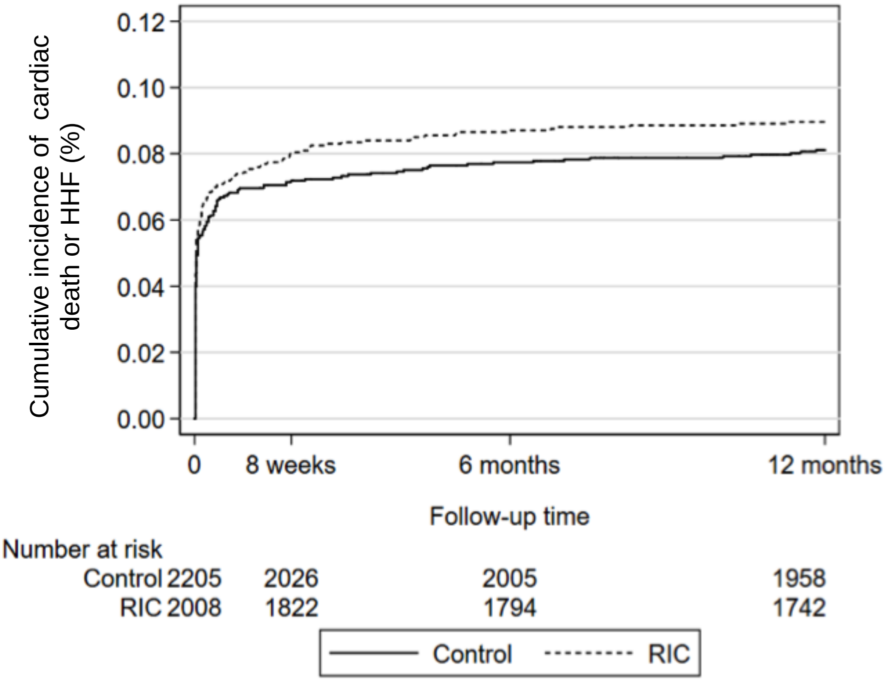

(b) Cumulative Incidence of Cardiac death or HHF in the CONDI-2 Trial at 12 months (ITT Analysis).

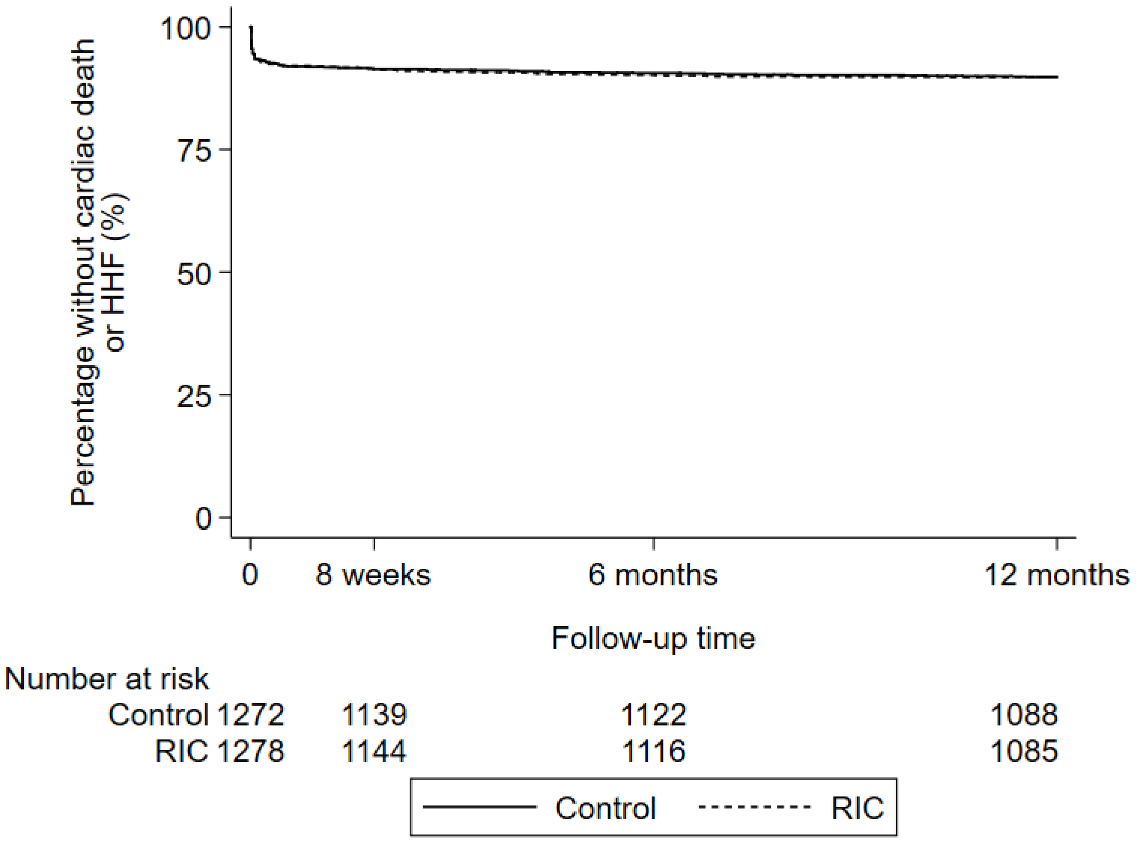

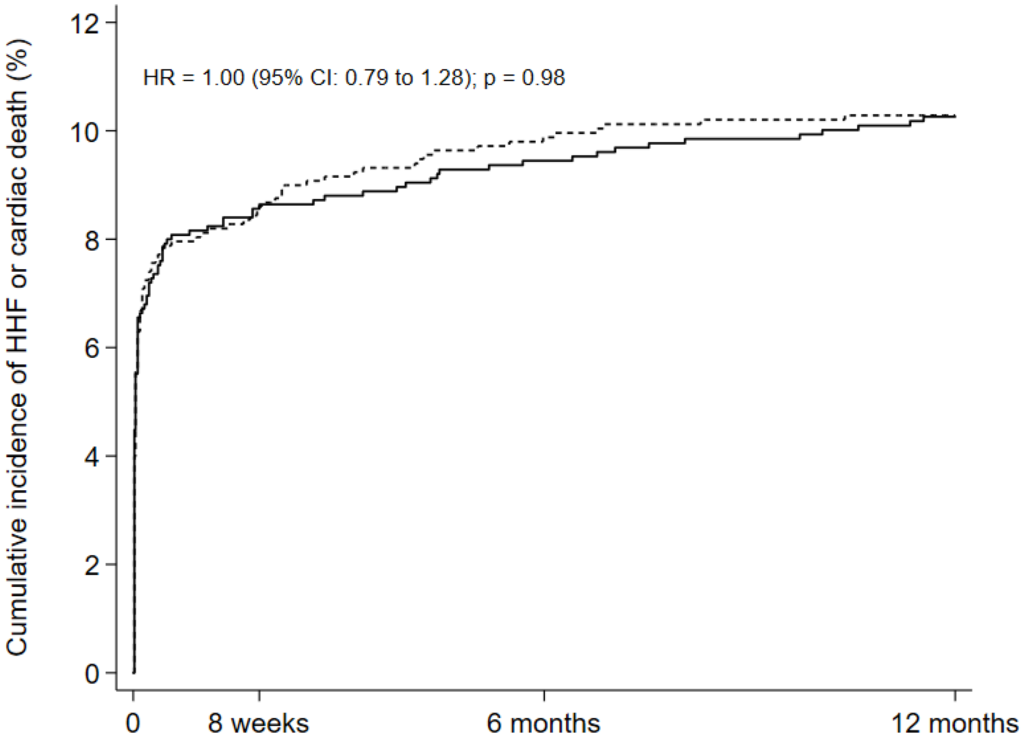

(c) Cumulative Incidence of Cardiac Death in the CONDI-2 Trial at 12 months (ITT Analysis).

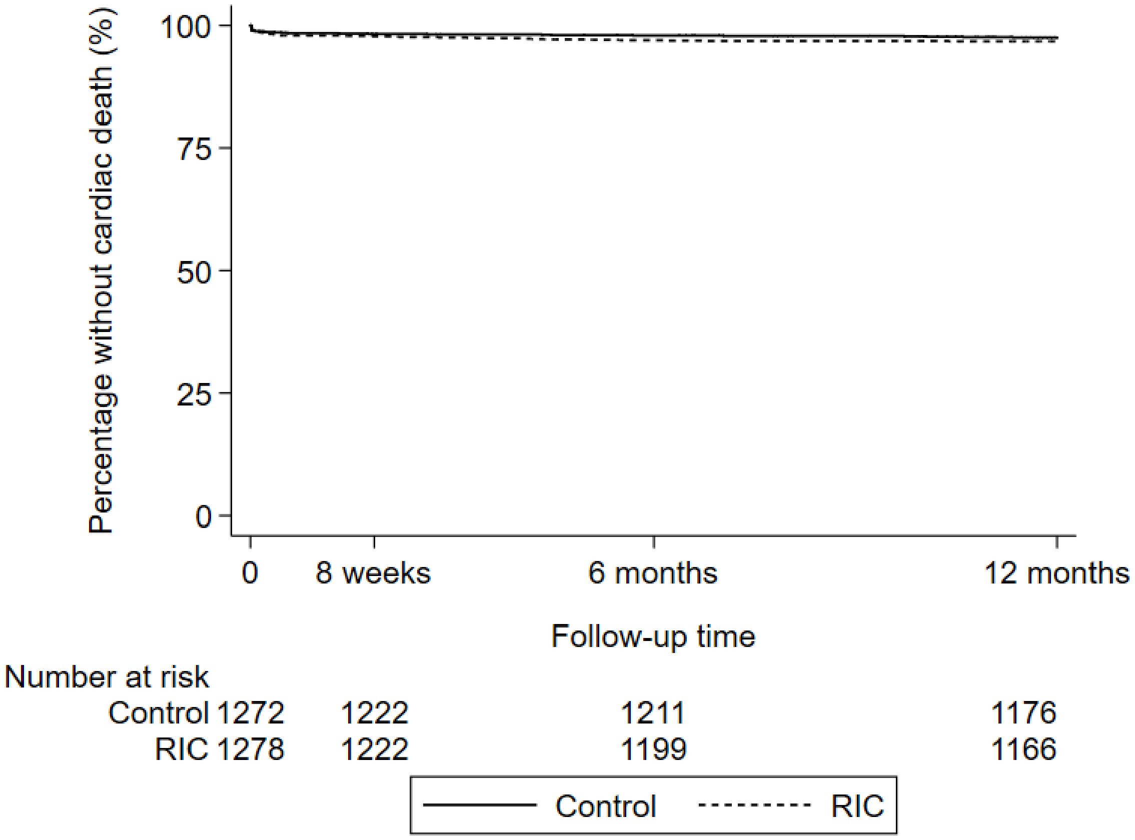

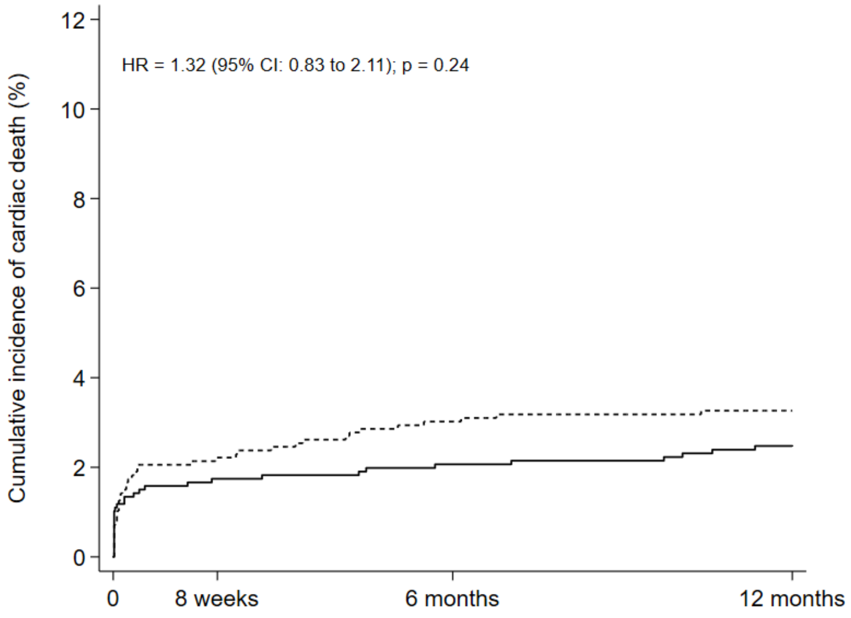

(d) Cumulative Incidence of HHF in the CONDI-2 Trial at 12 months (ITT Analysis).

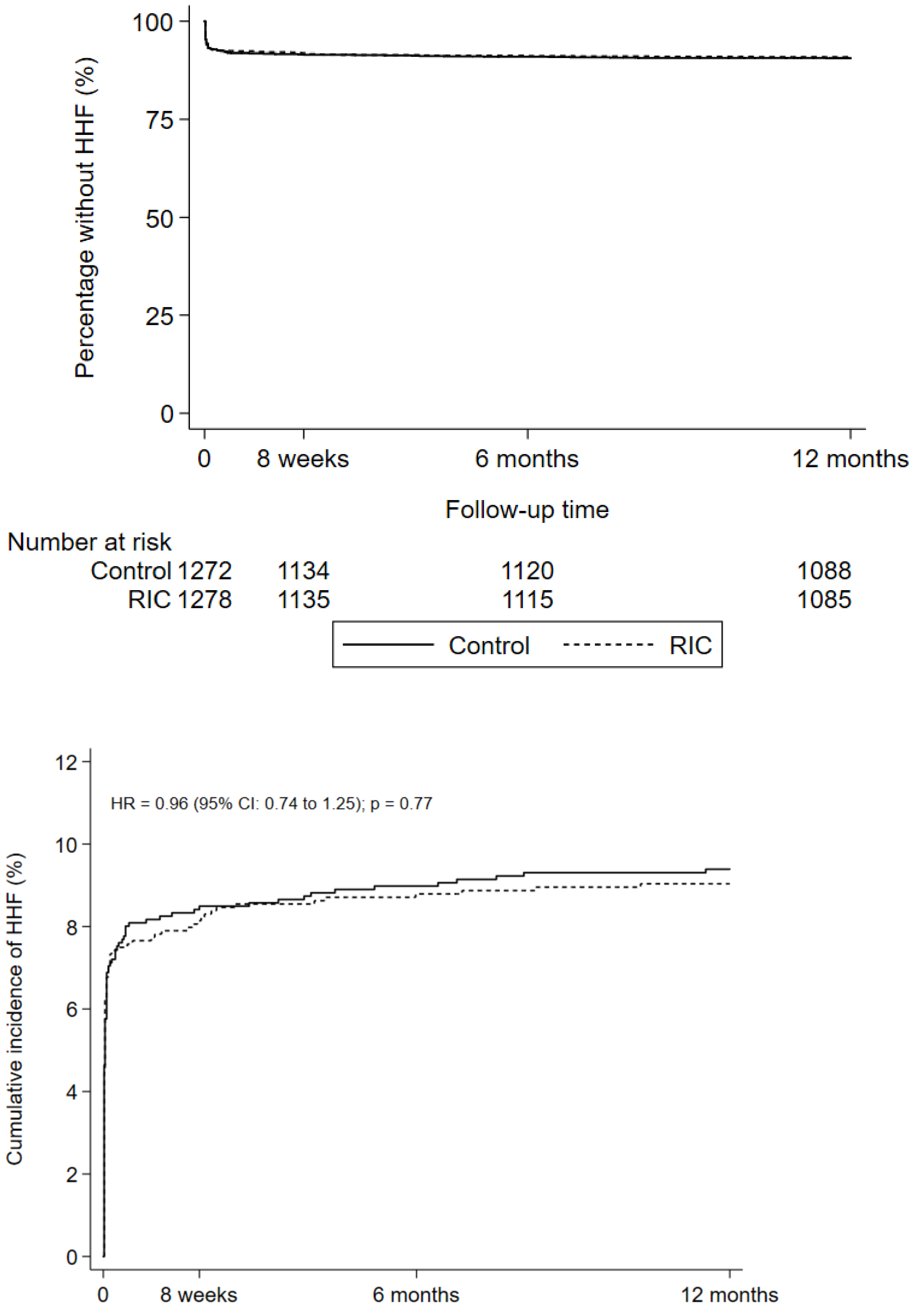

Figure S3.

(a) Cumulative Incidence of Cardiac Death or HHF in the CONDI-2 Trial at 12 Months (PP Analysis).

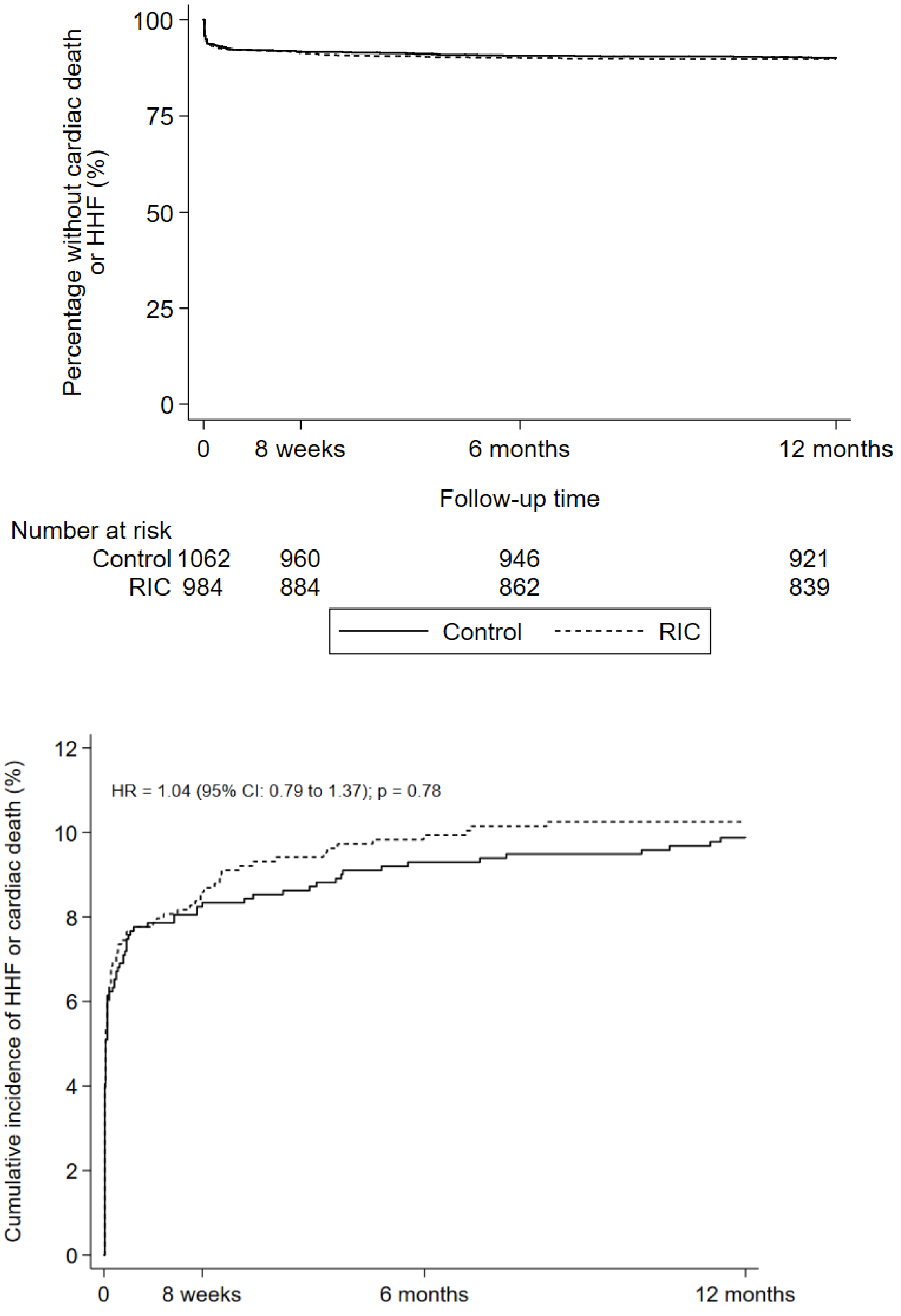

(b) **Cumulative Incidence of Cardiac Death in the CONDI-2 Trial at 12 Months (PP Analysis).**

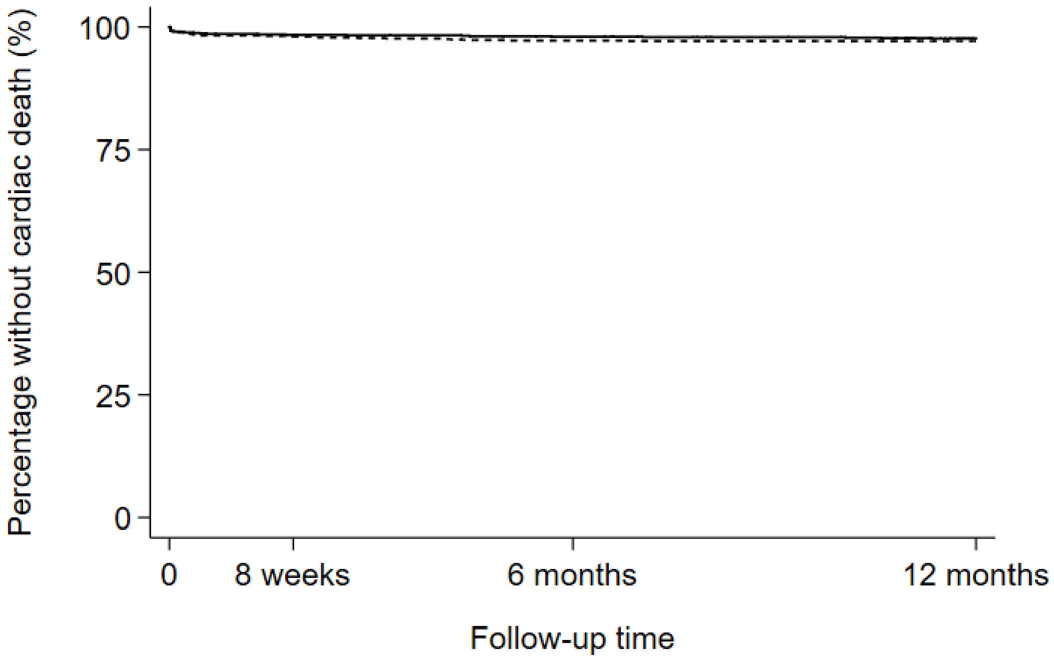

Number at risk

	0	8 weeks	6 months	12 months
Control	1062	1028	1020	992
RIC	984	947	930	905

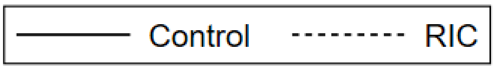

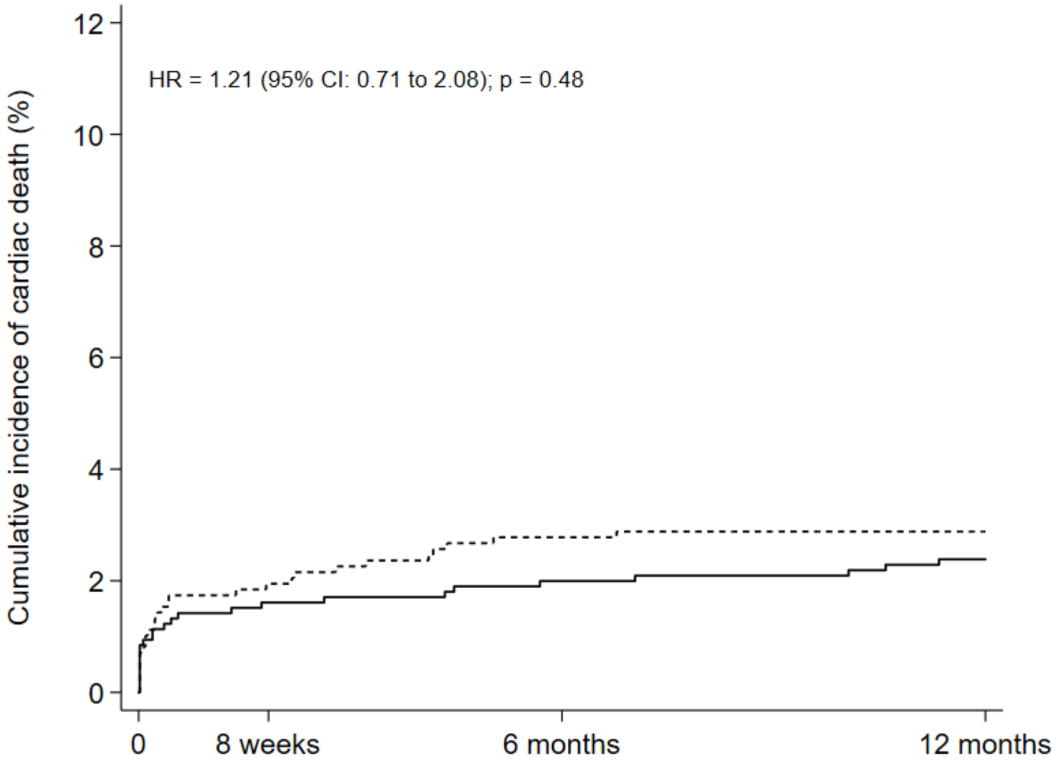

(c) Cumulative Incidence of HHF in the CONDI-2 Trial at 12 Months (PP Analysis).

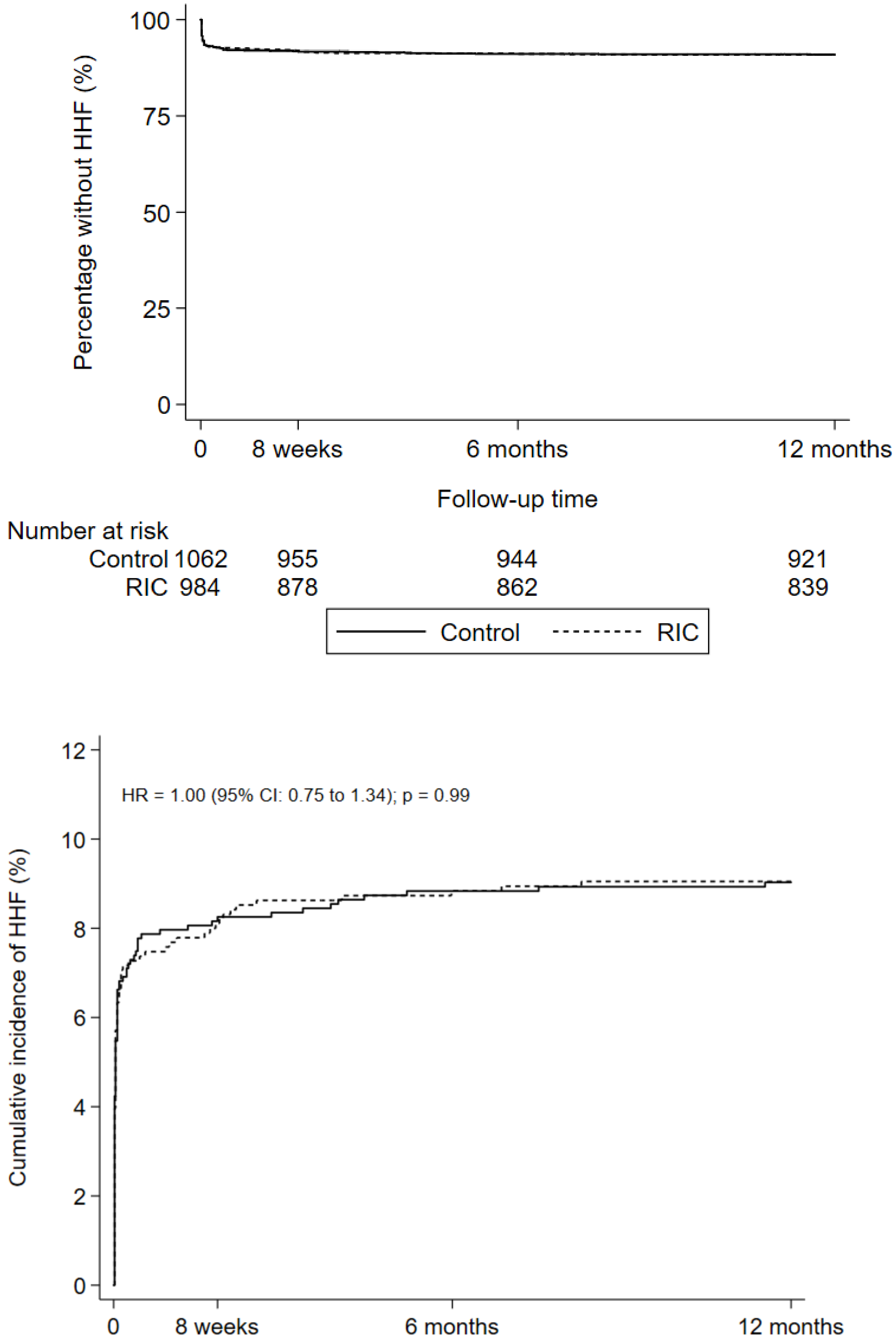

Figure S4. Consort Diagram of the ERIC-PPCI Trial.

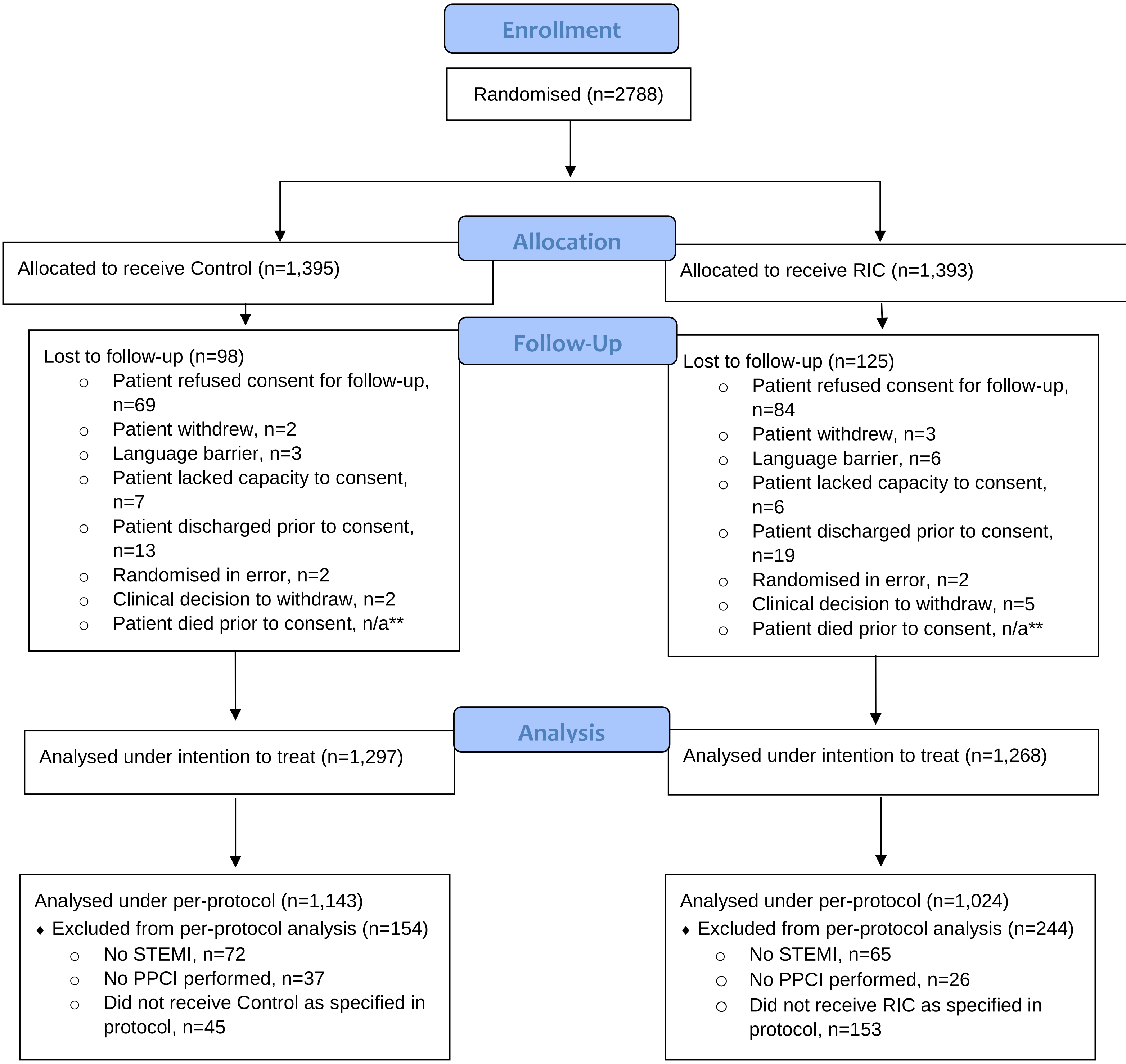

*Full screening log data were not available as not all sites were able to collect screening logs given the emergency context of patient enrolment and randomization.

**ERIC-PPCI had approval from the UK Confidentiality Group (CAG) to collect data on patients how died before consent could be obtained.

Figure S5.

(a) Cumulative Incidence of Cardiac Death or HHF in the ERIC-PPCI Trial at 12 Months (ITT Analysis).

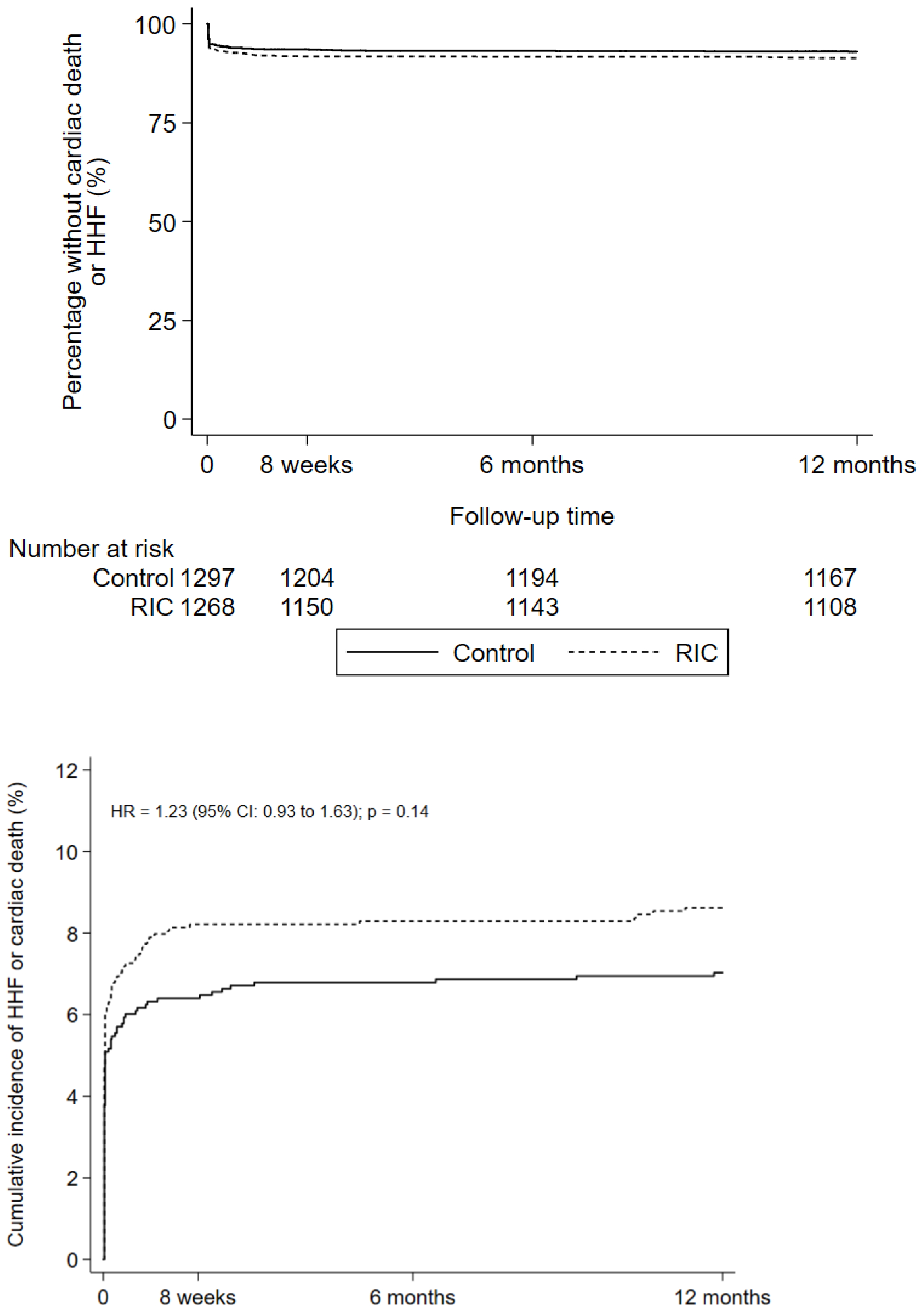

(b) Cumulative Incidence of Cardiac Death in the ERIC-PPCI Trial at 12 Months (ITT Analysis).

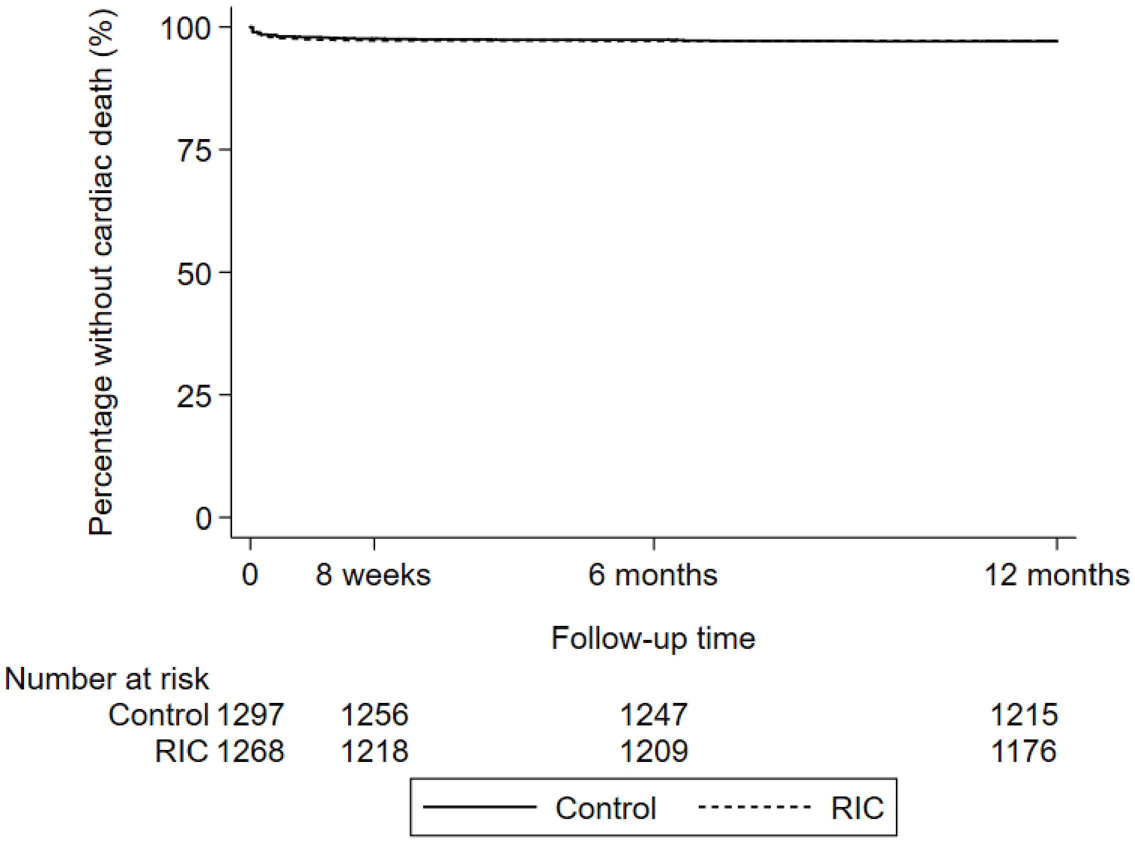

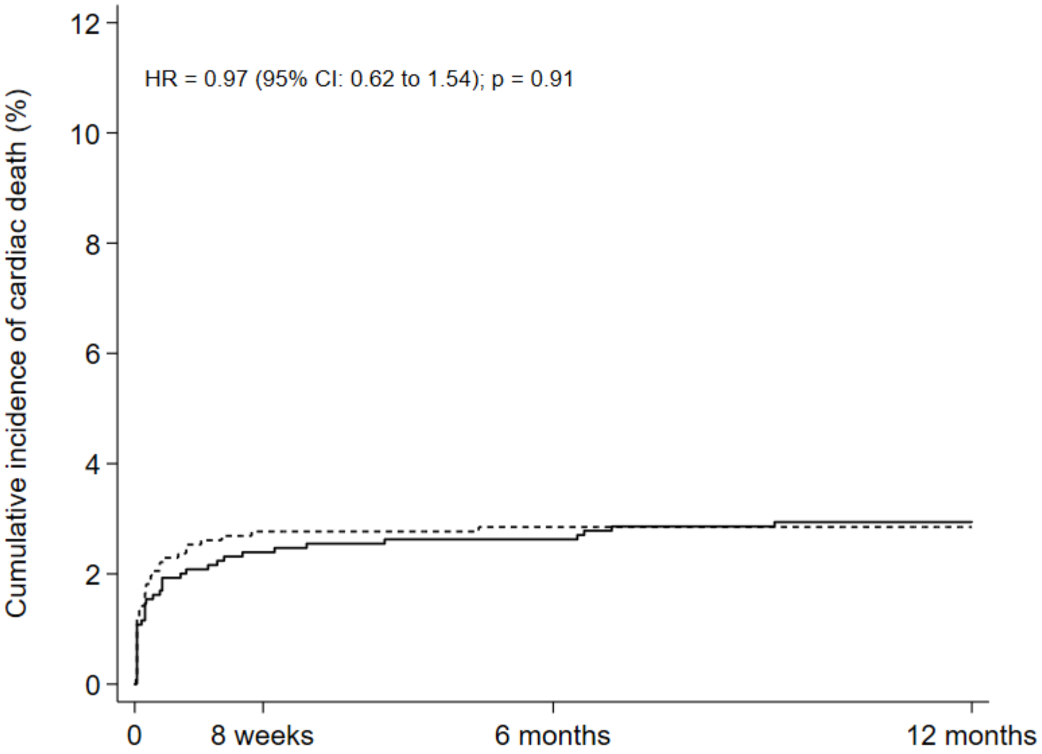

(c) Cumulative Incidence of HHF in the ERIC-PPCI Trial at 12 months (ITT Analysis).

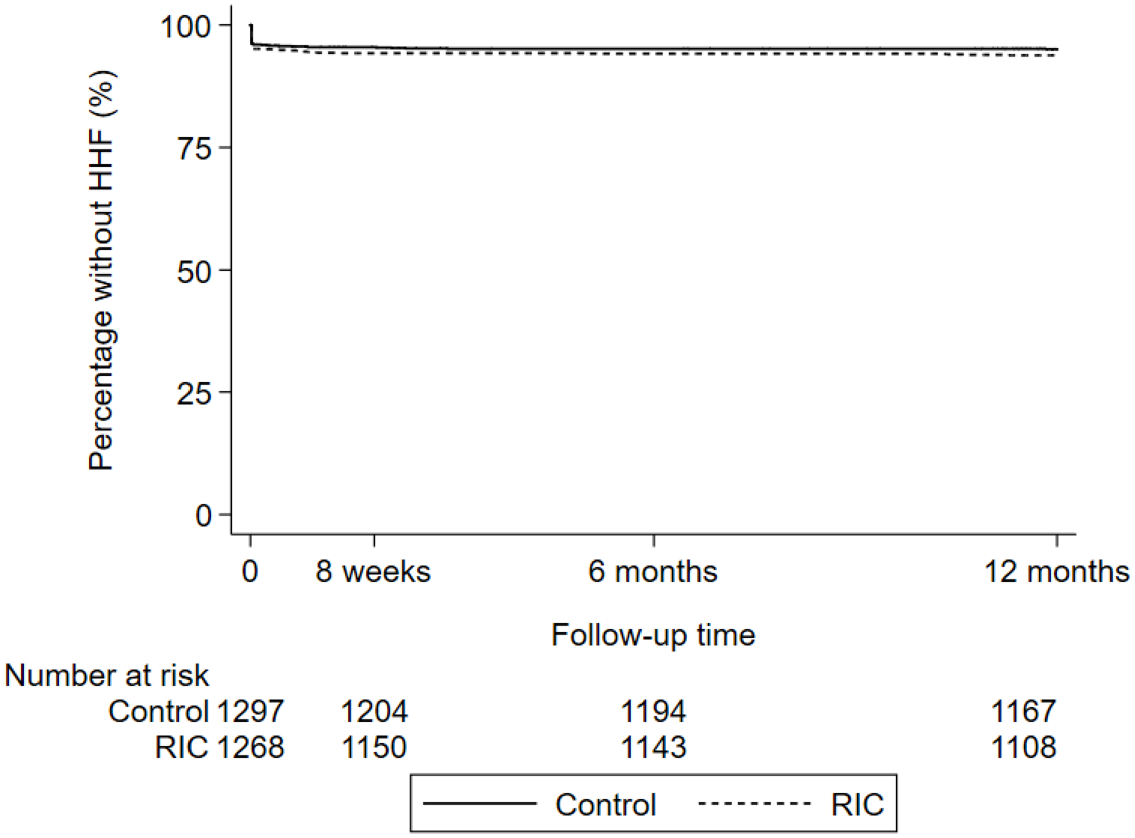

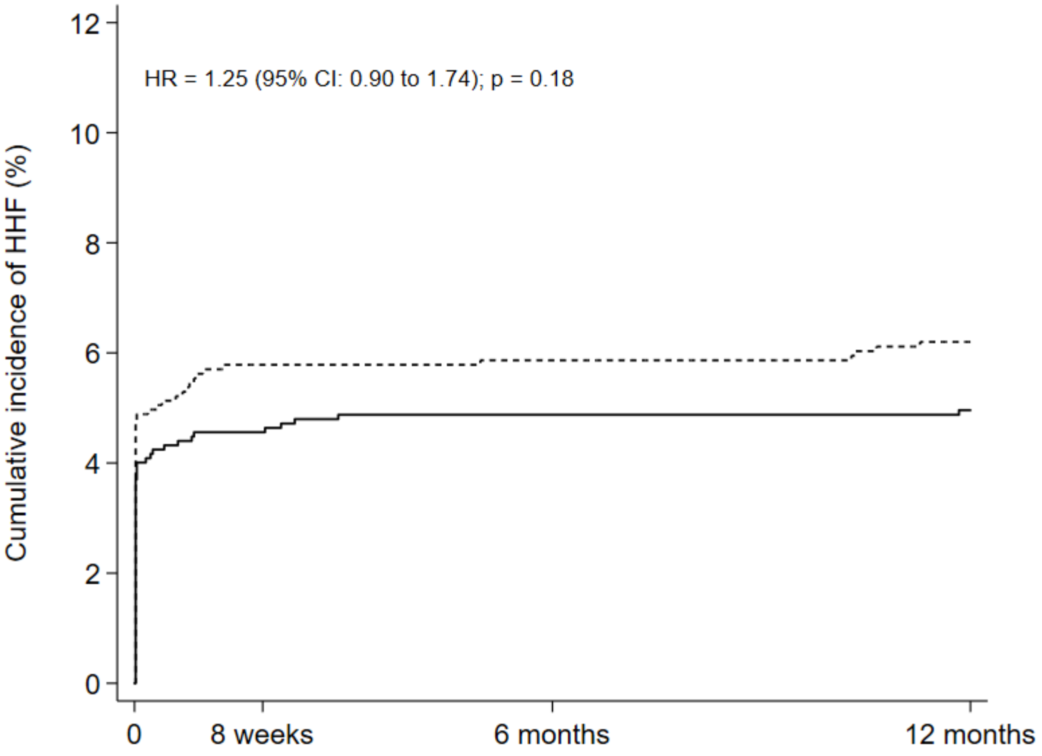

Figure S6.

(a) Cumulative Incidence of Cardiac Death or HHF in the ERIC-PPCI Trial at 12 Months (PP Analysis).

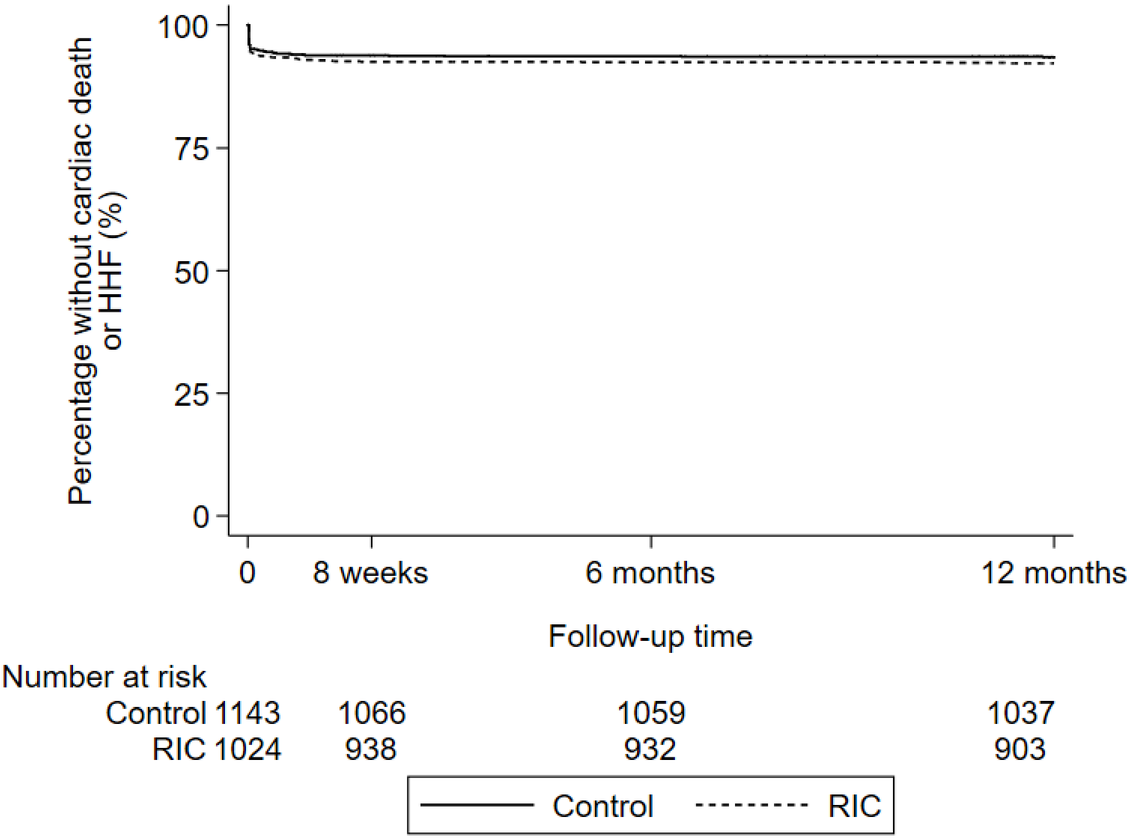

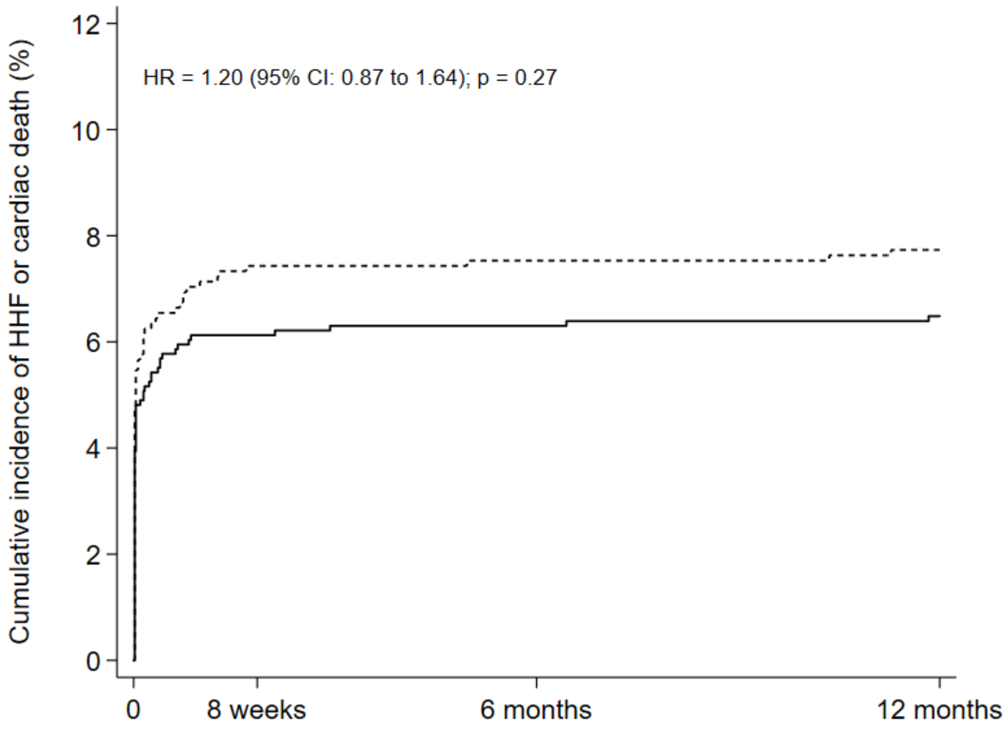

(b) Cumulative Incidence of Cardiac Death in the ERIC-PPCI Trial at 12 Months (PP Analysis).

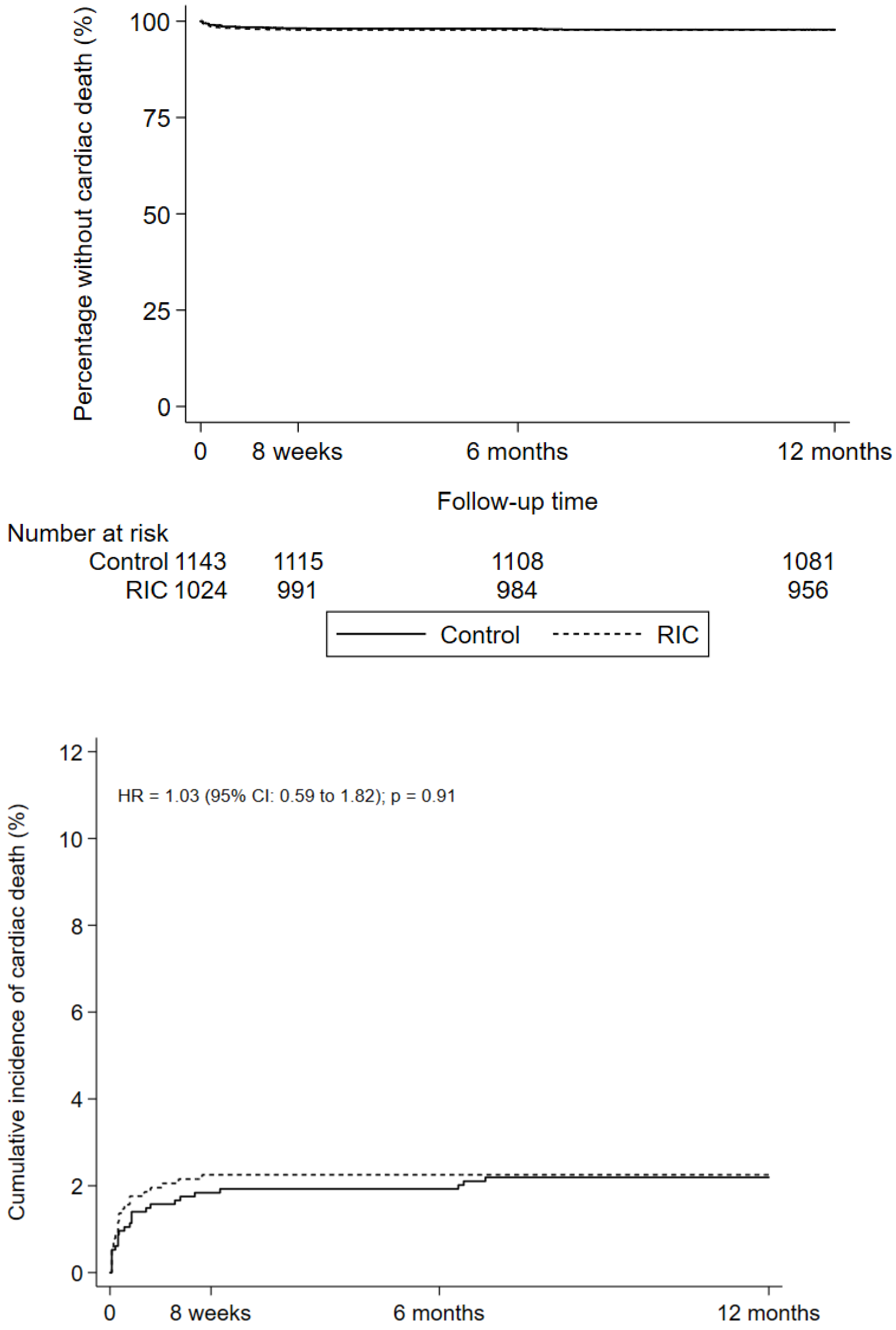

(c) Cumulative Incidence of HHF in the ERIC-PPCI Trial at 12 Months (PP Analysis).

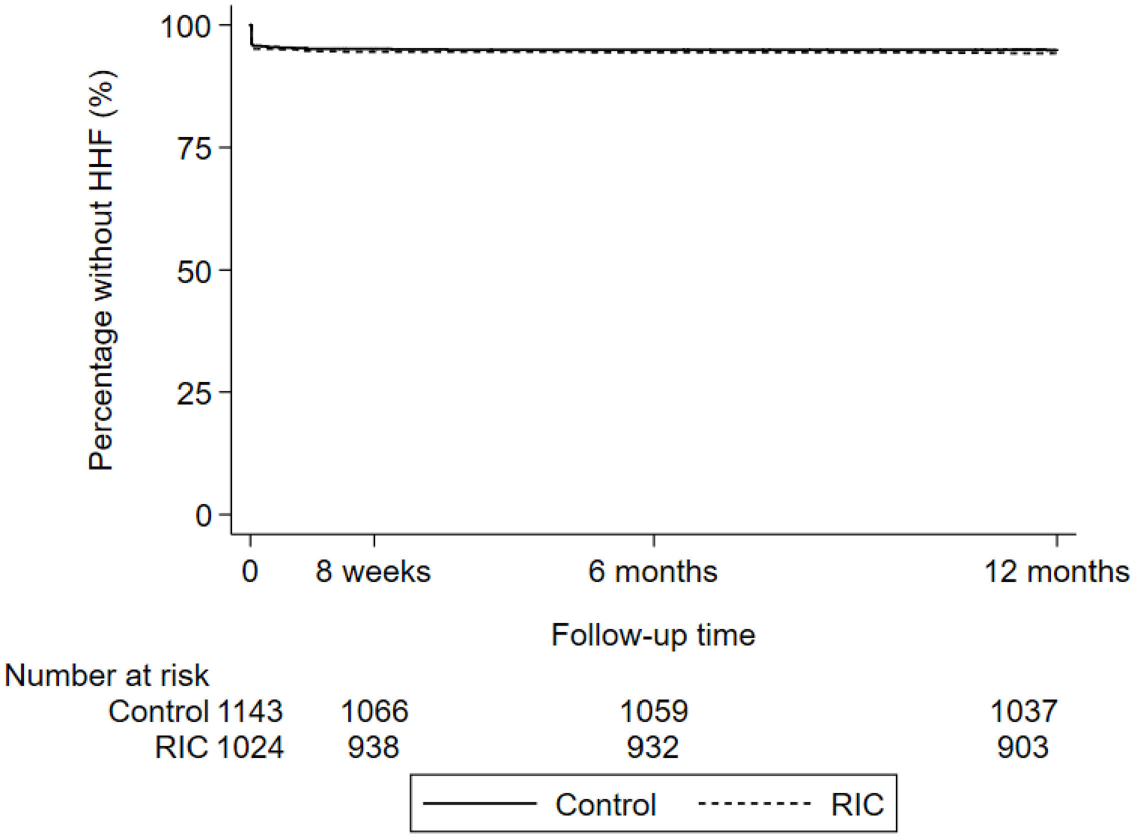

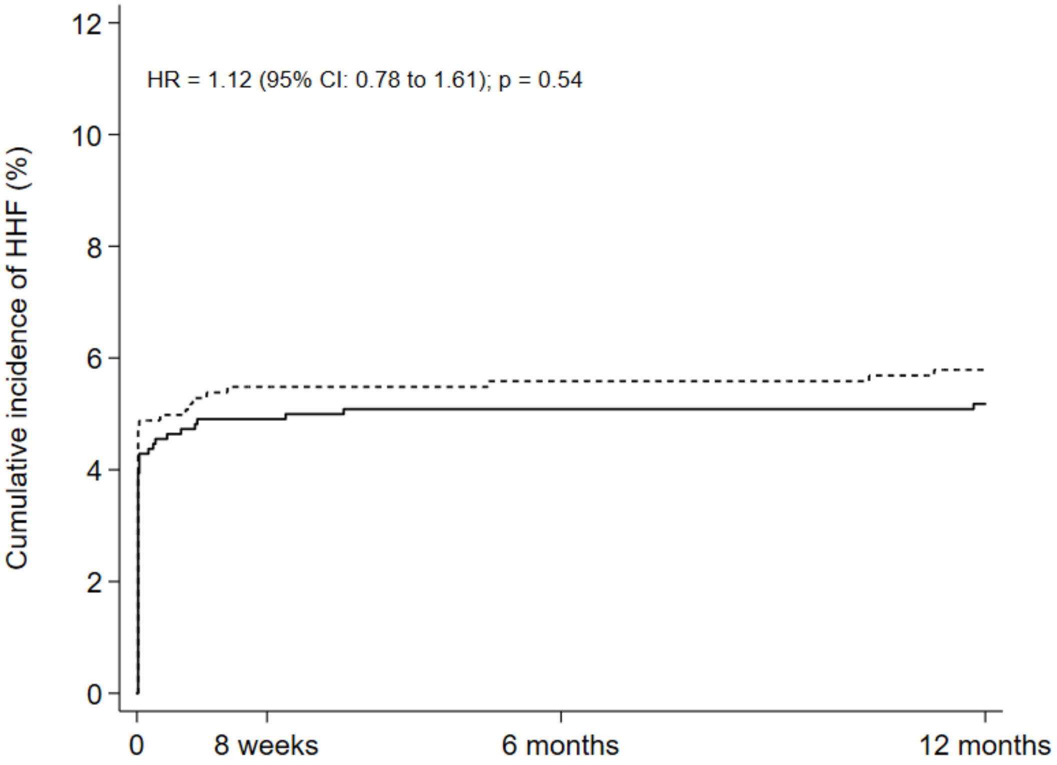

Table S1a. Baseline Characteristics of Patients and PPCI Procedure Details in the CONDI-2/ERIC-PPCI Trial (ITT Analysis).

	Control (n=2569)		RIC (n=2546)	
**Demographics**				
Age (years), mean (SD)	63.1	(12.2)	63.9	(12.1)
Missing, n	20		22	
Female, n (%)	576	(22.4)	611	(24.0)
Missing, n	0		0	
Body mass index (BMI; kg/m2), mean (SD)	27.5	(4.7)	27.5	(4.9)
Missing, n	129		120	
Current smokers, n (%)	1015	(40.9)	993	(40.5)
Missing, n	86		97	
**Comorbidity, n (%)**				
Hypertension	1020	(40.1)	1100	(43.7)
Missing, n	25		28	
Previous acute myocardial infarction (MI)	253	(9.9)	265	(10.5)
Missing, n	16		16	
Hypercholesterolemia	682	(27.2)	696	(28.0)
Missing, n	66		56	
Medically treated diabetes	265	(10.4)	302	(11.9)
Missing, n	12		12	
Family history of ischaemic heart disease (IHD)	819	(34.3)	853	(36.5)
Not known, n	182		209	
**Medications at admission for PPCI, n (%)**				
Sulphonylurea	62	(2.5)	61	(2.4)
Missing, n	50		50	
Metformin	196	(7.8)	221	(8.8)
Missing, n	51		47	
Insulin	74	(2.9)	81	(3.2)
Missing, n	21		13	
Clopidogrel	85	(3.3)	98	(3.9)
Missing, n	21		18	
Ticagrelor	52	(2.0)	62	(2.5)
Missing, n	20		19	
Prasugrel	4	(0.2)	3	(0.1)
Missing, n	23		18	
Statins	652	(25.6)	665	(26.3)
Missing, n	22		20	
Aspirin	456	(17.9)	665	(19.2)
Missing, n	21		18	
Beta-blockers	384	(15.1)	407	(16.1)

Missing, n	30		21	
ACE inhibitors/Angiotensin receptor blockers	456	(17.9)	466	(18.5)
Missing, n	28		21	
**BP and Killip class at randomisation**				
Systolic blood pressure (mmHg), mean (SD)	131	(24)	131	(24)
Missing, n	79		78	
Diastolic blood pressure (mmHg), mean (SD)	77	(15)	76	(15)
Missing, n	83		80	
Killip class, n (%)				
Class I	2462	(95.9)	2435	(95.6)
Class II	75	(2.9)	75	(2.9)
Class III	14	(0.5)	11	(0.4)
Class IV (including cardiogenic shock)	17	(0.7)	25	(1.0)
Missing, n	1		0	
**RIC procedure**				
RIC procedure started, n (%)	-		2496	(98.0)
Missing, n	-		0	
Number of complete RIC cycles administered, n (%)				
1	-		37	(1.6)
2	-		40	(1.8)
3	-		142	(6.3)
4	-		2027	(90.2)
Missing, n	-		250	
**PPCI procedure**				
PPCI completed, n (%)	2258	(87.9)	2227	(87.5)
TIMI flow grade at admission, n (%)				
TIMI 0	1513	(67.9)	1478	(67.1)
TIMI 1	154	(6.9)	145	(6.6)
TIMI 2	218	(9.8)	225	(10.2)
TIMI 3	342	(15.4)	355	(16.1)
Missing, n	31		24	
Symptom to balloon time (min), median (IQR)	177	(128 to 279)	178	(130 to 278)
Missing, n	339		361	
First medical contact to balloon time (min), median (IQR)	102	(82 to 126)	103	(83 to 128)
Missing, n	356		385	
Infarct-related coronary artery, n (%)				
Left anterior descending	974	(43.1)	911	(40.9)
Circumflex	297	(13.2)	298	(13.4)
Right coronary	985	(43.6)	1014	(45.6)
Other	2	(0.1)	3	(0.1)
Missing, n	0		1	
Vessels with clinically significant disease, n (%)				
0	1	(0.0)	2	(0.1)

1	1314	(58.2)	1274	(57.2)
2	659	(29.2)	641	(28.8)
3	284	(12.6)	310	(13.9)
Missing, n	0		0	
Stenting of culprit lesion by PPCI, n (%)	2104	(93.2)	2080	(93.4)
Missing, n	0		1	
Supplementary staged PCI, n (%)	291	(12.9)	261	(11.7)
Supplementary staged CABG, n (%)	52	(2.3)	62	(2.8)
TIMI flow grade after procedure, n (%)				
TIMI 0	27	(1.2)	26	(1.2)
TIMI 1	20	(0.9)	9	(0.4)
TIMI 2	112	(5.0)	86	(3.9)
TIMI 3	2064	(92.8)	2079	(94.5)
Missing, n	35		27	
**Medications administered in relation to PPCI, n (%)**				
Opioids*	549	(52.1)	522	(50.2)
Missing, n	1204		1187	
Heparin	1904	(84.4)	1893	(85.1)
Missing, n	3		3	
Aspirin	2147	(95.2)	2129	(95.9)
Missing, n	3		6	
Clopidogrel	610	(27.0)	573	(25.7)
Missing, n	2		1	
Ticagrelor	1551	(68.7)	1554	(69.8)
Missing, n	1		0	
Prasugrel	103	(4.6)	97	(4.4)
Missing, n	2		1	
Nitrates	1758	(79.1)	1693	(77.5)
Missing, n	36		42	
Glycoprotein IIb/IIIa inhibitor	450	(20.0)	404	(18.2)
Missing, n	8		4	
Bivalirudin	505	(22.4)	489	(22.0)
Missing, n	8		3	
Cangrelor*	134	(12.3)	127	(11.7)
Missing, n	1170		1142	

Abbreviations: RIC = remote ischemic conditioning; SD = standard deviation; IQR = interquartile range; BMI = body mass index; MI = myocardial infarction; IHD = ischemic heart disease; PPCI = primary percutaneous coronary intervention; BP = blood pressure; CABG = coronary artery bypass graft surgery
*Opioids and cangrelor data collected only in CONDI-2.

Table S2. Subgroup Analyses for the CONDI-2/ERIC-PPCI Trial (ITT Analysis).

	**Control** (n=2569)	**RIC** (n=2546)	**Treatment effect, RIC vs. Control (95% CI)**	**Interaction p-value**
Age (years), n (%)				
<55	24 (3.5)	22 (3.5)	1.00 (0.56 to 1.78)	
55-<65	40 (5.3)	48 (7.0)	1.33 (0.87 to 2.02)	
65-<75	58 (9.4)	65 (9.0)	0.97 (0.68 to 1.38)	
≥75	79 (16.9)	85 (17.9)	1.05 (0.77 to 1.43)	0.70
Medically treated diabetes, n (%)				
No	179 (7.9)	193 (8.7)	1.11 (0.91 to 1.36)	
Yes	38 (14.6)	43 (14.5)	0.98 (0.63 to 1.51)	0.60
TIMI flow grade at admission, n (%)				
TIMI 0-1	156 (9.2)	169 (10.3)	1.11 (0.89 to 1.38)	
TIMI 2-3	36 (5.7)	42 (6.5)	1.14 (0.73 to 1.78)	0.92
Infarct location, n (%)				
Left anterior descending	132 (13.3)	147 (15.7)	1.21 (0.96 to 1.53)	
Other	65 (5.0)	66 (4.9)	0.98 (0.70 to 1.39)	0.33
First medical contact to balloon time (min), n (%)				
<90	46 (6.2)	56 (7.7)	1.24 (0.84 to 1.84)	
90-<120	63 (7.9)	60 (8.2)	1.01 (0.71 to 1.44)	
120-≤720	73 (10.9)	75 (10.8)	1.01 (0.73 to 1.39)	0.67
**Exploratory analyses**				
Timing of intervention, n (%)				
In ambulance (n=2,290)	113 (10.0)	112 (9.8)	0.98 (0.76 to 1.28)	
On arrival at hospital (n=2,825)	107 (7.5)	127 (9.1)	1.22 (0.95 to 1.58)	0.24
Timing of intervention in relation to reperfusion, n (%)				
≤4 cycles of RIC prior to reperfusion (n=1,030)	220 (8.6)	82 (8.0)	1.07 (0.81 to 1.41)	0.64
4 cycles of RIC prior to reperfusion (n=1,079)	220 (8.6)	106 (9.9)	1.03 (0.80 to 1.31)	0.84
Gender, n (%)				
Male	152 (7.7)	172 (8.9)	1.17 (0.94 to 1.46)	
Female	68 (11.9)	67 (11.1)	0.92 (0.66 to 1.29)	0.24
Smoking status, n (%)				
Current smoker	64 (6.3)	78 (7.9)	1.26 (0.90 to 1.75)	
Ex-smoker	70 (9.4)	65 (8.7)	0.92 (0.65 to 1.28)	
Never smoked	73 (10.2)	80 (11.4)	1.12 (0.81 to 1.54)	0.42
BMI (kg/m^2), n (%)				
<25	64 (8.2)	74 (9.7)	1.18 (0.85 to 1.65)	
≥25	141 (8.6)	148 (9.0)	1.05 (0.83 to 1.32)	0.56
Symptom to balloon time (min), n (%)				
<150	46 (5.4)	62 (7.9)	1.47 (1.01 to 2.16)	
150-<240	61 (9.4)	59 (8.3)	0.89 (0.62 to 1.27)	
240-≤720	66 (10.0)	65 (10.1)	1.01 (0.72 to 1.42)	0.15
Hypertension, n (%)				
No	110 (7.2)	108 (7.7)	1.06 (0.81 to 1.38)	
Yes	105 (10.4)	125 (11.5)	1.10 (0.85 to 1.43)	0.82
Beta-blockers, n (%)				
No	169 (7.9)	190 (9.0)	1.15 (0.93 to 1.41)	
Yes	40 (10.5)	43 (10.7)	1.00 (0.65 to 1.54)	0.58
ACE-inhibitors, n (%)				
No	164 (7.9)	181 (8.9)	1.12 (0.91 to 1.38)	
Yes	45 (10.0)	52 (11.2)	1.13 (0.76 to 1.69)	0.95

	Control (n=2569)	RIC (n=2546)	Treatment effect, RIC vs. Control (95% CI)	Interaction p-value
Angiotensin II receptor blockers, n (%)				
No	182 (8.0)	201 (9.1)	1.15 (0.94 to 1.40)	
Yes	28 (11.3)	32 (10.6)	0.90 (0.54 to 1.49)	0.37
Statins, n (%)				
No	148 (7.8)	169 (9.1)	1.17 (0.94 to 1.46)	
Yes	64 (9.9)	64 (9.7)	0.97 (0.69 to 1.38)	0.39
Metformin, n (%)				
No	184 (8.0)	201 (8.9)	1.12 (0.91 to 1.36)	
Yes	20 (10.3)	24 (11.0)	1.06 (0.58 to 1.91)	0.86
Sulphonylurea, n (%)				
No	199 (8.1)	218 (9.0)	1.11 (0.91 to 1.34)	
Yes	5 (8.1)	7 (11.8)	1.39 (0.44 to 4.38)	0.70
Insulin, n (%)				
No	198 (8.1)	213 (8.7)	1.09 (0.90 to 1.32)	
Yes	15 (20.5)	21 (26.2)	1.30 (0.67 to 2.52)	0.61
Ticagrelor, n (%)				
No	84 (10.4)	91 (11.7)	1.12 (0.84 to 1.51)	
Yes	124 (7.6)	131 (8.0)	1.06 (00.83 to 1.35)	0.75

The Table presents Kaplan-Meier estimates of the n (%) experiencing hospitalization for heart failure or cardiovascular death within 12 months.
Abbreviations: RIC = remote ischemic conditioning; CI = confidence interval

Table S3. Baseline Characteristics of Patients and PPCI Procedure Details in the CONDI-2 Trial (ITT analysis).

	Control (n=1272)		RIC (n=1278)	
**Demographics**				
Age (years), mean (SD)	62.9	(12.1)	63.6	(12.1)
Missing, n	0		0	
Female, n (%)	294	(23.1)	322	(25.2)
Missing, n	0		0	
Body mass index (BMI; kg/m2), mean (SD)	27.3	(4.5)	27.4	(4.8)
Missing, n	56		46	
Current smokers, n (%)	571	(45.7)	549	(44.1)
Missing, n	22		32	
**Comorbidity, n (%)**				
Hypertension	501	(39.4)	552	(43.2)
Missing, n	2		1	
Previous acute myocardial infarction (MI)	141	(11.1)	132	(10.3)
Missing, n	2		2	
Hypercholesterolemia	314	(24.7)	317	(24.8)
Missing, n	3		2	
Medically treated diabetes	124	(9.8)	156	(12.2)
Missing, n	1		1	
Family history of ischaemic heart disease (IHD)	389	(31.9)	407	(33.2)
Not known, n	53		51	
**Medications at admission for PPCI, n (%)**				
Sulphonylurea	18	(1.5)	20	(1.6)
Missing, n	34		38	
Metformin	83	(6.7)	106	(8.5)
Missing, n	34		37	
Insulin	33	(2.6)	43	(3.4)
Missing, n	3		2	
Clopidogrel	40	(3.2)	37	(2.9)
Missing, n	3		4	
Ticagrelor	5	(0.4)	18	(1.4)
Missing, n	3		4	
Prasugrel	1	(0.1)	0	(0.0)
Missing, n	3		4	
Statins	300	(23.7)	310	(24.4)
Missing, n	4		5	
Aspirin	222	(17.5)	225	(17.7)
Missing, n	4		4	
Beta-blockers	187	(14.8)	207	(16.2)

Missing, n	9		4	
ACE inhibitors/Angiotensin receptor blockers	204	(16.2)	195	(15.3)
Missing, n	9		4	
**BP and Killip class at randomisation**				
Systolic blood pressure (mmHg), mean (SD)	129	(24)	130	(25)
Missing, n	11		44	
Diastolic blood pressure (mmHg), mean (SD)	74	(14)	74	(15)
Missing, n	13		44	
Killip class, n (%)				
Class I	1193	(93.9)	1198	(93.7)
Class II	54	(4.2)	56	(4.4)
Class III	13	(1.0)	8	(0.6)
Class IV (including cardiogenic shock)	11	(0.9)	16	(1.3)
Missing, n	1		0	
**RIC procedure**				
RIC procedure started, n (%)	-		1245	(97.4)
Number of complete RIC cycles administered, n (%)				
1	-		4	(0.4)
2	-		12	(1.2)
3	-		62	(6.1)
4	-		945	(92.4)
Missing, n	-		222	
**PPCI procedure**				
PPCI completed, n (%)	1090	(85.7)	1085	(84.9)
TIMI flow grade at admission, n (%)				
TIMI 0	638	(58.9)	638	(59.0)
TIMI 1	78	(7.2)	58	(5.4)
TIMI 2	135	(12.5)	136	(12.6)
TIMI 3	233	(21.5)	249	(23.0)
Missing, n	6		4	
Symptom to balloon time (min), median (IQR)	173	(123 to 280)	172	(123 to 276)
Missing, n	185		200	
First medical contact to balloon time (min), median (IQR)	94	(76 to 119)	95	(76 to 118)
Missing, n	184		201	
Infarct-related coronary artery, n (%)				
Left anterior descending	481	(44.1)	434	(40.0)
Circumflex	153	(14.0)	152	(14.0)
Right coronary	456	(41.8)	499	(46.0)
Missing, n	0		0	
Vessels with clinically significant disease, n (%)				
0	0	(0.0)	0	(0.0)
1	654	(60.0)	651	(60.0)
2	305	(28.0)	278	(25.6)

3	131	(12.0)	156	(14.4)
Missing, n	0		0	
Stenting of culprit lesion by PPCI, n (%)	990	(90.8)	984	(90.7)
Missing, n	0		0	
Supplementary staged PCI, n (%)	189	(17.3)	164	(15.1)
Supplementary staged CABG, n (%)	36	(3.3)	47	(4.3)
TIMI flow grade after procedure, n (%)				
TIMI 0	13	(1.2)	11	(1.0)
TIMI 1	13	(1.2)	2	(0.2)
TIMI 2	49	(4.5)	33	(3.1)
TIMI 3	1010	(93.1)	1035	(95.7)
Missing, n	5		4	
**Medications administered in relation to PPCI, n (%)**				
Opioids	549	(52.1)	522	(50.2)
Missing, n	36		45	
Heparin	824	(75.7)	822	(76.0)
Missing, n	2		3	
Aspirin	1072	(98.4)	1073	(99.2)
Missing, n	1		3	
Clopidogrel	230	(21.1)	225	(20.7)
Missing, n	0		0	
Ticagrelor	760	(69.7)	761	(70.1)
Missing, n	0		0	
Prasugrel	1	(0.1)	1	(0.1)
Missing, n	0		0	
Nitrates	789	(74.8)	744	(71.3)
Missing, n	35		42	
Glycoprotein IIb/IIIa inhibitor	143	(13.2)	130	(12.0)
Missing, n	7		4	
Bivalirudin	442	(40.8)	426	(39.4)
Missing, n	7		3	
Cangrelor	134	(12.3)	127	(11.7)
Missing, n	2		0	

Abbreviations: RIC = remote ischemic conditioning; SD = standard deviation; IQR = interquartile range; BMI = body mass index; MI = myocardial infarction; IHD = ischemic heart disease; PPCI = primary percutaneous coronary intervention; BP = blood pressure; CABG = coronary artery bypass graft surgery
*Opioids and cangrelor data collected only in CONDI-2.

Table S4. Primary and Secondary Outcomes in the CONDI-2 Trial (ITT Analysis).

	**Control** (n=1272)	**RIC** (n=1278)	**Treatment effect, RIC vs. Control (95% CI)**	**p-value**
**Primary outcome, n (%)**				
Combined HHF and Cardiac death within 12 months	129 (10.3)	130 (10.3)	1.00 (0.79 to 1.28)	0.98
Cardiac death within 12 months	31 (2.5)	41 (3.3)	1.32 (0.83 to 2.11)	0.24
HHF within 12 months	118 (9.4)	114 (9.0)	0.96 (0.74 to 1.25)	0.77
**Secondary outcomes, n (%)**				
MACCE within 12 months	109 (8.7)	111 (8.8)	1.02 (0.78 to 1.32)	0.90
All-cause mortality within 12 months	48 (3.8)	62 (4.9)	1.29 (0.89 to 1.88)	0.18
Reinfarction within 12 months	27 (2.2)	22 (1.8)	0.81 (0.46 to 1.43)	0.47
Unplanned revascularisation within 12 months	29 (2.4)	26 (2.1)	0.90 (0.53 to 1.52)	0.69
Stroke within 12 months	15 (1.2)	14 (1.1)	0.94 (0.45 to 1.94)	0.86
Combined HHF and cardiac death within 30 days	103 (8.2)	103 (8.1)	0.99 (0.76 to 1.31)	0.96
Cardiac death within 30 days	20 (1.6)	26 (2.1)	1.29 (0.72 to 2.32)	0.39
HHF within 12 months	103 (8.2)	99 (7.8)	0.96 (0.73 to 1.26)	0.76
MACCE within 30 days	49 (3.9)	48 (3.8)	0.98 (0.65 to 1.45)	0.90
All-cause mortality within 30 days	23 (1.8)	26 (2.1)	1.12 (0.64 to 1.97)	0.68
Reinfarction within 30 days	11 (0.9)	7 (0.6)	0.63 (0.25 to 1.63)	0.34
Unplanned revascularisation within 30 days	11 (0.9)	12 (1.0)	1.09 (0.48 to 2.46)	0.84
Stroke within 30 days	4 (0.3)	5 (0.4)	1.25 (0.33 to 4.64)	0.74
ICD implantation within 12 months	6 (0.5)	8 (0.6)	1.33 (0.46 to 3.81)	0.60
Repeat HHF, mean count (SD)	0.11 (0.40)	0.11 (0.38)	0.94 (0.72 to 1.23)	0.66
Repeat HHF and cardiac death, mean count (SD)	0.14 (0.47)	0.14 (0.47)	1.01 (0.77 to 1.31)	0.96

For time-to-event outcomes, Kaplan-Meier estimates of the n (%) with the outcome at 30 days/12 months are presented.
Abbreviations: RIC = remote ischemic conditioning; CI = confidence interval; HHF = hospitalisation for heart failure; MACCE = composite of all-cause mortality, reinfarction, unplanned revascularisation, and stroke; ICD = implantable cardioverter-defibrillator; SD = standard deviation

Table S5. Subgroup Analyses for the CONDI-2 trial (ITT Analysis).

	**Control** (n=1272)	**RIC** (n=1278)	**Treatment effect, RIC vs Control (95% CI)**	**Interaction p-value**
Age (years), n (%)				
<55	16 (4.7)	12 (3.7)	0.78 (0.37 to 1.65)	
55-<65	26 (6.8)	30 (8.5)	1.26 (0.75 to 2.14)	
65-<75	38 (12.1)	40 (11.2)	0.92 (0.59 to 1.43)	
≥75	49 (22.0)	48 (21.2)	0.94 (0.63 to 1.40)	0.71
Medically treated diabetes, n (%)				
No	103 (9.1)	106 (9.5)	1.05 (0.80 to 1.38)	
Yes	25 (20.7)	24 (15.8)	0.75 (0.43 to 1.31)	0.28
TIMI flow grade at admission, n (%)				
TIMI 0-1	89 (12.5)	80 (11.6)	0.92 (0.68 to 1.24)	
TIMI 2-3	20 (5.5)	27 (7.1)	1.30 (0.73 to 2.32)	0.30
Infarct location, n (%)				
Left anterior descending	78 (16.4)	79 (18.4)	1.14 (0.83 to 1.56)	
Other	32 (5.3)	28 (4.3)	0.81 (0.49 to 1.35)	0.26
First medical contact to balloon time (min), n (%)				
<90	39 (8.2)	47 (9.9)	1.22 (0.80 to 1.87)	
90-<120	35 (10.3)	29 (8.5)	0.81 (0.50 to 1.33)	
120-≤720	36 (13.6)	30 (12.0)	0.88 (0.54 to 1.42)	0.40
**Exploratory analyses**				
Gender, n (%)				
Male	89 (9.2)	94 (9.9)	1.08 (0.81 to 1.45)	
Female	40 (13.7)	36 (11.4)	0.81 (0.52 to 1.27)	0.29
Smoking status, n (%)				
Current smoker	46 (8.1)	52 (9.6)	1.18 (0.80 to 1.76)	
Ex-smoker	38 (11.0)	36 (9.9)	0.89 (0.57 to 1.41)	
Never smoked	39 (12.0)	39 (12.0)	1.00 (0.64 to 1.56)	0.65
BMI (kg/m^2), n (%)				
<25	40 (10.3)	44 (11.3)	1.10 (0.72 to 1.68)	
≥25	85 (10.5)	83 (10.0)	0.96 (0.71 to 1.30)	0.61
Symptom to balloon time (min), n (%)				
<150	31 (7.0)	35 (8.2)	1.18 (0.73 to 1.92)	
150-<240	35 (12.1)	27 (8.6)	0.71 (0.43 to 1.17)	
240-≤720	35 (10.9)	39 (12.4)	1.14 (0.72 to 1.79)	0.28
Hypertension, n (%)				
No	63 (8.2)	66 (9.2)	1.12 (0.79 to 1.58)	
Yes	65 (13.2)	64 (11.8)	0.88 (0.63 to 1.25)	0.34
Beta-blockers, n (%)				
No	102 (9.6)	112 (10.6)	1.11 (0.85 to 1.46)	
Yes	22 (12.0)	17 (8.4)	0.68 (0.36 to 1.28)	0.16
ACE-inhibitors, n (%)				
No	99 (9.4)	105 (9.8)	1.05 (0.80 to 1.38)	
Yes	25 (12.6)	24 (12.4)	0.98 (0.56 to 1.72)	0.84

	**Control** (n=1272)	**RIC** (n=1278)	**Treatment effect, RIC vs Control (95% CI)**	**Interaction p-value**
Angiotensin II receptor blockers, n (%)				
No	107 (9.6)	109 (10.1)	1.06 (0.81 to 1.39)	
Yes	17 (12.7)	20 (10.9)	0.83 (0.43 to 1.58)	0.49
Statins, n (%)				
No	86 (9.0)	96 (10.1)	1.13 (0.84 to 1.51)	
Yes	39 (13.3)	33 (10.8)	0.80 (0.51 to 1.28)	0.22
Metformin, n (%)				
No	103 (9.0)	108 (9.6)	1.07 (0.82 to 1.40)	
Yes	14 (17.1)	12 (11.6)	0.66 (0.31 to 1.43)	0.25
Sulphonylurea, n (%)				
No	115 (9.5)	118 (9.8)	1.03 (0.79 to 1.33)	
Yes	2 (11.8)	2 (10.5)	0.86 (0.12 to 6.11)	0.86
Insulin, n (%)				
No	118 (9.7)	116 (9.5)	0.98 (0.76 to 1.27)	
Yes	8 (24.6)	13 (30.6)	1.32 (0.55 to 3.20)	0.53
Ticagrelor, n (%)				
No	50 (11.8)	56 (13.2)	1.12 (0.77 to 1.64)	
Yes	79 (9.5)	74 (8.8)	0.93 (0.68 to 1.28)	0.46

This Table presents Kaplan-Meier estimates of the n (%) experiencing hospitalisation for heart failure or cardiovascular death within 12 months.
Abbreviations: RIC = remote ischemic conditioning; CI = confidence interval

Table S6. Baseline characteristics of Patients and PPCI Procedure Details in the CONDI-2 Trial (PP Analysis).

	**Control** (n=1062)		**RIC** (n=984)	
**Demographics**				
Age (years), mean (SD)	63.3	(11.7)	63.9	(11.5)
Missing, n	20		40	
Female, n (%)	228	(21.5)	236	(24.0)
Missing, n	0		0	
Body mass index (BMI; kg/m2), mean (SD)	27.5	(4.5)	27.5	(4.8)
Missing, n	49		34	
Current smokers, n (%)	488	(46.6)	443	(46.0)
Missing, n	14		22	
**Comorbidity, n (%)**				
Hypertension	421	(39.7)	418	(42.5)
Missing, n	1		1	
Previous acute myocardial infarction (MI)	117	(11.0)	97	(9.9)
Missing, n	1		1	
Hypercholesterolemia	258	(24.3)	235	(23.9)
Missing, n	2		2	
Medically treated diabetes	107	(10.1)	121	(12.3)
Missing, n	0		1	
Family history of ischaemic heart disease (IHD)	329	(32.2)	317	(33.6)
Not known, n	39		40	
**Medications at admission for PPCI, n (%)**				
Sulphonylurea	18	(1.7)	16	(1.7)
Missing, n	26		25	
Metformin	71	(6.9)	88	(9.2)
Missing, n	26		25	
Insulin	28	(2.6)	29	(3.0)
Missing, n	0		1	
Clopidogrel	34	(3.2)	24	(2.4)
Missing, n	0		3	
Ticagrelor	4	(0.4)	11	(1.1)
Missing, n	0		3	
Prasugrel	0	(0.0)	0	(0.0)
Missing, n	0		3	
Statins	239	(22.5)	220	(22.4)
Missing, n	1		4	
Aspirin	173	(16.3)	162	(16.5)
Missing, n	1		3	
Beta-blockers	155	(14.7)	155	(15.8)

Missing, n	6		3	
ACE inhibitors/Angiotensin receptor blockers	172	(16.3)	146	(14.9)
Missing, n	6		3	
**BP and Killip class at randomisation**				
Systolic blood pressure (mmHg), mean (SD)	129	(24)	129	(24)
Missing, n	6		13	
Diastolic blood pressure (mmHg), mean (SD)	74	(14)	74	(15)
Missing, n	8		13	
Killip class, n (%)				
Class I	999	(94.1)	925	(94.0)
Class II	47	(4.4)	43	(4.4)
Class III	9	(0.8)	4	(0.4)
Class IV (including cardiogenic shock)	7	(0.7)	12	(1.2)
Missing, n	0		0	
**RIC procedure**				
RIC procedure started, n (%)	-		984	(100.0)
Number of complete RIC cycles administered, n (%)				
1	-		4	(0.4)
2	-		11	(1.1)
3	-		59	(6.1)
4	-		896	(92.4)
Missing, n	-		14	
**PPCI procedure**				
PPCI completed, n (%)	1062	(100.0)	984	(100.0)
TIMI flow grade at admission, n (%)				
TIMI 0	627	(59.3)	584	(59.5)
TIMI 1	76	(7.2)	54	(5.5)
TIMI 2	132	(12.5)	118	(12.0)
TIMI 3	222	(21.0)	225	(22.9)
Missing, n	5		3	
Symptom to balloon time (min), median (IQR)	172	(123 to 277)	175	(123 to 276)
Missing, n	3		6	
First medical contact to balloon time (min), median (IQR)	94	(76 to 119)	95	(75 to 118)
Missing, n	2		7	
Infarct-related coronary artery, n (%)				
Left anterior descending	468	(44.1)	400	(40.7)
Circumflex	147	(13.8)	133	(13.5)
Right coronary	447	(42.1)	451	(45.8)
Missing, n	0		0	
Vessels with clinically significant disease, n (%)				
1	634	(59.7)	593	(60.3)
2	300	(28.2)	252	(25.6)
3	128	(12.1)	139	(14.1)

Missing, n	0		0	
Stenting of culprit lesion by PPCI, n (%)	969	(91.2)	892	(90.7)
Missing, n	0		0	
Supplementary staged PCI, n (%)	187	(17.6)	148	(15.0)
Supplementary staged CABG, n (%)	35	(3.3)	43	(4.4)
TIMI flow grade after procedure, n (%)				
TIMI 0	11	(1.0)	10	(1.0)
TIMI 1	13	(1.2)	2	(0.2)
TIMI 2	47	(4.4)	31	(3.2)
TIMI 3	986	(93.3)	938	(95.6)
Missing, n	5		3	
**Medications given in relation to PPCI, n (%)**				
Opioids*	530	(51.6)	461	(48.9)
Missing, n	35		41	
Heparin	801	(75.6)	739	(75.3)
Missing, n	2		3	
Aspirin	1045	(98.5)	973	(99.2)
Missing, n	1		3	
Clopidogrel	227	(21.4)	208	(21.1)
Missing, n	0		0	
Ticagrelor	742	(69.9)	686	(69.7)
Missing, n	0		0	
Prasugrel	1	(0.1)	1	(0.1)
Missing, n	0		0	
Nitrates	767	(74.5)	672	(71.0)
Missing, n	33		38	
Glycoprotein IIb/IIIa inhibitor	141	(13.4)	116	(11.8)
Missing, n	7		4	
Bivalirudin	436	(41.3)	398	(40.6)
Missing, n	7		3	
Cangrelor*	129	(12.2)	113	(11.5)
Missing, n	2		0	

Abbreviations: RIC = remote ischemic conditioning; SD = standard deviation; IQR = interquartile range; BMI = body mass index; MI = myocardial infarction; IHD = ischemic heart disease; PPCI = primary percutaneous coronary intervention; BP = blood pressure; CABG = coronary artery bypass graft surgery
*Opioids and cangrelor data collected only in CONDI-2.

Table S7. Primary and Secondary Outcomes in the CONDI-2 Trial (PP Analysis).

	**Control** (n=1062)	**RIC** (n=984)	**Treatment effect, RIC vs Control (95% CI)**	**p-value**
**Primary outcome, n (%)**				
Combined HHF and cardiac death within 12 months	104 (9.9)	100 (10.3)	1.04 (0.79 to 1.37)	0.78
Cardiac death within 12 months	25 (2.4)	28 (2.9)	1.21 (0.71 to 2.08)	0.48
HHF within 12 months	95 (9.0)	88 (9.0)	1.00 (0.75 to 1.34)	0.99
**Secondary outcomes, n (%)**				
MACCE within 12 months	90 (8.6)	82 (8.4)	0.99 (0.73 to 1.33)	0.93
All-cause mortality within 12 months	36 (3.4)	44 (4.5)	1.33 (0.85 to 2.06)	0.21
Reinfarction within 12 months	23 (2.2)	14 (1.5)	0.66 (0.34 to 1.28)	0.22
Unplanned revascularisation within 12 months	26 (2.5)	21 (2.2)	0.88 (0.49 to 1.56)	0.66
Stroke within 12 months	12 (1.2)	11 (1.2)	1.00 (0.44 to 2.26)	0.99
Combined HHF and cardiac death within 30 days	83 (7.9)	78 (8.0)	1.01 (0.74 to 1.38)	0.93
Cardiac death within 30 days	15 (1.4)	17 (1.7)	1.22 (0.61 to 2.45)	0.57
HHF within 12 months	84 (8.0)	75 (7.7)	0.96 (0.71 to 1.32)	0.82
MACCE within 30 days	42 (4.0)	35 (3.6)	0.90 (0.57 to 1.41)	0.65
All-cause mortality within 30 days	18 (1.7)	17 (1.7)	1.02 (0.53 to 1.98)	0.95
Reinfarction within 30 days	10 (1.0)	5 (0.5)	0.54 (0.18 to 1.58)	0.26
Unplanned revascularisation within 30 days	10 (1.0)	10 (1.0)	1.08 (0.45 to 2.60)	0.86
Stroke within 30 days	4 (0.4)	4 (0.4)	1.08 (0.27 to 4.32)	0.91
ICD implantation within 12 months	5 (0.5)	7 (0.7)	1.51 (0.48 to 4.75)	0.48
Repeat HHF, mean count (SD)	0.11 (0.40)	0.11 (0.40)	1.00 (0.73 to 1.36)	0.99
Repeat HHF and Cardiac death, mean count (SD)	0.14 (0.47)	0.14 (0.48)	1.03 (0.76 to 1.40)	0.83

For time-to-event outcomes, Kaplan-Meier estimates of the n (%) with the outcome at 30 days/12 months are presented.
Abbreviations: RIC = remote ischemic conditioning; CI = confidence interval; HHF = hospitalization for heart failure; MACCE = composite of all-cause mortality, reinfarction, unplanned revascularisation, and stroke; ICD = implantable cardioverter-defibrillator; SD = standard deviation

Table S8. Subgroup Analyses for the CONDI-2 Trial (PP Analysis).

	**Control** (n=1062)	**RIC** (n=984)	**Treatment effect, RIC vs. Control (95% CI)**	**Interaction p-value**
Age (years), n (%)				
<55	10 (3.7)	9 (3.7)	1.01 (0.41 to 2.50)	
55-<65	24 (7.4)	22 (7.9)	1.06 (0.60 to 1.90)	
65-<75	30 (11.0)	35 (12.3)	1.12 (0.69 to 1.83)	
≥75	40 (21.3)	34 (20.0)	0.92 (0.58 to 1.46)	0.95
Medically treated diabetes, n (%)				
No	85 (9.0)	81 (9.5)	1.06 (0.78 to 1.44)	
Yes	19 (18.1)	19 (16.1)	0.86 (0.46 to 1.63)	0.56
TIMI flow grade at admission, n (%)				
TIMI 0-1	85 (12.2)	76 (12.0)	0.98 (0.72 to 1.34)	
TIMI 2-3	19 (5.4)	24 (7.0)	1.32 (0.72 to 2.41)	0.40
Infarct location, n (%)				
Left anterior descending	74 (15.9)	73 (18.4)	1.18 (0.85 to 1.62)	
Other	30 (5.1)	27 (4.7)	0.91 (0.54 to 1.53)	0.42
First medical contact to balloon time (min), n (%)				
<90	36 (7.7)	44 (10.4)	1.36 (0.88 to 2.12)	
90-<120	34 (10.3)	27 (8.7)	0.83 (0.50 to 1.37)	
120-≤720	34 (13.3)	29 (12.5)	0.94 (0.57 to 1.54)	0.30
**Exploratory analyses**				
Gender, n (%)				
Male	76 (9.2)	70 (9.4)	1.03 (0.74 to 1.42)	
Female	28 (12.4)	30 (12.8)	1.03 (0.62 to 1.73)	0.99
Smoking status, n (%)				
Current smoker	35 (7.2)	43 (9.8)	1.37 (0.88 to 2.15)	
Ex-smoker	33 (11.3)	22 (7.9)	0.69 (0.40 to 1.18)	
Never smoked	32 (12.2)	33 (13.9)	1.15 (0.71 to 1.87)	0.14
BMI (kg/m^2), n (%)				
<25	32 (10.3)	30 (10.5)	1.02 (0.62 to 1.68)	
≥25	69 (9.9)	68 (10.4)	1.05 (0.75 to 1.46)	0.94
Symptom to balloon time (min), n (%)				
<150	29 (6.7)	31 (8.1)	1.23 (0.74 to 2.03)	
150-<240	33 (11.6)	27 (9.6)	0.82 (0.49 to 1.37)	
240-≤720	33 (10.7)	37 (12.6)	1.18 (0.74 to 1.89)	0.48
Hypertension, n (%)				
No	54 (8.5)	54 (9.6)	1.15 (0.79 to 1.67)	
Yes	50 (12.1)	46 (11.2)	0.92 (0.61 to 1.37)	0.43
Beta-blockers, n (%)				
No	82 (9.2)	89 (10.9)	1.20 (0.89 to 1.62)	
Yes	20 (13.1)	11 (7.3)	0.53 (0.25 to 1.10)	0.04
ACE-inhibitors, n (%)				
No	83 (9.4)	84 (10.2)	1.08 (0.80 to 1.46)	
Yes	19 (11.3)	16 (11.0)	0.96 (0.50 to 1.87)	0.76

	**Control** (n=1062)	**RIC** (n=984)	**Treatment effect, RIC vs. Control (95% CI)**	**Interaction p-value**
Angiotensin II receptor blockers, n (%)				
No	89 (9.5)	83 (9.9)	1.05 (0.78 to 1.41)	
Yes	13 (11.3)	17 (12.3)	1.06 (0.52 to 2.19)	0.97
Statins, n (%)				
No	72 (8.8)	77 (10.2)	1.17 (0.85 to 1.61)	
Yes	31 (13.2)	23 (10.6)	0.79 (0.46 to 1.35)	0.22
Metformin, n (%)				
No	87 (9.1)	83 (9.6)	1.06 (0.79 to 1.43)	
Yes	10 (14.2)	9 (10.6)	0.71 (0.29 to 1.75)	0.41
Sulphonylurea, n (%)				
No	95 (9.4)	90 (9.6)	1.03 (0.77 to 1.37)	
Yes	2 (11.8)	2 (13.3)	1.09 (0.15 to 7.72)	0.95
Insulin, n (%)				
No	99 (9.7)	90 (9.5)	0.99 (0.74 to 1.31)	
Yes	5 (18.2)	10 (34.5)	2.09 (0.71 to 6.12)	0.18
Ticagrelor, n (%)				
No	103 (9.8)	100 (10.4)	1.06 (0.81 to 1.40)	
Yes	1 (25.0)	0 (0.0)	-	-

This Table presents Kaplan-Meier estimates of the n (%) experiencing hospitalization for heart failure or cardiovascular death within 12 months.
Abbreviations: RIC = remote ischemic conditioning; CI = confidence interval

Table S9. Baseline Characteristics of Patients and PPCI Procedure Details in the ERIC-PPCI Trial (ITT Analysis).

	Control (n=1297)		RIC (n=1268)	
**Demographics**				
Age (years), mean (SD)	63.4	(12.3)	64.1	(12.1)
Missing, n	20		22	
Female, n (%)	282	(21.7)	289	(22.8)
Missing, n	0		0	
Body mass index (BMI; kg/m2), mean (SD)	27.7	(5.0)	27.6	(5.0)
Missing, n	73		74	
Current smokers, n (%)	444	(36.0)	444	(36.9)
Missing, n	64		65	
**Comorbidity, n (%)**				
Hypertension	519	(40.7)	548	(44.2)
Missing, n	23		27	
Previous acute myocardial infarction (MI)	112	(8.7)	133	(10.6)
Missing, n	14		14	
Hypercholesterolemia	368	(29.8)	379	(31.2)
Missing, n	63		54	
Medically treated diabetes	141	(11.0)	146	(11.6)
Missing, n	11		11	
Family history of ischaemic heart disease (IHD)	430	(36.8)	446	(40.2)
Not known, n	129		158	
**Medications at admission for PPCI, n (%)**				
Sulphonylurea	44	(3.4)	41	(3.3)
Missing, n	16		12	
Metformin	113	(8.8)	115	(9.1)
Missing, n	17		10	
Insulin	41	(3.2)	38	(3.0)
Missing, n	18		11	
Clopidogrel	45	(3.5)	61	(4.9)
Missing, n	18		14	
Ticagrelor	47	(3.7)	44	(3.5)
Missing, n	17		15	
Prasugrel	3	(0.2)	3	(0.2)
Missing, n	20		14	
Statins	352	(27.5)	355	(28.3)
Missing, n	18		15	
Aspirin	234	(18.3)	260	(20.7)
Missing, n	17		14	
Beta-blockers	197	(15.4)	200	(16.0)

Missing, n	21		17	
ACE inhibitors/Angiotensin receptor blockers	252	(19.7)	271	(21.7)
Missing, n	19		17	
**BP and Killip class at randomisation**				
Systolic blood pressure (mmHg), mean (SD)	132	(24)	133	(23)
Missing, n	68		74	
Diastolic blood pressure (mmHg), mean (SD)	80	(16)	79	(15)
Missing, n	70		76	
Killip class, n (%)				
Class I	1269	(97.8)	1237	(97.6)
Class II	21	(1.6)	19	(1.5)
Class III	1	(0.1)	3	(0.2)
Class IV (including cardiogenic shock)	6	(0.5)	9	(0.7)
Missing, n	0		0	
**RIC procedure**				
RIC procedure started, n (%)	-		1251	(98.7)
Missing, n	-		0	
Number of complete RIC cycles administered, n (%)				
1	-		33	(2.7)
2	-		28	(2.3)
3	-		80	(6.5)
4	-		1082	(88.5)
Missing, n	-		28	
**PPCI procedure**				
PPCI completed, n (%)	1168	(90.1)	1142	(90.1)
TIMI flow grade at admission, n (%)				
TIMI 0	875	(76.6)	840	(74.9)
TIMI 1	76	(6.6)	87	(7.8)
TIMI 2	83	(7.3)	89	(7.9)
TIMI 3	109	(9.5)	106	(9.4)
Missing, n	25		20	
Symptom to balloon time (min), median (IQR)	182	(133 to 279)	181	(137 to 281)
Missing, n	154		161	
First medical contact to balloon time (min), median (IQR)	109	(90 to 132)	111	(92 to 137)
Missing, n	172		184	
Infarct-related coronary artery, n (%)				
Left anterior descending	493	(42.2)	477	(41.8)
Circumflex	144	(12.3)	146	(12.8)
Right coronary	529	(45.3)	515	(45.1)
Other	2	(0.2)	3	(0.3)
Missing, n	0		1	
Vessels with clinically significant disease, n (%)				
0	1	(0.1)	2	(0.2)

1	660	(56.5)	623	(54.6)
2	354	(30.3)	363	(31.8)
3	153	(13.1)	154	(13.5)
Missing, n	0		0	
Stenting of culprit lesion by PPCI, n (%)	1114	(95.4)	1096	(96.1)
Missing, n	0		1	
Supplementary staged PCI, n (%)	102	(8.7)	97	(8.5)
Supplementary staged CABG, n (%)	16	(1.4)	15	(1.3)
TIMI flow grade after procedure, n (%)				
TIMI 0	14	(1.2)	15	(1.3)
TIMI 1	7	(0.6)	7	(0.6)
TIMI 2	63	(5.5)	53	(4.7)
TIMI 3	1054	(92.6)	1044	(93.3)
Missing, n	30		23	
**Medications given in relation to PPCI, n (%)**				
Opioids*	0		0	
Missing, n	1168		1142	
Heparin	1080	(92.5)	1071	(93.8)
Missing, n	1		0	
Aspirin	1075	(92.2)	1056	(92.7)
Missing, n	2		3	
Clopidogrel	380	(32.6)	348	(30.5)
Missing, n	2		1	
Ticagrelor	791	(67.8)	793	(69.4)
Missing, n	1		0	
Prasugrel	102	(8.7)	96	(8.4)
Missing, n	2		1	
Nitrates	969	(83.0)	949	(83.1)
Missing, n	1		0	
Glycoprotein IIb/IIIa inhibitor	307	(26.3)	274	(24.0)
Missing, n	1		0	
Bivalirudin	63	(5.4)	63	(5.5)
Missing, n	1		0	
Cangrelor*	0		0	
Missing, n	1168		1142	

Abbreviations: RIC = remote ischemic conditioning; SD = standard deviation; IQR = interquartile range; BMI = body mass index; MI = myocardial infarction; IHD = ischemic heart disease; PPCI = primary percutaneous coronary intervention; BP = blood pressure; CABG = coronary artery bypass graft surgery
*Opioids and cangrelor data collected only in CONDI-2.

Table S10. Primary and Secondary Outcomes in the ERIC-PPCI Trial (ITT Analysis).

	**Control** (n=1297)	**RIC** (n=1268)	**Treatment effect, RIC vs Control (95% CI)**	**p-value**
**Primary outcome, n (%)**				
Combined HHF and cardiac death within 12 months	91 (7.0)	109 (8.6)	1.23 (0.93 to 1.63)	0.14
Cardiac death within 12 months	38 (2.9)	36 (2.8)	0.97 (0.62 to 1.54)	0.91
HHF within 12 months	64 (5.0)	78 (6.2)	1.25 (0.90 to 1.74)	0.18
**Secondary outcomes, n (%)**				
MACCE within 12 months	88 (6.8)	101 (8.0)	1.18 (0.89 to 1.57)	0.25
All-cause mortality within 12 months	52 (4.0)	60 (4.8)	1.19 (0.82 to 1.72)	0.37
Reinfarction within 12 months	16 (1.3)	16 (1.3)	1.03 (0.51 to 2.06)	0.94
Unplanned revascularisation within 12 months	32 (2.5)	35 (2.9)	1.13 (0.70 to 1.82)	0.62
Stroke within 12 months	6 (0.5)	9 (0.7)	1.55 (0.55 to 4.34)	0.41
Combined HHF and cardiac death within 30 days	82 (6.3)	101 (8.0)	1.27 (0.95 to 1.70)	0.11
Cardiac death within 30 days	27 (2.1)	33 (2.6)	1.25 (0.75 to 2.09)	0.38
HHF within 12 months	59 (4.6)	72 (5.7)	1.25 (0.89 to 1.77)	0.20
MACCE within 30 days	48 (3.7)	61 (4.8)	1.31 (0.90 to 1.91)	0.16
All-cause mortality within 30 days	29 (2.2)	37 (2.9)	1.31 (0.81 to 2.13)	0.28
Reinfarction within 30 days	10 (0.8)	12 (1.0)	1.23 (0.53 to 2.85)	0.63
Unplanned revascularisation within 30 days	18 (1.4)	17 (1.4)	0.97 (0.50 to 1.88)	0.93
Stroke within 30 days	3 (0.2)	5 (0.4)	1.71 (0.41 to 7.18)	0.46
ICD implantation within 12 months	22 (1.8)	29 (2.4)	1.36 (0.78 to 2.35)	0.27
Repeat HHF, mean count (SD)	0.06 (0.32)	0.07 (0.28)	1.10 (0.78 to 1.55)	0.59
Repeat HHF and cardiac death, mean count (SD)	0.09 (0.38)	0.10 (0.33)	1.06 (0.79 to 1.42)	0.70

For time-to-event outcomes, Kaplan-Meier estimates of the n (%) with the outcome at 30 days/12 months are presented.
Abbreviations: RIC = remote ischemic conditioning; CI = confidence interval; HHF = hospitalization for heart failure; MACCE = composite of all-cause mortality, reinfarction, unplanned revascularisation, and stroke; ICD = implantable cardioverter-defibrillator; SD = standard deviation

Table S11. Subgroup Analyses for the ERIC-PPCI Trial (ITT analysis).

	**Control** (n=1297)	**RIC** (n=1268)	**Treatment effect, RIC vs. Control (95% CI)**	**Interaction p-value**
Age (years), n (%)				
<55	8 (2.3)	10 (3.3)	1.47 (0.58 to 3.71)	
55-<65	14 (3.7)	18 (5.4)	1.45 (0.72 to 2.92)	
65-<75	20 (6.6)	25 (6.9)	1.06 (0.59 to 1.90)	
≥75	30 (12.2)	37 (14.9)	1.23 (0.76 to 1.99)	0.89
Medically treated diabetes, n (%)				
No	76 (6.6)	87 (7.9)	1.19 (0.87 to 1.61)	
Yes	13 (9.3)	19 (13.1)	1.43 (0.71 to 2.90)	0.63
TIMI flow grade at admission, n (%)				
TIMI 0-1	67 (6.8)	89 (9.3)	1.37 (1.00 to 1.88)	
TIMI 2-3	16 (6.1)	15 (5.7)	0.95 (0.47 to 1.92)	0.35
Infarct location, n (%)				
Left anterior descending	54 (10.4)	68 (13.5)	1.31 (0.92 to 1.88)	
Other	33 (4.7)	38 (5.4)	1.17 (0.74 to 1.87)	0.71
First medical contact to balloon time (min), n (%)				
<90	7 (2.7)	9 (3.6)	1.34 (0.50 to 3.61)	
90-<120	28 (6.2)	31 (8.0)	1.31 (0.78 to 2.18)	
120-≤720	37 (9.2)	45 (10.2)	1.12 (0.72 to 1.73)	0.88
**Exploratory analyses**				
Gender, n (%)				
Male	63 (6.2)	78 (8.0)	1.29 (0.93 to 1.80)	
Female	28 (9.9)	31 (10.8)	1.08 (0.65 to 1.81)	0.57
Smoking status, n (%)				
Current smoker	18 (4.1)	26 (5.9)	1.45 (0.80 to 2.65)	
Ex-smoker	32 (8.0)	29 (7.6)	0.95 (0.57 to 1.57)	
Never smoked	34 (8.8)	41 (10.9)	1.26 (0.80 to 1.99)	0.53
BMI (kg/m^2), n (%)				
<25	24 (6.1)	30 (8.0)	1.32 (0.77 to 2.26)	
≥25	56 (6.8)	65 (8.0)	1.18 (0.83 to 1.69)	0.74
Symptom to balloon time (min), n (%)				
<150	15 (3.6)	27 (7.5)	2.10 (1.12 to 3.94)	
150-<240	26 (7.2)	32 (8.1)	1.13 (0.67 to 1.89)	
240-≤720	31 (9.0)	26 (7.9)	0.87 (0.52 to 1.46)	0.10
Hypertension, n (%)				
No	47 (6.2)	42 (6.1)	0.97 (0.64 to 1.47)	
Yes	40 (7.7)	61 (11.2)	1.47 (0.99 to 2.19)	0.16
Beta-blockers, n (%)				
No	67 (6.2)	78 (7.4)	1.20 (0.87 to 1.66)	
Yes	18 (9.1)	26 (13.0)	1.46 (0.80 to 2.66)	0.58
ACE-inhibitors, n (%)				
No	65 (6.3)	76 (7.8)	1.23 (0.88 to 1.72)	
Yes	20 (8.0)	28 (10.4)	1.32 (0.74 to 2.34)	0.84

	Control (n=1297)	RIC (n=1268)	Treatment effect, RIC vs. Control (95% CI)	Interaction p-value
Angiotensin II receptor blockers, n (%)				
No	75 (6.5)	92 (8.2)	1.27 (0.94 to 1.73)	
Yes	11 (9.7)	12 (10.0)	1.04 (0.46 to 2.36)	0.65
Statins, n (%)				
No	62 (6.7)	73 (8.1)	1.22 (0.87 to 1.71)	
Yes	25 (7.1)	31 (8.8)	1.24 (0.73 to 2.11)	0.95
Metformin, n (%)				
No	81 (7.0)	93 (8.2)	1.18 (0.88 to 1.59)	
Yes	6 (5.3)	12 (10.5)	2.01 (0.76 to 5.36)	0.31
Sulphonylurea, n (%)				
No	84 (6.8)	100 (8.3)	1.22 (0.91 to 1.63)	
Yes	3 (6.8)	5 (12.4)	1.79 (0.43 to 7.50)	0.61
Insulin, n (%)				
No	80 (6.5)	97 (8.0)	1.24 (0.92 to 1.67)	
Yes	7 (17.2)	8 (21.1)	1.24 (0.45 to 3.41)	0.99
Ticagrelor, n (%)				
No	34 (8.8)	35 (10.0)	1.13 (0.71 to 1.81)	
Yes	45 (5.6)	57 (7.2)	1.07 (0.87 to 1.89)	0.70

This Tables presents Kaplan-Meier estimates of the n (%) experiencing hospitalization for heart failure or cardiovascular death within 12 months.
Abbreviations: RIC = remote ischemic conditioning; CI = confidence interval

Table S12. Baseline Characteristics of Patients and PPCI Procedure Details in the ERIC-PPCI Trial (PP Analysis).

	Control (n=1143)		RIC (n=1024)	
**Demographics**				
Age (years), mean (SD)	63.3	(12.1)	64.4	(11.8)
Missing, n	12		11	
Female, n (%)	239	(20.9)	221	(21.6)
Missing, n	0		0	
Body mass index (BMI; kg/m2), mean (SD)	27.7	(5.0)	27.6	(4.9)
Missing, n	63		48	
Current smokers, n (%)	408	(37.4)	356	(36.5)
Missing, n	52		49	
**Comorbidity, n (%)**				
Hypertension	448	(39.9)	442	(44.1)
Missing, n	20		21	
Previous acute myocardial infarction (MI)	87	(7.7)	100	(9.9)
Missing, n	11		11	
Hypercholesterolemia	325	(29.8)	303	(30.8)
Missing, n	53		41	
Medically treated diabetes	120	(10.6)	126	(12.4)
Missing, n	9		8	
Family history of ischaemic heart disease (IHD)	386	(37.3)	368	(40.8)
Not known, n	109		121	
**Medications at admission for PPCI, n (%)**				
Sulphonylurea	37	(3.3)	37	(3.6)
Missing, n	11		8	
Metformin	98	(8.7)	98	(9.6)
Missing, n	12		6	
Insulin	35	(3.1)	35	(3.4)
Missing, n	13		7	
Clopidogrel	41	(3.6)	50	(4.9)
Missing, n	14		9	
Ticagrelor	46	(4.1)	39	(3.8)
Missing, n	12		9	
Prasugrel	3	(0.3)	2	(0.2)
Missing, n	15		9	
Statins	308	(27.3)	284	(28.0)
Missing, n	14		8	
Aspirin	199	(17.6)	217	(21.4)
Missing, n	12		9	
Beta-blockers	168	(14.9)	165	(16.3)

Missing, n	17		12	
ACE inhibitors/Angiotensin receptor blockers	213	(18.9)	206	(20.4)
Missing, n	15		12	
Beta-blockers	168	(14.9)	165	(16.3)
**BP and Killip class at randomisation**				
Systolic blood pressure (mmHg), mean (SD)	132	(24)	133	(23)
Missing, n	48		42	
Diastolic blood pressure (mmHg), mean (SD)	79	(16)	79	(15)
Missing, n	50		43	
Killip class, n (%)				
Class I	1119	(97.9)	996	(97.3)
Class II	18	(1.6)	17	(1.7)
Class III	0	(0.0)	3	(0.3)
Class IV (including cardiogenic shock)	6	(0.5)	8	(0.8)
Missing, n	0		0	
**RIC procedure**				
RIC procedure started, n (%)	-		1024	(100.0)
Missing, n	-		0	
Number of complete RIC cycles administered, n (%)				
1	-		4	(0.4)
2	-		4	(0.4)
3	-		42	(4.2)
4	-		961	(95.1)
Missing, n	-		13	
**PPCI procedure**				
PPCI completed, n (%)	1143	(100.0)	1024	(100.0)
TIMI flow grade at admission, n (%)				
TIMI 0	858	(76.7)	746	(74.3)
TIMI 1	74	(6.6)	78	(7.8)
TIMI 2	82	(7.3)	80	(8.0)
TIMI 3	105	(9.4)	100	(10.0)
Missing, n	24		20	
Symptom to balloon time (min), median (IQR)	182	(133 to 279)	181	(137 to 284)
Missing, n	24		33	
First medical contact to balloon time (min), median (IQR)	109	(90 to 132)	112	(92 to 137)
Missing, n	41		54	
Infarct-related coronary artery, n (%)				
Left anterior descending	480	(42.0)	438	(42.8)
Circumflex	140	(12.2)	127	(12.4)
Right coronary	522	(45.7)	456	(44.6)
Other	1	(0.1)	2	(0.2)
Missing, n	0		1	
Vessels with clinically significant disease, n (%)				

0	1	(0.1)	2	(0.2)
1	644	(56.3)	559	(54.6)
2	346	(30.3)	324	(31.6)
3	152	(13.3)	139	(13.6)
Missing, n	0		0	
Stenting of culprit lesion by PPCI, n (%)	1090	(95.4)	981	(95.9)
Missing, n	0		1	
Supplementary staged PCI, n (%)	102	(8.9)	83	(8.1)
Supplementary staged CABG, n (%)	16	(1.4)	12	(1.2)
TIMI flow grade after procedure, n (%)				
TIMI 0	14	(1.3)	15	(1.5)
TIMI 1	7	(0.6)	7	(0.7)
TIMI 2	62	(5.6)	47	(4.7)
TIMI 3	1031	(92.5)	932	(93.1)
Missing, n	29		23	
**Medications administered in relation to PPCI, n (%)**				
Opioids*	0		0	
Missing, n	1143		1024	
Heparin	1055	(92.4)	958	(93.6)
Missing, n	1		0	
Aspirin	1055	(92.5)	945	(92.6)
Missing, n	2		3	
Clopidogrel	370	(32.4)	309	(30.2)
Missing, n	2		1	
Ticagrelor	782	(68.5)	719	(70.2)
Missing, n	1		0	
Prasugrel	97	(8.5)	81	(7.9)
Missing, n	2		1	
Nitrates	950	(83.2)	851	(83.1)
Missing, n	1		0	
Glycoprotein IIb/IIIa inhibitor	302	(26.4)	242	(23.6)
Missing, n	1		0	
Bivalirudin	63	(5.5)	60	(5.9)
Missing, n	1		0	
Cangrelor*	0		0	
Missing, n	1143		1024	

Abbreviations: RIC = remote ischemic conditioning; SD = standard deviation; IQR = interquartile range; BMI = body mass index; MI = myocardial infarction; IHD = ischemic heart disease; PPCI = primary percutaneous coronary intervention; BP = blood pressure; CABG = coronary artery bypass graft surgery
*Opioids and cangrelor data collected only in CONDI-2.

Table S13. Primary and Secondary Outcomes in the ERIC-PPCI Trial (PP Analysis).

	**Control** (n=1143)	**RIC** (n=1024)	**Treatment effect, RIC vs Control (95% CI)**	**p-value**
**Primary outcome, n (%)**				
Combined HHF and cardiac death within 12 months	178 (8.1)	179 (9.0)	1.11 (0.90 to 1.36)	0.35
Cardiac death within 12 months	50 (2.3)	51 (2.6)	1.12 (0.76 to 1.66)	0.56
HHF within 12 months	154 (7.0)	147 (7.4)	1.05 (0.83 to 1.31)	0.70
**Secondary outcomes, n (%)**				
MACCE within 12 months	159 (7.3)	160 (8.0)	1.11 (0.89 to 1.38)	0.35
All-cause mortality within 12 months	73 (3.3)	85 (4.3)	1.29 (0.94 to 1.76)	0.12
Reinfarction within 12 months	37 (1.7)	27 (1.4)	0.80 (0.49 to 1.32)	0.38
Unplanned revascularisation within 12 months	54 (2.5)	51 (2.6)	1.05 (0.71 to 1.53)	0.81
Stroke within 12 months	17 (0.8)	20 (1.0)	1.30 (0.68 to 2.47)	0.43
Combined HHF and cardiac death within 30 days	153 (7.0)	151 (7.5)	1.08 (0.87 to 1.36)	0.48
Cardiac death within 30 days	33 (1.5)	38 (1.9)	1.27 (0.80 to 2.02)	0.32
HHF within 12 months	140 (6.4)	130 (6.5)	1.02 (0.80 to 1.29)	0.89
MACCE within 30 days	78 (3.6)	79 (4.0)	1.12 (0.82 to 1.53)	0.49
All-cause mortality within 30 days	37 (1.7)	40 (2.0)	1.19 (0.76 to 1.86)	0.44
Reinfarction within 30 days	19 (0.9)	14 (0.7)	0.81 (0.41 to 1.62)	0.55
Unplanned revascularisation within 30 days	26 (1.2)	24 (1.2)	1.02 (0.59 to 1.78)	0.94
Stroke within 30 days	7 (0.3)	9 (0.5)	1.42 (0.53 to 3.80)	0.49
ICD implantation within 12 months	24 (1.1)	28 (1.4)	1.28 (0.75 to 2.21)	0.37
Repeat HHF, mean count (SD)	0.09 (0.37)	0.09 (0.35)	1.00 (0.79 to 1.27)	0.99
Repeat HHF and cardiac death, mean count (SD)	0.11 (0.43)	0.11 (0.41)	1.03 (0.82 to 1.29)	0.83

For time-to-event outcomes, Kaplan-Meier estimates of the n (%) with the outcome at 30 days/12 months are presented.
Abbreviations: RIC = remote ischemic conditioning; CI = confidence interval; HHF = hospitalization for heart failure; MACCE = composite of all-cause mortality, reinfarction, unplanned revascularisation, and stroke; ICD = implantable cardioverter-defibrillator; SD = standard deviation

Table S14. Subgroup Analyses for the ERIC-PPCI Trial (PP Analysis).

	**Control** (n=1143)	**RIC** (n=1024)	**Treatment effect, RIC vs. Control (95% CI)**	**Interaction p-value**
Age (years), n (%)				
<55	18 (3.1)	14 (2.9)	0.93 (0.46 to 1.87)	
55-<65	37 (5.6)	34 (6.1)	1.09 (0.69 to 1.74)	
65-<75	48 (8.8)	55 (9.5)	1.09 (0.74 to 1.60)	
≥75	64 (16.0)	66 (17.4)	1.09 (0.77 to 1.54)	0.98
Medically treated diabetes, n (%)				
No	148 (7.5)	143 (8.2)	1.09 (0.86 to 1.37)	
Yes	29 (12.9)	35 (14.4)	1.09 (0.67 to 1.79)	0.98
TIMI flow grade at admission, n (%)				
TIMI 0-1	142 (8.7)	142 (9.8)	1.12 (0.89 to 1.41)	
TIMI 2-3	31 (5.8)	35 (6.7)	1.18 (0.73 to 1.91)	0.85
Infarct location, n (%)				
Left anterior descending	120 (12.7)	127 (15.2)	1.22 (0.95 to 1.57)	
Other	58 (4.6)	52 (4.5)	0.95 (0.65 to 1.38)	0.28
First medical contact to balloon time (min), n (%)				
<90	43 (6.0)	50 (7.8)	1.31 (0.87 to 1.97)	
90-<120	61 (7.8)	54 (8.2)	1.02 (0.70 to 1.47)	
120-≤720	70 (10.8)	69 (10.9)	1.03 (0.74 to 1.43)	0.59
**Exploratory analyses**				
Gender, n (%)				
Male	129 (7.5)	128 (8.3)	1.12 (0.87 to 1.42)	
Female	49 (10.5)	51 (11.2)	1.05 (0.71 to 1.56)	0.81
Smoking status, n (%)				
Current smoker	50 (5.6)	61 (7.7)	1.38 (0.95 to 2.00)	
Ex-smoker	57 (8.8)	44 (7.5)	0.83 (0.56 to 1.23)	
Never smoked	62 (10.5)	65 (12.0)	1.15 (0.81 to 1.63)	0.18
BMI (kg/m^2), n (%)				
<25	51 (7.8)	54 (9.2)	1.18 (0.81 to 1.73)	
≥25	116 (8.1)	117 (8.8)	1.08 (0.84 to 1.40)	0.71
Symptom to balloon time (min), n (%)				
<150	43 (5.1)	54 (7.7)	1.50 (1.01 to 2.24)	
150-<240	58 (9.1)	52 (8.2)	0.91 (0.62 to 1.32)	
240-≤720	64 (9.9)	62 (10.4)	1.05 (0.74 to 1.48)	0.18
Hypertension, n (%)				
No	93 (7.1)	88 (7.8)	1.11 (0.83 to 1.48)	
Yes	82 (9.5)	88 (10.3)	1.08 (0.80 to 1.46)	0.92
Beta-blockers, n (%)				
No	139 (7.5)	146 (8.8)	1.17 (0.93 to 1.48)	
Yes	32 (10.0)	30 (9.4)	0.93 (0.56 to 1.53)	0.41
ACE-inhibitors, n (%)				
No	139 (7.7)	143 (8.8)	1.13 (0.89 to 1.43)	
Yes	32 (8.4)	33 (9.4)	1.13 (0.69 to 1.84)	1.00

	**Control** (n=1143)	**RIC** (n=1024)	**Treatment effect, RIC vs. Control (95% CI)**	**Interaction p-value**
Angiotensin II receptor blockers, n (%)				
No	149 (7.6)	150 (8.6)	1.14 (0.91 to 1.43)	
Yes	23 (10.8)	26 (10.9)	0.97 (0.56 to 1.71)	0.61
Statins, n (%)				
No	123 (7.5)	131 (8.8)	1.18 (0.92 to 1.51)	
Yes	51 (9.4)	46 (9.2)	0.97 (0.65 to 1.45)	0.43
Metformin, n (%)				
No	152 (7.6)	151 (8.5)	1.11 (0.89 to 1.39)	
Yes	16 (9.5)	18 (9.8)	1.00 (0.51 to 1.96)	0.77
Sulphonylurea, n (%)				
No	164 (7.8)	163 (8.5)	1.09 (0.88 to 1.36)	
Yes	4 (7.4)	6 (11.8)	1.55 (0.44 to 5.49)	0.60
Insulin, n (%)				
No	166 (7.8)	159 (8.3)	1.05 (0.85 to 1.31)	
Yes	9 (14.5)	18 (28.2)	2.08 (0.93 to 4.62)	0.11
Ticagrelor, n (%)				
No	70 (6.5)	77 (7.9)	1.23 (0.89 to 1.70)	
Yes	1 (2.2)	1 (2.6)	1.18 (0.07 to 18.87)	0.98

This Tables presents Kaplan-Meier estimates of the n (%) experiencing hospitalization for heart failure or cardiovascular death within 12 months.
Abbreviations: RIC = remote ischemic conditioning; CI = confidence interval

Table S15. MI size quantified by AUC for troponin T over 48 hours for CONDI-2/ERIC-PPCI trial

	Control	RIC	Treatment effect, RIC vs Control (95% CI)	p-value
**Biomarker Substudy**				
AUC for troponin T (ng*hr/ml)				
Median (IQR)	91.9 (37.6 to 185.6)	110.3 (48.0 to 218.5)		
N with complete data	363	345		
N with multiple imputation	1,330	1,332	1.05 (0.92 to 1.18)	0.48

CONDI 2

Effect of Remote Ischaemic Conditioning on clinical outcomes in ST-elevation myocardial infarction patients undergoing primary Percutaneous Coronary Intervention: A multicentre randomised controlled clinical study

Data and Safety Monitoring Committee (DSMC) Charter

for the

Effect of Remote Ischaemic Conditioning on clinical outcomes in ST-elevation myocardial infarction patients undergoing primary Percutaneous Coronary Intervention: A multicentre randomised controlled clinical study (Condi 2) trial

Introduction

Title	Effect of Remote Ischaemic Conditioning on clinical outcomes in ST-elevation myocardial infarction patients undergoing primary Percutaneous Coronary Intervention: A multicentre randomised controlled clinical study
**Acronym**	Condi 2
**Sponsor**	Aarhus University Hospital
**Regional Scientific Ethical Committee of Central Denmark**	No. 1-10-72-167-13

Condi 2 is a randomised controlled trial to determine whether remote ischaemic conditioning (RIC) improves clinical outcomes after in patients with ST-segment elevation myocardial infarction undergoing Primary Percutaneous Coronary Intervention (PPCI). Prof Hans Erik Bøtker (Aarhus University Hospital, Dk) is the Chief Investigator and the trial is managed by the Clinical Trials Unit at Aarhus University Hospital (AUH).

The intervention is non-pharmacological. Patients are randomised to one of two groups: Conventional treatment (control) or Remote Ischaemic Conditioning (RIC). The RIC treatment is applied during ambulance transportation to the hospital. Automated CellAegis autoRIC™ cuff devices will be used to deliver the RIC protocols. The active RIC treatment consists of four 5-minute inflations of the autoRIC™ cuff on the upper arm to 200mmHg, separated by 5-minute periods when the cuff will be deflated. For patients presenting with a systolic blood pressure (SBP) ≥175mmHg, a manual blood pressure cuff will be used and inflated to 25 mmHg above systolic blood pressure.

The reporting of final results from the Condi 2 trial will be conducted in collaboration with Prof Derek Hausenloy (University College London, UK), who is Chief Investigator of the ERIC-PPCI trial , in order to maximise power to assess the impact of RIPC on clinical endpoints. In total 4300 STEMI patients will be recruited: 2300 STEMI patients will be randomised to CONDI 2 (Figure 1) and 2000 STEMI patients will be randomised to ERIC-PPCI (Figure 2)

Primary endpoint

The primary endpoint is occurrence of cardiac death and hospitalisation for heart failure (HHF) in STEMI patients 12 months after PPCI.

Major secondary endpoints

1. Cardiac death and HHF at 30 days post PPCI.
2. All-cause death at 30 days and 12 months.
3. Repeat coronary revascularisation at 30 days and 12 months.
4. Reinfarction at 30 days and 12 months.
5. Stroke at 30 days and 12 months.
6. Left ventricular function on day three and three-six months post pPCI (Echocardiography)
7. Myocardial infarct size at day 3 (72 hour area under curve serum creatinine kinase)
8. Quality of life at 6-8 weeks and 12 months (EuroQol EQ-5D-5L)

Sub-studies

1. Persisting ST-segment elevation on ECG 90 min. after primary PCI
2. Myocardial infarct size at day three (72 hour area under curve serum creatinine kinase). Furthermore, TnT in a subset of patients (Aarhus, Aalborg, Belgrade)
3. Left ventricular function on day three and three months post pPCI (Echocardiography)

Clinical outcome data will be collected at the telephone or outpatient follow-up at 6-12 weeks and one year post-PPCI. The source data for the events are print-outs from the electronic medical record of each patient. Furthermore, documents from "The West-Danish heart database" or "The East-Danish Heart database". Data documentation in Spain and Serbia are prints of the electronic or paper medical record of each patient.

Outline of scope of charter

The purpose of this document is to describe the roles and responsibilities of the independent DSMC for the Condi 2 trial, including the timing of meetings, methods of providing information to and from the DSMC, frequency and format of meetings, statistical issues and relationships with other committees.

The following documents were reviewed in preparation of this charter:

- CONDI-2 trial protocol Version 10.4 29th Oct 2015
- ERIC-PPCI trial protocol version 2, issued 24th Feb 2015
- Case report form (CRF) version 1, issued 22nd April 2014

- London School of Hygiene and Tropical Medicine (LSHTM) Template charter for DMCs, issued 7th July 2015
- DAMOCLES study group proposed charter for data monitoring committees data monitoring committees[1]

Roles and responsibilities

The DSMC role is to examine the data accumulated during progress of the Condi 2 trial and ensure that the benefit/risk ratio remains acceptable for participating patients. It is the only committee which will have access to data broken down by treatment during the trial and on this basis, the primary responsibility of the DSMC is to review interim analyses of outcome data and to recommend to the Trial Steering Committee (TSC) whether the trial needs to be changed or terminated based on these analyses.

More specifically, the duties of the DSMC will include:

- monitoring evidence for treatment differences in the primary and secondary endpoints
- monitoring evidence for treatment harm (e.g. SAEs, deaths)
- assessing the impact and relevance of external evidence, particularly any DSMC recommendations from the ongoing ERIC-PPCI trial
- deciding whether to recommend that the trial continues to recruit participants or whether recruitment should be terminated either for everyone or for some treatment groups and/or some participant subgroups
- assessing data quality, including completeness
- reviewing recruitment figures and monitor losses to follow-up
- monitoring compliance with the protocol by participants and investigators
- monitoring continuing appropriateness of patient information
- monitoring compliance with previous DSMC recommendations
- considering the ethical implications of any recommendations made by the DSMC

[1] DAMOCLES Study Group (2005) A proposed charter for clinical trial data monitoring committees: helping them to do their job well. Lancet. 365: 711-22.

DSMC input early in the trial

All potential DSMC members should have sight of the trial protocol before finalising their agreement to join the committee. Before recruitment begins the trial will have undergone review by the sponsor, scrutiny by other trial committees and a research ethics committee. Therefore, if a potential DSMC member has major reservations about the trial (e.g. the protocol or the logistics) they should report these to the Dept. Cardiology, Clinical Trials Unit, AUH and may decide not to accept the invitation to join. DSMC members should be independent and constructively critical of the ongoing trial, but also supportive of aims and methods of the trial.

Initial DSMC meeting

The first DSMC meeting will be held primo 2016 and hereafter then at least annually depending on the rate of accrual of data.

A DSMC statistical analysis plan, including a list of tables, will be provided prior to the initial meeting to familiarise the DSMC members with the intended content of the DSMC reports. The DSMC will review the statistical analysis plan at the first meeting and confirm the content of the report to be provided at subsequent meetings. Alterations to the report can be made at subsequent meetings to reflect the requirements of the DSMC.

DSMC composition

All members of the DSMC have previous expertise in trials.

The members of the DSMC for this trial are:

Chair: Henning Kelbæk, Independent interventional cardiologist

Hector Bueno, Independent interventional cardiologist

Morten Madsen, Independent Senior medical statistician

Jakob Hjort, Independent data manager

All DSMC members will maintain confidentiality, review available reports prior to meetings and contribute actively to developing the DSMC recommendations

The DSMC Chair will communicate the written recommendations of the DSMC to the Chair of the TSC, determine whether additional meetings are required, and maintain documentation of communication between the DSMC and the TSC.

The independent statistician supplying the DSMC, Morten Madsen, will produce the confidential interim reports for the DSMC and distribute these at least one week prior to each meeting. He will also participate in DSMC meetings, guiding the DSMC through the report, and contributing to DSMC discussions. The statistician will also prepare and distribute meeting minutes to DSMC members as password protected files within one week after each meeting.

The Chief Investigator, Prof Hans Erik Bøtker, and/or relevant members of the Condi 2 Clinical Trials Unit will be invited to join the open session of the DSMC meeting to provide an update on trial progress. The Condi 2 Clinical Trials Unit will provide a report on trial progress for discussion during the open session, for example recruitment, trial conduct etc.

See **Table 1** for full contact details of the DSMC and Independent Statistician.

DSMC membership and their obligations

Members of a DSMC are required to:

(1) Confirm their agreement to take on the responsibilities of membership as outlined in this Charter

(2) Agree to maintain the confidentiality of the data provided and the deliberations of the Committee

(3) Have no conflict of interest which will prejudice their role as a DSMC member

By agreeing to adopt this Charter, all members confirm the above.

Relationships

Figure 3 details the organisation of the Condi 2 trial and the reporting structure for the DSMC. The DSMC does not make decisions about the trial, but rather makes recommendations to the TSC.

As data from Condi 2 will be combined with data from ERIC-PPCI for the reporting of final results, it is intended that the TSC for both trials will be copied into the recommendations of each trial’s DSMC to the TSC. If a decision is reached to stop or modify either trial, this will be immediately communicated to the relevant committees of both trials.

Payments to DSMC members

Members will be reimbursed for standard class travel for DSMC duties.

The need for DSMC members to disclose information about any competing interests

All competing interests should be disclosed prior to agreeing this Charter. These are not restricted to financial matters – involvement in other trials or intellectual investment could be relevant. Although members may well be able to act objectively despite such connections, complete disclosure helps to enhance credibility (see Annex 1).

DSMC members should not use interim results to inform trading in pharmaceutical shares, and careful consideration should be given to trading in stock of companies with competing products.

DSMC organisation

DSMC meetings will be conducted to review interim data. It is intended that the DSMC will meet to review interim results after 3-6 months of recruitment and then at least annually depending on the rate of accrual of data.

The DSMC will decide on the type of meeting required (face-to-face or teleconference). The Condi 2 Clinical Trials Unit will make arrangements accordingly.

The format of the meetings will be as follows:

1. Open session: Introduction and any "open" parts of the report
2. Closed session: DSMC discussion of "closed" parts of the report

Attendance at open and closed sessions

In the open session all those attending the closed session are joined by the Chief Investigator and/or relevant members of the Condi 2 Clinical Trials Unit. External experts may also be invited to the DSMC meetings at the request of the DSMC members. No unblinded data will be discussed during this session.

In the closed session only members of the DSMC and others whom they specifically invite (e.g. Independent Statistician) will be present. The unblinded interim results will be discussed and the DSMC will decide on their recommendation to the Trial Steering Committee (TSC).

DSMC closed session discussions will remain confidential and will not be communicated to the Condi 2 Clinical Trials Unit.

Trial documentation and procedures to ensure confidentiality and proper communication

Data for preparation of the interim report will be accessed by Morten Madsen from the trial database using a unique username and password. In addition to this report, the DSMC will be provided with an update on trial progress from the Condi 2 Clinical Trials Unit. These documents will be circulated at least 1 week in advance of the DSMC meeting.

The interim reports on the ERIC-PPCI trial will be copied to the Condi 2 DSMC members for consideration alongside the unblinded Condi 2 report. Similarly the interim reports from the Condi 2 trial will be copied to the ERIC-PPCI DSMC members for consideration alongside the ERIC-PPCI report.

Intended content of material to be available

Open sessions: Accumulating information relating to recruitment and data quality (e.g. data return rates) will be presented.

Closed sessions: In addition to all the material available in the open session, the closed session material will include safety and efficacy data by treatment group. The unblinded ERIC-PPCI interim reports from previous meetings of the ERIC-PPCI DSMC will also be available at this session.

The unblinded interim reports will contain details of:

1. Patient recruitment
2. Demographic and baseline characteristics
3. Details of the RIC intervention
4. Treatment times for PPCI (onset to balloon, door to balloon, 1st ECG to balloon times)
5. IV medications given during PPCI
6. Mortality and other safety endpoints
7. Primary efficacy endpoint - cardiac death and HHF
8. Unexpected Adverse and Serious Adverse Events
9. Other endpoints identified by the DSMC

The DSMC members should store the papers safely after each meeting so they may check the next report against them. After the trial is reported, the DSMC members may destroy all interim reports.

DSMC recommendations and decision making

Possible recommendations could include:-

- No action needed, trial continues as planned
- Early stopping due, for example, to clear benefit or harm of a treatment, futility, or external evidence
- Stopping recruitment within a subgroup
- Extending recruitment (based on actual control arm response rates being different to predicted rather than on emerging differences) or extending follow-up
- Sanctioning and/or proposing protocol changes

Interim analyses and stopping rules

It is not expected that the trial will be stopped for reasons of efficacy or futility based on observed treatment differences. A robust demonstration of efficacy is required to dispel scepticism that this simple intervention may be effective. Similarly, if there is no effect of the intervention then stopping for futility will reduce the ability to demonstrate this. Therefore the primary reason to recommend stopping the trial will be for safety reasons.

For the primary outcome, the DSMC will consider stopping if there is evidence of a difference in rate between the RIC and control group achieving $p < 0.001$.

For the main safety endpoints (e.g. mortality) the DSMC will consider stopping if there is evidence of greater event rate in the RIC group achieving $p < 0.01$.

How decisions or recommendations will be reached within the DSMC

It is recommended that every effort should be made for the DSMC to reach a unanimous decision. If the DSMC cannot achieve this, a vote may be taken, and the majority position taken. Details of the vote should not be routinely included in the report to the TSC as these may inappropriately convey information about the state of the trial data.

It is important that the implications (e.g. ethical, statistical, practical, regulatory) for the trial be considered before any recommendation is made.

The role of the Chair is to summarise discussions and encourage consensus; it may be best for the Chair to give their own opinion last.

DSMC quorate for decision-making

Effort should be made for all members to attend. The meeting will be organised by the Condi 2 Clinical Trials Unit who will try to ensure that a date is chosen to enable this. Members who cannot attend in person at face-to-face meetings can attend by teleconference.

If, at short notice, any DSMC members cannot attend then the DSMC may still meet if the Chair and one other member is present and the absent member may provide input directly to the DSMC Chair. If the DSMC is considering recommending major action after such a meeting the DSMC Chair should talk with the absent member as soon after the meeting as possible to check they agree. If they do not, a further meeting should be arranged with the full DSMC.

1.1. Attendance at meetings

If a member does not attend a meeting, it should be ensured that the member is available for the next meeting. If a member does not attend a second meeting, they should be asked if they wish to remain part of the DSMC. If a member does not attend a third meeting, they should be replaced.

Reporting

The DSMC will report its recommendations in writing to the TSC. This will be a letter sent to the trial TSC chair within 3 weeks of the DSMC meeting. A copy of the letter should be sent to the chief investigator and Clinical Trials Unit for the Trial Master File and copied to TSC for the ERIC-PPCI trial. If the trial is to continue largely unchanged then it is often useful for the report from the DSMC to include a summary paragraph suitable for trial promotion purposes (see Annex 2).

If the decision is made to recommend stopping the study the DSMC Chair will contact the TSC Chair immediately following the meeting to discuss this decision and the reasons why. The TSC together with the sponsor of the trial will make the final decision as to whether to stop the trial.

Disagreement between the DSMC and the body to which it reports

If the DSMC has serious problems or concerns with the TSC's or Sponsor's decision a meeting of these groups should be held. The information to be shown would depend upon the action proposed

and the DSMC's concerns. Depending on the reason for the disagreement confidential data may have to be revealed to all those attending such a meeting.

Post trial

Publication of results

The TSC will provide draft reports of the primary results of the trial to the DSMC for their consideration prior to submission for publication. The DSMC may provide comments to the TSC on the draft reports, and give advice about data interpretation.

The information about the DSMC included in published trial reports

DSMC members will be named and their affiliations listed in the main report, unless they explicitly request otherwise. A brief summary of the timings and conclusions of DSMC meetings may be included if appropriate in the body of this paper.

Any constraints on DSMC members divulging information about their deliberations after the trial has been published

The DSMC may discuss issues from their involvement in the trial 6 months after the primary trial results have been published. If a DSMC needs to discuss their involvement any earlier, permission is required from the TSC.

Agreement

This Charter is agreed by the following member of the DSMC:

Name	Signature and date

Figures and appendices

Figure 1: Trial flow diagram CONDI 2

Figure 2: Trial flow diagram ERIC-PPCI

Figure 3: Flow of information for ERIC-PPCI trial DSMC meetings

Table 1: Contact details for DSMC members and independent statistician

Annex 1: Competing interest form

Annex 2: Suggested letter from DSMC to TSC where no recommendations are being made.

Annex 3: Confidentiality Agreement

Figure 1: Trial flow diagram Condi 2

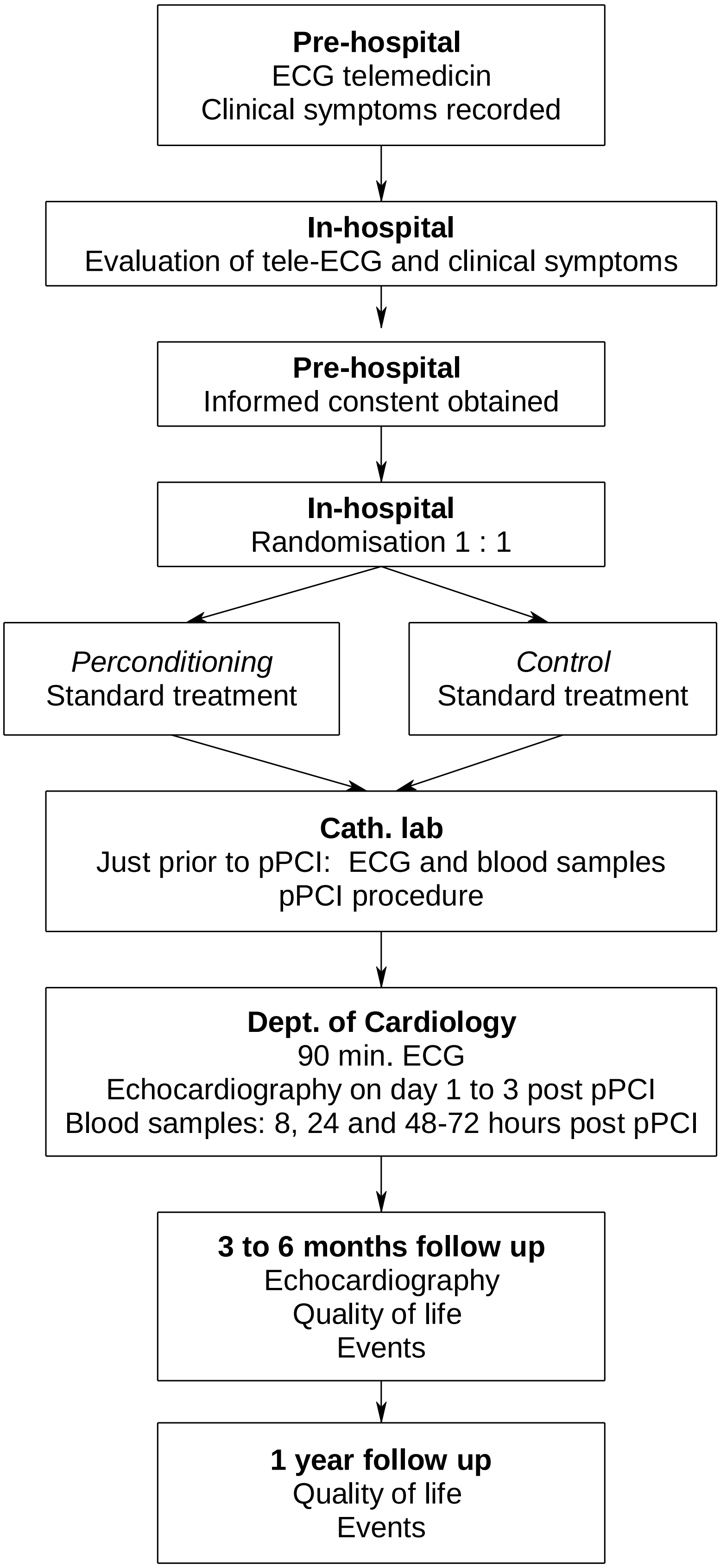

Figure 2: Trial flow diagram ERIC-PPCI

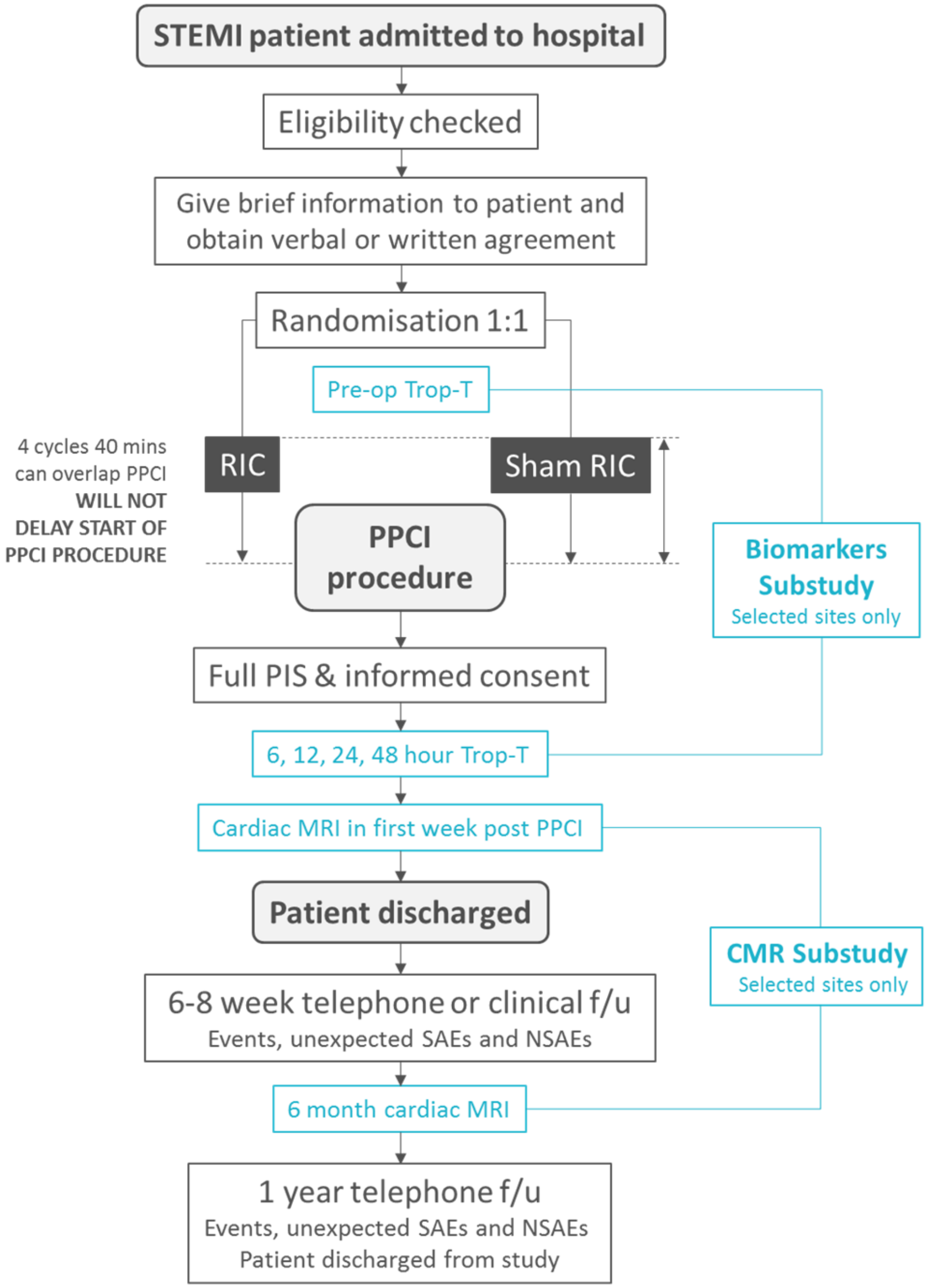

Figure 3: Flow of information for Condi 2 trial DSMC meetings

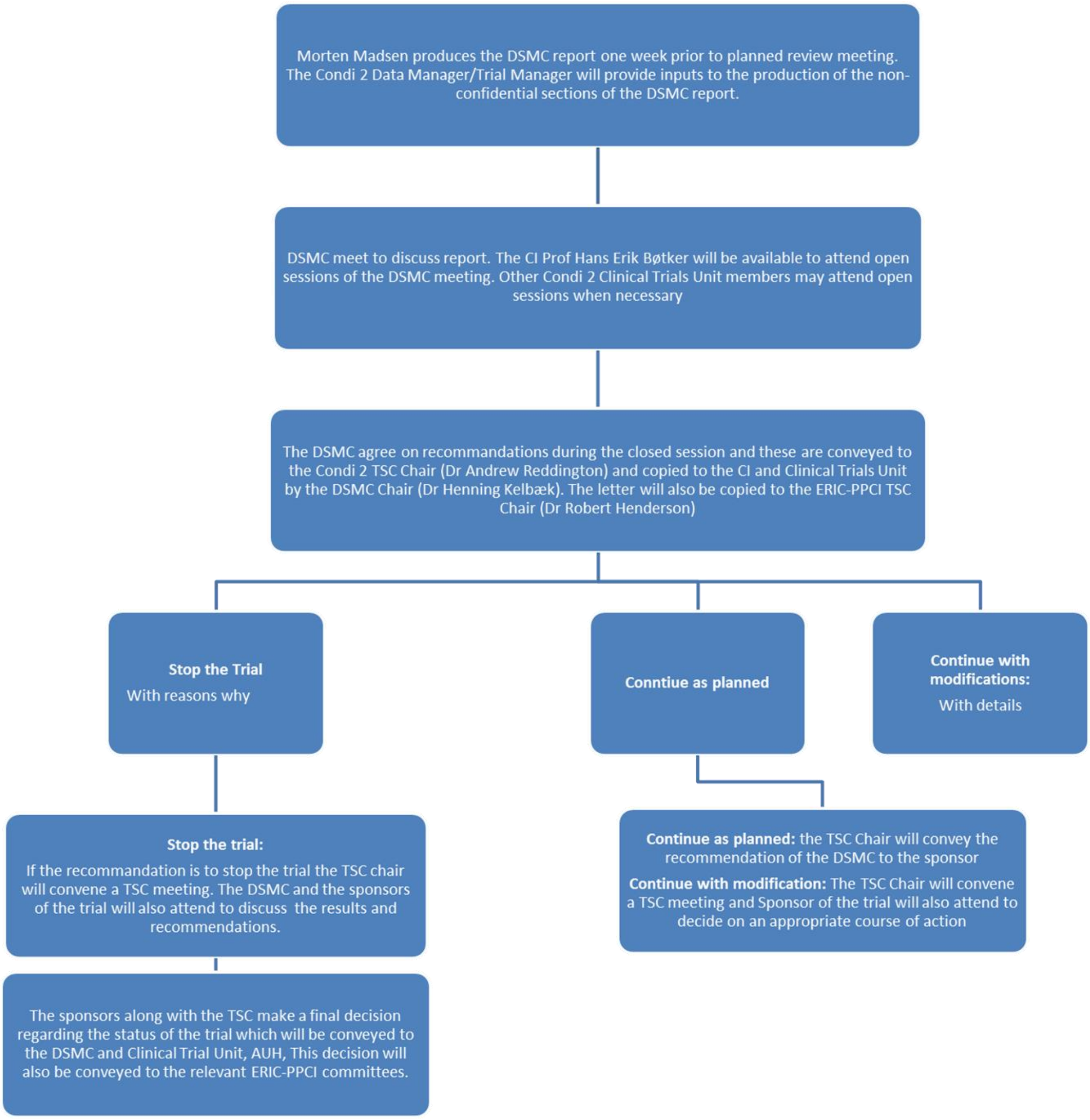

Table 1: Contact details of DSMC members and independent statistician

**Dr. Henning Kelbæk (DSMC Chair) (independent interventional cardiologist)**<br><br>Roskilde Hospital<br>Køgevej 7-13,<br>4000 Roskilde, Denmark<br>Email: hkelbaek@dadlnet.dk
**Dr Héctor Bueno (independent interventional cardiologist)**<br><br>Director. Multidisciplinary Translational Cardiovascular Research (MTCR) Group<br>Centro Nacional de Investigaciones Cardiovasculares (CNIC)<br>Scientific Director<br>Department of Cardiology<br>Email: hector.bueno@cnic.es
**Jakob Hjort (Independent data manager)**<br>Palle Juul-Jensens Boulevard 82<br>Bygning 2, stuen<br>8200 Aarhus N<br>Email: J.hjort@clin.au.dk
**Morten Madsen (Independent Statistician for Condi 2)**<br>Department of Epidemiology, Aarhus University Hospital,<br>Olof Palmes Allé 43-45<br>DK-8200 Aarhus N<br>8200 Aarhus<br>E-mail: Morten.madsen@clin.au.dk

Annex 1: Competing interests form

Annex 2: Suggested report from DSMC to TSC where no recommendations are being made

Annex 3: Agreement on the non-disclosure of confidential information for the members of the data safety and monitoring committee (DSMC)

ERIC-PPCI

Effect of Remote Ischaemic Conditioning on clinical outcomes in ST segment elevation myocardial infarction patients undergoing Primary Percutaneous Coronary Intervention

Data and Safety Monitoring Committee (DSMC) Charter

for the

Effect of Remote Ischaemic Conditioning on clinical outcomes in ST-segment elevation myocardial infarction patients undergoing Primary Percutaneous Coronary Intervention (ERIC-PPCI) trial

Introduction

**Title**	Effect of Remote Ischaemic Conditioning on clinical outcomes in ST-segment elevation myocardial infarction patients undergoing Primary Percutaneous Coronary Intervention
**Acronym**	ERIC-PPCI
**Sponsor**	University College London
**NCT**	NCT02342522

ERIC-PPCI is a randomised controlled trial to determine whether remote ischaemic conditioning (RIC) improves clinical outcomes after in patients with ST-segment elevation myocardial infarction undergoing Primary Percutaneous Coronary Intervention (PPCI). Prof Derek Hausenloy (University College London, UK) is the Chief Investigator and the trial is managed by the Clinical Trials Unit at the London School of Hygiene & Tropical Medicine (LSHTM).

The intervention is non-pharmacological. Patients are randomised to one of two groups: sham RIC (control) or RIC. The RIC treatment is applied on arrival at the hospital. Automated CellAegis autoRIC™ cuff devices will be used to deliver the RIC and sham RIC protocols. The active RIC treatment consists of four 5-minute inflations of the autoRIC™ cuff on the upper arm to 200mmHg, separated by 5-minute periods when the cuff will be deflated. For patients presenting with a systolic blood pressure (SBP) ≥175mmHg, a manual blood pressure cuff will be used and inflated to 25 mmHg above systolic blood pressure. The sham RIC consists of a four 5-minute simulated inflations of the autoRIC™ cuff, separated by 5-minute periods when the cuff will be deflated.

The reporting of final results from the ERIC-PPCI trial will be conducted in collaboration with Prof Hans Erik Bøtker (Aarhus University, Denmark), who is Chief Investigator of the CONDI2 trial, in order to maximise power to assess the impact of RIPC on clinical endpoints. In total 4300 STEMI patients will be recruited: 2000 STEMI patients will be randomised to ERIC-PPCI (Figure 1) and 2300 STEMI patients will be randomised to CONDI 2 (Figure 2).

Primary endpoint

The primary endpoint is occurrence of cardiac death and hospitalisation for heart failure (HHF) in STEMI patients 12 months after PPCI.

Major secondary endpoints

9. Cardiac death and HHF at 30 days post PPCI.
10. All-cause death at 30 days and 12 months.
11. Repeat coronary revascularisation at 30 days and 12 months.
12. Reinfarction at 30 days and 12 months.
13. Stroke at 30 days and 12 months.
14. TIMI flow post PPCI
15. Quality of life at 6-8 weeks and 12 months (EuroQol EQ-5D-5L)

Clinical outcome data will be collected at the telephone or outpatient follow-up at 6-8 weeks and one year post-PPCI.

Outline of scope of charter

The purpose of this document is to describe the roles and responsibilities of the independent DSMC for the ERIC-PPCI trial, including the timing of meetings, methods of providing information to and from the DSMC, frequency and format of meetings, statistical issues and relationships with other committees.

The following documents were reviewed in preparation of this charter:

- ERIC-PPCI trial protocol version 2, issued 24th Feb 2015
- CONDI-2 trial protocol version 10.4, issued October 2015
- Case report form (CRF) version 3, issued February 2016
- London School of Hygiene and Tropical Medicine (LSHTM) Template charter for DMCs, issued 7th July 2015
- DAMOCLES study group proposed charter for data monitoring committees data monitoring committees[2]

Roles and responsibilities

The DSMC role is to examine the data accumulated during progress of the ERIC-PPCI trial and ensure that the benefit/risk ratio remains acceptable for participating patients. It is the only committee which will have access to data broken down by treatment during the trial and on this basis, the primary responsibility of the DSMC is to review interim analyses of outcome data and to recommend

[2] DAMOCLES Study Group (2005) A proposed charter for clinical trial data monitoring committees: helping them to do their job well. Lancet. 365: 711-22.

to the Trial Steering Committee (TSC) whether the trial needs to be changed or terminated based on these analyses.

More specifically, the duties of the DSMC will include:

- monitoring evidence for treatment differences in the primary and secondary endpoints
- monitoring evidence for treatment harm (e.g. SAEs, deaths)
- assessing the impact and relevance of external evidence, particularly DSMC reports and recommendations from the ongoing CONDI 2 trial
- deciding whether to recommend that the trial continues to recruit participants or whether recruitment should be terminated either for everyone or for some treatment groups and/or some participant subgroups
- assessing data quality, including completeness
- reviewing recruitment figures and monitor losses to follow-up
- monitoring compliance with the protocol by participants and investigators
- monitoring continuing appropriateness of patient information
- monitoring compliance with previous DSMC recommendations
- considering the ethical implications of any recommendations made by the DSMC

DSMC input early in the trial

All potential DSMC members should have sight of the trial protocol before finalising their agreement to join the committee. Before recruitment begins the trial will have undergone review by the sponsor, scrutiny by other trial committees and a research ethics committee. Therefore, if a potential DSMC member has major reservations about the trial (e.g. the protocol or the logistics) they should report these to the ERIC-PPCI Clinical Trials Unit and may decide not to accept the invitation to join. DSMC members should be independent and constructively critical of the ongoing trial, but also supportive of aims and methods of the trial.

Initial DSMC meeting

The DSMC will hold an initial meeting as early in the course of the trial as is reasonable practicable, to discuss the protocol, the trial, any analysis plan, future meetings, and to have the opportunity to clarify any aspects with the Trial Steering Committee.

A DSMC statistical analysis plan, including a list of tables, will be provided prior to the initial meeting to familiarise the DSMC members with the intended content of the DSMC reports. The DSMC will

review the statistical analysis plan at the first meeting and confirm the content of the report to be provided at subsequent meetings. Alterations to the report can be made at subsequent meetings to reflect the requirements of the DSMC.

DSMC composition

All members of the DSMC have previous expertise in trials.

The members of the DSMC for this trial are:

Chair: Prof Colin Berry - Chair (Professor of Cardiology and Imaging)

Prof Tom Meade (Emeritus Professor of Epidemiology)

Andrew Copas (Reader in Statistics)

All DSMC members will maintain confidentiality, review available reports prior to meetings and contribute actively to developing the DSMC recommendations

The DSMC Chair will communicate the written recommendations of the DSMC to the Chair of the TSC, determine whether additional meetings are required, and maintain documentation of communication between the DSMC and the TSC.

The independent statistician supplying the DSMC, Jennifer Nicholas, will produce the confidential interim reports for the DSMC and distribute these at least one week prior to each meeting. She will also participate in DSMC meetings, guiding the DSMC through the report, and contributing to DSMC discussions. The statistician will also prepare and distribute meeting minutes to DSMC members as password protected files within one week after each meeting.

The Chief Investigator, Prof Derek Hausenloy, and/or relevant members of the ERIC-PPCI Clinical Trials Unit will be invited to join the open session of the DSMC meeting to provide an update on trial progress. The ERIC-PPCI Clinical Trials Unit will provide a report on trial progress for discussion during the open session, for example recruitment, trial conduct etc.

See **Table 1** for full contact details of the DSMC and Independent Statistician.

DSMC membership and their obligations

Members of a DSMC are required to:

(4) Confirm their agreement to take on the responsibilities of membership as outlined in this Charter

(5) Agree to maintain the confidentiality of the data provided and the deliberations of the Committee

(6) Have no conflict of interest which will prejudice their role as a DSMC member

By agreeing to adopt this Charter, all members confirm the above.

Relationships

Figure 3 details the organisation of the ERICC-PPCI trial and the reporting structure for the DSMC. The DSMC does not make decisions about the trial, but rather makes recommendations to the TSC.

The DSMC for ERIC-PPCI and CONDI 2 will act as independent bodies. Therefore, the ERIC-PPCI DSMC will make their recommendations to the ERIC-PPCI TSC. This recommendation letter will be copied to the CONDI 2 TSC for their information. A reciprocal arrangement is place so the chair of the ERIC-PPCI TSC receives a copy of recommendation letters of the CONDI 2 DSMC.

The ERIC-PPCI DSMC members will be provided with copies of the CONDI 2 DSMC reports.

Payments to DSMC members

Members will be reimbursed for standard class travel for DSMC duties.

The need for DSMC members to disclose information about any competing interests

All competing interests should be disclosed prior to agreeing this Charter. These are not restricted to financial matters – involvement in other trials or intellectual investment could be relevant. Although members may well be able to act objectively despite such connections, complete disclosure helps to enhance credibility (see Annex 1).

DSMC members should not use interim results to inform trading in pharmaceutical shares, and careful consideration should be given to trading in stock of companies with competing products.

DSMC organisation

DSMC meetings will be conducted to review interim data. It is intended that the DSMC will meet to review interim results after 3-6 months of recruitment and then at least annually depending on the rate of accrual of data.

The DSMC will decide on the type of meeting required (face-to-face or teleconference). The ERIC-PPCI Clinical Trials Unit will make arrangements accordingly.

The format of the meetings will be as follows:

3. Open session: Introduction and any “open” parts of the report
4. Closed session: DSMC discussion of “closed” parts of the report

Attendance at open and closed sessions

In the open session all those attending the closed session are joined by the Chief Investigator and/or relevant members of the ERIC-PPCI Clinical Trials Unit. External experts may also be invited to the DSMC meetings at the request of the DSMC members. No unblinded data will be discussed during this session.

In the closed session only members of the DSMC and others whom they specifically invite (e.g. Independent Statistician) will be present. The unblinded interim results will be discussed and the DSMC will decide on their recommendation to the Trial Steering Committee (TSC).

DSMC closed session discussions will remain confidential and will not be communicated to the ERIC-PPCI Clinical Trials Unit.

Trial documentation and procedures to ensure confidentiality and proper communication

Data for preparation of the interim report will be accessed by Jennifer Nicholas from the trial database using a unique username and password. In addition to this report, the DSMC will be provided with an update on trial progress from the ERIC-PPCI Clinical Trials Unit. These documents will be circulated at least 1 week in advance of the DSMC meeting.

The interim reports on the CONDI 2 trial will be copied to the ERIC-PPCI DSMC members for consideration alongside the unblinded ERIC-PPCI report. Similarly, the interim reports from the ERIC-PPCI trial will be copied to the CONDI 2 DSMC members for consideration alongside the CONDI 2 report.

Intended content of material to be available

Open sessions: Accumulating information relating to recruitment and data quality (e.g. data return rates) will be presented.

Closed sessions: In addition to all the material available in the open session, the closed session material will include safety and efficacy data by treatment group. The unblinded CONDI 2 interim reports from previous meetings of the CONDI 2 DSMC will also be available at this session.

The unblinded interim reports will contain details of:

1. Patient recruitment

2. Demographic and baseline characteristics

3. Details of the RIC intervention

4. Treatment times for PPCI (onset to balloon, door to balloon, 1st ECG to balloon times)

5. IV medications given during PPCI

6. Mortality and other safety endpoints

7. Primary efficacy endpoint - cardiac death and HHF

8. Unexpected Adverse and Serious Adverse Events

9. Other endpoints identified by the DSMC

The DSMC members should store the papers safely after each meeting so they may check the next report against them. After the trial is reported, the DSMC members may destroy all interim reports.

DSMC recommendations and decision making

Possible recommendations could include:-

- No action needed, trial continues as planned
- Early stopping due, for example, to clear benefit or harm of a treatment, futility, or external evidence
- Stopping recruitment within a subgroup
- Extending recruitment (based on actual control arm response rates being different to predicted rather than on emerging differences) or extending follow-up
- Sanctioning and/or proposing protocol changes

Interim analyses and stopping rules

It is not expected that the trial will be stopped for reasons of efficacy or futility based on observed treatment differences. A robust demonstration of efficacy is required to dispel scepticism that this simple intervention may be effective. Similarly, if there is no effect of the intervention then stopping

for futility will reduce the ability to demonstrate this. Therefore, the primary reason to recommend stopping the trial will be for safety reasons:

- For the primary outcome, the DSMC will consider stopping if there is evidence of a difference in rate between the RIC and control group
- For the main safety endpoints (e.g. mortality) the DSMC will consider stopping if there is evidence of greater event rate in the RIC group.

As per the guidance of the DAMOCLES study group[3], the DSMC will consider the strength of any formal statistical comparison alongside the internal consistency of results, consistency with external evidence and ability of the results to influence clinical practice.

How decisions or recommendations will be reached within the DSMC

It is recommended that every effort should be made for the DSMC to reach a unanimous decision. If the DSMC cannot achieve this, a vote may be taken, and the majority position taken. Details of the vote should not be routinely included in the report to the TSC as these may inappropriately convey information about the state of the trial data.

It is important that the implications (e.g. ethical, statistical, practical, regulatory) for the trial be considered before any recommendation is made.

The role of the Chair is to summarise discussions and encourage consensus; it may be best for the Chair to give their own opinion last.

DSMC quorate for decision-making

Effort should be made for all members to attend. The meeting will be organised by the ERIC-PPCI Clinical Trials Unit who will try to ensure that a date is chosen to enable this. Members who cannot attend in person at face-to-face meetings can attend by teleconference.

If, at short notice, any DSMC members cannot attend then the DSMC may still meet if the Chair and one other member is present and the absent member may provide input directly to the DSMC Chair. If the DSMC is considering recommending major action after such a meeting the DSMC Chair should talk with the absent member as soon after the meeting as possible to check they agree. If they do not, a further meeting should be arranged with the full DSMC.

---

[3] DAMOCLES Study Group (2005) A proposed charter for clinical trial data monitoring committees: helping them to do their job well. Lancet. 365: 711-22.

1.2. Attendance at meetings

If a member does not attend a meeting, it should be ensured that the member is available for the next meeting. If a member does not attend a second meeting, they should be asked if they wish to remain part of the DSMC. If a member does not attend a third meeting, they should be replaced.

Reporting

The DSMC will report its recommendations in writing to the TSC. This will be a letter sent to the trial TSC chair within 3 weeks of the DSMC meeting. A copy of the letter should be sent to the chief investigator and Clinical Trials Unit for the Trial Master File and copied to the trial TSC chair of the CONDI 2 trial. If the trial is to continue largely unchanged then it is often useful for the report from the DSMC to include a summary paragraph suitable for trial promotion purposes (see Annex 2).

If the decision is made to recommend stopping the study the DSMC Chair will contact the TSC Chair immediately following the meeting to discuss this decision and the reasons why. The TSC together with the sponsor of the trial will make the final decision as to whether to stop the trial.

Disagreement between the DSMC and the body to which it reports

If the DSMC has serious problems or concerns with the TSC's or Sponsor's decision a meeting of these groups should be held. The information to be shown would depend upon the action proposed and the DSMC's concerns. Depending on the reason for the disagreement confidential data may have to be revealed to all those attending such a meeting.

Post trial

Publication of results

The TSC will provide draft reports of the primary results of the trial to the DSMC for their consideration prior to submission for publication. The DSMC may provide comments to the TSC on the draft reports, and give advice about data interpretation.

The information about the DSMC included in published trial reports

DSMC members will be named and their affiliations listed in the main report, unless they explicitly request otherwise. A brief summary of the timings and conclusions of DSMC meetings may be included if appropriate in the body of this paper.

Any constraints on DSMC members divulging information about their deliberations after the trial has been published

The DSMC may discuss issues from their involvement in the trial 6 months after the primary trial results have been published. If a DSMC needs to discuss their involvement any earlier, permission is required from the TSC.

Agreement

This Charter is agreed by the following member of the DSMC:

Name	Signature and date

Figures and appendices

Figure 1: Trial flow diagram ERIC-PPCI

Figure 2: Trial flow diagram CONDI 2

Figure 3: Flow of information for ERIC-PPCI trial DSMC meetings

Table 1: Contact details for DSMC members and independent statistician

Annex 1: Competing interest form

Annex 2: Suggested letter from DSMC to TSC where no recommendations are being made.

Annex 3: Confidentiality Agreement

Figure 1: Trial flow diagram ERIC-PPCI

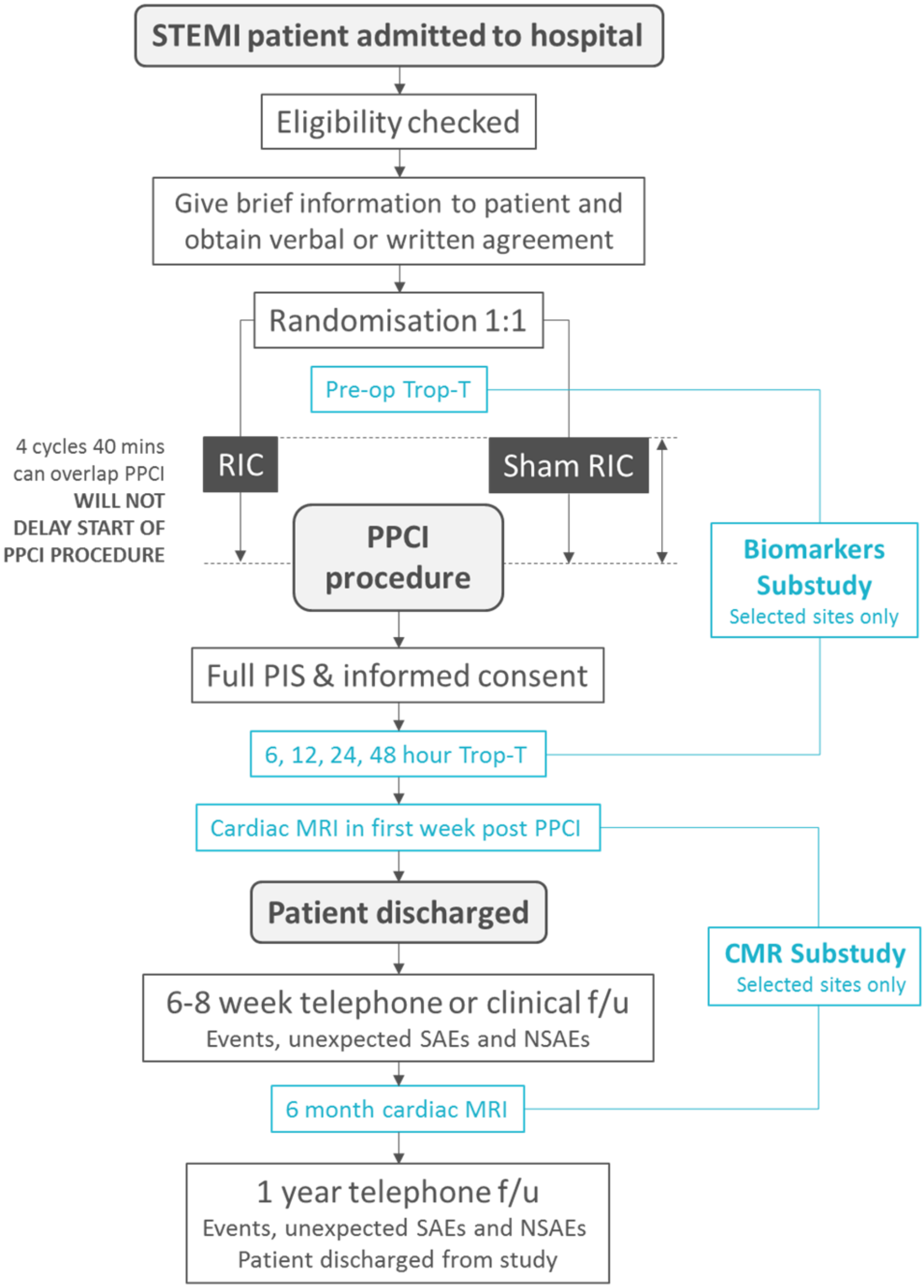

Figure 2: Trial flow diagram CONDI 2

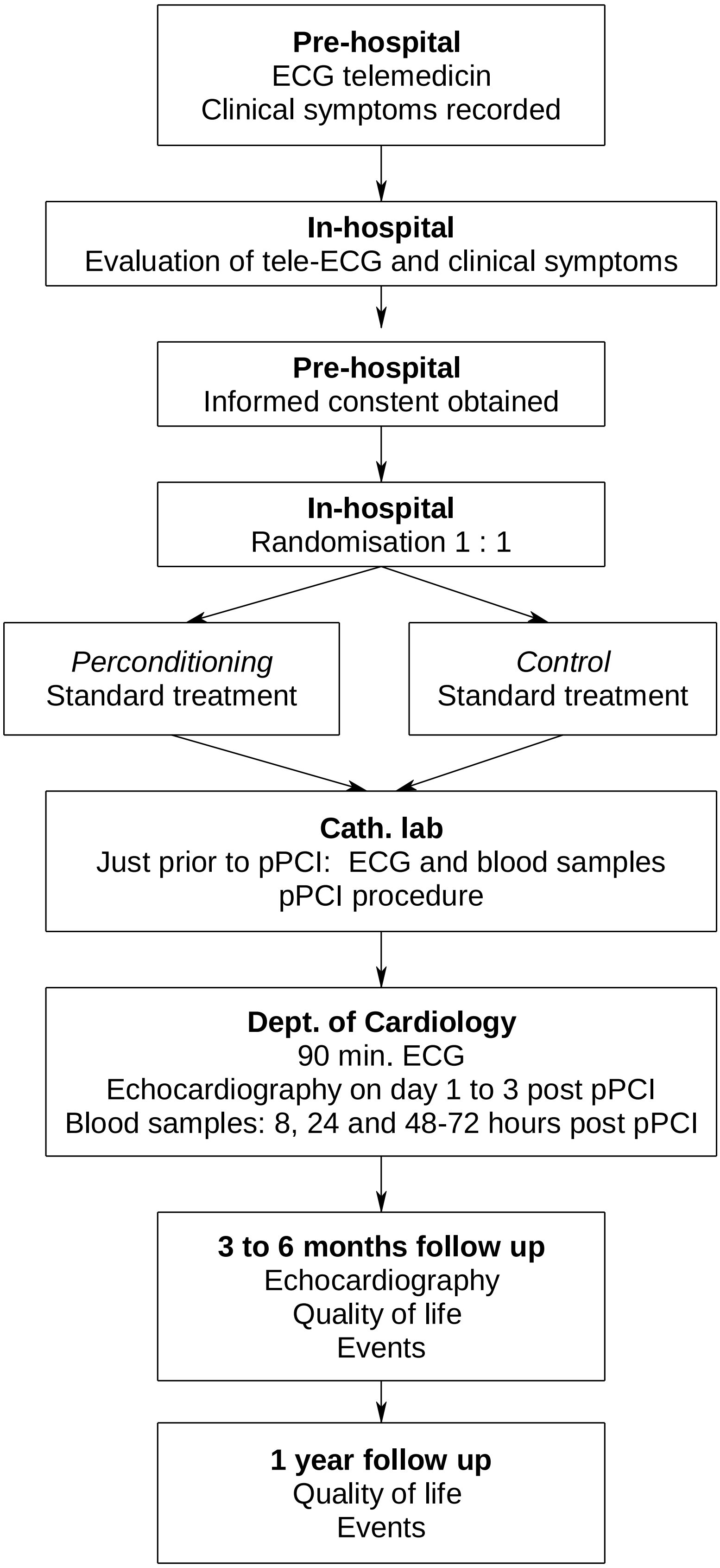

Figure 3: Flow of information for ERIC-PPCI trial DSMC meetings

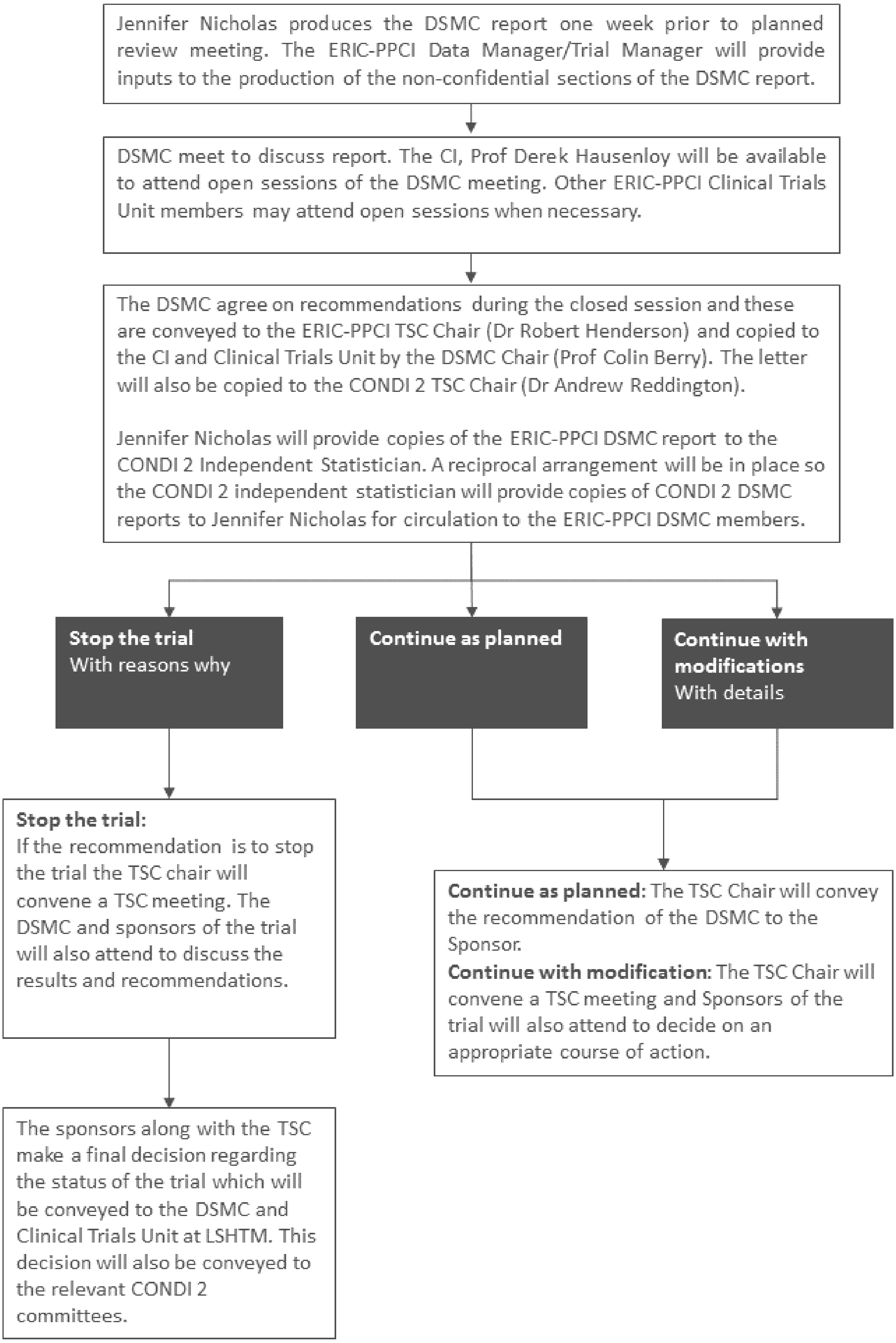
Jennifer Nicholas produces the DSMC report one week prior to planned review meeting. The ERIC-PPCI Data Manager/Trial Manager will provide inputs to the production of the non-confidential sections of the DSMC report.
DSMC meet to discuss report. The CI, Prof Derek Hausenloy will be available to attend open sessions of the DSMC meeting. Other ERIC-PPCI Clinical Trials Unit members may attend open sessions when necessary.
The DSMC agree on recommendations during the closed session and these are conveyed to the ERIC-PPCI TSC Chair (Dr Robert Henderson) and copied to the CI and Clinical Trials Unit by the DSMC Chair (Prof Colin Berry). The letter will also be copied to the CONDI 2 TSC Chair (Dr Andrew Reddington).
Jennifer Nicholas will provide copies of the ERIC-PPCI DSMC report to the CONDI 2 Independent Statistician. A reciprocal arrangement will be in place so the CONDI 2 independent statistician will provide copies of CONDI 2 DSMC reports to Jennifer Nicholas for circulation to the ERIC-PPCI DSMC members.
Stop the trial
With reasons why
Continue as planned
Continue with modifications
With details
Stop the trial:
If the recommendation is to stop the trial the TSC chair will convene a TSC meeting. The DSMC and sponsors of the trial will also attend to discuss the results and recommendations.
Continue as planned: The TSC Chair will convey the recommendation of the DSMC to the Sponsor.
Continue with modification: The TSC Chair will convene a TSC meeting and Sponsors of the trial will also attend to decide on an appropriate course of action.
The sponsors along with the TSC make a final decision regarding the status of the trial which will be conveyed to the DSMC and Clinical Trials Unit at LSHTM. This decision will also be conveyed to the relevant CONDI 2 committees.

Table 1: Contact details of DSMC members and independent statistician

**Professor Colin Berry (DSMC Chair)**<br>BHF Glasgow Cardiovascular Research Centre<br>University of Glasgow, G12 8TA<br>Email: Colin.Berry@glasgow.ac.uk
**Professor Tom Meade**<br>Dept. Non-Communicable Disease Epidemiology<br>London School of Hygiene & Tropical Medicine<br>Keppel Street<br>London<br>WC1E 7HT<br>Tel: 020 7927 2182<br>Email: Tom.Meade@lshtm.ac.uk
**Dr Andrew Copas**<br>UCL Research Department of Infection and Population Health<br>The Mortimer Market Centre<br>Capper St<br>London<br>WC1E 6JB<br>Tel: 020 3108 2062<br>Email: a.copas@ucl.ac.uk
**Dr Jennifer Nicholas (Independent Statistician for ERIC-PPCI)**<br>Lecturer in Medical Statistics<br>Department of Medical Statistics,<br>London School of Hygiene and Tropical Medicine,<br>Keppel Street,<br>London<br>WC1E 7HT<br>Tel: 020 958 8119<br>E-mail: jennifer.nicholas@lshtm.ac.uk

Annex 1: Competing interests form

Annex 2: Suggested report from DSMC to TSC where no recommendations are being made

Annex 3: Agreement on the non-disclosure of confidential information for the members of the data safety and monitoring committee (DSMC)

Data handling- ERIC-PPCI

ERIC-PPCI used a bespoke database built by Sealed Envelope, Inc (London, UK) and hosted by Rackspace. The database was compliant with NHS and UK data handling requirements. Patient data was entered directly to the database by hospital research staff using a password protected online portal. Data was reviewed and queried by LSHTM Staff to ensure accuracy. Patient identifiable information was stored only with patient consent or with the approval of the UK Confidentiality Advisory Group.

Data handling- CONDI-2

Condi-2 used the 24-7 accessible web-based clinical trial support system TrialPartner (Aarhus, DK). The database is compliant with all Danish/EU data handling requirements. Personal logons permitted patient data to be entered directly to the database by hospital research staff at the recruiting sites. Data were reviewed and queried by the staff at the coordination centre at Aarhus University Hospital to ensure completeness and accuracy. Patient identifiable information was stored only with patient consent.

References

1. Hicks KA, Tcheng JE, Bozkurt B, et al. 2014 ACC/AHA Key Data Elements and Definitions for Cardiovascular Endpoint Events in Clinical Trials: A Report of the American College of Cardiology/American Heart Association Task Force on Clinical Data Standards (Writing Committee to Develop Cardiovascular Endpoints Data Standards). Journal of the American College of Cardiology 2015;66:403-69.
2. Thygesen K, Alpert JS, Jaffe AS, et al. Third universal definition of myocardial infarction. European heart journal 2012;33:2551-67.
