# Supplementary material for: Effect of remote ischaemic conditioning on clinical outcomes in patients with acute myocardial infarction (CONDI-2/ERIC-PPCI): a single-blind randomised controlled trial
Source: Lancet. 2019 Oct 19;394(10207):1415–24. doi: 10.1016/S0140-6736(19)32039-2 (PMC6891239; doi:10.1016/S0140-6736(19)32039-2)
Supplement: Supplementary appendix 2 [file mmc2.pdf]

THE LANCET

Supplementary appendix

This appendix formed part of the original submission and has been peer reviewed. We post it as supplied by the authors.

Supplement to: Hausenloy DJ, Kharbanda RK, Møller UK, et al. Effect of remote ischaemic conditioning on clinical outcomes in patients with acute myocardial infarction (CONDI-2/ERIC-PPCI): a single-blind randomised controlled trial. *Lancet* 2019; published online September 6. http://dx.doi.org/10.1016/S0140-6736(19)32039-2.

CONDI-2/ERIC-PPCI Protocols and Statistical Analysis Plans

Table of Contents

	Page
ERIC-PPCI protocol Version 1.0	2
ERIC-PPCI protocol Version 5.0	37
Summary of changes to ERIC-PPCI protocol	79
CONDI-2 protocol Version 4.0	81
CONDI-2 protocol Version 10.0	98
Summary of changes to CONDI-2 protocol	120
COMBINED CONDI-2/ERIC-PPCI SAP Version 1	121
COMBINED CONDI-2/ERIC-PPCI SAP Version 3	133
Summary of changes to CONDI-2/ERIC-PPCI SAP	146

ERIC-PPCI

Effect of Remote Ischaemic Conditioning on clinical outcomes in ST segment elevation myocardial infarction patients undergoing Primary Percutaneous Coronary Intervention

Trial Protocol Version 1

Sponsored by UCL

Funded by the British Heart Foundation

Protocol Number: Version 1.0

Title: Effect of Remote Ischaemic Conditioning on clinical outcomes in ST-segment elevation myocardial infarction patients undergoing Primary Percutaneous Coronary Intervention (**ERIC-PPCI**): A multicentre randomised controlled clinical trial

Chief investigator: Prof Derek J Hausenloy

The Hatter Cardiovascular Institute,

University College London,

67 Chenies Mews, London WC1E 6HX, UK.

Tel: 0203 447 9591

Email: d.hausenloy@ucl.ac.uk

Co-investigators: Prof Derek M Yellon, The Hatter Cardiovascular Institute, UCL, London

Prof Simon Redwood, King College London, London

Dr Rajesh Kharbanda, John Radcliffe Hospital, Oxford

Mr Tim Clayton, London School of Hygiene and Tropical Medicine (LSHTM), London

Location: UK Multicentre

ERIC-PPCI is a collaboration with the **Effect of RIC on Clinical Outcomes in STEMI Patients Undergoing pPCI (CONDI-2)** trial based in Denmark, Serbia and Spain.

Local sites and investigators: Listed on the ERIC-PPCI website: http://ericppci.lshtm.ac.uk/

Sponsor: University College London

Medical contact: Prof Derek J Hausenloy

Clinical Trials Unit: London School of Hygiene and Tropical Medicine

Table of Contents

1. Trial summary 6
   1.1. Protocol summary 6
   1.2. Trial flowchart 8

2. Introduction 9
   2.1. Background 9
   2.2. Scientific rationale 9
   2.3. Research collaboration with Denmark 10

3. Hypothesis 10

4. Endpoints 10
   4.1. Primary endpoint 10
   4.2. Secondary endpoints 10
   4.3. Endpoint Validation 11

5. Power calculations and sample size determination 12
   5.1. Primary combined clinical endpoint 12
   5.2. Biomarker substudy to assess the effect of RIC on MI size (48 hr AUC) 12
   5.3. CMR substudy to assess the effect of RIC on MI size at 6 months 12

6. Selection of patients 13
   6.1. Inclusion criteria 13
   6.2. Exclusion criteria 13

7. Ethical considerations 13
   7.1. Consent 13
   7.2. Declaration of Helsinki and UCL Good Clinical Practice 14
   7.3. Ethical committee review 14

8. Randomisation 14
   8.1. Randomisation procedure 14
   8.2. Access to randomisation site 14

9. Blinding 14
   9.1. Unblinded trial staff 14
   9.2. Blinded trial staff 14
   9.3. Emergency unblinding 14

10. Trial treatment 15
   10.1. Remote ischaemic conditioning (RIC) 15
   10.2. Sham RIC 15
   10.3. Duration of treatment 15

11. Data collection and follow up 16
   11.1. Trial procedures table 16
   11.2. Data collection 17
   11.3. Trial procedures 18
   11.4. Compliance and loss to follow up 19

12. Biomarker substudy 19
   12.1. Procedure 19
   12.2. Analysis methods 19

13. CMR substudy 19
   13.1. CMR substudy endpoints 19
   13.2. Exclusion criteria for CMR substudy 20

14. Safety Reporting 20
14.1. Definition 20
14.2. Expected adverse events (recognised to be caused by the RIC stimulus) 20
14.3. Expected serious adverse events related to usual clinical care 21
14.4. Unexpected Serious Adverse Events 21
14.5. Unexpected Non-Serious Adverse Events 21
14.6. Reporting unexpected adverse events 22
15. Withdrawal of patients 22
15.1. Criteria for withdrawal from the trial 22
15.2. Follow up of patients withdrawing from the trial 22
15.3. Reporting withdrawal of patients 22
16. Statistics 22
16.1. Trial statistician 22
16.2. Statistical analysis 23
16.3. Planned subgroup analysis 23
16.4. Procedure to account for missing or spurious data 23
17. Data handling and record keeping 23
18. Insurance 23
19. Publications policy 23
20. Expected value of the results 24
21. Trial organisation 24
21.1. Trial Steering Committee (TSC) 24
21.2. Project Management Group (PMG) 25
21.3. Data Safety Monitoring Committee (DSMC) 25
21.4. Endpoint Validation Committee (EVC) 25
22. References 26
23. Appendix 1 – CMR protocol 29
24. Appendix 2 – Glossary 30
25. Appendix 3 – EuroQol EQ-5D-5L 34

1. Trial summary

1.1. Protocol summary

Title	**E**ffect of **R**emote **I**schaemic **C**onditioning on clinical outcomes in ST-segment elevation myocardial infarction patients undergoing **P**rimary **P**ercutaneous **C**oronary **I**ntervention (ERIC-PPCI): A multicentre randomised controlled clinical trial.
Sponsor	University College London.
Medical condition or disease under investigation	Acute Myocardial Infarction.
Purpose of clinical trial	To determine whether remote ischaemic conditioning (RIC) improves clinical outcomes in STEMI patients undergoing PPCI.
Trial design	Randomised double blind placebo controlled trial.
Primary objectives	To determine the effect of RIC on cardiac death and hospitalisation for heart failure (HHF) at 12 months in STEMI patients undergoing PPCI.
Secondary objectives	To determine the effects of RIC on:<br>• Rates of cardiac death and HHF at 30 days.<br>• Rates of all-cause death, repeat coronary revascularisation, reinfarction, stroke at 30 days and 12 months.<br>• TIMI flow post-PPCI<br>• Quality of life at 6-8 weeks and one year (EQ-5D-5L)<br>• Biomarkers substudy: (400 patients at selected sites) 48 hour area under the curve high-sensitivity troponin T and CK-MB.<br>• CMR Substudy: (250 patients at selected sites) Myocardial infarct size expressed as a percentage of the LV mass by Cardiac MRI at 6 months.
Sample size	**ERIC-PPCI trial** (UK) 2000 patients undergoing PPCI following STEMI.<br>The trial is a collaboration with the CONDI-2 trial (Denmark, Serbia, Spain) which will recruit 2300 patients (NCT01857414).<br>In total the sample size is 4300 patients.

Inclusion criteria	1. Onset of STEMI symptoms within 12 hours, lasting for more than 30 minutes<br>2. Age >18 years<br>3. Suspected STEMI (ST-elevation at the J-point in two contiguous leads with the cut-off points: ≥0.2 millivolt (mV) in men or ≥0.15 mV in women in leads V2-V3 and/or ≥0.1 mV in other leads.)<br>4. Eligibility for coronary angiography with follow on PPCI if indicated
Exclusion criteria	1. Previous coronary artery bypass graft surgery<br>2. Myocardial infarction (MI) within the previous 30 days<br>3. Treatment with thrombolysis within the previous 30 days<br>4. Left bundle branch block<br>5. Patients treated with therapeutic hypothermia<br>6. Conditions precluding use of RIC (paresis of upper limb, use of an a-v shunt)<br>7. Life expectancy of less than 1 year due to non-cardiac pathology
Trial treatment	The intervention will be applied at a single time point on arrival at the PCI centre, starting prior to the PPCI procedure. The trial treatment may overlap with the start of the PPCI procedure and will not delay PPCI.<br><br>Active treatment (RIC) will consist of four 5 minute inflations of an automated autoRIC™ cuff on the upper arm to 200mmHg. For patients presenting with a systolic blood pressure (SBP)≥175mmHg, a manual blood pressure cuff will be used in place of the autoRIC™ cuff. This will be inflated to 25 mmHg above systolic blood pressure. The inflations will be separated by 5 minute periods when the blood pressure cuff will be deflated.<br><br>Control treatment (sham RIC) will consist of placing an identical looking autoRIC™ cuff on the upper arm which is designed to deliver four 5 minute simulated inflations. The simulated inflations will be separated by 5 minute periods when the blood pressure cuff will remain uninflated.

A glossary of terms and abbreviations used in this protocol is included as Appendix 2.

1.2. Trial flowchart

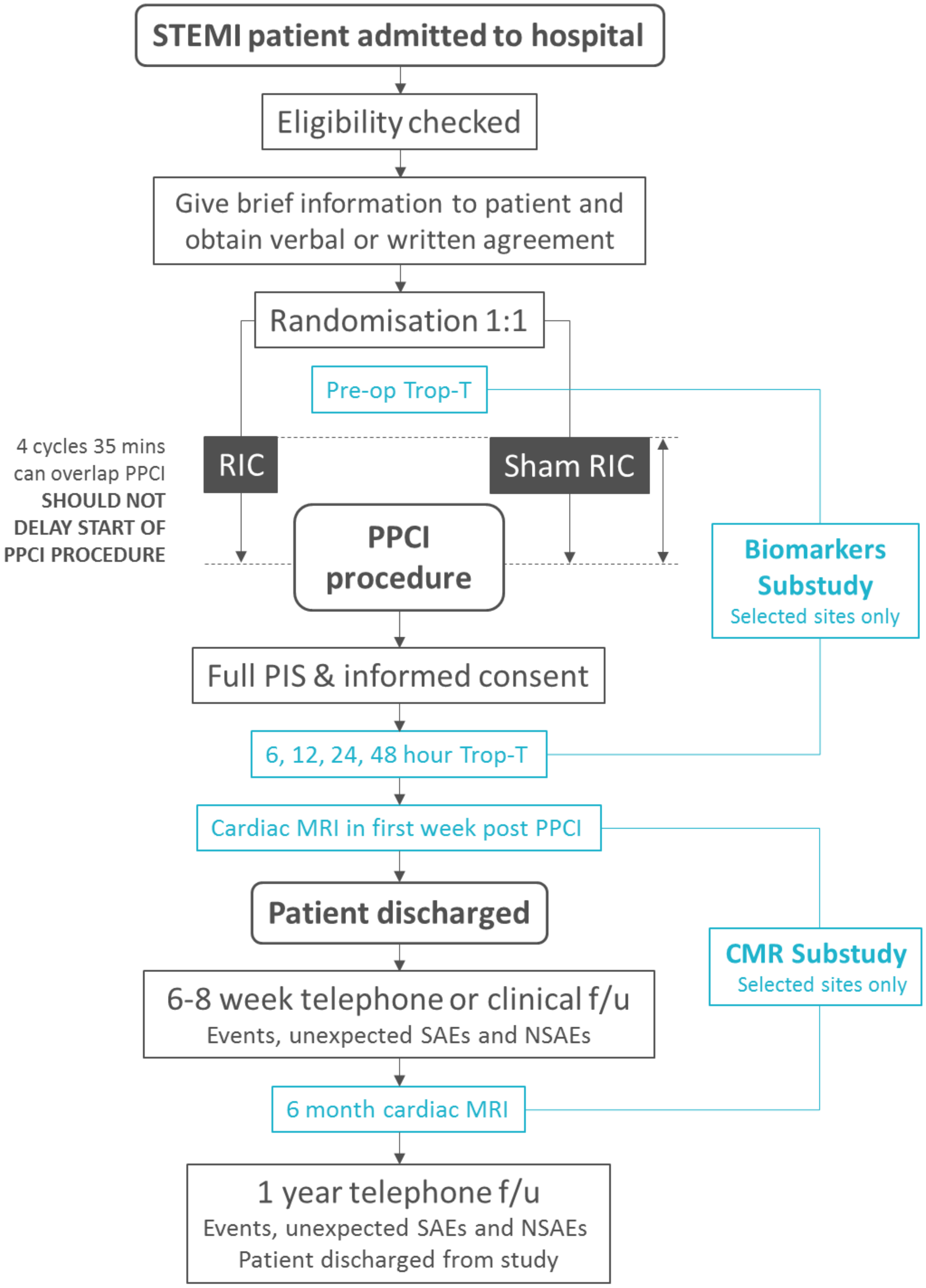

2. Introduction

2.1. Background

Ischaemic heart disease, the leading cause of death in the UK, accounts for 90,000 deaths per year and costs the UK economy nearly £9 billion per annum. Each year 150,000 patients have an acute myocardial infarction (BHF Stats 2010). In patients presenting with an ST-elevation myocardial infarction (STEMI), early myocardial reperfusion using primary percutaneous coronary intervention (PPCI) is the most effective therapeutic intervention for limiting myocardial infarct (MI) size, a major determinant of prognosis post-PPCI. However, despite PPCI, the morbidity and mortality of STEMI patients remain significant, paving the way for novel therapeutic strategies for protecting the heart against acute ischaemia-reperfusion injury (IRI). In this respect remote ischaemic conditioning (RIC) represents a non-invasive and low cost therapeutic strategy for further reducing MI size, preventing the onset of heart failure, and improving clinical outcomes in PPCI patients. For systematic reviews on RIC please see the following references[1, 2].

2.2. Scientific rationale

RIC describes the phenomenon in which the application of multiple cycles of brief non-lethal ischaemia and reperfusion to an organ or tissue remote from the heart protects the myocardium from a lethal sustained episode of acute IRI[1, 2]. The mechanisms underlying RIC are unclear but have been attributed to a neuro-hormonal pathway linking the preconditioned organ or tissue to the heart [2-4]. In 2002, Kharbanda et al[5] first demonstrated that the RIC stimulus could be induced non-invasively in human volunteers by simply inflating and deflating a blood pressure cuff placed on the upper arm. We and others have shown that this RIC stimulus is beneficial in reducing perioperative myocardial injury in the settings of cardiac bypass and abdominal aortic aneurysm surgery[6-9], and reducing periprocedural myocardial and renal injury in the setting of elective PCI[10, 11]. However, several recent studies have reported neutral findings with RIC in these clinical settings[12-15]. The reasons for this are unclear but have been attributed to differences in study design, patient selection and concomitant medication (which are known to interfere with RIC cardioprotection)[16]. Preliminary data suggest that long term outcomes may be improved in RIC treated CABG patients[17], although this needs to be confirmed in prospective large randomised controlled clinical trials such as ERICCA[18] and RIPHeart,[19] which have been prospectively designed and powered to investigate the effect of RIC on long term clinical outcomes in patients undergoing a coronary artery bypass graft operation.

RIC has now been investigated in four small proof-of-concept clinical studies in STEMI patients undergoing PPCI including one by this research group[20-24]. The first study was published by Botker et al (the research collaborator in Denmark)[20, 21] and reported that RIC (four 5 minute upper arm cuff inflations and deflations) administered in the ambulance by paramedics on route to the PPCI centre, significantly increased the mean salvage index from 0·57 in control to 0.69 with RIC at 30 days (as measured by myocardial SPECT). In a post-hoc subgroup analysis of patients presenting with a left anterior descending STEMI, myocardial salvage was increased further, and there was a significant reduction in final MI size and improvement in LV ejection fraction at 30 days[20, 21]. Interestingly, 6 year follow up of this patient cohort revealed less all-cause death in those patients given RIC at the time of their PPCI, although this study has not been prospectively designed to investigate long term endpoints [25]. In another study of 96 patients, Rentoukas et al[22] demonstrated that RIC (three 4 minute upper arm cuff inflations and deflations) administered at the PPCI centre improved ST-segment resolution and reduced MI size when compared to control. Crimi et al [23] have found that RIC administered at the onset of myocardial reperfusion was beneficial in STEMI patients undergoing PPCI.

2.3. Research collaboration with Denmark

The ERIC-PPCI trial will be conducted in collaboration with Prof Hans Erik Bøtker (Aarhus University, Denmark), who is PI of the Effect of RIC on Clinical Outcomes in STEMI Patients Undergoing pPCI (CONDI-2) trial (NCT01857414).

The CONDI 1 trial (CI Hans Bøtker) was the first to successfully demonstrate a beneficial effect of RIC in 142 STEMI patients undergoing PPCI in terms of increased myocardial salvage and smaller MI size [20], effects which were present 6 months later in terms of improved LV systolic function [26]. A €3.0 million research grant has been awarded by the Danish Research Council to investigate the effect of RIC on 12 month clinical outcomes in STEMI patients undergoing PPCI.

The results of ERIC-PPCI and CONDI 2 will be combined in order to maximise power to assess the impact of RIPC on clinical endpoints.

3. Hypothesis

Remote ischaemic conditioning improves long term clinical outcomes (cardiac death and hospitalisation for heart failure) at 12 months in STEMI patients undergoing PPCI.

4. Endpoints

4.1. Primary endpoint

To investigate whether RIC reduces the combined primary endpoint rate of cardiac death and hospitalisation for heart failure (HHF) at 12 months in STEMI patients undergoing PPCI.

4.1.1. Definition for cardiac death

All deaths where there is no clinical or post mortem evidence of a non-cardiac aetiology.

4.1.2. Definition for hospitalisation for heart failure

This will include both heart failure during the index hospitalisation and re-hospitalisation for heart failure. Hospitalisation will be defined as a treatment occurring in hospital. Heart failure will be judged to be present on symptoms (at least one of the following: New or worsening dyspnea, orthopnea, paroxysmal nocturnal dyspnea or increasing fatigue/worsening exercise tolerance) and signs (one of the following: new pulmonary oedema by chest X-ray in the absence of a non-cardiac cause, crepitations believed to be due to pulmonary oedema, and use of loop diuretics to treat presumed pulmonary congestion)[41].

4.2. Secondary endpoints

To investigate whether RIC can affect the following secondary endpoints below:

1. Rates of cardiac death and HHF at 30 days. This data will be collected at the clinical follow up outpatient appointment (at 6-8 weeks post-PPCI).
2. Rates of all-cause death, repeat coronary revascularisation, reinfarction, stroke at 30 days and 12 months. This data will be collected at the clinical follow up outpatient appointment (at 6-8 weeks and one year post-PPCI).
3. TIMI flow post PPCI[28]
4. Quality of life at 6-8 weeks and 12 months (EuroQol EQ-5D-5L) (for an example EQ-5D-5L see Appendix 3)

Biomarkers substudy (400 patients at selected sites)

5. MI size on 48 hour area-under-the-curve (AUC) hsTrop T and CK-MB

MRI substudy (250 patients at selected sites)

6. MI size on 6 months cardiac MRI (new late gadolinium enhancement expressed as a percentage of the LV mass)
7. Microvascular obstruction on cardiac MRI (hypodense area on late gadolinium enhancement).
8. Myocardial salvage index (AAR [T2 weighted imaging or angiography jeopardy score] subtract final MI size).
9. LV remodelling on 6 month cardiac MRI scan:
    - index LV end-diastolic and end-systolic volumes
    - LV ejection fraction
    - LV mass and wall thickness

4.2.1. Definition of stroke

Stroke is defined as a focal, central neurological deficit lasting >72 hours which results in irreversible brain damage or body impairment. Probable stroke will be recorded if there are features suggestive of this but insufficient evidence to classify as definite stroke.

Both definite and probable stroke will be included in the secondary endpoint.

4.2.2. Definition of reinfarction

Reinfarction is defined as an acute MI that occurs within 28 days of an incident or recurrent MI. The ECG diagnosis of suspected reinfarction following the initial MI may be confounded by the initial evolutionary ECG changes. Reinfarction should be considered when ST elevation >0.1 mV recurs, or new pathognomonic Q waves appear, in at least two contiguous leads, particularly when associated with ischemic symptoms for 20 min or longer. Re-elevation of the ST-segment can, however, also be seen in threatened myocardial rupture and should lead to additional diagnostic workup. ST depression or LBBB alone are nonspecific findings and should not be used to diagnose reinfarction.

In patients in whom reinfarction is suspected from clinical signs or symptoms following the initial MI, an immediate measurement of cTn is recommended. A second sample should be obtained 3–6 h later. If the cTn concentration is elevated, but stable or decreasing at the time of suspected reinfarction, the diagnosis of reinfarction requires a 20% or greater increase of the cTn value in the second sample. If the initial cTn concentration is normal, the criteria for new acute MI apply.

4.2.3. Definition of coronary revascularisation

Revascularisation is defined as coronary revascularisation by PCI or CABG following index PPCI, excluding staged PPCI.

4.3. Endpoint Validation

The primary combined endpoint, stroke, MI, reinfarction and revascularisation will be validated by an independent event validation committee (EVC). The EVC will be blinded to the randomised treatment allocation.

5. Power calculations and sample size determination

5.1. Primary combined clinical endpoint

The primary combined endpoint will be cardiac death and HHF at 12 months. These endpoints have been selected as they are the most relevant clinical endpoints that are likely to be affected by RIC, an intervention which protects cardiomyocytes against acute IRI. According to the UK NIAP 2008 database, cardiac mortality at 12 months ranged from 5.8%-16.7% depending on the call to balloon time, with the overall 12 month death rate of 8.7% for all PPCI patients. In a recent Danish clinical study post-PPCI, the one year mortality was 9.4% and the cumulative risk of readmission with heart failure was 8% [30]. In another, non-UK based, clinical study, the incidence of HHF was 12.7% at 12 months post-PPCI [31]. We have based our power calculations on these published studies, accounting for the marked improvements in clinical outcomes in the contemporary era by using much more conservative event rates.

As a conservative estimate we will use a combined cardiac death and HHF event rate of 11.0% at 12 months for all-comer STEMI patients. In the combined ERIC-PPCI / CONDI 2 trial we estimate the effect size to be a 25% relative reduction in the event rate. The rationale for this is based on proof-of-concept clinical studies in which RIC, and related therapeutic interventions such as ischaemic postconditioning, have reported 40-50% reductions in MI size[32]. To demonstrate a 25% reduction in the primary composite endpoint in the RIC-treated group (from 11.0% to 8.25%), with 80% power and at the 5% significance level, will require 1805 patients per treatment arm which equates to 3610 patients in total. Therefore, we will need to recruit 4300 STEMI patients (allowing for a 15% drop out rate at 12 months) between UK and Denmark (2000 STEMI patients in the UK and 2300 STEMI patients in Denmark). Therefore, in the ERIC-PPCI trial we intend to recruit 2000 STEMI patients in the UK through 30 PPCI sites.

If the event rate in the control arm is higher than the estimated 11% or the losses less than 15% then the power will increase. The ERIC-PPCI UK arm of the trial of 2000 patients alone would provide 80% power to detect a reduction of approximately a third from 11.0% to 7.3% allowing for 5% losses to follow up.

5.2. Biomarker substudy to assess the effect of RIC on MI size (48 hr AUC)

A major secondary endpoint of the ERIC-PPCI study will be MI size as measured by 48 hr AUC high-sensitive Troponin T and CK-MB. To date the effect of RIC on this specific endpoint has not been investigated. Using 72 hr AUC Trop I, Thibault et al [32] demonstrated that ischaemic postconditioning reduced MI size from 24.6 (SD 20.6) x10^4 to 13.0 (SD 7.0) x10^4 IU/L. This equates to a relative reduction in MI size of 47%. In order to demonstrate a more conservative 25% relative reduction in MI size with RIC in PPCI patients from 24.6 (SD 20.6) x10^4 using 48 hr AUC hsTrop T with 80% power and at the 5% significance level, will require 177 patients in each treatment group or 354 patients in total. Allowing for 10% dropout rate would require 400 patients in total.

In view of the clear positive skewness of the Troponin AUC it is anticipated that this outcome will be analysed on the log scale. This will lead to estimation of the relative reduction in the MI size, which is expected to be a more precise estimate of any treatment effect. Therefore, the planned sample size of 400 patients is likely to provide greater than 80% power and allow for larger than a 10% loss in outcomes due to incomplete Troponin AUCs.

5.3. CMR substudy to assess the effect of RIC on MI size at 6 months

The CMR substudy primary endpoint is MI size (mass of late gadolinium enhancement) expressed as percentage of LV mass at 6 months. No clinical studies have previously used cardiac MRI to assess the effect of RIC on MI size. Using myocardial SPECT, Botker et al [20] were unable to demonstrate a significant reduction in MI size at 30 days in all STEMI patients. Lonborg et al [33] used CMR to demonstrate that ischaemic postconditioning reduced MI size at 3 months post-PPCI from 17±8% to 14±7% (mass of infarct expressed as a % of the LV mass). This equates to a relative reduction in MI size of 17.6% (equivalent to an absolute reduction of 3%). Based on this data, to demonstrate a similar relative reduction in MI size with RIC in PPCI patients from 17% (SD 8%) in the 6 month CMR scan, with 80% power and at the 5% significance level, will require approximately 112 patients in each treatment group or 224 patients in total. To allow for a 10% dropout rate we plan to recruit 250 patients in total.

6. Selection of patients

6.1. Inclusion criteria

1. Onset of STEMI symptoms within 12 hours, lasting for more than 30 minutes
2. Patients older than 18 years
3. Suspected STEMI (ST-elevation at the J-point in two contiguous leads with the cut-off points: ≥0.2 millivolt (mV) in men or ≥0.15 mV in women in leads V2-V3 and/or ≥0.1 mV in other leads)
4. Eligibility for coronary angiography with follow on PPCI if indicated

6.2. Exclusion criteria

1. Previous coronary artery bypass graft surgery
2. Myocardial infarction (MI) within the previous 30 days
3. Treatment with thrombolysis within the previous 30 days
4. Left bundle branch block (LBBB)
5. Patients treated with therapeutic hypothermia
6. Conditions precluding use of RIC (paresis of upper limb, use of an a-v shunt)
7. Life expectancy of less than 1 year due to non-cardiac pathology

For centres recruiting into CMR substudy study only: Please refer to section 13 as additional exclusion criteria apply to substudy patients.

7. Ethical considerations

7.1. Consent

As patients admitted with a STEMI will need to receive urgent treatment, randomisation and the trial intervention will need to be started as early as possible to ensure that there is no delay to the PPCI procedure. The physical, mental and emotional state of patients may also be affected due to their MI and the use of analgesic drugs in pain management, which could impair their decision making capacity. Therefore, the consent process in this situation requires careful consideration bearing in mind applicable regulatory requirements, adherence to ICH-GCP and the requirements in the Declaration of Helskini.

7.1.1. Patient agreement

The patient will be approached at the time that their STEMI is confirmed and information about the trial should be provided to the patient to their level of capacity. A patient leaflet is provided for this purpose. The patient leaflet may be signed by the patient if they agree to participate, however verbal agreement is also acceptable. Agreement to take part in the trial should be recorded in the patient notes. A signed patient leaflet is not mandatory and will not be considered as equivalent to informed consent.

7.1.2. Informed consent

After the procedure the patient should be given sufficient time to consider the trial and ask questions, following which informed consent will be taken. If the patient elects to withdraw from the study at this point then we will seek consent to use the data and samples already acquired.

7.1.1. Patients lacking capacity

A friend or relative’s advice should be taken on whether they think the patient would wish to be part of the trial, particularly if the patient had expressed any prior wishes or advanced decisions. A consultee declaration form should be completed.

7.1.2. Withdrawal

A patient may decide to withdraw from the trial at any time without prejudice to their future care.

7.1.3. Patients not surviving the PPCI procedure

Written consent will not be available for those patients that die during the PPCI procedure. A friend or relative will be contacted to ask whether we can continue to use the data collected in the trial so far. Verbal agreement from the friend or relative is acceptable in this case, or they may complete the consultee declaration form.

7.2. Declaration of Helsinki and UCL Good Clinical Practice

The trial will conform to the spirit and the letter of the declaration of Helsinki, and in accordance with the UCL Good Clinical Practice Guidelines.

7.3. Ethical committee review

Note to REC: This section will be completed once the outcome of the ethics application is known:

…….Research Ethics Committee have reviewed and approved the trial. The REC number is ….. .

Copies of the letters of approval will be filed in the trial site files at each centre.

8. Randomisation

8.1. Randomisation procedure

Patients will be randomised to either RIC or control by a designated research investigator, this investigator will therefore be unblinded to treatment allocation. Randomisation will be coordinated centrally by the LSHTM CTU via a secure website www.sealedenvelope.com and will be stratified by centre using random permuted blocks.

8.2. Access to randomisation site

Access to the randomisation website will be strictly controlled at each site and limited to unblinded research investigators delegated by the PI to be responsible for performing either the RIC or sham RIC protocol.

Patients will be randomised to receive either **RIC** or **sham RIC**.

9. Blinding

9.1. Unblinded trial staff

At each site specific staff will be delegated by the PI to perform the randomisation and intervention procedures. These staff will be the only people in each centre aware of the treatment allocation for the patient and will not be involved with data collection other than any relating to the randomisation and intervention procedures.

At the LSHTM clinical trials unit only the unblinded trial statistician will have access to allocation data.

9.2. Blinded trial staff

The PI at each site, patients, the interventional cardiologist undertaking the PPCI procedure, other catheter laboratory staff and the blinded research doctor/nurse collecting the data and following up the patient will be blinded to the treatment allocation. Outcome assessments will be blinded. The event validation committee will be blinded to treatment allocation.

In the CMR substudy the research fellow analysing the CMR scan will be blinded to the treatment allocation.

9.3. Emergency unblinding

The benign and short term nature of the intervention makes the need for emergency unblinding unlikely, if required patients will be able to be unblinded through the randomisation website.

10. Trial treatment

Patients will be randomised on arrival at the hospital and the unblinded research investigator will then deliver either the RIC or sham RIC protocol.

Automated CellAegis autoRIC™ cuff devices www.cellaegisdevices.com will be used to deliver the RIC and sham RIC protocols.

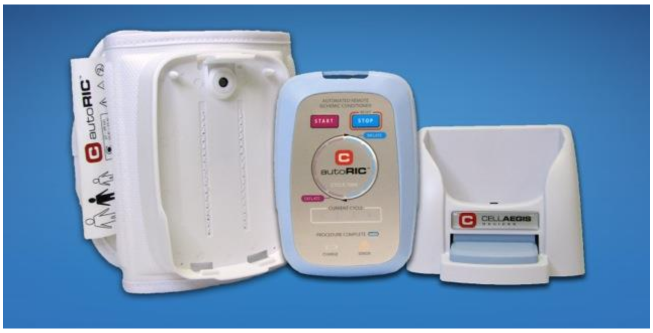

AutoRIC™ devices are used in conjunction with single use disposable inner cuffs. An autoRIC™ device, an autoRIC™ sham device and disposable cuffs will be provided to the participating sites.

The advantage of the autoRIC™ device is that once it has been placed on the upper arm, the preprogrammed standard RIC or sham RIC protocol is simply delivered by pressing a single start button.

10.1. Remote ischaemic conditioning (RIC)

An automated autoRIC™ cuff will be placed on the upper arm and inflated to 200mmHg for 5 minutes and then deflated for 5 minutes, a cycle which will be undertaken 4 times in total. For patients presenting with a systolic blood pressure (SBP)≥175mmHg, a manual blood pressure cuff will be used and inflated to 25 mmHg above systolic blood pressure.

10.2. Sham RIC

An autoRIC™ cuff visually identical to that used in the RIC protocol will be placed on the upper arm and a simulated RIC protocol applied. Inflation will be simulated and held for 5 minutes, deflation will then be simulated and held for five minutes, a cycle which will be undertaken 4 times in total.

The sham device's components and external appearance are identical to that of the autoRIC. However, as compared to the autoRIC, the sham device control unit's pump is disconnected such that the control unit cannot inflate the applicator cuff. The sham device provides the same sound and vibration as that of the pump inflating and the same LED indicators on the control unit. The operation of the autoRIC control unit with respect to the (simulated) RIC procedure initiation, cycle indication, and termination are identical in the sham device, with the exception that the applicator cuff does not inflate.

10.3. Duration of treatment

Although the RIC / sham RIC protocol lasts 40 minutes in total, the cuff should be removed after the 4th cycle of inflation and the last 5 min of reperfusion undertaken with the cuff removed. This means that after 35 min the intervention is completed. In the cases where the door to PPCI time is less than 35 min, the RIC / sham RIC protocol may overlap with the beginning of the PPCI procedure when the coronary angiogram is being performed. Experimental studies have reported that the RIC stimulus is still effective even if given after the onset of reperfusion[34].

Under no circumstances should the RIC protocol delay the onset of the PPCI procedure.

11. Data collection and follow up

11.1. Trial procedures table

	pre-PPCI	PPCI	Post-PPCI in hospital					After discharge		
		0 hrs	6 hrs	12 hrs	24 hrs	48 hrs	2-7 days	6-8 weeks	6 months	12 months
**Clinical assessments**										
Review of eligibility criteria	X									
Patient agreement	X									
History and examination	X									
PIS & Informed consent			X →							
**Trial intervention**										
Randomisation	X									
RIC / Sham RIC*	X →									
**Clinical outcomes**										
Cardiac death		X			X			X		X
HHF					X			X		X
MI		X			X			X		X
Revascularisation					X			X		X
Stroke		X			X			X		X
**Quality of life**										
EQ-5D-5L								X		X
**Safety reporting**										
SAE / NSAEs		X			X			X		X
**Substudies (at selected sites only)**										
Biomarker substudy: Trop-T and CK-MB		X	X	X	X	X				
CMR substudy: Cardiac MRI							X		X	

* The RIC/Sham RIC may overlap with the PPCI procedure and should not delay the start of PPCI

11.2. **Data collection**

All patients will have a full medical history taken and various clinical examinations as part of usual care. The following are to be recorded on the trial CRF:

- Weight and height (BMI will be calculated automatically)
- Blood Pressure
- Heart Rate
- Gender
- Ethnicity
- Date of birth
- Medical history:
  - Diabetes Mellitus
  - Hypercholesterolaemia
  - Hypertension
  - Previous myocardial infarction
  - Previous PCI
  - Previous CABG
  - Previous stroke
  - Atrial fibrillation
  - Peripheral arterial disease
  - Smoking history
  - Family history of IHD
  - Other medical history
- NYHA class
- CCS class
- Ejection fraction (if recorded)
- Analgesia use
- Morphine doses
- Medication at admission and discharge:
  - Aspirin
  - β-blocker
  - Calcium-channel blocker
  - Nitrates
  - Cholesterol-lowering drug
  - ACE inhibitor/A2 receptor antagonist
  - Insulin
  - Sulphonylurea
  - Metformin
  - Other medication
- ECG at admission and prior to discharge
- Call to balloon time, door to balloon time and symptoms to balloon time
- Angiographic data (TIMI flow pre and post-PPCI)
- Use of thrombectomy
- Details of the PPCI procedure
- Procedural drugs
- NHS number. Mortality data will be tracked up to 10 years after randomisation.

11.3. Trial procedures

On admission

- Review of eligibility criteria
- Provide information to patient's level of capacity
- Patient agreement (please refer to section 7.1)
- Randomisation
- Randomly allocated trial intervention – RIC or sham RIC
- Biomarker substudy only: baseline Troponin-T/CK-MB

Post-PPCI

- Full Patient Information Sheet
- Informed consent
- Biomarker substudy only: 6, 12, 24 and 48 hour Troponin-T/CK-MB
- CMR Substudy only: 2-7 day cardiac MRI
- Events
  - Death
  - Heart failure during the index hospitalisation
  - MI
  - Stroke
- Unexpected SAEs and NSAEs

6-8 weeks post-PPCI (telephone or outpatient follow-up)

- Events
  - Death
  - Hospitalisation for heart failure
  - MI
  - Stroke
  - Revascularisation
- Quality of life (EQ-5D-5L)
- Unexpected SAEs and NSAEs
- Blinding check (patient is asked which allocation they think that they received)

6 months post-PPCI

- CMR Substudy only: 6 month cardiac MRI (outpatient appointment)

12 months post-PPCI (telephone follow up)

- Events
  - Cardiac Death
  - Hospitalisation for heart failure
  - Other cause of death
  - MI
  - Stroke
  - Revascularisation
- Quality of life (EQ-5D-5L)
- Unexpected SAEs and NSAEs

11.4. Compliance and loss to follow up

Problems with compliance are expected to be rare given that the intervention is non-invasive in nature and is administered at a single time point.

Patients are free to withdraw from the trial at any time without prejudice to their future care. Loss to follow up is expected to be rare as the trial is designed to be minimally disruptive to participants. Data collected up to the point of withdrawal will be used unless the patient specifically requests that it is not.

The patients will be followed up at 6-8 weeks and 12 months after PPCI, in order to determine endpoints contributing to the primary endpoint (cardiac death and hospitalisation for heart failure). These follow ups will either be planned to coincide with existing clinical appointments or will otherwise be conducted by telephone. A non-compliance and dropout rate of 15% has been accounted for in the sample size.

12. Biomarker substudy

MI size as measured as the 48 hour AUC serum level of high-sensitive Troponin-T (hsTropT) and CK-MB will be analysed in 400 patients. After the data has been acquired for the MI size in 400 patients a prespecified interim analysis will be performed.

12.1. Procedure

From each patient, a single blood sample will be taken at each of the 5 timepoints (0, 6, 12, 24 and 48 hours following PPCI procedure). Each blood sample will be processed (centrifuged to separate serum) and stored (at -20°C or below) at the local hospital. The serum samples will be couriered every 3 months to a central laboratory in London. The central laboratory will supply polyboxes and dry ice for transporting samples.

12.2. Analysis methods

Quantitative serum hsTropT measurement will be performed using a one step immunoassay based on electrochemiluminescence technology (Elecsys 2010, Roche, Switzerland). The reference range will be ≤14 ng/L (14 ng/L is the 99th centile of reference population with cardiovascular risk of <10%).

Quantitative serum CK-MB measurement will be performed using a standard immunoassay method.

13. CMR substudy

The CMR substudy will recruit 250 PPCI patients through a selection of PPCI centres with facilities for performing CMR scans in PPCI patients. All sites performing CMR for the ERIC-PPCI study will have a Siemens 1.5 T scanner and will use a standardised CMR protocol which is included in this protocol as appendix 1.

Training in the CMR protocol will be provided to each recruiting site. Each patient will receive two CMR scans, the first performed within the week following the PPCI procedure and the second at 6 months. All CMR scans will be analysed at a central CMR core lab which will be staffed by an independently funded senior CMR clinical fellow.

The ERIC-PPCI CMR substudy will also put the research infrastructure in place for future PPCI/CMR clinical studies in the UK.

13.1. CMR substudy endpoints

The primary endpoint of the CMR substudy will be MI size on the 6 month CMR scan (measured in mass of late gadolinium enhancement and expressed as a percentage of the LV mass).

Several other CMR parameters will be collected as follows:

13.1.1. The acute post-PPCI CMR scan

1. Left ventricle (LV) ejection fraction and indexed LV end systolic and diastolic volumes and mass using short axis SSFP cine imaging.
2. MI size measured by the mass of late gadolinium enhancement (20 min after administration of contrast) of cardiac MRI scan expressed as a percentage of LV mass.
3. Area at risk (AAR) measured as the increase in T2 values using a Siemens T2 mapping sequence, which has been validated against conventional measures of area at risk [35-37]. The AAR will also be estimated using the modified BARI and APPROACH angiography scores [38].
4. Myocardial salvage index = AAR subtract MI size/AAR. The myocardial salvage index (using T2-weighted CMR and late gadolinium enhancement) has been demonstrated to predict prognosis post-PPCI [39].
5. The incidence and extent of microvascular obstruction (hypo-enhancement on late gadolinium enhancement 20 min after administration of contrast).
6. The incidence and extent of intramyocardial haemorrhage (hypo-enhancement on Siemens T2* mapping sequence)[40].

13.1.2. The follow up CMR scan 6 months post-PPCI

1. LV ejection fraction and indexed LV end systolic and diastolic volumes and mass.
2. MI size measured by the mass of late gadolinium enhancement.

13.2. Exclusion criteria for CMR substudy

Known contraindication to cardiac magnetic resonance imaging (MRI) such as:

1. Significant claustrophobia
2. Severe allergy to gadolinium chelate contrast
3. Severe renal insufficiency (defined as estimated glomerular filtration rate [eGFR] <30 mL/min/1.73 m2)
4. Presence of MRI contraindicated implanted devices (e.g. pacemaker, implanted cardiac defibrillator, cardiac resynchronisation therapy device, cochlear implant)
5. Embedded metal objects (e.g., shrapnel)
6. Any other contraindication for cardiac MRI

Patients may still enroll in the main trial without enrolling in the CMR substudy.

14. Safety Reporting

14.1. Definition

Unexpected events that have not been defined as endpoints (section 4), expected complications of the RIC stimulus or expected complications of usual clinical care (section 14.3) should be reported as either an SAE or NSAE, depending on their severity. Safety reporting for each patient should commence from time of randomisation to completion of follow up at one year after the PPCI procedure.

14.2. Expected adverse events (recognised to be caused by the RIC stimulus)

The benign nature of the RIC stimulus excludes there being any expected serious adverse events. The following are expected non-serious events in response to the RIC stimulus and will be recorded on the Case Report Form. They do not need to be reported to the Clinical Trials Unit.

1. Skin petechiae caused by cuff inflation

14.3. Expected serious adverse events related to usual clinical care

These events are recognised complications of PPCI. They will be recorded on the Case Report Form but do not need to be reported separately on an SAE form:

1. Death
2. Acute renal failure which may require haemodialysis, peritoneal dialysis, or haemofiltration
3. Ventricular tachycardia of fibrillation requiring direct-current (DC) cardioversion
4. Significant heart block requiring temporary or permanent cardiac pacing
5. Tamponade requiring urgent surgical intervention
6. Cardiogenic shock requiring intra-aortic balloon pump or other assist devices

The following events are recognised complications of routine clinical care and for the purposes of this trial will not be designated as SAEs. They do not need to be reported:

1. Atrial fibrillation
2. Acute mitral valve cordal rupture or ventricular septal rupture requiring surgical intervention
3. Persistent complete heart block requiring permanent pacemaker implantation
4. Aspiration pneumonia following VF arrest
5. Rib fracture following chest compression

14.4. Unexpected Serious Adverse Events

Any untoward medical occurrence/effect that:

1. Results in death
2. Is life-threatening*
3. Requires hospitalisation or prolongation of existing inpatient's hospitalisation
4. Results in persistent or significant disability or incapacity

**Life-threatening* in the definition of a serious adverse event refers to an event in which the patient was at risk of death at the time of event; it does not refer to an event which hypothetically might have caused death if it were more severe.

SAEs should be reported to the Clinical Trials Unit within 7 days. The report should include an assessment of causality by the Principal Investigator at each site (see section 14.6.2). The Chief Investigator will be responsible for the prompt notification of findings that could adversely affect the health of patients or impact on the conduct of the trial. Notification of confirmed unexpected and related SAEs will be to the Sponsor, the Research Ethics Committee and the Data and Safety Monitoring Committee (DSMC).

14.5. Unexpected Non-Serious Adverse Events

Unexpected non-serious adverse events should be evaluated by the Principal Investigator or research nurse. This should include an assessment of causality (see section 14.6.2) and intensity (see section 14.6.1) and reports made within 14 days. The Clinical Trials Unit will keep detailed records of all unexpected adverse events reported. Reports will be reviewed by the Chief Investigator to consider intensity, causality and expectedness. As appropriate these will be reported to the sponsor, the DSMC and the Ethics Committee.

14.6. Reporting unexpected adverse events

Investigators will make their reports of all unexpected adverse events, whether serious or not, to the Clinical Trials Unit, London School of Hygiene and Tropical Medicine.

14.6.1. Assessment of intensity

Mild: The patient is aware of the event or symptom, but the event or symptom is easily tolerated.

Moderate: The patient experiences sufficient discomfort to interfere with or reduce his or her usual level of activity.

Severe: Significant impairment of functioning; the patient is unable to carry out usual activities and/or the patient's life is at risk from the event.

14.6.2. Assessment of causality

Probable: A causal relationship is clinically / biologically highly plausible and there is a plausible time sequence between onset of the adverse event and the RIC procedure.

Possible: A causal relationship is clinically / biologically plausible and there is a plausible time sequence between onset of the adverse event and the RIC procedure.

Unlikely: A causal relationship is improbable and another documented cause of the adverse event is most plausible.

Unrelated: A causal relationship can definitely be excluded and another documented cause of the adverse event is most plausible.

15. Withdrawal of patients

15.1. Criteria for withdrawal from the trial

A patient may decide to withdraw from the trial at any time without prejudice to their future care. Withdrawal will be uncommon, because of the non-invasive nature of the planned intervention and the follow up which will be integrated within routine clinical care wherever possible. We have allowed in our sample size calculation for a non-compliance and drop out rate of up to 15% although it is expected to be lower than this.

15.2. Follow up of patients withdrawing from the trial

Patients who are randomised but withdraw before the intervention will undergo standard clinical care according to local protocols. If patients undergo the intervention but subsequently withdraw, they will undergo standard clinical care. Patients will be encouraged to allow data and samples that have been collected before withdrawal to be used in the analyses. However, if consent to use data/samples is also withdrawn, then these will be discarded. Patients withdrawing from the study will continue to be followed up by their local team. There should be no need for further follow up from the research team.

15.3. Reporting withdrawal of patients

The clinical trials unit at LSHTM should be informed by email if a patient has withdrawn from the trial. A withdrawal from will be completed on the trial CRF.

16. Statistics

16.1. Trial statistician

Statistical analysis will be coordinated from the Clinical Trials Unit at London School of Hygiene and Tropical Medicine.

16.2. Statistical analysis

A detailed statistical analysis plan will be produced prior to unblinding of any data. The primary analysis will be a comparison of the cardiac death or HHF event rate one year after randomisation between the RIC and control arms of the trial amongst all STEMI patients. Hazard ratios and confidence intervals will be calculated using Cox proportional hazards modelling and Kaplan-Meier curves will be produced. In addition risk differences at one year will also be calculated together with 95% confidence intervals. The results for the individual components of the primary endpoint will also be presented together with other time to event secondary endpoints such as cardiac death or HHF at 30 days. Differences in means (continuous variables) together with 95% confidence intervals will be calculated using linear regression models and analysis of covariance techniques where appropriate. The primary analysis will be performed on an intention to treat basis i.e. by including all patients where possible according to the group to which they were randomised irrespective of whether they received the intervention as allocated. A secondary per protocol analysis will be undertaken including only patients who receive the allocated intervention as intended.

16.3. Planned subgroup analysis

We plan to undertake a limited number of pre specified subgroup analyses: these will be expected to include diabetes, LAD vs non-LAD STEMI, TIMI flow and time of onset of chest pain to PPCI. The subgroup analyses will be detailed in the statistical analysis plan.

16.4. Procedure to account for missing or spurious data

All patients randomised to the trial will be analysed on an intention to treat basis. Data will be validated and the data analysis will take appropriate account of missing values. This process will be detailed in the statistical analysis plan.

17. Data handling and record keeping

Electronic data will be entered on an online database and stored securely on London School of Hygiene and Tropical Medicine servers. Data will be kept for 15 years following completion of the trial. The use of the data from the trial will be controlled by the chief investigator and the Clinical Trials Unit at the London School of Hygiene and Tropical Medicine.

18. Insurance

All recruiting centres will be covered by NHS indemnity for negligent harm providing researchers hold a contract of employment with the NHS, including honorary contracts held by academic staff. Medical coinvestigators will also be covered by their own medical defence insurance for non-negligent harm.

19. Publications policy

It is our intention to disseminate the results of the trial as widely as possible. This is likely to be through a publication in a peer reviewed journal, and through presentations at National and International Cardiology conferences. Publications will follow the CONSORT guidelines. Authorship will follow international guidelines.

20. Expected value of the results

There is an urgent need to improve clinical outcomes in STEMI patients undergoing PPCI. If ERIC-PPCI demonstrates reduced major adverse cardiac events at 12 months in patients treated with RIC at the time of PPCI, there is the potential to change the current management of PPCI patients, to a non-invasive, non-pharmacological, and cost effective therapeutic strategy with benefits in both patient survival and for the prevention of heart failure.

21. Trial organisation

21.1. Trial Steering Committee (TSC)

The TSC will meet every 6 months. The TSC will be responsible for drafting the final report and submission for publication.

Dr Rob Henderson - chair (Independent Interventional Cardiologist)

Prof Derek J Hausenloy (Chief Investigator)

Mr Tim Clayton (Co-Principal Investigator/Senior Medical Statistician with CTU)

Prof Derek Yellon (Co-Principal Investigator)

Dr Rod Stables (Independent Interventional Cardiologist)

Prof Simon Redwood (Co-applicant/Interventional Cardiologist)

Prof Michael Marber (Independent Cardiologist)

Mrs Rosemary Knight (Senior Trial Manager)

Dr Rajesh Kharbanda (Co-Principal Investigator/Interventional Cardiologist)

Mr Paul Hambley (previous PPCI patient)

Mr Alan Berry (previous PPCI patient)

Prof Hans Erik Botker (Interventional Cardiologist and Chief Investigator for Danish CONDI 2 trial)

Observers:

Dr Shannon Amoils (BHF representative)

Ms Tabitha Kavoi (Sponsor representative)

Mr Richard Evans (Trial Manager)

21.2. Project Management Group (PMG)

Prof Derek J Hausenloy (Chief Investigator)

Dr Rajesh Kharbanda (Co-Principal Investigator/Interventional Cardiologist)

Prof Derek Yellon (Co-Principal Investigator)

Mr Tim Clayton (Co-Principal Investigator/Senior Medical Statistician)

Mr Richard Evans (Trial Manager)

Mr Matthew Dodd (Data Manager)

Mrs Rosemary Knight (Senior Trial Manager)

Dr Manish Ramlall (Clinical Research Fellow)

21.3. Data Safety Monitoring Committee (DSMC)

Prof Colin Berry - **Chair** (independent interventional cardiologist)

Prof Tom Meade (Emeritus Professor of Epidemiology)

Andrew Copas (independent statistician)

Dr Jennifer Nicholas (unblinded statistician at the CTU) will support the DSMC

A DSMC Charter will be established with the members of the DSMC.

The DSMC will meet periodically to determine whether there are any unforeseen effects of RIC.

21.4. Endpoint Validation Committee (EVC)

The EVC will meet periodically to validate and adjudicate primary endpoints.

Membership to be decided.

22. References

(1) Przyklenk K, Bauer B, Ovize M, Kloner RA, Whittaker P. Regional ischemic 'preconditioning' protects remote virgin myocardium from subsequent sustained coronary occlusion. *Circulation* 1993;87(3):893-9.

(2) Hausenloy DJ, Yellon DM. Remote ischaemic preconditioning: underlying mechanisms and clinical application. *Cardiovasc Res* 1-8-2008;79(3):377-86.

(3) Lim SY, Yellon DM, Hausenloy DJ. **The neural and humoral pathways in remote limb ischemic preconditioning**. *Basic Res Cardiol* 2010;105(5):651-5.

(4) Redington KL, Disenhouse T, Strantzas SC et al. Remote cardioprotection by direct peripheral nerve stimulation and topical capsaicin is mediated by circulating humoral factors. *Basic Res Cardiol* 2012;107(2):1-10.

(5) Kharbanda RK, Mortensen UM, White PA et al. **Transient limb ischemia induces remote ischemic preconditioning in vivo**. *Circulation* 3-12-2002;106(23):2881-3.

(6) Hausenloy DJ, Mwamure PK, Venugopal V et al. Effect of remote ischaemic preconditioning on myocardial injury in patients undergoing coronary artery bypass graft surgery: a randomised controlled trial. *Lancet* 18-8-2007;370(9587):575-9.

(7) Venugopal V, Hausenloy DJ, Ludman A et al. Remote ischaemic preconditioning reduces myocardial injury in patients undergoing cardiac surgery with cold-blood cardioplegia: a randomised controlled trial. *Heart* 2009;95(19):1567-71.

(8) Thielmann M, Kottenberg E, Boengler K et al. Remote ischemic preconditioning reduces myocardial injury after coronary artery bypass surgery with crystalloid cardioplegic arrest. *Basic Res Cardiol* 2010;105(5):657-64.

(9) Ali ZA, Callaghan CJ, Lim E et al. Remote ischemic preconditioning reduces myocardial and renal injury after elective abdominal aortic aneurysm repair: a randomized controlled trial. *Circulation* 11-9-2007;116(11 Suppl):I98-105.

(10) Hoole S, Heck PM, Sharples L et al. Cardiac Remote Ischemic Preconditioning in Coronary Stenting (CRISP Stent) Study: a prospective, randomized control trial. *Circulation* 17-2-2009;119(6):820-7.

(11) Hong DM, Jeon Y, Lee CS et al. Effects of remote ischemic preconditioning with postconditioning in patients undergoing off-pump coronary artery bypass surgery--randomized controlled trial. *Circ J* 2012;76(4):884-90.

(12) Rahman IA, Mascaro JG, Steeds RP et al. Remote ischemic preconditioning in human coronary artery bypass surgery: from promise to disappointment? *Circulation* 14-9-2010;122(11 Suppl):S53-S59.

(13) Young PJ, Dalley P, Garden A et al. A pilot study investigating the effects of remote ischemic preconditioning in high-risk cardiac surgery using a randomised controlled double-blind protocol. *Basic Res Cardiol* 2012;107(3):1-10.

(14) Iliodromitis EK, Kyrzopoulos S, Paraskevaidis IA et al. Increased C reactive protein and cardiac enzyme levels after coronary stent implantation. Is there protection by remote ischaemic preconditioning? *Heart* 2006;92(12):1821-6.

(15) Prasad A, Gossl M, Hoyt J et al. Remote ischemic preconditioning immediately before percutaneous coronary intervention does not impact myocardial necrosis, inflammatory response and circulating endothelial progenitor cell counts. A single center randomized sham controlled trial. *Catheter Cardiovasc Interv* 19-4-2012.

(16) Hausenloy DJ, Yellon DM. **"Conditional Conditioning" in cardiac bypass surgery**. *Basic Res Cardiol* 2012;107(3):1-6.

(17) Thielmann M, Kottenberg E, Kleinbongard P et al. Cardioprotective and prognostic effects of remote ischaemic preconditioning in patients undergoing coronary artery bypass surgery: a single-centre randomised, double-blind, controlled trial. *Lancet* 17-8-2013;382(9892):597-604.

(18) Hausenloy DJ, Candilio L, Laing C et al. Effect of remote ischemic preconditioning on clinical outcomes in patients undergoing coronary artery bypass graft surgery (ERICCA): rationale and study design of a multi-centre randomized double-blinded controlled clinical trial. *Clin Res Cardiol* 2012;101(5):339-48.

(19) Meybohm P, Zacharowski K, Cremer J et al. Remote ischaemic preconditioning for heart surgery. The study design for a multi-center randomized double-blinded controlled clinical trial--the RIPHeart-Study. *Eur Heart J* 2012;33(12):1423-6.

(20) Botker HE, Kharbanda R, Schmidt MR et al. Remote ischaemic conditioning before hospital admission, as a complement to angioplasty, and effect on myocardial salvage in patients with acute myocardial infarction: a randomised trial. *Lancet* 27-2-2010;375(9716):727-34.

(21) Munk K, Andersen NH, Schmidt MR et al. Remote Ischemic Conditioning in Myocardial Infarct Patients Treated with Primary Angioplasty: Impact on Left Ventricular Function Assessed by Comprehensive Echocardiography and Gated SPECT. *Circ Cardiovasc Imaging* 8-9-2010.

(22) Rentoukas I, Giannopoulos G, Kaoukis A et al. Cardioprotective role of remote ischemic periconditioning in primary percutaneous coronary intervention: enhancement by opioid action. *JACC Cardiovasc Interv* 2010;3(1):49-55.

(23) Crimi G, Pica S, Raineri C et al. Remote ischemic post-conditioning of the lower limb during primary percutaneous coronary intervention safely reduces enzymatic infarct size in anterior myocardial infarction: a randomized controlled trial. *JACC Cardiovasc Interv* 2013;6(10):1055-63.

(24) White SK, Frohlich GM, Sado DM et al. Remote ischemic conditioning reduces myocardial infarct size and edema in patients with ST-segment elevation myocardial infarction. *JACC Cardiovasc Interv* 2014.

(25) Sloth AD, Schmidt MR, Munk K et al. Improved long-term clinical outcomes in patients with ST-elevation myocardial infarction undergoing remote ischaemic conditioning as an adjunct to primary percutaneous coronary intervention. *Eur Heart J* 2014;35(3):168-75.

(26) Munk K, Andersen NH, Schmidt MR et al. Remote Ischemic Conditioning in Patients With Myocardial Infarction Treated With Primary Angioplasty: Impact on Left Ventricular Function Assessed by Comprehensive Echocardiography and Gated Single-Photon Emission CT. *Circ Cardiovasc Imaging* 1-11-2010;3(6):656-62.

(27) van dH, I, De Luca G, Ottervanger JP et al. ST-segment elevation resolution and outcome in patients treated with primary angioplasty and glucose-insulin-potassium infusion. *Am Heart J* 2005;149(6):1135.

(28) Gibson CM, Cannon CP, Daley WL et al. **TIMI frame count: a quantitative method of assessing coronary artery flow**. *Circulation* 1-3-1996;93(5):879-88.

(29) Rentrop KP, Cohen M, Blanke H, Phillips RA. Changes in collateral channel filling immediately after controlled coronary artery occlusion by an angioplasty balloon in human subjects. *J Am Coll Cardiol* 1985;5(3):587-92.

(30) Terkelsen CJ, Jensen LO, Tilsted HH et al. Health care system delay and heart failure in patients with ST-segment elevation myocardial infarction treated with primary percutaneous coronary intervention: follow-up of population-based medical registry data. *Ann Intern Med* 20-9-2011;155(6):361-7.

(31) Zalewski J, Nycz K, Przewlocki T et al. Evolution of myocardial perfusion during primary angioplasty in spontaneously reperfused infarct-related artery: impact on long-term clinical outcomes and left ventricular function recovery. *Int J Cardiol* 17-2-2011;147(1):25-31.

(32) Thibault H, Piot C, Staat P et al. **Long-term benefit of postconditioning**. *Circulation* 26-2-2008;117(8):1037-44.

(33) Lonborg J, Kelbaek H, Vejlstrup N et al. Cardioprotective effects of ischemic postconditioning in patients treated with primary percutaneous coronary intervention, evaluated by magnetic resonance. *Circ Cardiovasc Interv* 1-2-2010;3(1):34-41.

(34) Andreka G, Vertesaljai M, Szantho G et al. Remote ischaemic postconditioning protects the heart during acute myocardial infarction in pigs. *Heart* 2007;93(6):749-52.

(35) Giri S, Chung YC, Merchant A et al. **T2 quantification for improved detection of myocardial edema**. *J Cardiovasc Magn Reson* 2009;11(1):56.

(36) Verhaert D, Thavendiranathan P, Giri S et al. **Direct T2 quantification of myocardial edema in acute ischemic injury**. *JACC Cardiovasc Imaging* 2011;4(3):269-78.

(37) Thavendiranathan P, Walls M, Giri S et al. Improved detection of myocardial involvement in acute inflammatory cardiomyopathies using T2 mapping. *Circ Cardiovasc Imaging* 2012;5(1):102-10.

(38) Ortiz-Perez JT, Meyers SN, Lee DC et al. Angiographic estimates of myocardium at risk during acute myocardial infarction: validation study using cardiac magnetic resonance imaging. *Eur Heart J* 2007;28(14):1750-8.

(39) Eitel I, Desch S, Fuernau G et al. Prognostic significance and determinants of myocardial salvage assessed by cardiovascular magnetic resonance in acute reperfused myocardial infarction. *J Am Coll Cardiol* 1-6-2010;55(22):2470-9.

(40) O'Regan DP, Ahmed R, Karunanithy N et al. Reperfusion hemorrhage following acute myocardial infarction: assessment with T2* mapping and effect on measuring the area at risk. *Radiology* 2009;250(3):916-22.

(41) Ross AM, Gibbons RJ, Stone GW, Kloner RA, Alexander RW. A randomized, double-blinded, placebo-controlled multicenter trial of adenosine as an adjunct to reperfusion in the treatment of acute myocardial infarction (AMISTAD-II). *J Am Coll Cardiol* 7-6-2005;45(11):1775-80.

23. Appendix 1 – CMR protocol

a) All Siemens 1.5 T

b) Transverse half Fourier acquisition single-shot turbo spin-echo sequences for extracardiac anatomical images.

c) Multiplanar balanced steady-state free precession (voxel size, 1.3 x 1.3 x 8 mm3) cine sequences for wall motion abnormalities volumetric analysis.

d) T2 maps acquired from three T2-weighted images at different T2 preparation time (0 ms, 24 ms, and 55 ms, respectively; repetition time = 3 x R-R, voxel 1.9 x 1.9x 6 mm3; motion correction and fitting should then be performed as previously described to obtain the colored T2 maps).

e) Segmented two-dimensional inversion-recovery turbo fast low-angle shot late gadolinium-enhanced (LGE) sequences at 10 - 15 minutes after contrast agent injection (voxel size, 1.3 x 1.3 x 8 mm3).

f) All images to be acquired in breath-hold and to be ECG-triggered.

g) Matching contiguous short-axis views of the entire left ventricle should be obtained for cines, T2 maps and LGE.

h) The contrast agent, Gadoterate meglumine, (gadolinium-DOTA, marketed as Dotarem, Guerbet S.A., Paris, France) at a dose of 0.1 mmol/kg should be administered as a bolus.

24. Appendix 2 – Glossary

AAR	Area at risk	A comparison of the severity of a coronary artery lesion and the volume of heart muscle tissue (myocardium) it supplies. (See also **APPROACH** and **BARI**)
ACS	Acute coronary syndrome	This refers to a group of symptoms caused by obstructed coronary arteries. The symptoms include- 'crushing chest pains', nausea and sweating. These symptoms usually occur as part of an **MI**.
NSAE	Non-Serious Adverse Event	See **SAE**
AF	Atrial fibrillation	A common irregular heartbeat caused by the top chambers in the heart (the atriums) quivering (fibrillating) This rhythm is often the cause of 'palpitations'.
APPROACH	Alberta Provincial Project for Outcome Assessment in Coronary Heart Disease Jeopardy Score	A myocardial jeopardy score used to estimate the amount of myocardium at risk based on the severity of the coronary artery lesion and the volume of myocardium it supplies. (See also **BARI**)
AUC	Area under the curve	Used to calculate release of enzymes (for example Troponin) over time. (See **hsTropT**)
BARI	Bypass Angioplasty Revascularisation Investigation Jeopardy Score	A myocardial jeopardy score used to estimate the amount of myocardium at risk based on the severity of the coronary artery lesion and the volume of myocardium it supplies. (See also **APPROACH**)
BHF	British Heart Foundation	A major funder and authority in cardiovascular research, education and care, and relies predominantly on voluntary donations to meet its aims. In order to increase income and maximise the impact of its work, it also works with other organisations to combat premature death and disability from cardiovascular disease.
Call to balloon time		The time taken from the phone call reporting the heart attack to the start of the angioplasty
CABG	Coronary Artery Bypass Graft	A surgical procedure performed to relieve angina and reduce the risk of death from coronary artery disease. Arteries or veins from elsewhere in the patient's body are grafted to the coronary arteries to bypass atherosclerotic narrowings and improve the blood supply to the myocardium (heart muscle).
CCS class	Canadian cardiovascular society	A simple way of classifying the extent of heart failure (see definition) using physical activity, chest pain and breathlessness as a measure. (See **NYHA**)

CK-MB	Creatinine Kinase	A blood test that measures the presence of cardiac enzymes. These act as markers that can assist in the diagnosis of a heart attack. (See **hs Trop-T**)
CMR	Cardiac MRI	See **MRI**
CONDI-2		The Danish arm of the trial. Data will be combined from ERIC-PPCI and CONDI 2 in the analysis.
CRF	Case Report Form	A specialised form (either on paper, or electronic when it's sometimes called an eCRF) used to collect clinical data for a trial or a study.
CTU	Clinical Trials Unit	A specialised research unit which designs, coordinates and analyses clinical trials and other studies.
DSMC	Data Safety and Monitoring Committee	An independent group of experts formed to monitor patient safety and treatment efficacy data while a clinical trial is ongoing.
ECG	Electrocardiogram	A test that records the electric activity of your heart. (ST elevation/depression, T wave, QRS complex- these terms represent aspects of an ECG reading).
EF	Ejection fraction	See **LVEF**
eGFR	Estimated glomerular filtration rate	This is a test to see how well the kidneys are working. It estimates how much blood is filtered by the kidneys over a given period of time.
HHF	Hospitalisation for heart failure	An admission of longer than 24 hours for heart failure. Heart failure is a health condition in which the heart has a reduced ability to pump blood to the body.
hs Trop-T	High sensitivity Troponin-T	A blood test that measures the presence of cardiac enzymes. These act as markers that can assist in the diagnosis of a heart attack. (see **CK-MB**)
IHD	Ischaemic heart disease	Ischaemia is the restriction in blood supply to tissues, resulting in reduced oxygen and glucose supply affecting the cells, causing pain.
IRI	Ischaemia reperfusion injury	the tissue damage caused when tissue experiences a period of ischaemia (or lack of oxygen) and subsequently blood supply returns to the tissue.
LAD	Left anterior descending	One of the arteries of the heart
LBBB	Left bundle branch block	A cardiac contraction condition where activation of the left ventricle is delayed, causing the left ventricle to contract later than the right ventricle. This may require treatment with a pacemaker.
LGE	Late gadolinium enhancement	See **MRI**
LSHTM	London School of Hygiene and Tropical Medicine	The **clinical trial unit** coordinating the ERIC-PPCI trial is based at LSHTM.
LV	left ventricle / left ventricular	Along with the right ventricle, one of the two large chambers that collect and expel blood in the heart.

LVEF	left ventricular ejection fraction	Often given as a percentage, it is the volumetric fraction of blood pumped out of the left ventricle in the heart with each heartbeat.
MI	Myocardial infarction	Or 'Heart attack'. An Interruption of blood supply caused by a blockage in the blood vessels to the heart leading to cell or tissue death (infarction). Sometimes referred to as NSTEMI or **STEMI**.
MRI	Magnetic resonance imaging	A medical imaging technique used in radiology to visualise internal structures in the body. (LGE- Late gadolinium-enhanced images- A more advanced MRI).
Myocardium		The myocardium is the muscle tissue of the heart, and forms a thick middle layer between the outer epicardium layer and the inner endocardium layer.
NIHR	National Institute for Health Research	The NIHR is the health research arm of the NHS.
NYHA class	New York heart association	A simple way of classifying the extent of heart failure (see definition) using physical activity, chest pain and breathlessness as a measure. See **CCS**.
OMT	Optimal Medical Therapy	This includes the best medication (tablets) that are currently available for heart failure, at doses that are individually tailored. This strategy often also involves insertion of a special type of pacemaker (called a biventricular pacemaker, which may also function as an Implantable Cardioverter Defibrillator).
PI	Principal Investigator	The doctor leading the trial at the site level. Each site has a principal investigator who will delegate roles and responsibilities to other staff using a delegation log.
PIS	Patient Information Sheet	A leaflet given to the patient which explains the trial and their involvement in it in lay language.
PPCI	Primary percutaneous intervention	This procedure is used to treat the narrowed coronary arteries of the heart. A small tube is inserted in the groin or wrist and advanced to the heart. Small balloons and stents are used to open up the narrowings and improve blood flow to the heart muscle. This is sometime also known as Primary Angioplasty.

RIC	Remote ischaemic conditioning	RIC describes the process of applying cycles of limited blood flow (ischaemia) and reinstated blood flow (reperfusion) to an organ or tissue as a protection mechanism for other organs. The full extent and mechanism of this protection is unclear. However there is evidence from previous extensive work on patients that there is a pathway linking the pre conditioned organ or tissue (in the case of ERIC-PPCI the upper arm) to the heart.
SAE	Serious Adverse Event	Any event such as an illness or an accident that results in death, is life-threatening, requires hospitalisation or prolongation of existing hospitalisation, results in persistent or significant disability or incapacity, or is a congenital anomaly or birth defect.
SBP	Systolic Blood Pressure	During each heartbeat, blood pressure varies between a maximum (systolic) and a minimum (diastolic) pressure.
SD	Standard deviation	Measures the amount of variation from the average.
SPECT		An imaging technique that produces three dimensional images of functional processes in the body.
SSFP	Steady-state free precession imaging	A magnetic resonance imaging (**MRI**) technique which uses steady states of magnetisations.
STEMI	ST elevated myocardial infarction	ST elevation refers to a finding on an **ECG**, wherein the trace in the ST segment is abnormally high above the isoelectric line.
TIMI Perfusion Grade (Blush)	Thrombolysis in myocardial infarction perfusion grade	A technique to assess myocardial infusion in the capillary bed on a coronary angiogram
TIMI flow grade	Thrombolysis in myocardial infarction flow grade	A measure to assess epicardial coronary blood flow
UK NIAP	UK National Infarct Angioplasty Project	A joint project set up by the British Cardiac Society and the Department of Health to test the feasibility of implementing a countrywide angioplasty service to treat cases of acute myocardial infarction in England.

25. Appendix 3 – EuroQol EQ-5D-5L

ERIC-PPCI

Effect of Remote Ischaemic Conditioning on clinical outcomes in ST segment elevation myocardial infarction patients undergoing Primary Percutaneous Coronary Intervention

EQ-5D-5L

Health Questionnaire

English version for the UK

ERIC-PPCI Trial Number:		E	P						
Date of Birth:	d	d	m	m	y	y	y	y	

EQ-5D-5L

ERIC-PPCI Trial Number: **E P** | | | | | |

Date completed d d m m y y y y

Under each heading, please tick the **ONE** box that best describes your health **TODAY**

Mobility

I have no problems in walking about ☐
I have slight problems in walking about ☐
I have moderate problems in walking about ☐
I have severe problems in walking about ☐
I am unable to walk about ☐

Self-Care

I have no problems washing or dressing myself ☐
I have slight problems washing or dressing myself ☐
I have moderate problems washing or dressing myself ☐
I have severe problems washing or dressing myself ☐
I am unable to wash or dress myself ☐

Usual Activities (e.g. work, study, housework, family or leisure activities)

I have no problems doing my usual activities ☐
I have slight problems doing my usual activities ☐
I have moderate problems doing my usual activities ☐
I have severe problems doing my usual activities ☐
I am unable to do my usual activities ☐

Pain/Discomfort

I have no pain or discomfort ☐
I have slight pain or discomfort ☐
I have moderate pain or discomfort ☐
I have severe pain or discomfort ☐
I have extreme pain or discomfort ☐

Anxiety / Depression

I am not anxious or depressed ☐
I am slightly anxious or depressed ☐
I am moderately anxious or depressed ☐
I am severely anxious or depressed ☐
I am extremely anxious or depressed ☐

EQ-5D-5L

ERIC-PPCI Trial Number: **E P** ☐☐☐☐☐

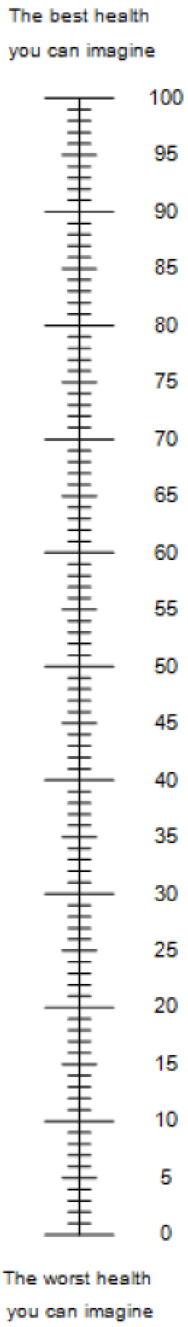

- We would like to know how good or bad your health is TODAY.
- This scale is numbered from 0 to 100.
- 100 means the **best** health you can imagine.
- 0 means the **worst** health you could imagine.
- Mark an X on the scale to indicate how your health is TODAY.
- Now, please write the number you marked on the scale in the box below.

YOUR HEALTH TODAY = ☐

ERIC-PPCI

Effect of Remote Ischaemic Conditioning on clinical outcomes in ST segment elevation myocardial infarction patients undergoing Primary Percutaneous Coronary Intervention

Trial Protocol Version 5

Sponsored by UCL

Funded by the British Heart Foundation

Protocol Number: Version 5

Title: Effect of Remote Ischaemic Conditioning on clinical outcomes in ST-segment elevation myocardial infarction patients undergoing Primary Percutaneous Coronary Intervention (**ERIC-PPCI**): A multicentre randomised controlled clinical trial

Chief investigator: Prof Derek J Hausenloy
The Hatter Cardiovascular Institute,
University College London,
67 Chenies Mews, London WC1E 6HX, UK.
Tel: 0203 447 9591
Email: d.hausenloy@ucl.ac.uk

Co-Principal investigators: Prof Derek M Yellon, The Hatter Cardiovascular Institute, UCL, London
Prof Rajesh Kharbanda, John Radcliffe Hospital, Oxford
Mr Tim Clayton, London School of Hygiene & Tropical Medicine (LSHTM), London

Location: UK Multicentre

ERIC-PPCI is a collaboration with the **Effect of RIC on Clinical Outcomes in STEMI Patients Undergoing pPCI (CONDI-2)** trial based in Denmark, Serbia and Spain.

Local sites and investigators: Listed on the ERIC-PPCI website: http://ericppci.lshtm.ac.uk/

Sponsor: University College London

Medical contact: Prof Derek J Hausenloy

Clinical Trials Unit: London School of Hygiene and Tropical Medicine

Table of Contents

1. Trial summary 42
 1.1. Protocol summary 42
 1.2. Trial flowchart 44

2. Introduction 45
 2.1. Background 45
 2.2. Scientific rationale 45
 2.3. Research collaboration with Denmark 46

3. Hypothesis 46

4. Endpoints 46
 4.1. Primary endpoint 46
 4.2. Secondary endpoints 46
 4.3. Endpoint Validation 48

5. Power calculations and sample size determination 48
 5.1. Primary combined clinical endpoint 48
 5.2. Biomarker substudy to assess the effect of RIC on MI size (48 hr AUC) 48
 5.3. CMR substudy to assess the effect of RIC on MI size at 6 months 48

6. Selection of patients 49
 6.1. Inclusion criteria 49
 6.2. Exclusion criteria 49
 6.3. Patients entered into observational research 49

7. Ethical considerations 49
 7.1. Consent 49
 7.2. Declaration of Helsinki and UCL Good Clinical Practice 51
 7.3. Ethical committee review 51

8. Randomisation 52
 8.1. Randomisation procedure 52
 8.2. Access to randomisation site 52

9. Blinding 52
 9.1. Unblinded trial staff 52
 9.2. Blinded trial staff 52
 9.3. Emergency unblinding 52

10. Trial treatment 53
 10.1. Remote ischaemic conditioning (RIC) 53
 10.2. Sham RIC 53
 10.3. Initiation of treatment 53
 10.4. Duration of treatment 53

11. Data collection and follow up 54
 11.1. Trial procedures table 54
 11.2. Data collection 55
 11.3. Trial procedures 56
 11.4. Compliance and loss to follow up 57

12. Biomarker substudy 57
 12.1. Procedure 57
 12.2. Analysis methods 57

13. CMR substudy 57
 13.1. CMR substudy endpoints 58

13.2. Exclusion criteria for CMR substudy 58

14. Safety Reporting 59
14.1. Definition 59
14.2. Expected adverse events (recognised to be caused by the RIC stimulus) 59
14.3. Expected serious adverse events related to usual clinical care 59
14.4. Unexpected Serious Adverse Events 59
14.5. Unexpected Non-Serious Adverse Events 60
14.6. Reporting unexpected adverse events 60

15. Withdrawal of patients 60
15.1. Criteria for withdrawal from the trial 60
15.2. Follow up of patients withdrawing from the trial 60
15.3. Reporting withdrawal of patients 61

16. Statistics 61
16.1. Trial statistician 61
16.2. Statistical analysis 61
16.3. Planned subgroup analysis 61
16.4. Procedure to account for missing or spurious data 61

17. Data handling and record keeping 61

18. Insurance 61
18.1. Master Indemnity Agreement 62

19. Publications policy 62

20. Expected value of the results 62

21. Trial organisation 62
21.1. Trial Steering Committee (TSC) 62
21.2. Project Management Group (PMG) 63
21.3. Data Safety and Monitoring Committee (DSMC) 63
21.4. Endpoint Validation Committee (EVC) 63

22. References 64

23. Appendix 1 – CMR protocol 67

24. Appendix 2 – Glossary 68

25. Appendix 3 – EuroQol EQ-5D-5L 72

Appendix 4: Additional Substudies 75

Coronary Physiology substudy 75
Background and rationale 75
Hypothesis 75
Method 75
Sample Size 75
Feasibility 75
References 76

Thrombosis substudy 77
Background 77
Hypothesis 77
Methods 77
Follow-up 78
Handling of results 78
References 78

26. Trial summary

26.1. Protocol summary

Title	**E**ffect of **R**emote **I**schaemic **C**onditioning on clinical outcomes in ST-segment elevation myocardial infarction patients undergoing **P**rimary **P**ercutaneous **C**oronary **I**ntervention (ERIC-PPCI): A multicentre randomised controlled clinical trial.
Sponsor	University College London.
Medical condition or disease under investigation	Acute Myocardial Infarction.
Purpose of clinical trial	To determine whether remote ischaemic conditioning (RIC) improves clinical outcomes in STEMI patients undergoing PPCI.
Trial design	Randomised single blind placebo controlled trial.
Primary objectives	To determine the effect of RIC on cardiac death and hospitalisation for heart failure (HHF) at 12 months in STEMI patients undergoing PPCI.
Secondary objectives	To determine the effects of RIC on:<br>• Rates of cardiac death and HHF at 30 days.<br>• Rates of all-cause death, repeat coronary revascularisation, reinfarction, stroke at 30 days and 12 months.<br>• Quality of life at 6-8 weeks and one year (EQ-5D-5L).<br>• Biomarkers substudy: (400 patients at selected sites) 48 hour area under the curve high-sensitivity troponin T and CK-MB.<br>• CMR Substudy: (250 patients at selected sites) Myocardial infarct size expressed as a percentage of the LV mass by Cardiac MRI at 6 months.<br>• Coronary Physiology Substudy: (180 patients at selected sites) Index of microcirculatory resistance (IMR) during and on completion of PPCI.<br>• Thrombosis Substudy: (all patients recruited at Lister Hospital) Global Thrombosis Test (GTT) and tests of thrombogenesis and fibrinolysis using micro-titre based assays on fresh frozen plasma (FFP).
Sample size	**ERIC-PPCI trial** (UK) 2800 patients undergoing PPCI following STEMI.<br>The trial is a collaboration with the CONDI-2 trial

	(Denmark, Serbia, Spain) which will recruit 2600 patients (NCT01857414).<br><br>In total the sample size is 5400 patients.
Inclusion criteria	5. Onset of STEMI symptoms within 12 hours, lasting for more than 30 minutes<br>6. Age >18 years<br>7. Suspected STEMI (ST-elevation at the J-point in two contiguous leads with the cut-off points: ≥0.2 millivolt (mV) in men or ≥0.15 mV in women in leads V2-V3 and/or ≥0.1 mV in other leads.)<br>8. Eligibility for coronary angiography with follow on PPCI if indicated
Exclusion criteria	8. Previous coronary artery bypass graft surgery<br>9. Myocardial infarction (MI) within the previous 30 days<br>10. Treatment with thrombolysis within the previous 30 days<br>11. Left bundle branch block<br>12. Patients treated with therapeutic hypothermia<br>13. Conditions precluding use of RIC (paresis of upper limb, use of an a-v shunt)<br>14. Life expectancy of less than 1 year due to non-cardiac pathology<br>15. Previous entry into the ERIC-PPCI trial
Trial treatment	The intervention will be applied at a single time point on arrival at the PCI centre, starting prior to the PPCI procedure. The trial treatment may overlap with the start of the PPCI procedure and will not delay PPCI.<br><br>Active treatment (RIC) will consist of four 5 minute inflations of an automated autoRIC™ cuff or manual blood pressure cuff on the upper arm to 200mmHg. For patients presenting with a systolic blood pressure (SBP)≥175mmHg, a manual blood pressure cuff will be used in place of the autoRIC™ cuff. This will be inflated to 25 mmHg above systolic blood pressure. The inflations will be separated by 5 minute periods when the blood pressure cuff will be deflated.<br><br>Control treatment (sham RIC) will consist of placing an identical looking autoRIC™ cuff on the upper arm which is designed to deliver four 5 minute simulated inflations, or will be delivered using a manual blood pressure cuff. The simulated inflations will be separated by 5 minute periods when the blood pressure cuff will remain uninflated.

A glossary of terms and abbreviations used in this protocol is included as Appendix 2.

26.2. **Trial flowchart**

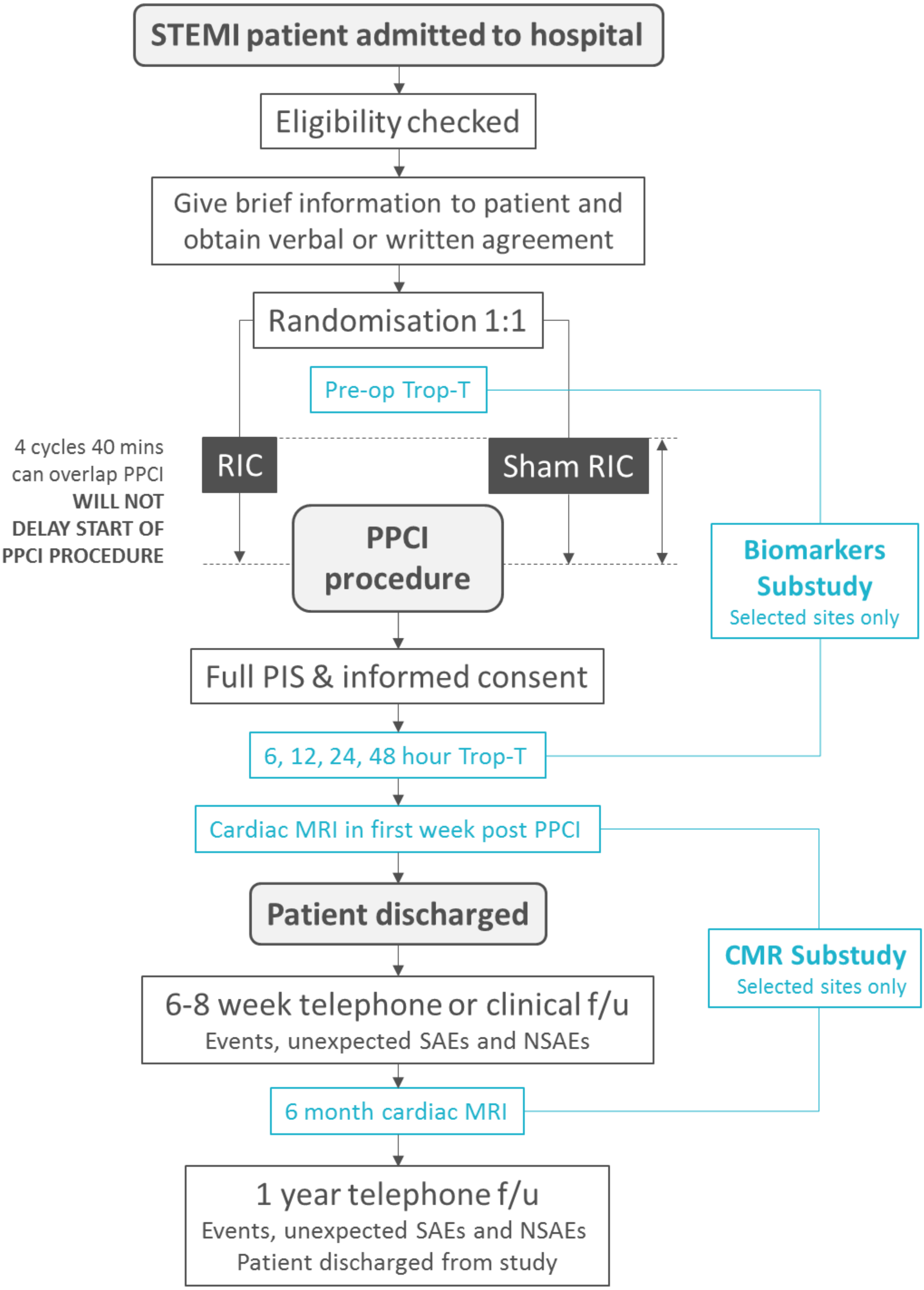

27. Introduction

27.1. Background

Ischaemic heart disease, the leading cause of death in the UK, accounts for 90,000 deaths per year and costs the UK economy nearly £9 billion per annum. Each year 150,000 patients have an acute myocardial infarction (BHF Stats 2010). In patients presenting with an ST-elevation myocardial infarction (STEMI), early myocardial reperfusion using primary percutaneous coronary intervention (PPCI) is the most effective therapeutic intervention for limiting myocardial infarct (MI) size, a major determinant of prognosis post-PPCI. However, despite PPCI, the morbidity and mortality of STEMI patients remain significant, paving the way for novel therapeutic strategies for protecting the heart against acute ischaemia-reperfusion injury (IRI). In this respect remote ischaemic conditioning (RIC) represents a non-invasive and low cost therapeutic strategy for further reducing MI size, preventing the onset of heart failure, and improving clinical outcomes in PPCI patients. For systematic reviews on RIC please see the following references[1, 2].

27.2. Scientific rationale

RIC describes the phenomenon in which the application of multiple cycles of brief non-lethal ischaemia and reperfusion to an organ or tissue remote from the heart protects the myocardium from a lethal sustained episode of acute IRI[1, 2]. The mechanisms underlying RIC are unclear but have been attributed to a neuro-hormonal pathway linking the preconditioned organ or tissue to the heart [2-4]. In 2002, Kharbanda et al[5] first demonstrated that the RIC stimulus could be induced non-invasively in human volunteers by simply inflating and deflating a blood pressure cuff placed on the upper arm. We and others have shown that this RIC stimulus is beneficial in reducing perioperative myocardial injury in the settings of cardiac bypass and abdominal aortic aneurysm surgery[6-9], and reducing periprocedural myocardial and renal injury in the setting of elective PCI[10, 11]. However, several recent studies have reported neutral findings with RIC in these clinical settings[12-15]. The reasons for this are unclear but have been attributed to differences in study design, patient selection and concomitant medication (which are known to interfere with RIC cardioprotection)[16]. Preliminary data suggest that long term outcomes may be improved in RIC treated CABG patients[17], although this needs to be confirmed in prospective large randomised controlled clinical trials such as ERICCA[18] and RIPHeart,[19] which have been prospectively designed and powered to investigate the effect of RIC on long term clinical outcomes in patients undergoing a coronary artery bypass graft operation.

RIC has now been investigated in four small proof-of-concept clinical studies in STEMI patients undergoing PPCI including one by this research group[20-24]. The first study was published by Botker et al (the research collaborator in Denmark)[20, 21] and reported that RIC (four 5 minute upper arm cuff inflations and deflations) administered in the ambulance by paramedics on route to the PPCI centre, significantly increased the mean salvage index from 0·57 in control to 0.69 with RIC at 30 days (as measured by myocardial SPECT). In a post-hoc subgroup analysis of patients presenting with a left anterior descending STEMI, myocardial salvage was increased further, and there was a significant reduction in final MI size and improvement in LV ejection fraction at 30 days[20, 21]. Interestingly, 6 year follow up of this patient cohort revealed less all-cause death in those patients given RIC at the time of their PPCI, although this study has not been prospectively designed to investigate long term endpoints [25]. In another study of 96 patients, Rentoukas et al[22] demonstrated that RIC (three 4 minute upper arm cuff inflations and deflations) administered at the PPCI centre improved ST-segment resolution and reduced MI size when compared to control. Crimi et al [23] have found that RIC administered at the onset of myocardial reperfusion was beneficial in STEMI patients undergoing PPCI.

27.3. Research collaboration with Denmark

The ERIC-PPCI trial will be conducted in collaboration with Prof Hans Erik Bøtker (Aarhus University, Denmark), who is CI of the Effect of RIC on Clinical Outcomes in STEMI Patients Undergoing pPCI (CONDI-2) trial (NCT01857414).

The CONDI 1 trial (CI Hans Bøtker) was the first to successfully demonstrate a beneficial effect of RIC in 142 STEMI patients undergoing PPCI in terms of increased myocardial salvage and smaller MI size [20], effects which were present 6 months later in terms of improved LV systolic function [26]. A €3.0 million research grant has been awarded by the Danish Research Council to investigate the effect of RIC on 12 month clinical outcomes in STEMI patients undergoing PPCI.

The results of ERIC-PPCI and CONDI 2 will be combined in order to maximise power to assess the impact of RIC on clinical endpoints.

The ERIC-PPCI and CONDI 2 trials will each have a separate DSMC and TSC although there will be an opportunity for the these committees to contact each other if required.

A combined and independent Endpoint Validation Committee will review death, HHF, stroke and MI from both trials.

28. Hypothesis

Remote ischaemic conditioning improves long term clinical outcomes (cardiac death and hospitalisation for heart failure) at 12 months in STEMI patients undergoing PPCI.

29. Endpoints

29.1. Primary endpoint

To investigate whether RIC reduces the combined primary endpoint rate of cardiac death and hospitalisation for heart failure (HHF) at 12 months in STEMI patients undergoing PPCI.

29.1.1. Definition for cardiac death

All deaths where there is no clinical or post mortem evidence of a non-cardiac aetiology.

29.1.2. Definition for hospitalisation for heart failure

This will include both heart failure during the index hospitalisation and re-hospitalisation for heart failure. Hospitalisation will be defined as a treatment occurring in hospital. Heart failure will be judged to be present on symptoms (at least one of the following: new or worsening dyspnea, orthopnea, paroxysmal nocturnal dyspnea or increasing fatigue/worsening exercise tolerance) and signs (one of the following: new pulmonary oedema by chest X-ray in the absence of a non-cardiac cause, crepitations believed to be due to pulmonary oedema, and use of loop diuretics to treat presumed pulmonary congestion)[41].

29.2. Secondary endpoints

To investigate whether RIC can affect the following secondary endpoints below:

5. Rates of cardiac death and HHF at 30 days. This data will be collected at the 6-8 weeks post-randomisation follow-up.
6. Rates of all-cause death, repeat coronary revascularisation, reinfarction, stroke at 30 days and 12 months. This data will be collected at the 6-8 weeks and one year post-randomisation).
7. Quality of life at 6-8 weeks and 12 months (EuroQol EQ-5D-5L) (for an example EQ-5D-5L see **Appendix 3**)

Biomarkers substudy (400 patients at selected sites)

8. MI size on 48 hour area-under-the-curve (AUC) hsTrop T and CK-MB

MRI substudy (250 patients at selected sites)

9. MI size on 6 months cardiac MRI (new late gadolinium enhancement expressed as a percentage of the LV mass).
10. Microvascular obstruction on cardiac MRI (hypodense area on late gadolinium enhancement).
11. Myocardial salvage index (AAR [T2 weighted imaging or angiography jeopardy score] subtract final MI size).
12. LV remodelling on 6 month cardiac MRI scan:
    - index LV end-diastolic and end-systolic volumes
    - LV ejection fraction
    - LV mass and wall thickness

Coronary Physiology substudy (180 patients at selected sites)

13. Index of microcirculatory resistance (IMR) during and on completion of PPCI

Thrombosis substudy (all patients recruited at Lister Hospital)

14. Thrombotic status pre and post-PPCI
15. Change in thrombotic status pre and post intervention
16. Differences in ECG ST-resolution and clinical outcomes

Myosin C substudy (24 patients at Barts Heart Centre)

14. MI size on 48 hour area-under-the-curve (AUC) Myosin C

29.2.1. Definition of stroke

Stroke is defined as a focal, central neurological deficit of cerebrovascular cause lasting more than 24 hours or is interrupted by death within 24 hours. Definition of reinfarction

Reinfarction is defined as an acute MI that occurs within 28 days of an incident or recurrent MI. The ECG diagnosis of suspected reinfarction following the initial MI may be confounded by the initial evolutionary ECG changes. Reinfarction should be considered when ST elevation >0.1 mV recurs, or new pathognomonic Q waves appear, in at least two contiguous leads, particularly when associated with ischaemic symptoms for 20 min or longer. Re-elevation of the ST-segment can, however, also be seen in threatened myocardial rupture and should lead to additional diagnostic workup. ST depression or LBBB alone are nonspecific findings and should not be used to diagnose reinfarction.

In patients in whom reinfarction is suspected from clinical signs or symptoms following the initial MI, an immediate measurement of cTn is recommended. A second sample should be obtained 3–6 h later. If the cTn concentration is elevated, but stable or decreasing at the time of suspected reinfarction, the diagnosis of reinfarction requires a 20% or greater increase of the cTn value in the second sample. If the initial cTn concentration is normal, the criteria for new acute MI apply.

29.2.2. Definition of coronary revascularisation

Revascularisation is defined as coronary revascularisation by PCI or CABG following index PPCI, excluding staged PPCI.

29.3. Endpoint Validation

The primary combined endpoint, stroke, MI, reinfarction and revascularisation will be validated by an independent combined event validation committee (EVC), which will validate death, HHF, MI and strokes occurring in both ERIC-PPCI and CONDI 2. The EVC will be blinded to the randomised treatment allocation.

30. Power calculations and sample size determination

30.1. Primary combined clinical endpoint

The primary combined endpoint will be cardiac death and HHF at 12 months. These endpoints have been selected as they are the most relevant clinical endpoints that are likely to be affected by RIC, an intervention which protects cardiomyocytes against acute IRI. According to the UK NIAP 2008 database, cardiac mortality at 12 months ranged from 5.8%-16.7% depending on the call to balloon time, with the overall 12 month death rate of 8.7% for all PPCI patients. In a recent Danish clinical study post-PPCI, the one year mortality was 9.4% and the cumulative risk of readmission with heart failure was 8% [30]. In another, non-UK based, clinical study, the incidence of HHF was 12.7% at 12 months post-PPCI [31]. We have based our power calculations on these published studies, accounting for the marked improvements in clinical outcomes in the contemporary era by using much more conservative event rates.

As a conservative estimate we will use a combined cardiac death and HHF event rate of 8.5% at 12 months for all-comer STEMI patients. In the combined ERIC-PPCI / CONDI 2 trial we estimate the effect size to be a 25% relative reduction in the event rate. The rationale for this is based on proof-of-concept clinical studies in which RIC, and related therapeutic interventions such as ischaemic postconditioning, have reported 40-50% reductions in MI size[32]. To demonstrate a 25% reduction in the primary composite endpoint in the RIC-treated group (from 8.5% to 6.4%), with 80% power and at the 5% significance level, will require 2395 patients per treatment arm which equates to 4790 patients in total. Therefore, we will need to recruit 5400 STEMI patients (allowing for a 10% drop out rate at 12 months) between UK and Denmark (2800 STEMI patients in the UK and 2600 STEMI patients in Denmark).In the ERIC-PPCI trial we intend to recruit 2800 STEMI patients in the UK through 30 PPCI sites.

If the event rate in the control arm is higher than the estimated 8.5% or the losses less than 10% then the power will increase. The ERIC-PPCI UK arm of the trial of 2800 patients alone would provide in excess of 80% power to detect a relative reduction of 35% from 8.5% to 5.5% allowing for 10% losses to follow up.

30.2. Biomarker substudy to assess the effect of RIC on MI size (48 hr AUC)

A major secondary endpoint of the ERIC-PPCI study will be MI size as measured by 48 hr AUC high-sensitive Troponin T and CK-MB. To date the effect of RIC on this specific endpoint has not been investigated. Using 72 hr AUC Trop I, Thibault et al [32] demonstrated that ischaemic postconditioning reduced MI size from 24.6 (SD 20.6) $x10^4$ to 13.0 (SD 7.0) $x10^4$ IU/L. This equates to a relative reduction in MI size of 47%. In order to demonstrate a more conservative 25% relative reduction in MI size with RIC in PPCI patients from 24.6 (SD 20.6) $x10^4$ using 48 hr AUC hsTrop T with 80% power and at the 5% significance level, will require 177 patients in each treatment group or 354 patients in total. Allowing for 10% dropout rate would require 400 patients in total.

In view of the clear positive skewness of the Troponin AUC it is anticipated that this outcome will be analysed on the log scale. This will lead to estimation of the relative reduction in the MI size, which is expected to be a more precise estimate of any treatment effect. Therefore, the planned sample size of 400 patients is likely to provide greater than 80% power and allow for larger than a 10% loss in outcomes due to incomplete Troponin AUCs.

30.3. CMR substudy to assess the effect of RIC on MI size at 6 months

The CMR substudy primary endpoint is MI size (mass of late gadolinium enhancement) expressed as percentage of LV mass at 6 months. No clinical studies have previously used cardiac MRI to assess the effect of RIC on MI size. Using myocardial SPECT, Botker et al [20] were unable to demonstrate a significant reduction in MI size at 30

days in all STEMI patients. Lonborg et al [33] used CMR to demonstrate that ischaemic postconditioning reduced MI size at 3 months post-PPCI from 17±8% to 14±7% (mass of infarct expressed as a % of the LV mass). This equates to a relative reduction in MI size of 17.6% (equivalent to an absolute reduction of 3%). Based on this data, to demonstrate a similar relative reduction in MI size with RIC in PPCI patients from 17% (SD 8%) in the 6 month CMR scan, with 80% power and at the 5% significance level, will require approximately 112 patients in each treatment group or 224 patients in total. To allow for a 10% dropout rate we plan to recruit 250 patients in total.

31. Selection of patients

31.1. Inclusion criteria

5. Onset of STEMI symptoms within 12 hours, lasting for more than 30 minutes
6. Patients older than 18 years
7. Suspected STEMI (ST-elevation at the J-point in two contiguous leads with the cut-off points: ≥0.2 millivolt (mV) in men or ≥0.15 mV in women in leads V2-V3 and/or ≥0.1 mV in other leads)
8. Eligibility for coronary angiography with follow on PPCI if indicated

31.2. Exclusion criteria

8. Previous coronary artery bypass graft surgery
9. Myocardial infarction (MI) within the previous 30 days
10. Treatment with thrombolysis within the previous 30 days
11. Left bundle branch block (LBBB)
12. Patients treated with therapeutic hypothermia
13. Conditions precluding use of RIC (paresis of upper limb, use of an a-v shunt)
14. Life expectancy of less than 1 year due to non-cardiac pathology
15. Previous entry into the ERIC-PPCI trial

For centres recruiting into CMR substudy only: Please refer to section 13 as additional exclusion criteria apply to substudy patients.

31.3. Patients entered into observational research

Patients may be entered into registries or observational studies while also participating in ERIC-PPCI.

32. Ethical considerations

32.1. Consent

As patients admitted with a STEMI will need to receive urgent treatment, randomisation and the trial intervention will need to be started as early as possible to ensure that there is no delay to the PPCI procedure. The physical, mental and emotional state of patients may also be affected due to their MI and the use of analgesic drugs in pain management, which could impair their decision making capacity. Therefore, the consent process in this situation requires careful consideration bearing in mind applicable regulatory requirements, adherence to ICH-GCP and the requirements in the Declaration of Helskini.

32.1.1. Patient agreement

The patient will be approached at the time that their STEMI is confirmed and information about the trial should be provided to the patient to their level of capacity. A patient leaflet is provided for this purpose. The patient leaflet may be signed by the patient if they agree to participate, however verbal assent is the only requirement. If the patient cannot give verbal assent they must not be entered into the trial.

Once agreement is obtained the patient may be entered into the trial. Agreement to take part in the trial should be recorded in the patient notes. A signed patient leaflet is not mandatory and will not be considered as equivalent to informed consent.

32.1.2. Personal Consultee

When during the PPCI procedure a personal consultee is present:

A friend or relative's advice should be taken on whether they think the patient would wish to be part of the trial, particularly if the patient had expressed any prior wishes or advanced decisions. A consultee declaration form should be completed. Once the patient has recovered they should still be approached to give informed consent regardless of whether a consultee declaration form has been completed.

When a patient has agreed but does not regain capacity to give informed consent:

After the PPCI procedure a friend or relative's advice should be taken on whether they think the patient would wish to be part of the trial, particularly if the patient had expressed any prior wishes or advanced decisions. A consultee declaration form should be completed.

32.1.3. Informed consent

As soon as the patient has recovered after the procedure the patient should be given sufficient time to consider the trial and ask questions, following which informed consent will be taken. If the patient elects to withdraw from the trial at this point then we will seek consent to use the data and samples already acquired.

32.1.4. Withdrawal

A patient may decide to withdraw from the trial at any time without prejudice to their future care.

32.1.5. Patients not surviving the PPCI procedure

Written consent will not be available for those patients that die during the PPCI procedure. Permission has been granted by the Confidentiality Advisory Group (CAG) to allow us to use non-identifiable information gathered up to that point in the trial. The CAG reference number is 15/CAG/0150.

32.1.6. Consent flowchart

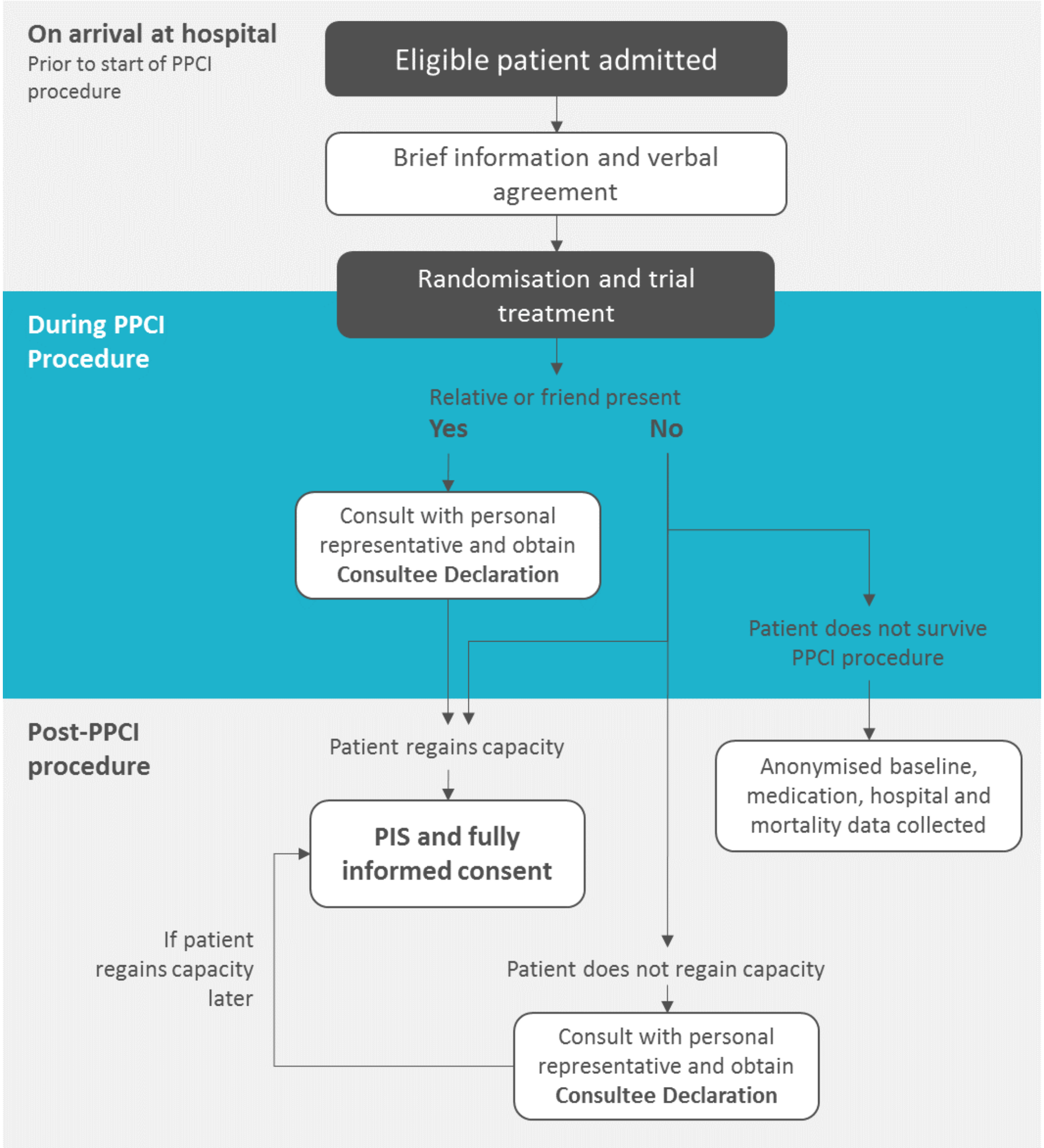

32.2. Declaration of Helsinki and UCL Good Clinical Practice

The trial will conform to the spirit and the letter of the declaration of Helsinki, and in accordance with the UCL Good Clinical Practice Guidelines.

32.3. Ethical committee review

NRES Committee London-Harrow have reviewed and approved the trial. The REC number is 15/LO/0217. Copies of the letters of approval will be filed in the trial site files at each centre.

33. Randomisation

33.1. Randomisation procedure

Patients will be randomised to either RIC or sham RIC by a designated research investigator, this investigator will therefore be unblinded to treatment allocation. Randomisation will be coordinated centrally by the LSHTM CTU via a secure website www.sealedenvelope.com and will be stratified by centre using random permuted blocks.

33.2. Access to randomisation site

Access to the randomisation website will be strictly controlled at each site and limited to unblinded research investigators delegated by the PI to be responsible for performing either the RIC or sham RIC protocol.

Patients will be randomised to receive either **RIC** or **sham RIC**.

34. Blinding

34.1. Unblinded trial staff

At each site specific staff will be delegated by the PI to perform the randomisation and intervention procedures. These staff will be the only people in each centre aware of the treatment allocation for the patient and will not be involved with data collection other than any relating to the randomisation and intervention procedures.

At the LSHTM clinical trials unit the unblinded trial statistician and the data managers will have access to allocation data.

34.2. Blinded trial staff

The patients and the research doctor/nurse collecting the data and following up the patient will be blinded to the treatment allocation. Outcome assessments will be blinded. The event validation committee will be blinded to treatment allocation.

In the CMR substudy the research fellow analysing the CMR scan will be blinded to the treatment allocation.

34.3. Emergency unblinding

The benign and short term nature of the intervention makes the need for emergency unblinding unlikely, if required patients will be able to be unblinded through the randomisation website.

35. Trial treatment

Patients will be randomised on arrival at the hospital and the unblinded research investigator will then deliver either the RIC or sham RIC protocol.

Automated CellAegis autoRIC™ cuff devices www.cellaegisdevices.com will be supplied to deliver the RIC and sham RIC protocols. A manual cuff (sphygmomanometer) will also be supplied to each site, as backup for the autoRIC™ device and to deliver the trial treatment for patients with a systolic blood pressure (SBP) ≥175mmHg.

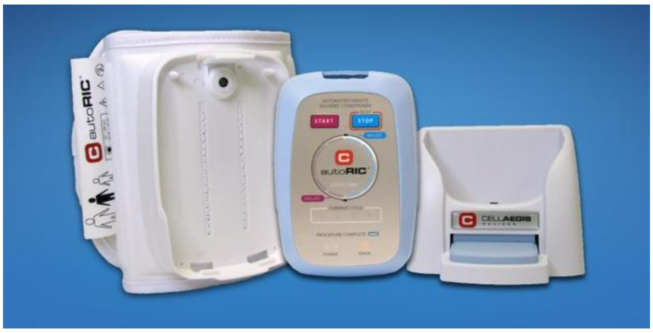

AutoRIC™ devices are used in conjunction with single use disposable inner cuffs. An autoRIC™ device, an autoRIC™ sham device and disposable cuffs will be provided to the participating sites.

The advantage of the autoRIC™ device is that once it has been placed on the upper arm, the preprogrammed standard RIC or sham RIC protocol is simply delivered by pressing a single start button.

35.1. Remote ischaemic conditioning (RIC)

An automated autoRIC™ cuff, or manual cuff, will be placed on the upper arm and inflated to 200mmHg for 5 minutes and then deflated for 5 minutes, a cycle which will be undertaken 4 times in total. For patients presenting with SBP ≥175mmHg, a manual blood pressure cuff will be used and inflated to 25 mmHg above systolic blood pressure.

35.2. Sham RIC

An autoRIC™ cuff visually identical to that used in the RIC protocol, or manual cuff, will be placed on the upper arm and a simulated RIC protocol applied. Inflation will be simulated and held for 5 minutes, deflation will then be simulated and held for five minutes, a cycle which will be undertaken 4 times in total.

The sham device's components and external appearance are identical to that of the autoRIC™. However, as compared to the autoRIC™, the sham device control unit's pump is disconnected such that the control unit cannot inflate the applicator cuff. The sham device provides the same sound and vibration as that of the pump inflating and the same LED indicators on the control unit. The operation of the autoRIC™ control unit with respect to the (simulated) RIC procedure initiation, cycle indication, and termination are identical in the sham device, with the exception that the applicator cuff does not inflate.

35.3. Initiation of treatment

The trial treatment should be started with sufficient time to ensure that as many cycles of ischaemia and reperfusion as possible are completed before the onset of reperfusion.

35.4. Duration of treatment

Although the RIC / sham RIC protocol lasts 40 minutes in total, the cuff should be removed after the 4th cycle of inflation and the last 5 min of reperfusion undertaken with the cuff removed. This means that after 35 min the intervention is completed. In the cases where the door to PPCI time is less than 35 min, the RIC / sham RIC protocol may overlap with the beginning of the PPCI procedure when the coronary angiogram is being performed.

Under no circumstances should the RIC protocol delay the onset of the PPCI procedure.

36. Data collection and follow up

36.1. Trial procedures table

	pre-PPCI	PPCI	Post-PPCI in hospital					After discharge		
		0 hrs	6 hrs	12 hrs	24 hrs	48 hrs	2-7 days	6-8 weeks	6 months	12 months
**Clinical assessments**										
Review of eligibility criteria	X									
Patient agreement	X									
History and examination	X									
PIS & Informed consent			**Prior to patient discharge**							
**Trial intervention**										
Randomisation	X									
RIC / Sham RIC*	X (May overlap with PPCI)									
**Clinical outcomes**										
Death		X	X					X		X
HHF			X					X		X
MI		X	X					X		X
Revascularisation			X					X		X
Stroke		X	X					X		X
**Quality of life**										
EQ-5D-5L								X		X
**Safety reporting**										
SAE / NSAEs		X	X					X		X
**Substudies (at selected sites only)**										
Biomarker substudy: Trop-T and CK-MB		X	X	X	X	X				
CMR substudy: Cardiac MRI							X		X	
Coronary Physiology substudy: IMR		X								
Thrombosis substudy (Lister Hospital only)		X			X	2-3 days post-randomisation		X		

* The RIC/Sham RIC may overlap with the PPCI procedure and should not delay the start of PPCI

36.2. Data collection

All patients will have a full medical history taken and various clinical examinations as part of usual care. The following are to be recorded on the trial CRF:

- Weight and height (BMI will be calculated automatically when entered on the eCRF)
- Blood Pressure
- Gender
- Ethnicity
- Date of birth
- Medical history:
  - Known Diabetes Mellitus
  - Hypercholesterolaemia
  - Hypertension
  - Previous myocardial infarction
  - Previous PCI
  - Previous CABG
  - Previous stroke
  - Atrial fibrillation
  - Peripheral arterial disease
  - Smoking history
  - Family history of IHD
  - History of renal disease
  - Other cardiac disease
- NYHA class
- Ejection fraction post procedure
- Analgesia use
- Medication at admission and discharge:
  - Antiplatelets
  - β-blockers
  - ACE inhibitors
  - Angiotension receptor blockers
  - Calcium channel blockers
  - Digoxin
  - Anti-diabetic drugs
  - Lipid lowering drugs
  - Anticoagulants
  - Diuretics
  - Antianginal drugs
- ECG at admission and prior to discharge
- Call to balloon time, door to balloon time and symptoms to balloon time
- Angiographic data (TIMI flow pre and post-PPCI)
- Use of thrombectomy
- Details of the PPCI procedure
- Procedural drugs
- NHS number. Mortality data will be tracked up to 10 years after randomisation.
- GCS on admission
- Killip Class

- Out of hospital cardiac arrest
- Ventilated pre procedure
- Arterial gas taken
- Creatinine

36.3. Trial procedures

On admission

- Review of eligibility criteria
- Provide information to patient's level of capacity
- Patient agreement (please refer to section 7.1)
- Randomisation
- Randomly allocated trial intervention – RIC or sham RIC
- Baseline Troponin-T/CK-MB
- Thrombosis substudy only: baseline point-of-care Global Thrombosis Test (GTT)
- Myosin C substudy only: baseline Myosin C

Post-PPCI

- Full Patient Information Sheet
- Informed consent
- Biomarker substudy only: 6, 12, 24 and 48 hour Troponin-T/CK-MB
- CMR substudy only: 2-7 day cardiac MRI
- Coronary Physiology substudy: IMR before and after stent placement
- Thrombosis substudy only: 24 and 48-72 hour point-of-care GTT
- Myosin C substudy only: 6, 12, 24 and 48 hour Myosin C
- Events
  - Death
  - Heart failure during the index hospitalisation
  - MI
  - Stroke
- Unexpected SAEs and NSAEs
- Bleeding
- Cardiac medication

6-8 weeks post-randomisation (telephone or outpatient follow-up)

- Events
  - Death
  - Hospitalisation for heart failure
  - MI
  - Stroke
  - Revascularisation
- Quality of life (EQ-5D-5L)
- Unexpected SAEs and NSAEs
- Device implantation
- Ejection fraction (if known)
- Cardiac medication
- Thrombosis substudy only: 6-8 week point-of-care GTT

6 months post-randomisation

- CMR Substudy only: 6 month cardiac MRI (outpatient appointment)

12 months post-randomisation (telephone follow up)

- Events
  - Death
  - Hospitalisation for heart failure
  - MI
  - Stroke
  - Revascularisation
- Quality of life (EQ-5D-5L)
- Unexpected SAEs and NSAEs
- Device implantation

36.4. Compliance and loss to follow up

Problems with compliance are expected to be rare given that the intervention is non-invasive in nature and is administered at a single time point.

Patients are free to withdraw from the trial at any time without prejudice to their future care. Loss to follow up is expected to be rare as the trial is designed to be minimally disruptive to participants. Data collected up to the point of withdrawal will be used unless the patient specifically requests that it is not.

The patients will be followed up at 6-8 weeks and 12 months after PPCI, in order to determine endpoints contributing to the primary endpoint (cardiac death and hospitalisation for heart failure).

These follow ups will either be planned to coincide with existing clinical appointments or will otherwise be conducted by telephone. A non-compliance and dropout rate of 15% has been accounted for in the sample size.

37. Biomarker substudy

MI size as measured as the 48 hour AUC serum level of high-sensitive Troponin-T (hsTropT) and CK-MB will be analysed in 400 patients. After the data has been acquired for the MI size in 400 patients a prespecified interim analysis will be performed.

37.1. Procedure

From each patient, a single blood sample will be taken at each of the 5 timepoints (0, 6, 12, 24 and 48 hours following PPCI procedure). Each blood sample will be analysed at the local hospital.

37.2. Analysis methods

Quantitative serum hsTropT measurement will be performed using a one step immunoassay based on electrochemiluminescence technology (Elecsys 2010, Roche, Switzerland). The reference range will be ≤14 ng/L (14 ng/L is the 99th centile of reference population with cardiovascular risk of <10%).

Quantitative serum CK-MB measurement will be performed using a standard immunoassay method.

38. CMR substudy

The CMR substudy will recruit 250 PPCI patients through a selection of PPCI centres with facilities for performing CMR scans in PPCI patients. All sites performing CMR for the ERIC-PPCI study will have a Siemens or Philips 1.5 T scanner and will use a standardised CMR protocol which is included in this protocol as appendix 1.

Training in the CMR protocol will be provided to each recruiting site by the ERIC-PPCI research fellow. Each patient will receive two CMR scans, the first performed within the week following the PPCI procedure and the

second at 6 months. All CMR scans will be analysed at a central CMR core lab which will be staffed by an independently funded senior CMR clinical fellow.

The ERIC-PPCI CMR substudy will also put the research infrastructure in place for future PPCI/CMR clinical studies in the UK.

38.1. CMR substudy endpoints

The primary endpoint of the CMR substudy will be MI size on the 6 month CMR scan (measured in mass of late gadolinium enhancement and expressed as a percentage of the LV mass).

Several other CMR parameters will be collected as follows:

38.1.1. The acute post-PPCI CMR scan

17. Left ventricle (LV) ejection fraction and indexed LV end systolic and diastolic volumes and mass using short axis SSFP cine imaging.
18. MI size measured by the mass of late gadolinium enhancement (20 min after administration of contrast) of cardiac MRI scan expressed as a percentage of LV mass.
19. Area at risk (AAR) measured as the increase in T2 values using a Siemens T2 mapping sequence, which has been validated against conventional measures of area at risk [35-37]. The AAR will also be estimated using the modified BARI and APPROACH angiography scores [38].
20. Myocardial salvage index = AAR subtract MI size/AAR. The myocardial salvage index (using T2-weighted CMR and late gadolinium enhancement) has been demonstrated to predict prognosis post-PPCI [39].
21. The incidence and extent of microvascular obstruction (hypo-enhancement on late gadolinium enhancement 20 min after administration of contrast).
22. The incidence and extent of intramyocardial haemorrhage (hypo-enhancement on Siemens T2* mapping sequence)[40].

38.1.2. The follow up CMR scan 6 months post-PPCI

23. LV ejection fraction and indexed LV end systolic and diastolic volumes and mass.
24. MI size measured by the mass of late gadolinium enhancement.

38.2. Exclusion criteria for CMR substudy

Known contraindication to cardiac magnetic resonance imaging (MRI) such as:

25. Significant claustrophobia
26. Severe allergy to gadolinium chelate contrast
27. Severe renal insufficiency (defined as estimated glomerular filtration rate [eGFR] <30 mL/min/1.73 m2)
28. Presence of MRI contraindicated implanted devices (e.g. pacemaker, implanted cardiac defibrillator, cardiac resynchronisation therapy device, cochlear implant)
29. Embedded metal objects (e.g., shrapnel)
30. Any other contraindication for cardiac MRI

Patients may still enroll in the main trial without enrolling in the CMR substudy.

39. Safety Reporting

39.1. Definition

Unexpected events that have not been defined as endpoints (section 4), expected complications of the RIC stimulus or expected complications of usual clinical care (section 14.3) should be reported as either an SAE or NSAE, depending on their severity. Safety reporting for each patient should commence from time of randomisation to completion of follow up at one year after the PPCI procedure.

39.2. Expected adverse events (recognised to be caused by the RIC stimulus)

The benign nature of the RIC stimulus excludes there being any expected serious adverse events. The following are expected non-serious events in response to the RIC stimulus and will be recorded on the Case Report Form. They do not need to be reported to the Clinical Trials Unit.

2. Skin petechiae caused by cuff inflation

39.3. Expected serious adverse events related to usual clinical care

These events are recognised complications of PPCI. They will be recorded on the Case Report Form but do not need to be reported separately on an SAE form:

31. Death
32. Acute renal failure which may require haemodialysis, peritoneal dialysis, or haemofiltration
33. Ventricular tachycardia of fibrillation requiring direct-current (DC) cardioversion
34. Significant heart block requiring temporary or permanent cardiac pacing
35. Tamponade requiring urgent surgical intervention
36. Cardiogenic shock requiring intra-aortic balloon pump or other assist devices

The following events are recognised complications of routine clinical care and for the purposes of this trial will not be designated as SAEs. They do not need to be reported:

37. Atrial fibrillation
38. Acute mitral valve cordal rupture or ventricular septal rupture requiring surgical intervention
39. Persistent complete heart block requiring permanent pacemaker implantation
40. Aspiration pneumonia following VF arrest
41. Rib fracture following chest compression

39.4. Unexpected Serious Adverse Events

Any untoward medical occurrence/effect that:

42. Results in death
43. Is life-threatening*
44. Requires hospitalisation or prolongation of existing inpatient's hospitalisation
45. Results in persistent or significant disability or incapacity

**Life-threatening* in the definition of a serious adverse event refers to an event in which the patient was at risk of death at the time of event; it does not refer to an event which hypothetically might have caused death if it were more severe.

SAEs should be reported to the Clinical Trials Unit within 7 days. The report should include an assessment of causality by the Principal Investigator at each site (see section 14.6.2). The Chief Investigator will be responsible

for the prompt notification of findings that could adversely affect the health of patients or impact on the conduct of the trial. Notification of confirmed unexpected and related SAEs will be to the Sponsor, the Research Ethics Committee and the Data and Safety Monitoring Committee (DSMC).

39.5. Unexpected Non-Serious Adverse Events

Unexpected non-serious adverse events should be evaluated by the Principal Investigator or research nurse. This should include an assessment of causality (see section 14.6.2) and intensity (see section 14.6.1) and reports made within 14 days. The Clinical Trials Unit will keep detailed records of all unexpected adverse events reported. Reports will be reviewed by the Chief Investigator to consider intensity, causality and expectedness. As appropriate these will be reported to the sponsor, the DSMC and the Ethics Committee.

39.6. Reporting unexpected adverse events

Investigators will make their reports of all unexpected adverse events, whether serious or not, to the Clinical Trials Unit, London School of Hygiene and Tropical Medicine.

39.6.1. Assessment of intensity

Mild: The patient is aware of the event or symptom, but the event or symptom is easily tolerated.

Moderate: The patient experiences sufficient discomfort to interfere with or reduce his or her usual level of activity.

Severe: Significant impairment of functioning; the patient is unable to carry out usual activities and/or the patient's life is at risk from the event.

39.6.2. Assessment of causality

Probable: A causal relationship is clinically / biologically highly plausible and there is a plausible time sequence between onset of the adverse event and the RIC procedure.

Possible: A causal relationship is clinically / biologically plausible and there is a plausible time sequence between onset of the adverse event and the RIC procedure.

Unlikely: A causal relationship is improbable and another documented cause of the adverse event is most plausible.

Unrelated: A causal relationship can definitely be excluded and another documented cause of the adverse event is most plausible.

40. Withdrawal of patients

40.1. Criteria for withdrawal from the trial

A patient may decide to withdraw from the trial at any time without prejudice to their future care. Withdrawal will be uncommon, because of the non-invasive nature of the planned intervention and the follow up which will be integrated within routine clinical care wherever possible. We have allowed in our sample size calculation for a non-compliance and drop out rate of up to 15% although it is expected to be lower than this.

40.2. Follow up of patients withdrawing from the trial

Patients who are randomised but withdraw before the intervention will undergo standard clinical care according to local protocols. If patients undergo the intervention but subsequently withdraw, they will undergo standard clinical care. Patients will be encouraged to allow data and samples that have been collected before withdrawal to be used in the analyses. However, if consent to use data/samples is also withdrawn, then these will be discarded. Patients withdrawing from the trial will continue to be followed up by their local team. There should be no need for further follow up from the research team.

40.3. Reporting withdrawal of patients

The Clinical Trials Unit at LSHTM should be informed by email if a patient has withdrawn from the trial. A withdrawal from will be completed on the trial eCRF.

41. Statistics

41.1. Trial statistician

Statistical analysis will be coordinated from the Clinical Trials Unit at London School of Hygiene and Tropical Medicine.

41.2. Statistical analysis

A detailed statistical analysis plan will be produced prior to unblinding of any data. The primary analysis will be a comparison of the cardiac death or HHF event rate one year after randomisation between the RIC and sham RIC arms of the trial amongst all STEMI patients. Hazard ratios and confidence intervals will be calculated using Cox proportional hazards modelling and Kaplan-Meier curves will be produced. In addition risk differences at one year will also be calculated together with 95% confidence intervals. The results for the individual components of the primary endpoint will also be presented together with other time to event secondary endpoints such as cardiac death or HHF at 30 days. Differences in means (continuous variables) together with 95% confidence intervals will be calculated using linear regression models and analysis of covariance techniques where appropriate. The primary analysis will be performed on an intention to treat basis i.e. by including all patients where possible according to the group to which they were randomised irrespective of whether they received the intervention as allocated. A secondary per protocol analysis will be undertaken including only patients who receive the allocated intervention as intended.

41.3. Planned subgroup analysis

We plan to undertake a limited number of pre specified subgroup analyses: these will be expected to include diabetes, LAD vs non-LAD STEMI, TIMI flow and time of onset of chest pain to PPCI. The subgroup analyses will be detailed in the statistical analysis plan.

41.4. Procedure to account for missing or spurious data

All patients randomised to the trial will be analysed on an intention to treat basis. Data will be validated and the data analysis will take appropriate account of missing values. This process will be detailed in the statistical analysis plan.

42. Data handling and record keeping

Data will be entered onto an online database and stored securely on Rackspace servers; http://www.rackspace.co.uk and managed by Sealed Envelope™. Data will be kept for 15 years following completion of the trial. The data controller for the trial is the Chief Investigator (UCLH are the data controller's organisation) and the data processor is London School of Hygiene and Tropical Medicine.

43. Insurance

All recruiting centres will be covered by NHS indemnity for negligent harm providing researchers hold a contract of employment with the NHS, including honorary contracts held by academic staff. Medical co-investigators will also be covered by their own medical defence insurance for non-negligent harm.

43.1. Master Indemnity Agreement

The CellAegis autoRIC^{TM} devices are covered by CellAegis for public and product indemnity. CellAegis is registered with the Department of Health under the Master Indemnity Agreement (MIA) reference number IFA2312.

44. Publications policy

It is our intention to disseminate the results of the trial as widely as possible. This is likely to be through a publication in a peer reviewed journal, and through presentations at National and International Cardiology conferences. Publications will follow the CONSORT guidelines. Authorship will follow international guidelines.

45. Expected value of the results

There is an urgent need to improve clinical outcomes in STEMI patients undergoing PPCI. If ERIC-PPCI demonstrates reduced major adverse cardiac events at 12 months in patients treated with RIC at the time of PPCI, there is the potential to change the current management of PPCI patients, to a non-invasive, non-pharmacological, and cost effective therapeutic strategy with benefits in both patient survival and for the prevention of heart failure.

46. Trial organisation

46.1. Trial Steering Committee (TSC)

The TSC will meet every 6 months. The TSC will be responsible for drafting the final report and submission for publication.

Dr Rob Henderson - chair (Independent Interventional Cardiologist)

Prof Derek J Hausenloy (Chief Investigator)

Associate Prof Tim Clayton (Co-Principal Investigator/Senior Medical Statistician with CTU)

Prof Derek Yellon (Co-Principal Investigator)

Prof Rod Stables (Independent Interventional Cardiologist)

Prof Simon Redwood (Co-applicant/Interventional Cardiologist)

Prof Michael Marber (Independent Cardiologist)

Mrs Rosemary Knight (Senior Manager of the Clinical Trials Unit)

Prof Rajesh Kharbanda (Co-Principal Investigator/Interventional Cardiologist)

Mr Paul Hambley (previous PPCI patient)

Mr Alan Berry (previous PPCI patient)

Prof Hans Erik Botker (Interventional Cardiologist and Chief Investigator for Danish CONDI 2 trial)

Observers:

Dr Shannon Amoils (BHF representative)

Ms Tabitha Kavoi (Sponsor representative)

Mr Alexander Perkins (Trial Manager)

Mr Richard Evans (Senior Manager of the Clinical Trials Unit)

46.2. Project Management Group (PMG)

Prof Derek J Hausenloy (Chief Investigator)

Prof Rajesh Kharbanda (Co-Principal Investigator/Interventional Cardiologist)

Prof Derek Yellon (Co-Principal Investigator)

Associate Prof Tim Clayton (Co-Principal Investigator/Senior Medical Statistician)

Mr Alexander Perkins (Trial Manager)

Mr Richard Evans (Senior Manager of the Clinical Trials Unit)

Mr Matthew Dodd (Data Manager)

Mrs Rosemary Knight (Senior Manager of the Clinical Trials Unit)

Dr Manish Ramlall (Clinical Research Fellow)

46.3. Data Safety and Monitoring Committee (DSMC)

Prof Colin Berry - **Chair** (independent interventional cardiologist)

Prof Tom Meade (Emeritus Professor of Epidemiology)

Dr Andrew Copas (independent statistician)

Dr Jennifer Nicholas (unblinded statistician at the CTU) will support the DSMC

The DSMC will meet periodically to determine whether there are any unforeseen effects of RIC.

46.4. Endpoint Validation Committee (EVC)

The EVC will meet periodically to validate and adjudicate primary endpoints.

<u>ERIC-PPCI Endpoint Validation Committee</u>

Dr Andrew Ludman (Independent cardiologist)

Dr Nick Cruden (Independent cardiologist)

<u>Combined Adjudication Team</u>
Prof Kristian Thygesen (Chair)
Dr Saqib Chowdhary (Independent cardiologist)
Dr Jesús María de la Hera Galarza (Independent cardiologist)
Final member to be decided

47. References

(1) Przyklenk K, Bauer B, Ovize M, Kloner RA, Whittaker P. Regional ischemic 'preconditioning' protects remote virgin myocardium from subsequent sustained coronary occlusion. *Circulation* 1993;87(3):893-9.

(2) Hausenloy DJ, Yellon DM. Remote ischaemic preconditioning: underlying mechanisms and clinical application. *Cardiovasc Res* 1-8-2008;79(3):377-86.

(3) Lim SY, Yellon DM, Hausenloy DJ. **The neural and humoral pathways in remote limb ischemic preconditioning**. *Basic Res Cardiol* 2010;105(5):651-5.

(4) Redington KL, Disenhouse T, Strantzas SC et al. Remote cardioprotection by direct peripheral nerve stimulation and topical capsaicin is mediated by circulating humoral factors. *Basic Res Cardiol* 2012;107(2):1-10.

(5) Kharbanda RK, Mortensen UM, White PA et al. **Transient limb ischemia induces remote ischemic preconditioning in vivo**. *Circulation* 3-12-2002;106(23):2881-3.

(6) Hausenloy DJ, Mwamure PK, Venugopal V et al. Effect of remote ischaemic preconditioning on myocardial injury in patients undergoing coronary artery bypass graft surgery: a randomised controlled trial. *Lancet* 18-8-2007;370(9587):575-9.

(7) Venugopal V, Hausenloy DJ, Ludman A et al. Remote ischaemic preconditioning reduces myocardial injury in patients undergoing cardiac surgery with cold-blood cardioplegia: a randomised controlled trial. *Heart* 2009;95(19):1567-71.

(8) Thielmann M, Kottenberg E, Boengler K et al. Remote ischemic preconditioning reduces myocardial injury after coronary artery bypass surgery with crystalloid cardioplegic arrest. *Basic Res Cardiol* 2010;105(5):657-64.

(9) Ali ZA, Callaghan CJ, Lim E et al. Remote ischemic preconditioning reduces myocardial and renal injury after elective abdominal aortic aneurysm repair: a randomized controlled trial. *Circulation* 11-9-2007;116(11 Suppl):I98-105.

(10) Hoole S, Heck PM, Sharples L et al. Cardiac Remote Ischemic Preconditioning in Coronary Stenting (CRISP Stent) Study: a prospective, randomized control trial. *Circulation* 17-2-2009;119(6):820-7.

(11) Hong DM, Jeon Y, Lee CS et al. Effects of remote ischemic preconditioning with postconditioning in patients undergoing off-pump coronary artery bypass surgery--randomized controlled trial. *Circ J* 2012;76(4):884-90.

(12) Rahman IA, Mascaro JG, Steeds RP et al. Remote ischemic preconditioning in human coronary artery bypass surgery: from promise to disappointment? *Circulation* 14-9-2010;122(11 Suppl):S53-S59.

(13) Young PJ, Dalley P, Garden A et al. A pilot study investigating the effects of remote ischemic preconditioning in high-risk cardiac surgery using a randomised controlled double-blind protocol. *Basic Res Cardiol* 2012;107(3):1-10.

(14) Iliodromitis EK, Kyrzopoulos S, Paraskevaidis IA et al. Increased C reactive protein and cardiac enzyme levels after coronary stent implantation. Is there protection by remote ischaemic preconditioning? *Heart* 2006;92(12):1821-6.

(15) Prasad A, Gossl M, Hoyt J et al. Remote ischemic preconditioning immediately before percutaneous coronary intervention does not impact myocardial necrosis, inflammatory response and circulating endothelial progenitor cell counts. A single center randomized sham controlled trial. *Catheter Cardiovasc Interv* 19-4-2012.

(16) Hausenloy DJ, Yellon DM. **"Conditional Conditioning" in cardiac bypass surgery**. *Basic Res Cardiol* 2012;107(3):1-6.

(17) Thielmann M, Kottenberg E, Kleinbongard P et al. Cardioprotective and prognostic effects of remote ischaemic preconditioning in patients undergoing coronary artery bypass surgery: a single-centre randomised, double-blind, controlled trial. *Lancet* 17-8-2013;382(9892):597-604.

(18) Hausenloy DJ, Candilio L, Laing C et al. Effect of remote ischemic preconditioning on clinical outcomes in patients undergoing coronary artery bypass graft surgery (ERICCA): rationale and study design of a multi-centre randomized double-blinded controlled clinical trial. *Clin Res Cardiol* 2012;101(5):339-48.

(19) Meybohm P, Zacharowski K, Cremer J et al. Remote ischaemic preconditioning for heart surgery. The study design for a multi-center randomized double-blinded controlled clinical trial--the RIPHeart-Study. *Eur Heart J* 2012;33(12):1423-6.

(20) Botker HE, Kharbanda R, Schmidt MR et al. Remote ischaemic conditioning before hospital admission, as a complement to angioplasty, and effect on myocardial salvage in patients with acute myocardial infarction: a randomised trial. *Lancet* 27-2-2010;375(9716):727-34.

(21) Munk K, Andersen NH, Schmidt MR et al. Remote Ischemic Conditioning in Myocardial Infarct Patients Treated with Primary Angioplasty: Impact on Left Ventricular Function Assessed by Comprehensive Echocardiography and Gated SPECT. *Circ Cardiovasc Imaging* 8-9-2010.

(22) Rentoukas I, Giannopoulos G, Kaoukis A et al. Cardioprotective role of remote ischemic periconditioning in primary percutaneous coronary intervention: enhancement by opioid action. *JACC Cardiovasc Interv* 2010;3(1):49-55.

(23) Crimi G, Pica S, Raineri C et al. Remote ischemic post-conditioning of the lower limb during primary percutaneous coronary intervention safely reduces enzymatic infarct size in anterior myocardial infarction: a randomized controlled trial. *JACC Cardiovasc Interv* 2013;6(10):1055-63.

(24) White SK, Frohlich GM, Sado DM et al. Remote ischemic conditioning reduces myocardial infarct size and edema in patients with ST-segment elevation myocardial infarction. *JACC Cardiovasc Interv* 2014.

(25) Sloth AD, Schmidt MR, Munk K et al. Improved long-term clinical outcomes in patients with ST-elevation myocardial infarction undergoing remote ischaemic conditioning as an adjunct to primary percutaneous coronary intervention. *Eur Heart J* 2014;35(3):168-75.

(26) Munk K, Andersen NH, Schmidt MR et al. Remote Ischemic Conditioning in Patients With Myocardial Infarction Treated With Primary Angioplasty: Impact on Left Ventricular Function Assessed by Comprehensive Echocardiography and Gated Single-Photon Emission CT. *Circ Cardiovasc Imaging* 1-11-2010;3(6):656-62.

(27) van dH, I, De Luca G, Ottervanger JP et al. ST-segment elevation resolution and outcome in patients treated with primary angioplasty and glucose-insulin-potassium infusion. *Am Heart J* 2005;149(6):1135.

(28) Gibson CM, Cannon CP, Daley WL et al. **TIMI frame count: a quantitative method of assessing coronary artery flow**. *Circulation* 1-3-1996;93(5):879-88.

(29) Rentrop KP, Cohen M, Blanke H, Phillips RA. Changes in collateral channel filling immediately after controlled coronary artery occlusion by an angioplasty balloon in human subjects. *J Am Coll Cardiol* 1985;5(3):587-92.

(30) Terkelsen CJ, Jensen LO, Tilsted HH et al. Health care system delay and heart failure in patients with ST-segment elevation myocardial infarction treated with primary percutaneous coronary intervention: follow-up of population-based medical registry data. *Ann Intern Med* 20-9-2011;155(6):361-7.

(31) Zalewski J, Nycz K, Przewlocki T et al. Evolution of myocardial perfusion during primary angioplasty in spontaneously reperfused infarct-related artery: impact on long-term clinical outcomes and left ventricular function recovery. *Int J Cardiol* 17-2-2011;147(1):25-31.

(32) Thibault H, Piot C, Staat P et al. **Long-term benefit of postconditioning**. *Circulation* 26-2-2008;117(8):1037-44.

(33) Lonborg J, Kelbaek H, Vejlstrup N et al. Cardioprotective effects of ischemic postconditioning in patients treated with primary percutaneous coronary intervention, evaluated by magnetic resonance. *Circ Cardiovasc Interv* 1-2-2010;3(1):34-41.

(34) Andreka G, Vertesaljai M, Szantho G et al. Remote ischaemic postconditioning protects the heart during acute myocardial infarction in pigs. *Heart* 2007;93(6):749-52.

(35) Giri S, Chung YC, Merchant A et al. **T2 quantification for improved detection of myocardial edema**. *J Cardiovasc Magn Reson* 2009;11(1):56.

(36) Verhaert D, Thavendiranathan P, Giri S et al. **Direct T2 quantification of myocardial edema in acute ischemic injury**. *JACC Cardiovasc Imaging* 2011;4(3):269-78.

(37) Thavendiranathan P, Walls M, Giri S et al. Improved detection of myocardial involvement in acute inflammatory cardiomyopathies using T2 mapping. *Circ Cardiovasc Imaging* 2012;5(1):102-10.

(38) Ortiz-Perez JT, Meyers SN, Lee DC et al. Angiographic estimates of myocardium at risk during acute myocardial infarction: validation study using cardiac magnetic resonance imaging. *Eur Heart J* 2007;28(14):1750-8.

(39) Eitel I, Desch S, Fuernau G et al. Prognostic significance and determinants of myocardial salvage assessed by cardiovascular magnetic resonance in acute reperfused myocardial infarction. *J Am Coll Cardiol* 1-6-2010;55(22):2470-9.

(40) O'Regan DP, Ahmed R, Karunanithy N et al. Reperfusion hemorrhage following acute myocardial infarction: assessment with T2* mapping and effect on measuring the area at risk. *Radiology* 2009;250(3):916-22.

(41) Ross AM, Gibbons RJ, Stone GW, Kloner RA, Alexander RW. A randomized, double-blinded, placebo-controlled multicenter trial of adenosine as an adjunct to reperfusion in the treatment of acute myocardial infarction (AMISTAD-II). *J Am Coll Cardiol* 7-6-2005;45(11):1775-80.

48. Appendix 1 – CMR protocol

i) Siemens or Philips 1.5 T scanners

j) Transverse half Fourier acquisition single-shot turbo spin-echo sequences for extracardiac anatomical images.

k) Multiplanar balanced steady-state free precession (voxel size, 1.3 x 1.3 x 8 mm3) cine sequences for wall motion abnormalities volumetric analysis.

l) T2 maps acquired from three T2-weighted images at different T2 preparation time (0 ms, 24 ms, and 55 ms, respectively; repetition time = 3 x R-R, voxel 1.9 x 1.9x 6 mm3; motion correction and fitting should then be performed as previously described to obtain the colored T2 maps).

m) Segmented two-dimensional inversion-recovery turbo fast low-angle shot late gadolinium-enhanced (LGE) sequences at 10 - 15 minutes after contrast agent injection (voxel size, 1.3 x 1.3 x 8 mm3).

n) All images to be acquired in breath-hold and to be ECG-triggered.

o) Matching contiguous short-axis views of the entire left ventricle should be obtained for cines, T2 maps and LGE.

p) The contrast agent, Gadoterate meglumine, (gadolinium-DOTA, marketed as Dotarem, Guerbet S.A., Paris, France) at a dose of 0.1 mmol/kg should be administered as a bolus.

49. Appendix 2 – Glossary

AAR	Area at risk	A comparison of the severity of a coronary artery lesion and the volume of heart muscle tissue (myocardium) it supplies. (See also **APPROACH** and **BARI**)
ACS	Acute coronary syndrome	This refers to a group of symptoms caused by obstructed coronary arteries. The symptoms include- 'crushing chest pains', nausea and sweating. These symptoms usually occur as part of an **MI**.
NSAE	Non-Serious Adverse Event	See **SAE**
AF	Atrial fibrillation	A common irregular heartbeat caused by the top chambers in the heart (the atriums) quivering (fibrillating) This rhythm is often the cause of 'palpitations'.
APPROACH	Alberta Provincial Project for Outcome Assessment in Coronary Heart Disease Jeopardy Score	A myocardial jeopardy score used to estimate the amount of myocardium at risk based on the severity of the coronary artery lesion and the volume of myocardium it supplies. (See also **BARI**)
AUC	Area under the curve	Used to calculate release of enzymes (for example Troponin) over time. (See **hsTropT**)
BARI	Bypass Angioplasty Revascularisation Investigation Jeopardy Score	A myocardial jeopardy score used to estimate the amount of myocardium at risk based on the severity of the coronary artery lesion and the volume of myocardium it supplies. (See also **APPROACH**)
BHF	British Heart Foundation	A major funder and authority in cardiovascular research, education and care, and relies predominantly on voluntary donations to meet its aims. In order to increase income and maximise the impact of its work, it also works with other organisations to combat premature death and disability from cardiovascular disease.
Call to balloon time		The time taken from the phone call reporting the heart attack to the start of the angioplasty
CABG	Coronary Artery Bypass Graft	A surgical procedure performed to relieve angina and reduce the risk of death from coronary artery disease. Arteries or veins from elsewhere in the patient's body are grafted to the coronary arteries to bypass atherosclerotic narrowings and improve the blood supply to the myocardium (heart muscle).
CK-MB	Creatinine Kinase	A blood test that measures the presence of cardiac enzymes. These act as markers that can assist in the diagnosis of a heart attack. (See **hs Trop-T**)
CMR	Cardiac MRI	See **MRI**

CONDI-2		The Danish arm of the trial. Data will be combined from ERIC-PPCI and CONDI 2 in the analysis.
CRF	Case Report Form	A specialised form (either on paper, or electronic when it's sometimes called an eCRF) used to collect clinical data for a trial or a study.
CTU	Clinical Trials Unit	A specialised research unit which designs, coordinates and analyses clinical trials and other studies.
DSMC	Data Safety and Monitoring Committee	An independent group of experts formed to monitor patient safety and treatment efficacy data while a clinical trial is ongoing.
ECG	Electrocardiogram	A test that records the electric activity of your heart. (ST elevation/depression, T wave, QRS complex- these terms represent aspects of an ECG reading).
EF	Ejection fraction	See **LVEF**
eGFR	Estimated glomerular filtration rate	This is a test to see how well the kidneys are working. It estimates how much blood is filtered by the kidneys over a given period of time.
HHF	Hospitalisation for heart failure	An admission of longer than 24 hours for heart failure. Heart failure is a health condition in which the heart has a reduced ability to pump blood to the body.
hs Trop-T	High sensitivity Troponin-T	A blood test that measures the presence of cardiac enzymes. These act as markers that can assist in the diagnosis of a heart attack. (see **CK-MB**)
IHD	Ischaemic heart disease	Ischaemia is the restriction in blood supply to tissues, resulting in reduced oxygen and glucose supply affecting the cells, causing pain.
IRI	Ischaemia reperfusion injury	the tissue damage caused when tissue experiences a period of ischaemia (or lack of oxygen) and subsequently blood supply returns to the tissue.
LAD	Left anterior descending	One of the arteries of the heart
LBBB	Left bundle branch block	A cardiac contraction condition where activation of the left ventricle is delayed, causing the left ventricle to contract later than the right ventricle. This may require treatment with a pacemaker.
LGE	Late gadolinium enhancement	See **MRI**
LSHTM	London School of Hygiene and Tropical Medicine	The **clinical trial unit** coordinating the ERIC-PPCI trial is based at LSHTM.
LV	left ventricle / left ventricular	Along with the right ventricle, one of the two large chambers that collect and expel blood in the heart.
LVEF	left ventricular ejection fraction	Often given as a percentage, it is the volumetric fraction of blood pumped out of the left ventricle in the heart with each heartbeat.

MI	Myocardial infarction	Or 'Heart attack'. An Interruption of blood supply caused by a blockage in the blood vessels to the heart leading to cell or tissue death (infarction). Sometimes referred to as NSTEMI or **STEMI**.
MRI	Magnetic resonance imaging	A medical imaging technique used in radiology to visualise internal structures in the body. (LGE- Late gadolinium-enhanced images- A more advanced MRI).
Myocardium		The myocardium is the muscle tissue of the heart, and forms a thick middle layer between the outer epicardium layer and the inner endocardium layer.
NIHR	National Institute for Health Research	The NIHR is the health research arm of the NHS.
NYHA class	New York Heart Association	A simple way of classifying the extent of heart failure (see definition) using physical activity, chest pain and breathlessness as a measure. See **CCS**.
OMT	Optimal Medical Therapy	This includes the best medication (tablets) that are currently available for heart failure, at doses that are individually tailored. This strategy often also involves insertion of a special type of pacemaker (called a biventricular pacemaker, which may also function as an Implantable Cardioverter Defibrillator).
PI	Principal Investigator	The doctor leading the trial at the site level. Each site has a principal investigator who will delegate roles and responsibilities to other staff using a delegation log.
PIS	Patient Information Sheet	A leaflet given to the patient which explains the trial and their involvement in it in lay language.
PPCI	Primary percutaneous coronary intervention	This procedure is used to treat the narrowed coronary arteries of the heart. A small tube is inserted in the groin or wrist and advanced to the heart. Small balloons and stents are used to open up the narrowings and improve blood flow to the heart muscle. This is sometime also known as Primary Angioplasty.

RIC	Remote ischaemic conditioning	RIC describes the process of applying cycles of limited blood flow (ischaemia) and reinstated blood flow (reperfusion) to an organ or tissue as a protection mechanism for other organs. The full extent and mechanism of this protection is unclear. However there is evidence from previous extensive work on patients that there is a pathway linking the pre conditioned organ or tissue (in the case of ERIC-PPCI the upper arm) to the heart.
SAE	Serious Adverse Event	Any event such as an illness or an accident that results in death, is life-threatening, requires hospitalisation or prolongation of existing hospitalisation, results in persistent or significant disability or incapacity, or is a congenital anomaly or birth defect.
SBP	Systolic Blood Pressure	During each heartbeat, blood pressure varies between a maximum (systolic) and a minimum (diastolic) pressure.
SD	Standard deviation	Measures the amount of variation from the average.
SPECT	Single-photon emission computed tomography	An imaging technique that produces three dimensional images of functional processes in the body.
SSFP	Steady-state free precession imaging	A magnetic resonance imaging (**MRI**) technique which uses steady states of magnetisations.
STEMI	ST elevated myocardial infarction	ST elevation refers to a finding on an **ECG**, wherein the trace in the ST segment is abnormally high above the isoelectric line.
TIMI Perfusion Grade (Blush)	Thrombolysis in myocardial infarction perfusion grade	A technique to assess myocardial infusion in the capillary bed on a coronary angiogram
TIMI flow grade	Thrombolysis in myocardial infarction flow grade	A measure to assess epicardial coronary blood flow
UK NIAP	UK National Infarct Angioplasty Project	A joint project set up by the British Cardiac Society and the Department of Health to test the feasibility of implementing a countrywide angioplasty service to treat cases of acute myocardial infarction in England.

50. Appendix 3 – EuroQol EQ-5D-5L

ERIC-PPCI

Effect of Remote Ischaemic Conditioning on clinical outcomes in ST segment elevation myocardial infarction patients undergoing Primary Percutaneous Coronary Intervention

EQ-5D-5L

Health Questionnaire

English version for the UK

**ERIC-PPCI Trial Number:**	E P ☐☐☐☐☐
**Date of Birth:**	d d m m y y y y

EQ-5D-5L

ERIC-PPCI Trial Number: E P | | | | | |

Date completed | d | d | m | m | y | y | y | y |

Under each heading, please tick the **ONE** box that best describes your health **TODAY**

Mobility

I have no problems in walking about ☐
I have slight problems in walking about ☐
I have moderate problems in walking about ☐
I have severe problems in walking about ☐
I am unable to walk about ☐

Self-Care

I have no problems washing or dressing myself ☐
I have slight problems washing or dressing myself ☐
I have moderate problems washing or dressing myself ☐
I have severe problems washing or dressing myself ☐
I am unable to wash or dress myself ☐

Usual Activities (e.g. work, study, housework, family or leisure activities)

I have no problems doing my usual activities ☐
I have slight problems doing my usual activities ☐
I have moderate problems doing my usual activities ☐
I have severe problems doing my usual activities ☐
I am unable to do my usual activities ☐

Pain/Discomfort

I have no pain or discomfort ☐
I have slight pain or discomfort ☐
I have moderate pain or discomfort ☐
I have severe pain or discomfort ☐
I have extreme pain or discomfort ☐

Anxiety / Depression

I am not anxious or depressed ☐
I am slightly anxious or depressed ☐
I am moderately anxious or depressed ☐
I am severely anxious or depressed ☐
I am extremely anxious or depressed ☐

EQ-5D-5L

ERIC-PPCI Trial Number: **E P** | | | | | |

- We would like to know how good or bad your health is TODAY.
- This scale is numbered from 0 to 100.
- 100 means the **best** health you can imagine.
- 0 means the **worst** health you could imagine.
- Mark an X on the scale to indicate how your health is TODAY.
- Now, please write the number you marked on the scale in the box below.

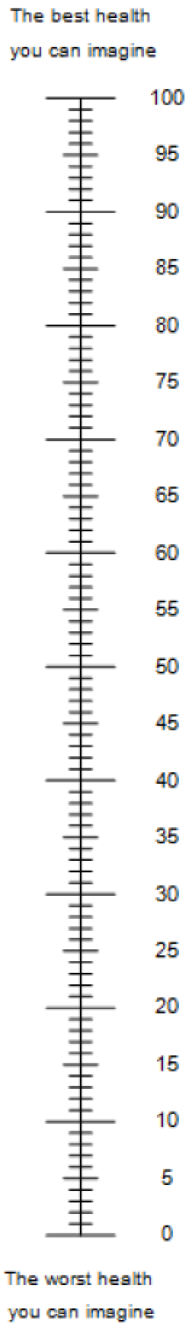

YOUR HEALTH TODAY =

Appendix 4: Additional Substudies

Coronary Physiology substudy

Background and rationale

The assessment of the efficacy of reperfusion at the time of PPCI is challenging. Angiography and ST-resolution alone are good but imperfect indices. There is increasing data to support the use of coronary physiology indices measured at the time of PPCI as a surrogate index to measure the efficacy of reperfusion and provide insights into the effects on the microcirculation. Thus, the index of microcirculatory resistance (IMR) relates to biochemical and MRI infarct size, and an IMR value of >40 units (observed in about 1/3 PPCI patients) at the completion of PPCI is associated with adverse outcome. Recent intervention studies have adopted IMR as primary endpoint to test the effects of drugs or aspiration, although the clinical significance of this is uncertain. Our own group has been studying the changes in coronary physiology indices at the time of PPCI after aspiration and before coronary stenting. In addition we have used the opportunity to sample coronary sinus, aortic, culprit and venous blood for biomarker studies.

Hypothesis

1. RIC has beneficial effects on IMR at the completion of PPCI compared with control
2. RIC improves the response of the microcirculation at the time of PPCI to coronary stenting
3. IMR relates to CMR imaging indices in an interventional trial
4. Identification of circulating marker or effluent from the myocardium induced by RIC

Method

Coronary flow and pressure measurements are made using a coronary pressure/temperature sensitive guidewire, using established methods in routine use in clinical PCI procedures. The pressure wire is interchangeable with the coronary guide wire used in all PCI procedures, so is incorporated in to the procedure. Measurements that assess the integrity of the small blood vessels of the heart are made during the PPCI procedure, once the artery is opened and blood flow has been restored to the heart, and after implantation of a coronary stent Saline is flushed through the guide catheter to measure coronary flow using the transit time calculation. Adenosine is used as in standard clinical practice to increase blood flow. [1]

Sample Size

There are no published trials in this area and so sample size calculations have been based on similar patient groups and published literature: In the TIME trial mean IMR in an ACS cohort is 30 (SD 20). An effect size of 30% reduction in IMR is proposed, and allowing for dropout a sample size of 152 patients has been calculated at 80% power, 5% significance. The PATA STEMI study has an IMR reduction from 38U with a 26% effect size, and 128 patients. A study comparing aspiration and abciximab powered to show an almost 40% reduction in IMR from 34 to 20 calculated a sample size of 40. Published data suggest that the mean IMR after PPCI is 38 with an SD of 30. We would propose an effect size of 30% reduction in the treatment group and at 80% power with significance 5% the sample size would be 168 patients (84 per group). Allowing for dropout 180 patients will be required.

Feasibility

Coronary physiology and IMR measures are practical and feasible in STEMI PPCI and we have extensive experience of these protocols in John Radcliffe Hospital Oxford as part of an ongoing OxAMI study, which is an observational cohort study. [2-6]

John Radcliffe Hospital, Oxford will be the lead centre. Additional selected sites will be invited to participate in this substudy.

References

1. McGeoch R, Watkins S, Berry C, Steedman T, Davie A, Byrne J, Hillis S, Lindsay M, Robb S, Dargie H, Oldroyd K. The index of microcirculatory resistance measured acutely predicts the extent and severity of myocardial infarction in patients with st-segment elevation myocardial infarction. *JACC. Cardiovascular interventions*. 2010;3:715-722
2. Patel N, Petraco R, Dall'Armellina E, Kassimis G, De Maria GL, Dawkins S, Lee R, Prendergast BD, Choudhury RP, Forfar JC, Channon KM, Davies J, Banning AP, Kharbanda RK. Zero-flow pressure measured immediately after primary percutaneous coronary intervention for st-segment elevation myocardial infarction provides the best invasive index for predicting the extent of myocardial infarction at 6 months: An oxami study (oxford acute myocardial infarction). *JACC. Cardiovascular interventions*. 2015;8:1410-1421
3. De Maria GL, Cuculi F, Patel N, Dawkins S, Fahrni G, Kassimis G, Choudhury RP, Forfar JC, Prendergast BD, Channon KM, Kharbanda RK, Banning AP. How does coronary stent implantation impact on the status of the microcirculation during primary percutaneous coronary intervention in patients with st-elevation myocardial infarction? *European heart journal*. 2015
4. Ruparelia N, Godec J, Lee R, Chai JT, Dall'Armellina E, McAndrew D, Digby JE, Forfar JC, Prendergast BD, Kharbanda RK, Banning AP, Neubauer S, Lygate CA, Channon KM, Haining NW, Choudhury RP. Acute myocardial infarction activates distinct inflammation and proliferation pathways in circulating monocytes, prior to recruitment, and identified through conserved transcriptional responses in mice and humans. *European heart journal*. 2015;36:1923-1934
5. Cuculi F, De Maria GL, Meier P, Dall'Armellina E, de Caterina AR, Channon KM, Prendergast BD, Choudhury RP, Forfar JC, Kharbanda RK, Banning AP. Impact of microvascular obstruction on the assessment of coronary flow reserve, index of microcirculatory resistance, and fractional flow reserve after st-segment elevation myocardial infarction. *Journal of the American College of Cardiology*. 2014;64:1894-1904
6. Cuculi F, Dall'Armellina E, Manlhiot C, De Caterina AR, Colyer S, Ferreira V, Morovat A, Prendergast BD, Forfar JC, Alp NJ, Choudhury RP, Neubauer S, Channon KM, Banning AP, Kharbanda RK. Early change in invasive measures of microvascular function can predict myocardial recovery following pci for st-elevation myocardial infarction. *European heart journal*. 2014;35:1971-1980

Thrombosis substudy

Background

The pathogenesis of acute coronary syndromes (ACS) involves thrombosis on a background of plaque rupture and erosion, which can manifest in ST-elevation MI (STEMI). The risk of thrombosis is determined by the balance between the prothrombotic forces in blood and the capacity of the innate endogenous thrombolytic system to naturally dissolve any thrombus. Until recently, endogenous thrombolysis has been difficult to measure. Impaired thrombolytic status has been shown in NSTEMI, end-stage renal disease, stroke, and diabetes; and is increasingly recognised as a risk factor for arterial thrombosis. Effectiveness of endogenous thrombolysis is determined by thrombus properties (clot strength, determined by fibrin density, pore size and size of fibrin strands) and the rate of fibrinolysis, determined mainly by release of t-PA from the endothelium and PAI-1 from activated platelets. The exact mechanism through which ischaemic preconditioning improves outcomes in patients with STEMI is still not fully understood. In dogs, ischaemic preconditioning has been accompanied by down-regulation of platelet-fibrinogen binding and formation of neutrophil-platelet aggregates (1). In patients, platelet reactivity was reduced after repetitive, compared to single cycle exercise (2), whilst marked systemic platelet activation has been demonstrated in ACS (3) or acute limb ischemia (4). In patients undergoing radiofrequency ablation of atrial fibrillation, RIC reduced platelet activation and reactivity (5). In patients with subarachnoid hemorrhage, RIC prolonged the PT and INR (6). Activated platelets play an important role in the process of myocardial ischemia-reperfusion injury, and platelet-derived P-selectin is a critical mediator. In patients with coronary artery disease (7), RIC prior to exercise stress testing reduced ADP-stimulated platelet aggregation. However, the potential clinical benefit of any of these findings remains to be seen. Our pilot data on thrombotic status in PPCI: Data on 80 STEMI patients has yielded promising results. Rapid endogenous fibrinolysis was related to spontaneous STsegment resolution, TIMI 3 flow at presentation, and uneventful outcomes. Impaired endogenous lysis was associated with TIMI 0 flow and MACE within 30 days.

Hypothesis

Platelet activation and impaired endogenous thrombolysis are critical determinants of outcome in patients with STEMI undergoing PPCI. RIC reduces ischaemic insult and improves outcome, and this is at least in part, through favourable effects on thrombotic status.

Methods

All patients participating in ERIC-PPCI that are recruited at Lister Hospital, Stevenage will be enrolled.

Blood samples

Venous blood samples (20ml) will be taken at presentation, 1 day post and 2-3 days post-STEMI and at the 6-8 week follow-up visit, for immediate point-of care thrombotic assessment, and the remainder will be stored in an accredited lab for later assessment of thrombogenesis and fibrinolysis. Samples will be taken by trained cath lab nurses and doctors already familiar with this protocol.

Assessment of Thrombotic Status

Thrombotic status will be assessed using the point-of-care Global Thrombosis Test (GTT, Thromboquest Ltd., UK) and tests of thrombogenesis and fibrinolysis using micro-titre based assays on fresh frozen plasma (FFP). The GTT is a relatively novel point-of-care test, utilising native, nonanticoagulated whole blood, without external agonists, under high shear stress conditions, akin to that in a stenosed artery. It assesses platelet aggregation (time to occlusive thrombus formation; occlusion time (OT) and endogenous thrombolytic status (time to endogenous lysis of thrombus formed in the first phase of the test; lysis time(LT)) [10,11,21].

Other Blood Tests

At each timepoint, a measurement of coagulation (fibrinogen, INR) and inflammation (high sensitivity C-reactive protein). Blood will also be spun and stored for future analysis as determined by study results.

Follow-up

Follow up will be the same as the ERIC-PPCI protocol, however all 6-8 week follow-up will be conducted at the outpatient clinic.

Handling of results

Patients receiving RIC or sham RIC, will be compared with respect to thrombotic status pre and post-PPCI, as well as the absolute change in thrombotic status pre and post intervention, and differences in ECG ST-resolution and clinical outcomes.

References

1. Preconditioning ischemia attenuates molecular indices of platelet activation-aggregation. Linden MD, Whittaker P, Frelinger AL 3rd, Barnard MR, Michelson AD, Przyklenk K. J Thromb Haemost. 2006 Dec;4(12):2670-7.

2. Brief low-workload myocardial ischaemia induces protection against exercise-related increase of platelet reactivity in patients with coronary artery disease. Scalone G, Coviello I, Barone L, Pisanello C, Sestito A, Lanza GA, Crea F. Heart. 2010 Feb;96(4):263-8.

3. Increased platelet binding to circulating monocytes in acute coronary syndromes. J. Sarma, C.A. Laan, S. Alam, et al. Circulation, 105 (2002), pp. 2166–2171.

4. Perioperative platelet and monocyte activation in patients with critical limb ischemia. A. Burdess, .F. Nimmo, N. Campbell, et al. J Vasc Surg, 52 (2010), pp. 697–703.

5. Effect of remote ischaemic preconditioning on platelet activation and reactivity induced by ablation for atrial fibrillation. Stazi A, Scalone G, Laurito M, Milo M, Pelargonio G, Narducci ML, Parrinello R, Figliozzi S, Bencardino G, Perna F, Lanza GA, Crea F. Circulation. 2014 Jan 7;129(1):11-7.

6. Effects of remote ischaemic preconditioning on the coagulation profile of patients with aneurysmal subarachnoid hemorrhage: a case-control study. Mayor F, Bilgin-Freiert A, Connolly M, Katsnelson M, Dusick JR, Vespa P, Koch S, Gonzalez NR. Neurosurgery. 2013 Nov;73(5):808-15;

7. Upper arm intermittent ischaemia reduces exercise-related increase of platelet reactivity in patients with obstructive coronary artery disease. I. Battipaglia, G. Scalone, M. Milo, et al. Heart, 97 (2011), pp. 1298–1303.

ERIC-PPCI Summary of Protocol Changes

Version number	Protocol date	Effective date (Approval date)	Changes from previous version
1	28 November 2014	n/a	N/A - Protocol submitted as part of initial ethics application.
2	24 February 2015	25 March 2015	First approved version of the protocol. Updates from the previous version were requested by the REC in the initial review and included:<br><br>1) Clarification of the consent procedures.<br><br>2) Addition of a flow chart showing the consenting plan.
3	8 March 2016	13 April 2016	The following changes were made:<br><br>1) The addition of three substudies to the protocol.<br><br>2) Minor adjustments to the trial that were raised during the first few months of recruitment.
4	6 September 2017	6 November 2017	The following changes were made:<br><br>1) The sample size of ERIC-PPCI was increased from 2000 patients to 2600 patients in response to a slightly lower than predicted event rate.<br>2) In Section 4: Endpoints of the protocol we have updated the definitions for hospitilisation for heart failure and stroke. This change brings the protocol in line with the standard operation procedures and makes the protocol more clear.<br>3) Reference to the Myosin C substudy has been removed as this substudy has not gone ahead and will not be part of the trial.
5	10 January 2018	6 March 2018	Protocol updated to reflect the increase in the sample size from 2600 to 2800

			patients. This was done to increase the power of the study while accounting for patients who have been lost to follow-up, withdrawn from the study or refused consent.

CONDI-2 Study Protocol

Effect of Remote Ischaemic Conditioning on clinical outcomes in ST-elevation myocardial infarction patients undergoing primary Percutaneous Coronary Intervention: A multicentre randomised controlled clinical study

Acronym: CONDI 2

Multinational investigator-driven, multi-centre, randomised, controlled, single-blind (Outcomes Assessor), parallel assignment, efficacy prospective clinical trial to investigate whether RIC can improve clinical outcomes (cardiovascular death and hospitalisation for heart failure) at one year in STEMI patients undergoing primary PCI.

Project Identification: Condi 2
Protocol Version: Version 4; May 27, 2013
Sponsor: Danish Strategic Research Council
Coordinating Principal Investigators:

Hans Erik Bøtker, senior consultant, MD, DMSci, Professor Cardiology, Department of Cardiology Aarhus, University Hospital Skejby, DK-8200 Aarhus N, Denmark, Telephone +45784520210

The clinical study will be conducted, and essential documentation archived, in compliance with UCR SOPs and standards, which incorporate the requirements of the ICH Guideline for Good Clinical Practice and EN ISO 14155:2011(E).

- **Synopsis**

**Name of Sponsor Investigator:**<br>Hans Erik Bøtker, senior consultant, MD, DMSci, Professor Cardiology, Department of Cardiology, Aarhus University Hospital Skejby, DK-8200 Aarhus N, Denmark
**Name of Investigational Medical Device**: The CellAegis automated cuff
**Title of Study:**<br>Effect of Remote Ischaemic Conditioning on clinical outcomes in ST-elevation myocardial infarction patients undergoing primary Percutaneous Coronary Intervention: A multinational multicentre randomised controlled clinical study
**Trial Management Groups (TMG)**<br>**Coordinating Principal Investigator:**<br>Hans Erik Bøtker, senior consultant, MD, DMSci, Professor Cardiology, Department of Cardiology, Aarhus University Hospital Skejby, DK-8200 Aarhus N, Denmark<br><br>**Data Monitoring Committee (DMC), trial safety committee:**<br>Rajesh K. Kharbanda (Oxford University Hospital)<br>Jens Flensted Lassen (Aarhus University Hospital)<br>NN (Madrid, S)<br><br>**Trial endpoints validation committee:**<br>Anne Kaltoft (Aarhus University Hospital)<br>Ulla Kristine Møller Liendgaard (Aarhus University Hospital)<br>NN (London, UK)<br>NN (Madrid, S)<br><br>**Trial Steering Committee (TSC)**:<br>Hans Erik Bøtker (Aarhus University Hospital, Skejby), Michael Rahbek Schmidt (Aarhus University Hospital), Thomas Engstrøm (Rigshospitalet), Jan Ravkilde (Aalborg Sygehus), Lisette Okkels Jensen (Odense University Hospital), Derek Hausenloy (The Hatter Cardiovascular Institute, London), Henrik Toft Sørensen (Aarhus University Hospital), Erika Frischknecht Christensen (Pre-Hospital, Aarhus), U Kristine Møller (Aarhus University Hospital, Skejby), Freddy Lippert (Pre-Hospital, Copenhagen), Borja Ibañez (Hospital Clínico San Carlos de Madrid)
**Study Centres**:<br>• Department of Cardiology, Aarhus University Hospital, Skejby, DK-8200 Aarhus N, Denmark<br>• The Heart Centre, Rigshospitalet, 2100 Copenhagen Ø, Denmark<br>• The Heart Centre, Aalborg Sygehus, 9100 Aalborg, Denmark

• Department of Cardiology, Odense University Hospital, 5000 Odense, Denmark<br>• The Hatter Cardiovascular Institute, University College, London WC1E 6HX, United Kingdom<br>• Hospital Clínico San Carlos de Madrid, 28040 Madrid, Spain	
**Planned study period**:<br>2013-2016	**Phase of development**:<br>Improve routine clinical care of STEMI patients
**Objectives**:<br>To determine whether Remote Ischaemic Conditioning (RIC) improves clinical outcomes (cardiovascular (CV) mortality and hospitalisation for heart failure (HHF)) at one year in ST-elevation Myocardial infarction (STEMI) patients undergoing primary Percutaneous Coronary Intervention (pPCI).	
**Diagnosis**:<br>ST-elevation Myocardial infarction (STEMI)	
**Methodology**:<br>A multinational and -multicentre investigator-driven, prospective randomised, controlled, single-blind, parallel assignment, clinical efficacy trial.	
**Randomisation and allocation procedure**:<br>Eligible patients will be randomised (via a secure web-site) prior to pPCI, preferably in the ambulance or on arrival at the hospital.<br>The assessor of clinical outcomes will be blinded to the treatment allocation.	
**Number of subjects (planned):**<br>2300 patients	
**Inclusion criteria**:<br>• Male and female patients (>18 years old) presenting with chest pain within 12 hours, lasting for more than 30 minutes<br>• Suspected STEMI (ST-elevation at the J-point in two contiguous leads with the cut-off points: ≥0.2 millivolt (mV) in men or ≥0.15 mV in women in leads V2-V3 and/or ≥0.1 mV in other leads) or significant ST–depression in V2-V4 (suspected posterior infarction) or new left bundle branch block, who are eligible for primary PCI.<br>• Informed consent obtained<br>• Life expectancy of more than 1 year	
**Exclusion criteria:**<br>• Previous by-pass surgery<br>• Myocardial infarction (MI) or treatment with thrombolysis within 30 days<br>• Patients treated with cooling	
**Criteria for evaluation**:<br>*Primary clinical endpoint*:<br>Cardiovascular mortality and hospitalisation for heart failure at one year.<br><br>*Secondary clinical endpoints*:	

Myocardial infarct size at day 3 (72 hour area under curve serum troponin T)<br>Left ventricular function on day three and three months post pPCI (Echocardiography)<br>Re-infarction, stroke and revascularisation at one year (Readmission)
**Power Calculations:**<br>To detect a 25% reduction in CV mortality in the RIC-treated group (from 17.4% to 13.0%), with a power of 80% and a significance level of 5%, a sample size of 1044 patients will be required for each trial arm (2088 in total). To account for and failure of fulfilling entry criteria on arrival we plan to include 2300 patients.<br><br>**Pre-specified subgroup analyses**: A limited number of subgroup analyses will be undertaken: age, gender, diabetes, chest pain to PCI time, and RIC to PCI time. These subgroups will be analysed using interaction tests.<br><br>**Statistical analysis:** A comparison of the event rate one year after STEMI between RIC and control arms of the trial. Survival analyses techniques will be used for CV death and HHF and other clinical endpoints.

Content

1 Synopsis 82
2 Abbreviations 86
3 Concept 87
4 Hypothesis 88
5 Objectives 88
5.1 Overall primary objective 88
5.2 Secondary objectives: 88
6 Endpoints 88
6.1 Primary clinical endpoint 88
6.2 Secondary clinical endpoints 88
7 S/T methodology and associated work plan 89
7.1 Trial Design: 89
7.2 Setting 89
7.3 Target population 89
8 Study progress 90
8.1 Patient information 90
8.2 Randomisation procedure ..... **Error! Bookmark not defined.**
8.3 Procedures and treatment 90
8.4 Discontinuation of study participation 92
9 Benefit of the study 92
10 Statistics 93
10.1 Power Calculations 93
10.2 Pre-specified subgroup analyses 93
10.3 Statistical analysis 93
11 Project timetables and recruitment feasibility 93
12 Ethical issues 94
12.1 Data storing 94
12.2 Expected impacts listed in the work programme 94
13 Financial standing 95
14 Reference List 96

- **Abbreviations**

AE	Adverse event
AR	Adverse reaction
CABG	Coronary artery bypasses graft
CHD	Coronary heart disease
DMC	Data Monitoring Committee and Endpoints validation committee
CV	Cardiovascular
ECCO	Echocardiography
ECG	Electrocardiogram
e-CRF	Electronic-Case Report Form
HF	Heart failure
HHF	Hospitalisation for heart failure
LAD	Proximal left anterior descending
MI	Myocardial infarct
mPTP	Mitochondrial permeability transition pore
PCI	Percutaneous coronary intervention
pPCI	Primary percutaneous coronary intervention
RIC	Remote Ischaemic Conditioning
SAE	Serious adverse event
SAR	Serious adverse reaction
STEMI	ST-elevation myocardial infarction
TMG	Trial Management Group
TSC	Trial Steering Committee

- **Concept**

Coronary heart disease (CHD) is the leading cause of death in Denmark and Europe, accounting for 1.92 million deaths in Europe per year (2008 European Cardiovascular Disease Statistics): over one in five men (21%) and one in five women (22%) die from CHD. Therefore, novel therapeutic strategies are urgently required to improve clinical outcomes in patients with CHD and further to reduce the health-care and non health-care costs of CHD. One area this needs to be achieved is in patients presenting with a ST-elevation myocardial infarction (STEMI). The current state-of-the-art treatment for patients presenting with a STEMI is timely myocardial reperfusion using primary percutaneous coronary intervention (pPCI) to restore blood flow in the infarct-related coronary artery in the shortest possible time. However STEMI patients undergoing percutaneous coronary intervention (PCI) have a significant mortality and morbidity at one year with 17.4% of patients dying from a cardiovascular (CV) cause or being hospitalised from heart failure (HHF) (1).

Recent advances in this area of research have focused on maintaining the patency of the infarct-related coronary artery and on securing perfusion by preventing distal embolization (2-5).

New treatment strategies are required beyond the open artery to protect the myocardium itself from the detrimental effects of ischaemia-reperfusion injury. Lethal myocardial reperfusion injury has been attributed to oxidative stress, calcium overload, cardiomyocyte hypercontracture and mitochondrial dysfunction induced by the opening of the mitochondrial permeability transition pore (mPTP) (6;7).

Both pre-clinical and recent clinical studies have demonstrated that treatment strategies such as Remote Ischaemic Conditioning (RIC) applied at the time of myocardial reperfusion can reduce myocardial infarct size, confirming the existence of myocardial reperfusion injury (6). In this respect, RIC has been shown to limit myocardial infarct size and preserve cardiac function in recent proof-of-concept clinical studies in STEMI patients undergoing pPCI (8;9). Cardioprotection by RIC is most commonly achieved by manual or automatic inflation of a blood pressure cuff to induce four 5-minute cycles of limb ischaemia and reperfusion (10;11), a technique that was first pioneered by our network allies at Aarhus University Hospital and University College London (12). On the basis of these studies, we have demonstrated that RIC applied in the ambulance during transport to the pPCI unit reduced MI size in STEMI patients undergoing pPCI (8). Subsequently, it has been demonstrated that RIC can reduce myocardial injury in patients undergoing coronary artery bypass graft (CABG) surgery (13), in children undergoing corrective cardiac surgery (14) and in patients with stable angina undergoing elective PCI (15).
However, whether this virtually cost-free non-pharmacological and non-invasive therapeutic strategy can actually improve clinical outcomes in STEMI patients has never been investigated in a randomized controlled clinical trial.

- **Hypothesis**

RIC followed by pPCI improves clinical outcomes in STEMI patients when compared to STEMI controls undergoing standard pPCI evaluated one year post PCI.

- **Objectives**

○ **Overall primary objective**

- To determine whether RIC improves clinical outcomes (Cardiovascular (CV) mortality and hospitalisation for heart failure (HHF)) at one year in STEMI patients undergoing pPCI.

○ **Secondary objectives:**

- To determine, in the pre-specified subgroups, whether age, gender, diabetes, and duration of chest pain to PCI influence the response to RIC.
- To determine whether RIC preserves left ventricular function measured by echocardiography (ECCO) after three day and three months post pPCI
- To determine whether a correlation between biomarkers, patient susceptibility to ischaemia-reperfusion injury and the treatment strategies of RIC can be found. Measured as Areal under the curve 72 hours post pPCI
- To trial the CellAegis automated cuff in its largest clinical study to date.

All these outcomes are relevant to STEMI patients and have the potential to change clinical practice.

Heart failure is defined according to the European Society of Cardiology guidelines, including re-commendations for the diagnostic investigations in ambulatory patients suspected of having heart failure that leads to hospital admission and initiation or change in anticongestive medication.

- **Endpoints**

Primary and secondary clinical endpoints will be adjudicated by an independent end-point committee.

○ **Primary clinical endpoint**

- CV mortality and HHF at one year

○ **Secondary clinical endpoints**

- Myocardial infarct size at day three (72 hour area under curve serum troponin T)
- Left ventricular function on day three and three months post pPCI (Echocardiography)

- Re-infarction, stroke and revascularisation at one year

- **S/T methodology and associated work plan**
  - **Trial Design:**

Multinational investigator-driven, multi-centre, randomised, controlled, single-blind (Outcomes Assessor), parallel assignment, prospective clinical efficacy trial to investigate whether RIC can improve clinical outcomes (CV mortality and HHF) at one year in STEMI patients undergoing pPCI.

  - **Setting**

In all 2300 STEMI patients will be included.
Four centres with on-site facilities for pPCI in Denmark will recruit app. 1300 STEMI patients in total over a 36 month period.
Two centres in respectively London, UK and Madrid, Spain will recruit app. 1000 STEMI patients in total over a 36 month period.
Data collection and analysis will be undertaken through the Department of Cardiology and Department of Clinical Epidemiology, Aarhus University Hospital Skejby Clinical Trials Unit.

  - **Target population**

Possible study candidates are patients with putative STEMI acute admitted to one of the heart centres. There will be no advertising for patients. A total of 2300 patients are to be included over a 36 months period.

Inclusion criteria for recruitment	Exclusion criteria for recruitment
Male and female patients (>18 years old) presenting with chest pain for more than 30 minutes	Previous by-pass surgery
Putative STEMI (ST-elevation at the J-point in two contiguous leads with the cut-off points: ≥0.2 mV in men or ≥0.15 mV in women in leads V2-V3 and/or ≥0.1 mV in other leads)	MI or treatment with thrombolysis within 30 days
New left bundle branch block who are eligible for pPCI (chest pain onset <12 hours)	Patients treated with cooling
Informed consent obtained	
Life expectancy of more than 1 year	

- **Study progress**

○ **Patient information**

The patient will be informed and treated according to the national and international guidelines for Good Clinical Practice and protected under the Act concerning the processing of personal data and health law.
The intervention is performed in the acute phase. Consequently in addition to the ordinary written patient information (A) a short written information (B) is given in the ambulance (One A4 page). Because the nature of the disease and the intervention are acute, the usual requirements about a 24-hour time for consideration and discussion with a lay representative cannot be met. The inherent acute nature of the study necessitates circumvention of these usual requirements as it has previously been done in comparable acute studies. This approach is reasonable because the intervention is without any known risk and because the intervention has potential benefit for the individual patient.

The admitting ambulance doctor or doctor at the receiving hospital or the paramedic will orally inform the patient based on the short written information (B) and hand out the approved written information's (A+B). After oral and written informed consents are, obtained the patient will sign the agreement form and be randomised via a secure web-site to either pPCI with or without RIC by the doctor on duty at one of the receiving hospitals. Computer-generated blocked randomisation lists, stratified by centre, will be prepared in advance of the study.
After information is given in the acute phase the patient does not have much time for reflection before signing the informed consent form. Therefore, additional oral information will be given to the patient after the acute phase by a study nurse or the doctor performing the pPCI based on the full written information (A). Both during the first and second stage of information it will be emphasised that the patient has the right to withdraw his/her informed consent at any time.

○ **Procedures and treatment**

Control group: Will be treated according to standard procedures in the ambulance.

Intervention group, RIC group: The CellAegis auto RIC (automated blood pressure cuff) will be placed on the right upper arm and inflated to 200mmHg for 5 minutes and then deflated for 5 minutes, a programmed cycle which is repeated 4 times in total (therefore the total length of the RIC protocol is 40 minutes). Crucially, the automated cuff includes an oxygen saturation finger probe, which can be used to confirm that complete arterial occlusion has taken place with cuff inflation. If the initial systolic blood pressure is >185mmHg, the cuff will be inflated to 15mmHg above the systolic blood pressure. In recruiting centres where randomisation occurs at the hospital or in cases with short transportation time, the RIC protocol will continue during PCI until successful or until immediately before reperfusion. Further the patients will be treated according to standard procedures in the ambulance.

	Acute intervention		Index event	Follow up
Randomisation	Intervention	Control		
Blood samples [1]			X	
e-CRF [2]			X	X
Echocardiography [3]			X	X

[1] Blood samples for CkMb and TnT drawn acute, 6-8 (±2), 24 (±2) and 72 (±4) hours post-PCI. Samples will be analyzed immediately.

[2] Research assistant complete the electronic case record form. Information regarding re-hospitalisation or death will be drawn from (electronic) patient chart.

[3] Echocardiography (sonogram of the heart), with no risk for the patient. Performed at day three (±1) and three months (±14 days) post-PCI

pPCI incl. the use of stents and antithrombotic regimens will be performed according to standard procedures at the treating hospital.
Blood samples will be drawn according to standard routine no extra biomarkers will be measured. An extra sample of 15 ml will be drawn 72 H post pPCI. All blood samples will be analyzed immediately. No bio bank will be established.
Information regarding re-hospitalisation for hearth failure, AMI, revascularisation, or death will be drawn from (electronic) patient chart.

- **Discontinuation of study participation**

On request a patient can withdraw from the study at any time, or when medically necessary, as judged by the Investigator. If a patient does not return for a scheduled visit, every effort should be made to contact the patient. In any circumstance, every effort should be made to document patient outcome if possible.
If a patient decides to withdraw from the study, he/she will be contacted in order to, if the patient agrees, obtain information about the reason(s) for discontinuation and any experienced adverse effect. The date and reason for the withdrawal will be recorded in the case record form.

- **Benefit of the study**

Potential benefits: Participating patients will be offered a clinical outpatient control, incl. an echocardiography three months after pPCI.

Disadvantage: In relation to the inflation of the blood pressure cuff temporary moderate pains in the treated arm might occur. Otherwise, the RIC has previously been proven to be without side effects.
An extra blood sampling of app. 15 ml will be drawn 72 hours after pPCI. A small but insignificant risk of local infection in relation to this is a risk.

- **Statistics**

  - **Power Calculations**

The primary combined endpoint of our clinical trial will be CV mortality and HHF, both heart failure (HF) during the index hospitalisation and re-hospitalisation for HF) at one year. In previous studies of STEMI patients undergoing pPCI, CV mortality at one year ranges from 5.8% to 16.7% depending on the call to balloon time (NIAP 2008 database, UK), TIMI blood flow (16) and the presence of coronary no-reflow (17). In the AMISTAD II study the event rate at 6 months in proximal left anterior descending (LAD) STEMI patients was: 11.3% CV mortality, 8.3% HHF (18). In another study, the incidence of HHF was 12.7% at one year post-PCI (16). Results from our own institution have revealed a 1-year total mortality of 9.4% and a cumulative risk of readmission with HF of 8% (1). Hence, we have chosen a primary event rate of 17.4%. To detect a 25% reduction in this primary endpoint in the RIC-treated group (from 17.4% to 13.0%), with a power of 80% and a significance level of 5%, a sample size of 1044 patients will be required for each trial arm (2088 in total). To account for failure of fulfilling entry criteria on arrival we plan to include 2300 patients.

  - **Pre-specified subgroup analyses**

We will undertake a limited number of subgroup analyses: age, gender, diabetes, duration from symptom onset to pPCI, and duration from end of RIC to pPCI. These subgroups will be analysed using interaction tests. These factors may influence the response to cardio-protective strategies such as RIC and pharmacological agents.

  - **Statistical analysis**

A detailed statistical analysis plan will be produced prior to un-blinding of any data. The primary analysis will be a comparison of the event rate one year after STEMI between RIC and control arms of the trial. Survival analyses will be used for CV mortality and HHF and other clinical endpoints. Areal under the curve will be used to elucidate the MI size. All patients will be included in the data analysis after the intention-to treat principle.

- **Project timetables and recruitment feasibility**

The total duration of the study will be 56 months:
1) 0-6 months; Study preparation
2) 6-42 months: Patient recruitment
3) 56-62 months: Study analysis. In order to recruit 2300 STEMI patients over 36 months, each of the centres will need to recruit 383 patients (11 patients/month), which equates to less than half of all the STEMI patients admitted to each centre during this time. Each of the recruiting centres provides 24 hours a day, 7 days a week pPCI service. The on-call cardiologists will recruit during office hours and out of office hours allowing non-stop recruitment.

- **Ethical issues**

The study protocol has to be approved by the regional ethics committee before initiation of the study. The study will be registered at the Danish Data Protection Agency.

The clinical trial will be registered on www.clinicaltrials.gov. Good clinical practice will be observed through the trial and we will respect the appropriate international, European and national legislation and guidelines.

The study will be monitored by the Data Monitoring Committee. Study investigators must report all unexpected events, independently of seriousness (adverse event (AE), serious adverse event (SAE)) to the safety group of the study. The study is not a pharmaceutical drug trial or a testing of new medical devices and therefore none of the reported events are to be considered as side-effects (adverse reaction (AR) or serious adverse reactions (SAR)).

The nominated sponsor of our research study will be University of Aarhus.
The study participants are during study covered under the law on patient insurance.

- **Data storing**

All study data are stored in an electronic CRF, where data are blinded and patients can be identified via a study identification number, only. In the medical record of the patient participation in the study will be recorded.
Data will be stored at the dept. of Cardiology, Aarhus University Hospital, Skejby for 15 years and subsequently the documents will be shredded.

- **Expected impacts listed in the work program**

RIC is low-cost intervention, which can be easily implemented as part of routine clinical care of STEMI patients undergoing pPCI. If successful, this project will be expected to improve clinical outcomes for STEMI patients by reducing the number of CV deaths and reducing the number of patients HHF at one year.

Positive as well as negative and inconclusive results will be widely disseminated through national and international scientific papers and conferences. The order of appearance of the scientific authors will be according to the included number of patients at the centre. Such that the centre including most patients will have the first authorship, followed by the second most including etc. Each centre will have one author per 300 included patients. Subsequently data managing authors (including data and safety committee members will be presented followed by pre-hospital collaborators). Writing authors Henrik Toft Sørensen and Hans Erik Bøtker will be second last and last respectively. Hans Erik Bøtker will be corresponding author. Other actively contributing collaborators (PCI-operators etc.) will be included as collaborating authors under the heading of the CONDI-2 investigators. This will secure inclusion on PubMed and other medical scientific databases.

The centres involved in recruiting the patients will not be involved in any competing studies, thereby maximising the feasibility of recruitment.

- **Financial standing**

The study is supported by The Danish Council for Strategic Research with Dkr. 19.6 kr. mill. and by The Danish Council for Independent Research, Medical Sciences with Dkr. 3.6 mill. The money is deposited on a bank account subjected to public current audit. None of the in the study implicated doctors or nurses have any financial benefit of the study.

- **Reference List**

(1) Terkelsen CJ, Jensen LO, Tilsted HH, Trautner S, Johnsen SP, Vach W, et al. Health care system delay and heart failure in patients with ST-segment elevation myocardial infarction treated with primary percutaneous coronary intervention: follow-up of population-based medical registry data. Ann Intern Med 2011 Sep 20;155(6):361-7.

(2) Kaltoft A, Bottcher M, Nielsen SS, Hansen HH, Terkelsen C, Maeng M, et al. Routine thrombectomy in percutaneous coronary intervention for acute ST-segment-elevation myocardial infarction: a randomized, controlled trial. Circulation 2006 Jul 4;114(1):40-7.

(3) Kelbaek H, Terkelsen CJ, Helqvist S, Lassen JF, Clemmensen P, Klovgaard L, et al. Randomized comparison of distal protection versus conventional treatment in primary percutaneous coronary intervention: the drug elution and distal protection in ST-elevation myocardial infarction (DEDICATION) trial. J Am Coll Cardiol 2008 Mar 4;51(9):899-905.

(4) Kelbaek H, Thuesen L, Helqvist S, Clemmensen P, Klovgaard L, Kaltoft A, et al. Drug-eluting versus bare metal stents in patients with st-segment-elevation myocardial infarction: eight-month follow-up in the Drug Elution and Distal Protection in Acute Myocardial Infarction (DEDICATION) trial. Circulation 2008 Sep 9;118(11):1155-62.

(5) Svilaas T, Vlaar PJ, van der Horst IC, Diercks GF, de Smet BJ, van den Heuvel AF, et al. Thrombus aspiration during primary percutaneous coronary intervention. N Engl J Med 2008 Feb 7;358(6):557-67.

(6) Yellon DM, Hausenloy DJ. Myocardial reperfusion injury. N Engl J Med 2007 Sep 13;357(11):1121-35.

(7) Hausenloy DJ, Yellon DM. The mitochondrial permeability transition pore: its fundamental role in mediating cell death during ischaemia and reperfusion. J Mol Cell Cardiol 2003 Apr;35(4):339-41.

(8) Botker HE, Kharbanda R, Schmidt MR, Bottcher M, Kaltoft AK, Terkelsen CJ, et al. Remote ischaemic conditioning before hospital admission, as a complement to angioplasty, and effect on myocardial salvage in patients with acute myocardial infarction: a randomised trial. Lancet 2010 Feb 27;375(9716):727-34.

(9) Munk K, Andersen NH, Schmidt MR, Nielsen SS, Terkelsen CJ, Sloth E, et al. Remote Ischemic Conditioning in Patients With Myocardial Infarction Treated With Primary Angioplasty: Impact on Left Ventricular Function Assessed by Comprehensive Echocardiography and Gated Single-Photon Emission CT. Circ Cardiovasc Imaging 2010 Nov;3(6):656-62.

(10) Przyklenk K, Bauer B, Ovize M, Kloner RA, Whittaker P. Regional ischemic 'preconditioning' protects remote virgin myocardium from subsequent sustained coronary occlusion. Circulation 1993 Mar;87(3):893-9.

(11) Schmidt MR, Smerup M, Konstantinov IE, Shimizu M, Li J, Cheung M, et al. Intermittent peripheral tissue ischemia during coronary ischemia reduces myocardial infarction through a KATP-dependent mechanism: first demonstration of remote ischemic perconditioning. Am J Physiol Heart Circ Physiol 2007 Apr;292(4):H1883-H1890.

(12) Kharbanda RK, Mortensen UM, White PA, Kristiansen SB, Schmidt MR, Hoschtitzky JA, et al. Transient limb ischemia induces remote ischemic preconditioning in vivo. Circulation 2002 Dec 3;106(23):2881-3.

(13) Hausenloy DJ, Mwamure PK, Venugopal V, Harris J, Barnard M, Grundy E, et al. Effect of remote ischaemic preconditioning on myocardial injury in patients undergoing coronary artery bypass graft surgery: a randomised controlled trial. Lancet 2007 Aug 18;370(9587):575-9.

(14) Cheung MM, Kharbanda RK, Konstantinov IE, Shimizu M, Frndova H, Li J, et al. Randomized controlled trial of the effects of remote ischemic preconditioning on children undergoing cardiac surgery: first clinical application in humans. J Am Coll Cardiol 2006 Jun 6;47(11):2277-82.

(15) Hoole SP, Heck PM, Sharples L, Khan SN, Duehmke R, Densem CG, et al. Cardiac Remote Ischemic Preconditioning in Coronary Stenting (CRISP Stent) Study: a prospective, randomized control trial. Circulation 2009 Feb 17;119(6):820-7.

(16) Zalewski J, Nycz K, Przewlocki T, Durak M, Cul M, Zajdel W, et al. Evolution of myocardial perfusion during primary angioplasty in spontaneously reperfused infarct-related artery: impact on long-term clinical outcomes and left ventricular function recovery. Int J Cardiol 2011 Feb 17;147(1):25-31.

(17) Ndrepepa G, Tiroch K, Keta D, Fusaro M, Seyfarth M, Pache J, et al. Predictive factors and impact of no reflow after primary percutaneous coronary intervention in patients with acute myocardial infarction. Circ Cardiovasc Interv 2010 Feb 1;3(1):27-33.

(18) Kloner RA, Forman MB, Gibbons RJ, Ross AM, Alexander RW, Stone GW. Impact of time to therapy and reperfusion modality on the efficacy of adenosine in acute myocardial infarction: the AMISTAD-2 trial. Eur Heart J 2006 Oct;27(20):2400-5.

CONDI-2 Study Protocol

Effect of Remote Ischaemic Conditioning on clinical outcomes in ST-elevation myocardial infarction patients undergoing primary Percutaneous Coronary Intervention: A multicentre randomised controlled clinical study

Acronym: CONDI 2

Multinational investigator-driven, multi-centre, randomised, controlled, single-blind (Outcomes Assessor), parallel assignment, efficacy prospective clinical trial to investigate whether RIC can improve clinical outcomes (cardiac mortality and hospitalisation for heart failure) at one year in STEMI patients undergoing primary PCI.

Project Identification: Condi 2
Protocol Version: Version 10.6 January 2017
Sponsor: Danish Strategic Research Council

Coordinating Principal Investigators:
Hans Erik Bøtker, senior consultant, MD, DMSci, Professor Cardiology,
Department of Cardiology Aarhus, University Hospital Skejby, DK-8200 Aarhus N,
Denmark, Telephone +4578452026

The clinical study will be conducted, and essential documentation archived, in compliance with UCR SOPs and standards, which incorporate the requirements of the ICH Guideline for Good Clinical Practice and EN ISO 14155:2011(E).

- **Synopsis**

**Name of Sponsor Investigator**:<br>Hans Erik Bøtker, senior consultant, MD, DMSci, Professor Cardiology, Department of Cardiology, Aarhus University Hospital Skejby, DK-8200 Aarhus N, Denmark
**Name of Investigational Medical Device**: The CellAegis automated cuff
**Title of Study**:<br>Effect of Remote Ischaemic Conditioning on clinical outcomes in ST-elevation myocardial infarction patients undergoing primary Percutaneous Coronary Intervention: A multinational multicentre randomised controlled clinical study
**Trial Management Groups (TMG)**:<br>Coordinating Principal Investigator:<br>Hans Erik Bøtker, senior consultant, MD, DMSci, Professor Cardiology, Department of Cardiology, Aarhus University Hospital Skejby, DK-8200 Aarhus N, Denmark<br><br>**Trial Steering Committee (TSC)**:<br>Hans Erik Bøtker (Aarhus University Hospital, Skejby), Michael Rahbek Schmidt (Aarhus University Hospital), Thomas Engstrøm (Rigshospitalet), Jan Ravkilde (Aalborg Sygehus), Lisette Okkels Jensen (Odense University Hospital), Derek Hausenloy (The Hatter Cardiovascular Institute, London), Henrik Toft Sørensen (Aarhus University Hospital), Erika Frischknecht Christensen (Pre-Hospital, Aarhus), U Kristine Møller (Aarhus University Hospital, Skejby), Freddy Lippert (Pre-Hospital, Copenhagen), Borja Ibañez (Hospital Clínico San Carlos de Madrid)<br><br>**Data Monitoring Committee (DMC), trial safety committee**:<br>Denmark: Henning Kelbæk (Roskilde Hospital), Jakob Hjort (data manager), Morten Madsen (Aarhus University),<br>Spain: Hector Bueno (Universidad Complutense de Madrid),<br><br>**Trial endpoints validation committee**:<br>Denmark: Chairman Kristian Thygesen (Aarhus University Hospital), Henning Rud Andersen (Aarhus University Hospital),<br>Spain: Dr Rodrigo Fernandez-Jimenez (National Centre for Cardiovascular Research), TBD<br>Serbia: TBD, TBD
**Study Centres:**<br>• Department of Cardiology, Aarhus University Hospital, Skejby, DK-8200 Aarhus N, Denmark<br>• The Heart Centre, Rigshospitalet, 2100 Copenhagen Ø, Denmark

- The Heart Centre, Aalborg Sygehus, 9100 Aalborg, Denmark
- Department of Cardiology, Odense University Hospital, 5000 Odense, Denmark
- Department of Cardiology, Clinical Center of Serbia, Beograd, Serbia
- Department of Cardiology, Hospital Universitario Central de Asturias, Oviedo, Spain
- Hatter Institute, University of London, UK

**Planned study period**: 2013-2018	**Phase of development**: Improve routine clinical care of STEMI patients

Objectives:
To determine whether Remote Ischaemic Conditioning (RIC) improves clinical outcomes cardiac mortality and hospitalisation for heart failure (HHF)) at one year in ST-elevation myocardial infarction (STEMI) patients undergoing primary Percutaneous Coronary Intervention (pPCI).

Diagnosis:
ST-elevation myocardial infarction (STEMI)

Methodology:
A multinational and -multicentre investigator-driven, prospective randomised, controlled, single-blind, parallel assignment, clinical efficacy trial.

Randomisation and allocation procedure:
Eligible patients will be randomised (via a secure web-site) prior to pPCI, preferably in the ambulance or on arrival at the hospital.
The assessor of clinical outcomes will be blinded to the treatment allocation.

Number of subjects (planned):
4.300 patients

Inclusion criteria:

- Male and female patients (>18 years old) presenting with chest pain within 12 hours, lasting for more than 30 minutes
- Suspected STEMI (ST-elevation at the J-point in two contiguous leads with the cut-off points: ≥0.2 millivolt (mV) in men or ≥0.15 mV in women in leads V2-V3 and/or ≥0.1 mV in other leads).
- Informed consent obtained
- Life expectancy of more than 1 year

Exclusion criteria:

- Previous by-pass surgery
- Myocardial infarction (MI) or treatment with thrombolysis within 30 days
- New left bundle branch block
- Patients treated with cooling
- Paresis of upper limb

- A-V-shunt

Criteria for evaluation:

Primary clinical endpoint:

- Cardiac mortality and hospitalisation for heart failure at one year.

Secondary clinical endpoints:

- Myocardial infarct size at day 3 (72 hour area under curve serum creatinine kinase)
- Left ventricular function on day three and three-six months post pPCI (Echocardiography)
- Re-infarction or recurrent infarction, stroke and unplanned coronary revascularisation at one year (New admission)
- Quality of life at 6-8 weeks and 12 months (EuroQol EQ-5D-5L)

Power Calculations:

To detect a 25% reduction in cardiac mortality in the RIC-treated group (from 8% to 6%%), with a power of 80% and a significance level of 5%, a sample size of 2,553 patients will be required for each trial arm (5106 in total). To account for and failure of fulfilling entry criteria on arrival we plan to include 5,700 patients.

Pre-specified subgroup analyses: A limited number of subgroup analyses will be undertaken: age, gender, diabetes, chest pain to PCI time, and RIC to PCI time. These subgroups will be analysed using interaction tests.

Statistical analysis: A comparison of the event rate one year after STEMI between RIC and control arms of the trial. Survival analyses techniques will be used for CARDIAC death and HHF and other clinical endpoints.

Content

1 Synopsis 99
2 Abbreviations 103
3 Concept 104
4 Hypothesis 105
5 Objectives 105
5.1 Overall primary objective 105
5.2 Secondary objectives: 105
6 Endpoints 105
6.1 Primary clinical endpoint 105
6.2 Secondary clinical endpoints 105
6.3 Primary endpoints are defined according to the following standard operating procedures: 106
6.4 Secondary endpoints are defined according to the following standard operating procedures: 108
7 S/T methodology and associated work plan 111
7.1 Trial Design: 111
7.2 Setting 111
7.3 Target population 111
8 Study progress 112
8.1 Patient information 112
8.2 Procedures and treatment 112
8.3 Discontinuation of study participation 113
9 Benefit of the study 114
10 Statistics 114
10.1 Power Calculations 114
10.2 Interim analyses 115
10.3 Pre-specified subgroup analyses 115
10.4 Statistical analysis 115
11 Project timetables and recruitment feasibility 115
12 Study status 116
13 Ethical issues 116
13.1 Data storing 116
13.2 Expected impacts listed in the work program 116
14 Financial standing 117
15 Reference List 118

- **Abbreviations**

AE	Adverse event
AR	Adverse reaction
CABG	Coronary artery bypasses graft
CHD	Coronary heart disease
DMC	Data Monitoring Committee and Endpoints validation committee
CV	Cardiovascular
ECCO	Echocardiography
ECG	Electrocardiogram
e-CRF	Electronic-Case Report Form
HF	Heart failure
HHF	Hospitalisation for heart failure
LAD	Proximal left anterior descending
MI	Myocardial infarct
mPTP	Mitochondrial permeability transition pore
PCI	Percutaneous coronary intervention
pPCI	Primary percutaneous coronary intervention
RIC	Remote Ischaemic Conditioning
SAE	Serious adverse event
SAR	Serious adverse reaction
STEMI	ST-elevation myocardial infarction
TMG	Trial Management Group
TSC	Trial Steering Committee

- **Concept**

Coronary heart disease (CHD) is the leading cause of death in Denmark and Europe, accounting for 1.92 million deaths in Europe per year (2008 European Cardiovascular Disease Statistics): over one in five men (21%) and one in five women (22%) die from CHD. Therefore, novel therapeutic strategies are urgently required to improve clinical outcomes in patients with CHD and further to reduce the health-care and non-health-care costs of CHD. One area this needs to be achieved is in patients presenting with a ST-elevation myocardial infarction (STEMI). The current state-of-the-art treatment for patients presenting with a STEMI is timely myocardial reperfusion using primary percutaneous coronary intervention (pPCI) to restore blood flow in the infarct-related coronary artery in the shortest possible time. However, STEMI patients undergoing percutaneous coronary intervention (PCI) have a significant mortality and morbidity at one year, with up to 17.4% of patients dying from a cardiac cause or being hospitalised from heart failure (HHF) (1).

Recent advances in this area of research have focused on maintaining the patency of the infarct-related coronary artery and on securing perfusion by preventing distal embolization (2-5).

New treatment strategies beyond the opening of the artery are required to protect the myocardium itself from the detrimental effects of ischaemia-reperfusion injury. Lethal myocardial reperfusion injury has been attributed to oxidative stress, calcium overload, cardiomyocyte hypercontracture and mitochondrial dysfunction induced by the opening of the mitochondrial permeability transition pore (mPTP) (6;7).

Both pre-clinical and recent clinical studies have demonstrated that treatment strategies such as Remote Ischaemic Conditioning (RIC) applied at the time of myocardial reperfusion can reduce myocardial infarct size, confirming the existence of myocardial reperfusion injury (6). In this respect, RIC has been shown to limit myocardial infarct size and preserve cardiac function in recent proof-of-concept clinical studies in STEMI patients undergoing pPCI (8;9). Cardioprotection by RIC is most commonly achieved by manual or automatic inflation of a blood pressure cuff to induce four 5-minute cycles of limb ischaemia and reperfusion (10;11), a technique that was first pioneered by our network allies at Aarhus University Hospital and University College London (12). Based on these studies, we have demonstrated that RIC applied in the ambulance during transport to the pPCI unit reduced MI size in STEMI patients undergoing pPCI (8). Subsequently, it has been demonstrated that RIC can reduce myocardial injury in patients undergoing coronary artery bypass graft (CABG) surgery (13), in children undergoing corrective cardiac surgery (14) and in patients with stable angina undergoing elective PCI (15).
However, whether this virtually cost-free non-pharmacological and non-invasive therapeutic strategy can actually improve clinical outcomes in STEMI patients has never been investigated in a randomized controlled clinical trial.

- **Hypothesis**

RIC followed by pPCI improves clinical outcomes in STEMI patients when compared to STEMI controls undergoing standard pPCI evaluated one year post PCI.

- **Objectives**

○ **Overall primary objective**

- To determine whether RIC improves clinical outcomes (cardiac mortality and hospitalisation (in-hospital or outpatient clinic) for heart failure (HHF)) at one year in STEMI patients undergoing pPCI.

○ **Secondary objectives:**

- To determine, in the pre-specified subgroups, whether age, gender, diabetes, and duration of chest pain to PCI influence the response to RIC.
- To determine whether RIC preserves left ventricular function measured by echocardiography after three day and three-six months post pPCI
- To determine whether a correlation between biomarkers, patient susceptibility to ischaemia-reperfusion injury and the treatment strategies of RIC can be found. Measured as area under the curve 72 hours post pPCI
- To trial the CellAegis automated cuff in its largest clinical study to date.
- Quality of life at 6-8 weeks and 12 months (EuroQol EQ-5D-5L)

All these outcomes are relevant to STEMI patients and have the potential to change clinical practice.

- **Endpoints**

An independent end-point committee will adjudicate primary and secondary clinical endpoints.

○ **Primary clinical endpoint**

- Cardiac mortality and HHF at one year

○ **Secondary clinical endpoints**

- Myocardial infarct size at day three (72 hour area under curve serum creatinine kinase). Furthermore, TnT in a subset of patients (Aarhus, Aalborg, Belgrade)
- Left ventricular function on day three and three months post pPCI (Echocardiography)
- Myocardial re-infarction/recurrent, stroke and revascularisation at one year
- Persisting ST-segment elevation on ECG 90 min. after primary PCI

The source data

The source data for the events are print-outs from the electronic medical record of each patient. Furthermore, documents from “The West-Danish heart database” or “The East-Danish Heart database”. Data documentation in Spain and Serbia are prints of the electronic or paper medical record of each patient.

- **Primary endpoints are defined according to the following standard operating procedures:**

Death:

Validation categories: Cardiac Death or Non-cardiac death

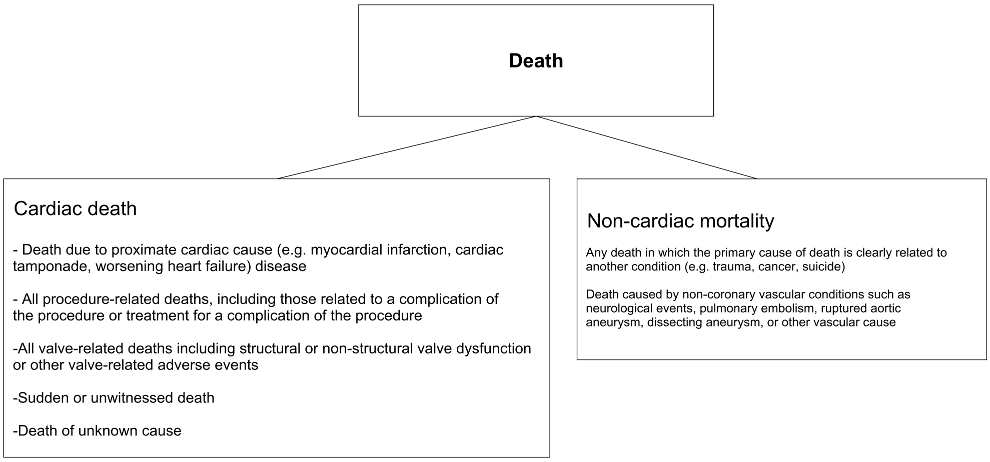

Modified from the "Updated standardized endpoint definitions for transcatheter aortic valve implantation: the Valve Academic Research Consortium-2 consensus document. European Heart Journal (2012) 33, 2401-2418

Heart failure

Validation categories: HHF; Non-HHF

- During index admission, New admission or unscheduled outpatient clinic visit **
- Symptoms, signs and results of diagnostic test need to be assessed by a medical doctor.

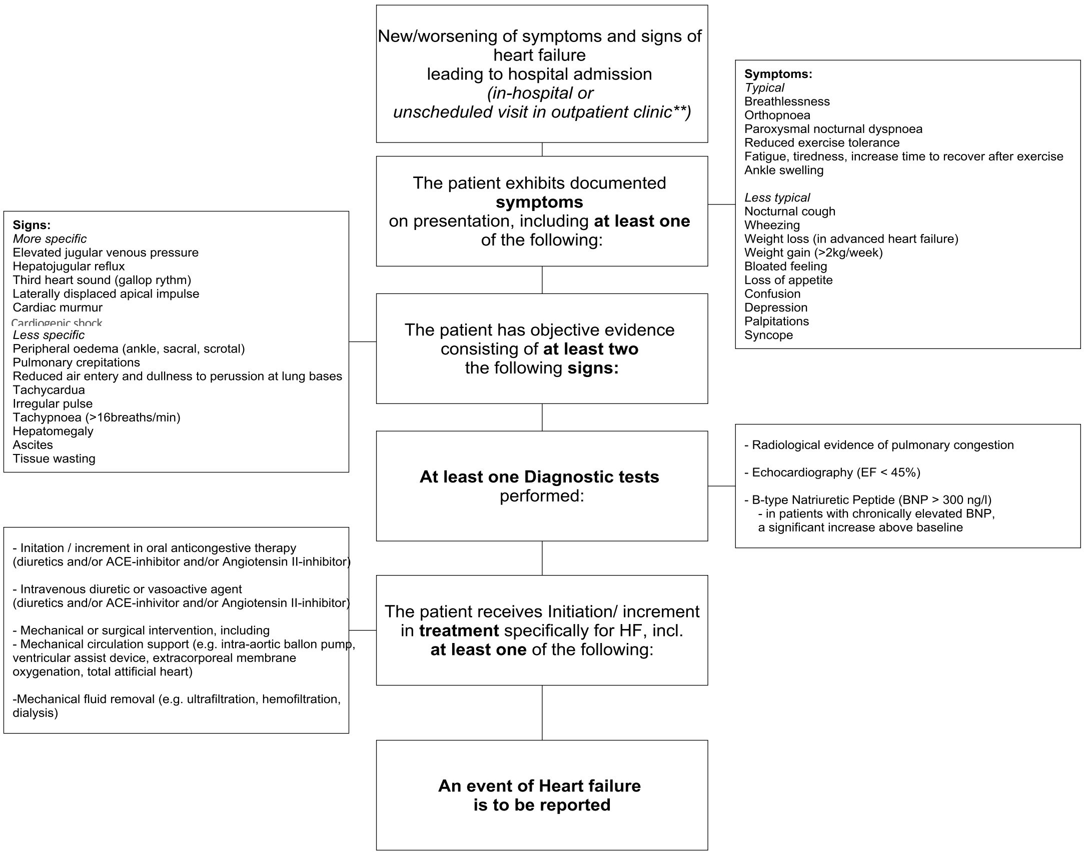

*According to ESC guidelines for diagnose and treatment of acute and chronic heart failure 2012. European Heart Journal (2012) 33, 1787-1847 and
"2014ACC/AHA Key Data Elements and Definitions for Cardiovascular Endpoint Events in Clinical Trial". Circulation.2015;132:302-361

Please note:

- Patients with cardiogenic shock will fulfil the definition of heart failure.
- That acute heart failure developed prior to randomisation should not be considered an event. Neither chronic heart failure diagnosed prior to randomisation.
- That initiation or increment of these drugs due to hypertension is NOT an event. The independent end-point committee will subsequently confirm the diagnosis.

Encounter type:

In-hospital: An event where the patient is admitted to the hospital with a primary diagnosis of HF where the length of stay is at least 24 h (or extends over a calendar date if the hospital admission and discharge times are unavailable), where the patient exhibits new or worsening symptoms of HF on presentation, has objective evidence of new or worsening HF, and receives initiation or intensification of treatment specifically for HF.

Unscheduled outpatient clinic: An event where the patient has an urgent, unscheduled office/practice/ED visit for a primary diagnosis of HF but is not admitted to the hospital, where the patient exhibits new or worsening symptoms of HF on presentation, has objective evidence of new or worsening HF, and receives initiation intensification of treatment specifically for HF, with the exception that changes to oral diuretic therapy do not qualify as initiation or intensification of or treatment.

- **Secondary endpoints are defined according to the following standard operating procedures:**

For the secondary endpoint, we are interested in those MIs occurring outside the setting of any future elective PCI procedure.

SOP for Diagnostic and clinical indications of MI *, More than 48 hours after index pPCI Event report in Condi 2

Validation categories: MI ; No MI

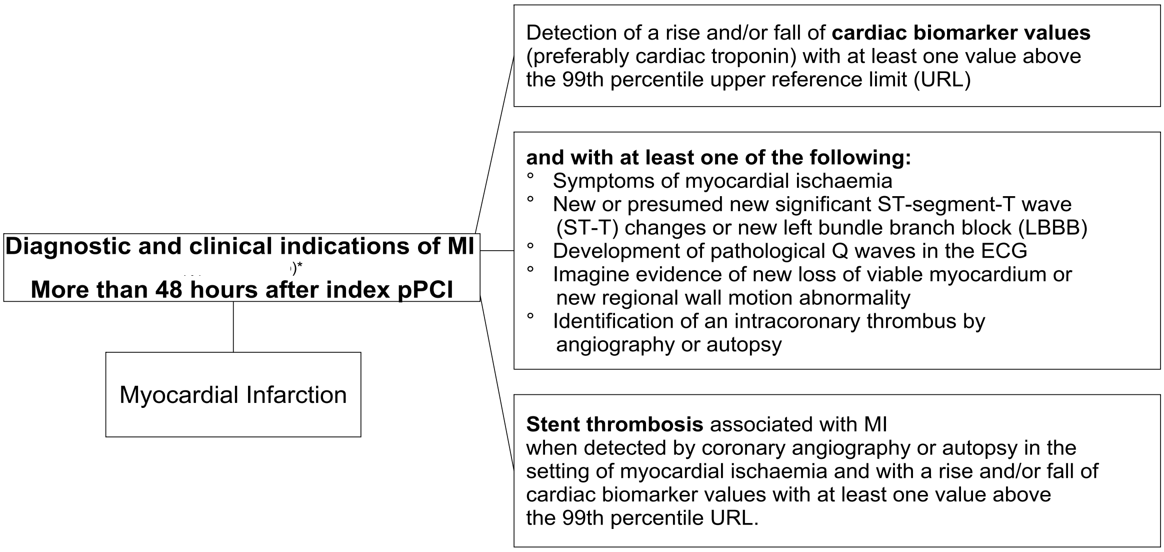

*Modified from: ”Third universal classification of myocardial infarction”. European Heart Journal (2012) 33, 2251-2567and “2014ACC/AHA Key Data Elements and Definitions for Cardiovascular Endpoint Events in Clinical Trial”. Circulation.2015;132:302-361

SOP for Stroke Event report in Condi 2

Validation categories: Definite ischaemic stroke; Haemorrhagic stroke; Transient ischaemic attack; No evidence of stroke

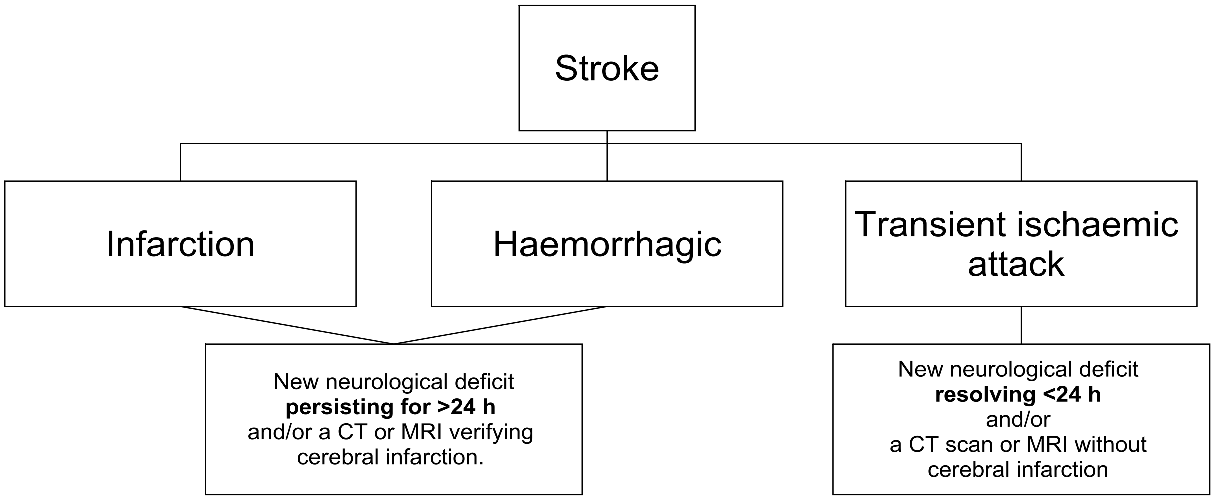

*According to "2014ACC/AHA Key Data Elements and Definitions for Cardiovascular Endpoint Events in Clinical Trial". Circulation.2015;132:302-361

<u>Evaluation of the electrocardiogram</u>:

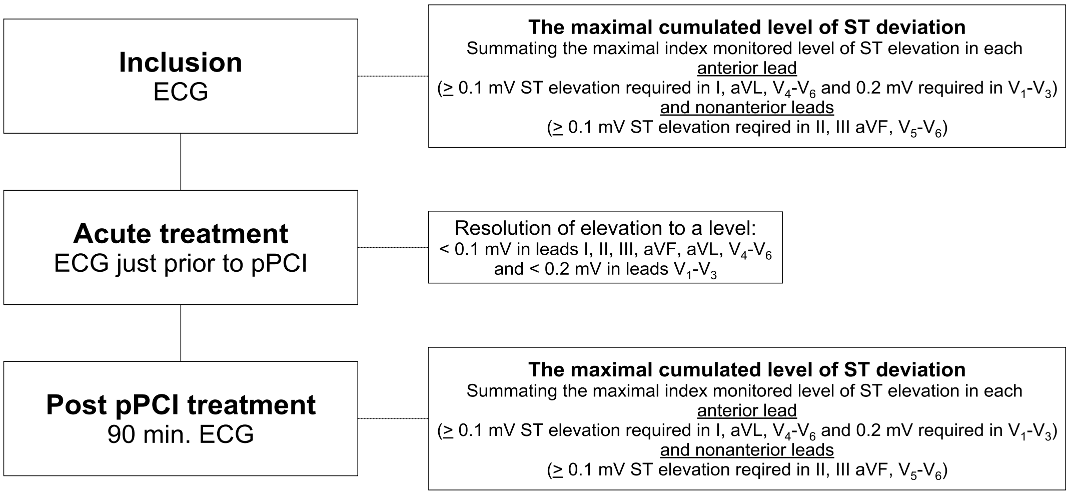

<u>*SOP for clinical indicated coronary REVASCULARISATON (not scheduled at index event) report in Condi 2*</u>*:*

Validation categories: Yes or No

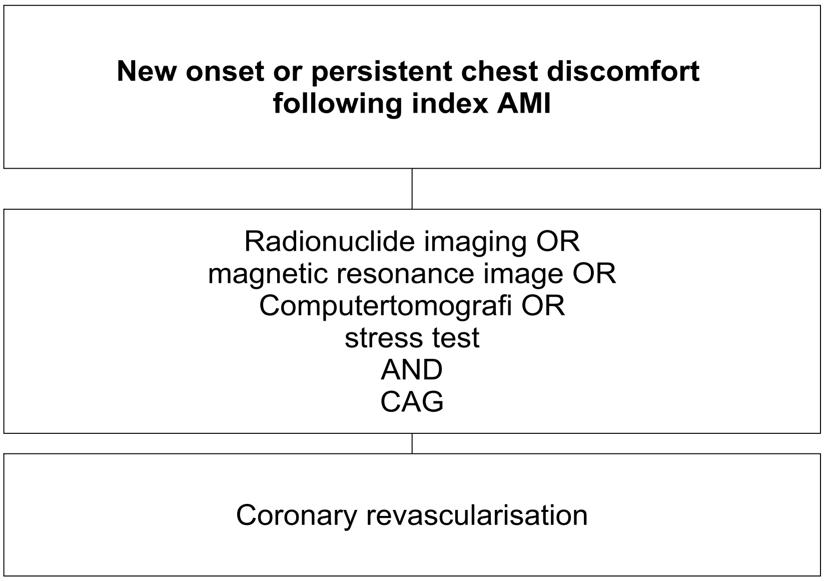

*Modified from: ”Third universal classification of myocardial infarction”. European Heart Journal (2012) 33, 2251-2567and “2014ACC/AHA Key Data Elements and Definitions for Cardiovascular Endpoint Events in Clinical Trial”. Circulation.2015;132:302-361

SOP for Procedure-related AMI report in Condi 2 (not part of secondary outcome measures)

Validation categories: Yes or No

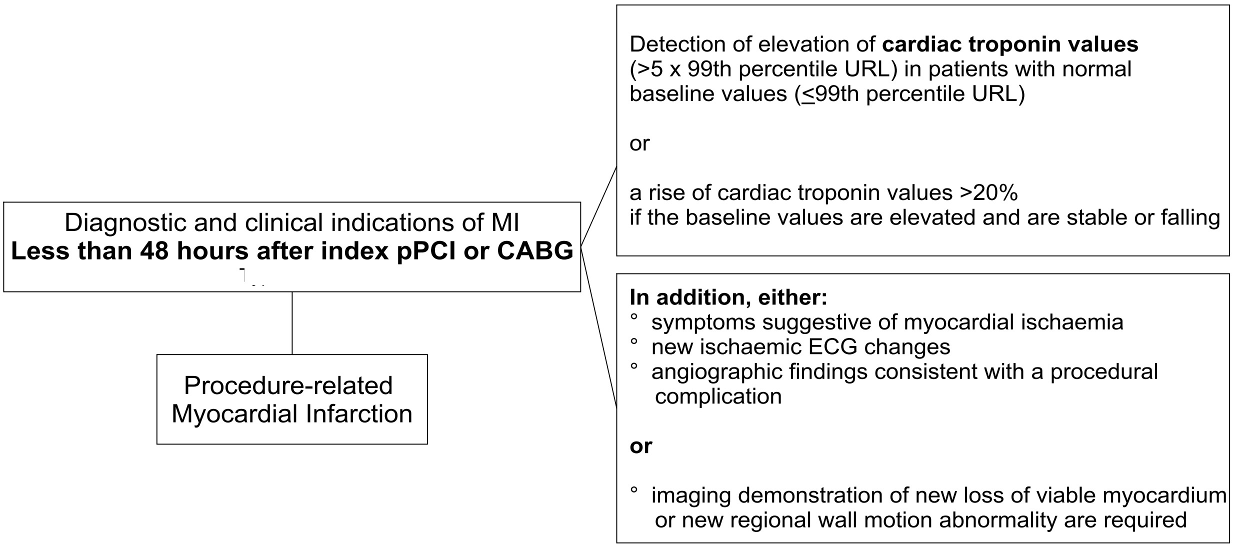

*Modified from: ”Third universal classification of myocardial infarction”. European Heart Journal (2012) 33, 2251-2567and “2014ACC/AHA Key Data Elements and Definitions for Cardiovascular Endpoint Events in Clinical Trial”. Circulation.2015;132:302-361

- **S/T methodology and associated work plan**

○ **Trial Design:**

Multinational investigator-driven, multi-centre, randomised, controlled, single-blind (Outcomes Assessor), parallel assignment, prospective clinical efficacy trial to investigate whether RIC can improve clinical outcomes (cardiac mortality and HHF) at one year in STEMI patients undergoing pPCI.

○ **Setting**

In all 5700, STEMI patients will be included.
Four centres with on-site facilities for pPCI in Denmark will recruit app. 1,800 STEMI patients in total over a 36-month period.
Centres in Asturias, Spain, and Belgrade, Serbia will recruit app. 800 STEMI patients in total over a 36-month period.
An alligned sister study in the ERICCA-collaboration (ERIC-pPCI) including 30 primary PCI centres in the UK will include 3,000 patients.
Data collection and analysis will be undertaken through the Department of Cardiology and Department of Clinical Epidemiology, Aarhus University Hospital Skejby Clinical Trials Unit.

○ **Target population**

Possible study candidates are patients with putative STEMI acute admitted to one of the heart centres. There will be no advertising for patients. A total of 5,700 patients are to be included over a 36 months period.

**Inclusion criteria for recruitment**	**Exclusion criteria for recruitment**
Male and female patients (>18 years old) presenting with chest pain for more than 30 minutes	Previous by-pass surgery
Putative STEMI<br>(ST-elevation at the J-point in two contiguous leads with the cut-off points: ≥0.2 mV in men or ≥0.15 mV in women in leads V2-V3 and/or ≥0.1 mV in other leads)	MI or treatment with thrombolysis within 30 days
Informed consent obtained	New left bundle branch block
Life expectancy of more than 1 year	Patients treated with cooling
	Paresis of upper limb
	A-V-shunt

- **Study progress**
  - **Patient information**

The patient will be informed and treated according to the national and international guidelines for Good Clinical Practice and protected under the Act concerning the processing of personal data and health law.
The intervention is performed in the acute phase. Consequently, in addition to the ordinary written patient information (A) a short written information (B) is given in the ambulance (one A4 sheet). Because the nature of the disease and the intervention are acute, the usual requirements about a 24-hour time for consideration and discussion with a lay representative cannot be met. The inherent acute nature of the study necessitates circumvention of these usual requirements as it has previously been done in comparable acute studies. This approach is reasonable because the intervention is without any known risk and because the intervention has potential benefit for the individual patient.

The admitting ambulance doctor, the doctor at the receiving hospital, or the paramedic will orally inform the patient based on the short written information (B) and hand out the written informations (A+B). After oral and written informed consents are, obtained the patient will sign the agreement form and be randomised via a secure web site to pPCI with or without RIC by the doctor on duty at one of the receiving hospitals. Computer-generated blocked randomisation lists, stratified by centre, will be prepared in advance of the study.
After information is given in the acute phase, the patient does not have much time for reflection before signing the informed consent form. Therefore, a study nurse will give the patient additional oral information after the acute phase by or the doctor performing the pPCI based on the full written information (A). Both during the first and second stage of information it will be emphasised that the patient has the right to withdraw his/her informed consent at any time.

  - **Procedures and treatment**

Control group: Will be treated according to standard procedures in the ambulance.

Intervention group, RIC group: The CellAegis auto RIC (automated blood pressure cuff) will be placed on the upper arm and inflated to 200mmHg for 5 minutes and then deflated for 5 minutes, a programmed cycle which is repeated 4 times in total (therefore the total length of the RIC protocol is 40 minutes). If the initial systolic blood pressure is >175mmHg, a manual cuff will used to and inflated to 25mmHg above the systolic blood pressure. In recruiting centres where randomisation occurs at the hospital or in cases with short transportation time, the RIC protocol will continue during PCI until successful or until immediately before reperfusion.

Further, the patients will be treated according to standard procedures in the ambulance.

	Acute intervention		Index event	Follow up
Randomisation	Intervention	Control		
Blood samples [1]			X	
e-CRF [2]			X	X
Echocardiography [3]			X	X
Electrocardiography [4]	X		X[4]	
Quality of life (EuroQol EQ-5D-5L)[5]	X			X

[1] Blood samples for Ck and TnT drawn acute, 6-8 (±4), 24 (±8) and 48-72 (+6) hours post-PCI. Samples will be analyzed immediately.

[2] Research assistant complete the electronic case record form. Information regarding re-hospitalisation or death will be drawn from (electronic) patient chart.

[3] Echocardiography (sonogram of the heart, performed at day one to three and three to six months after-pPCI)

[4] Electrocardiography will be measure acute (admitting ECG), just prior to pPCI and 90 minutes post-PCI

[5] Quality of life (EuroQol EQ-5D-5L) at 6-8 weeks and 12 months via printed copy or via internet based questionnaire.

pPCI incl. the use of stents and antithrombotic regimens will be performed according to standard procedures at the treating hospital.
Blood samples will be drawn according to standard routine; no extra biomarkers will be measured. An extra sample of 15 ml will be drawn 72 H post pPCI. All blood samples will be analysed immediately. We will not establish a bio bank.

In Denmark, we will draw information regarding re-hospitalisation for hearth failure, AMI, revascularisation, or death from the (electronic) patient chart.

The Quality of life questionnaire (EuroQol EQ-5D-5L) will be performed as a sub-study on app. 400 patients. The patients will after the acute pPCI treatment be asked for the capacity to fill in the questionnaire via a printed copy or via a secured individual internet based access.

- **Discontinuation of study participation**

On request, a patient can withdraw from the study at any time, or when medically necessary, as judged by the Investigator. If a patient does not return for a scheduled visit, every effort should be made to contact the patient. In any circumstance, every effort should be made to document patient outcome if possible.
If a patient decides to withdraw from the study, he/she will be contacted in order to, if the patient agrees, obtain information about the reason(s) for discontinuation and any experienced adverse effect. The date and reason for the withdrawal will be recorded in the case record form.

- **Benefit of the study**

Potential benefits: Participating patients will be offered a clinical outpatient control, incl. echocardiography, three months after pPCI.

Disadvantage: In relation to the inflation of the blood pressure cuff temporary moderate pain in the treated arm might occur. Otherwise, RIC has previously been proven to be without side effects.
An extra blood sampling of app. 15 ml will be drawn 72 hours after pPCI. A small but insignificant risk of local infection in relation to this is a risk.

- **Statistics**
  - **Power Calculations**

Primary combined clinical endpoint
The primary combined endpoint will be cardiac death and HHF at 12 months in all-comer STEMI patients. Importantly, the CONDI-2 trial will also be adequately powered to detect an effect of RIC on clinical outcomes in the higher-risk proximal LAD STEMI patient subgroup. The reason for this is that previous clinical trials have suggested that the STEMI patients most likely to benefit from a cardioprotective intervention administered as an adjunct to pPCI, are those presenting with a proximal LAD STEMI. These endpoints have been selected as they are the most relevant clinical endpoints on which RIC, an intervention which protects cardiomyocytes against acute IRI, is most likely to impact on. According to the UK NIAP 2008 database, cardiac mortality at 12 months ranged from 5.8%-16.7% depending on the call to balloon time, with the overall 12 month death rate of 8.7% for all pPCI patients. Results from our own institution have revealed a 1-year total mortality of 9.4% and a cumulative risk of readmission with HF of 8% (1). In another clinical study, the incidence of HHF was 12.7% at 12 months post-pPCI (18). The previous clinical data include all patients presenting with a STEMI. We have based our power calculations on these published series, accounting for the marked improvements in clinical outcomes in the contemporary era by using much more conservative event rates.

All STEMI patients: As a conservative estimate we will use a combined cardiac death and HHF event rate of 8% at 12 months for all-comer STEMI patients. In the CONDI-2 study we estimate the effect size to be a 25% relative reduction in the event rate. The rationale for this is based on the reductions in MI size observed in proof-of-concept clinical studies in which RIC and related therapeutic interventions such as ischemic postconditioning which have reported 40-50% reductions in MI size. To demonstrate a 25% reduction in the primary composite endpoint in the RIC-treated group (from 8.0% to 6%), with 80% power and at the 5% significance level, would require 2553 patients per treatment arm which equates to 5106 patients in total. Therefore, we will need to recruit 5,700 all-comer STEMI patients (allowing for a 10% drop-out rate at 12 months) between UK and Denmark (2,600 STEMI patients in the Denmark, Serbia and Spain and 3,000 STEMI patients in UK).

Proximal LAD STEMI: Patients presenting with the larger proximal LAD STEMI have worse clinical endpoints. In the AMISTAD II study (2,118 patients), which investigated the effect of adenosine as an adjunct to pPCI, only recruited LAD STEMI patients, and in these patients the 6-month primary combined endpoint was 19.6% (11.3% cardiac death, 8.3% HHF) (18). As a conservative estimate we will use a combined cardiac death and HHF event rate of 15% at 12 months for proximal LAD STEMI patients. To demonstrate a 30% reduction in the primary composite endpoint in the RIC-treated group (from 15.0% to 10.5%), with 80% power and at the 5% significance level, would require 862 patients per treatment arm which equates to 1,724 patients in total (862 proximal LAD STEMI patients each in the UK and Denmark). Therefore, assuming that about 45-50% of all STEMI patients present with a proximal LAD STEMI, in order to recruit 1,724 proximal LAD STEMI patients, we will need to recruit about 4,300 all STEMI patients (allowing for a 5% drop-out rate at 12 months) between UK, Serbia, Spain, and Denmark.

- **Interim analyses**

We will perform an interim analysis for evaluation of the actual event rate after inclusion of 50% of the patients. If this shows lower event rate than expected, a new sample size calculation will be performed. Furthermore, we will perform an independent safety analysis after inclusion of 50% of the patients.

- **Pre-specified subgroup analyses**

We will undertake a limited number of subgroup analyses: age, gender, diabetes, duration from symptom onset to pPCI, and duration from end of RIC to pPCI. These subgroups will be analysed using interaction tests. These factors may influence the response to cardio-protective strategies such as RIC and pharmacological agents.

- **Statistical analysis**

We will produce a detailed statistical analysis plan prior to un-blinding of any data. The primary analysis will be a comparison of the event rate one year after STEMI between RIC and control arms of the trial. We will use survival analyses for cardiac mortality, HHF, and other clinical endpoints. Further, we will use area under the curve to assess the MI size. All patients will be included in the data analysis after the intention-to-treat principle.

- **Project timetables and recruitment feasibility**

The total duration of the study will be 56 months:
1) 0-6 months: Study preparation
2) 6- 48 months: Patient recruitment
3) 56-62 months: Study analysis. In order to recruit 5,700 STEMI patients over 42 months, 136 patients are to be included per months. Each of the recruiting centres provides 24 hours a

day, 7 days a week pPCI service. The on-call cardiologists will recruit during office hours and out of office hours allowing non-stop recruitment.

- **Study status**

Until now, (medio January 2017), 1,823 patients are included in the study. Of which 1,382 are included in Denmark, 152 in Belgrade and 289 in Spain.Furthermoremore 1,320 patients are enrolled in the ERIC-pPCI

- **Ethical issues**

The regional ethics committee before initiation of the study (No. 1-10-72-167-13) approves the study protocol. The study is registered at the Danish Data Protection Agency. Furthermore, the local regional ethics committees in Asturias, Spain, and in Belgrade and Novi Sad, Serbia have approved the study and awaits final approval in the UK.

The clinical trial is registered on www.clinicaltrials.gov (NCT01857414). Good clinical practice will be observed through the trial and we will respect the appropriate international, European and national legislation and guidelines.

The Data Monitoring Committee will monitor the study. Study investigators must report all unexpected events, independently of seriousness (adverse event (AE), serious adverse event (SAE)) to the safety group of the study. The study is not a pharmaceutical drug trial or a testing of new medical devices and therefore none of the reported events is to be considered as side effects (adverse reaction (AR) or serious adverse reactions (SAR)).

The nominated sponsor of our research study will be University of Aarhus.
The study participants are during study covered under the law on patient insurance.

- **Data storing**

All study data are stored in an electronic CRF, where data are blinded and patients can be identified via a study identification number, only. In the medical record of the patient participation in the study will be recorded.
Data will be stored at the dept. of Cardiology, Aarhus University Hospital, Skejby for 15 years and subsequently the documents will be shredded.

- **Expected impacts listed in the work program**

RIC is low-cost intervention, which can be easily implemented as part of routine clinical care of STEMI patients undergoing pPCI. If successful, this project will be expected to improve clinical outcomes for STEMI patients by reducing the number of cardiac deaths and reducing the number of patients HHF at one year.

Positive as well as negative and inconclusive results will be widely disseminated through national and international scientific journals and conferences.
The authorship order: Professor Derek Haunseloy representing the ERRICA cooperation in the UK will be first author and professor Hans Erik Bøtker, Dk will be last author. The following authorships will be given according to numbers of patients included per site. There must be included 100 patients to have one authorship and 250 patients to obtain the second authorship. However, all principal investigators will be offered authorship. From Spain, this includes: MD Borja Ibanez, MD José Manuel García Ruiz and MD Richard Houghton. From Serbia, Professor Nebojsa Radovanovic, Professor Goran Stankovic, and MD Dejan Milasinovic. From Denmark MD Michael Rabek Schmidt and UK Møller Liendgaard. From UK: In the UK, PIs from the top 10 recruiting sites will be invited to be co-author on main paper – in addition Manish Ramlall (clinical fellow), Tim Clayton (senior statistician), Jennifer Nicholas (trial statistician), Richard Evans (Trials Manager), Matthew Dodd (Database manager), Rosemary Knight (senior trials manager).
All Danish/Spanish/Serbian PCI operators and committee members to be listed as collaborators (PubMed listed).
The order of appearance of the scientific authors will be according to the included number of patients at the centre. This will secure inclusion on PubMed and other medical scientific databases.

The centres involved in recruiting the patients will not be involved in any competing studies, thereby maximising the feasibility of recruitment.

- **Financial standing**

The study is supported by: The Danish Council for Strategic Research with DKK. 19.6 kr. mill, The Danish Council for Independent Research, Medical Sciences with DKK. 3.6 mill, and The Novo Nordic Foundation 600.000 DKK. The Lundbeck Foundation with DKK 4.6 mill. The money is deposited on a bank account subjected to public current audit. None of the in the study implicated doctors or nurses have any financial benefit of the study.

- **Reference List**

(1) Terkelsen CJ, Jensen LO, Tilsted HH, Trautner S, Johnsen SP, Vach W, et al. Health care system delay and heart failure in patients with ST-segment elevation myocardial infarction treated with primary percutaneous coronary intervention: follow-up of population-based medical registry data. Ann Intern Med 2011 Sep 20;155(6):361-7.

(2) Kaltoft A, Bottcher M, Nielsen SS, Hansen HH, Terkelsen C, Maeng M, et al. Routine thrombectomy in percutaneous coronary intervention for acute ST-segment-elevation myocardial infarction: a randomized, controlled trial. Circulation 2006 Jul 4;114(1):40-7.

(3) Kelbaek H, Terkelsen CJ, Helqvist S, Lassen JF, Clemmensen P, Klovgaard L, et al. Randomized comparison of distal protection versus conventional treatment in primary percutaneous coronary intervention: the drug elution and distal protection in ST-elevation myocardial infarction (DEDICATION) trial. J Am Coll Cardiol 2008 Mar 4;51(9):899-905.

(4) Kelbaek H, Thuesen L, Helqvist S, Clemmensen P, Klovgaard L, Kaltoft A, et al. Drug-eluting versus bare metal stents in patients with st-segment-elevation myocardial infarction: eight-month follow-up in the Drug Elution and Distal Protection in Acute Myocardial Infarction (DEDICATION) trial. Circulation 2008 Sep 9;118(11):1155-62.

(5) Svilaas T, Vlaar PJ, van der Horst IC, Diercks GF, de Smet BJ, van den Heuvel AF, et al. Thrombus aspiration during primary percutaneous coronary intervention. N Engl J Med 2008 Feb 7;358(6):557-67.

(6) Yellon DM, Hausenloy DJ. Myocardial reperfusion injury. N Engl J Med 2007 Sep 13;357(11):1121-35.

(7) Hausenloy DJ, Yellon DM. The mitochondrial permeability transition pore: its fundamental role in mediating cell death during ischaemia and reperfusion. J Mol Cell Cardiol 2003 Apr;35(4):339-41.

(8) Botker HE, Kharbanda R, Schmidt MR, Bottcher M, Kaltoft AK, Terkelsen CJ, et al. Remote ischaemic conditioning before hospital admission, as a complement to angioplasty, and effect on myocardial salvage in patients with acute myocardial infarction: a randomised trial. Lancet 2010 Feb 27;375(9716):727-34.

(9) Munk K, Andersen NH, Schmidt MR, Nielsen SS, Terkelsen CJ, Sloth E, et al. Remote Ischemic Conditioning in Patients With Myocardial Infarction Treated With Primary Angioplasty: Impact on Left Ventricular Function Assessed by Comprehensive Echocardiography and Gated Single-Photon Emission CT. Circ Cardiovasc Imaging 2010 Nov;3(6):656-62.

(10) Przyklenk K, Bauer B, Ovize M, Kloner RA, Whittaker P. Regional ischemic 'preconditioning' protects remote virgin myocardium from subsequent sustained coronary occlusion. Circulation 1993 Mar;87(3):893-9.

(11) Schmidt MR, Smerup M, Konstantinov IE, Shimizu M, Li J, Cheung M, et al. Intermittent peripheral tissue ischemia during coronary ischemia reduces myocardial infarction through a KATP-dependent mechanism: first demonstration of remote ischemic perconditioning. Am J Physiol Heart Circ Physiol 2007 Apr;292(4):H1883-H1890.

(12) Kharbanda RK, Mortensen UM, White PA, Kristiansen SB, Schmidt MR, Hoschtitzky JA, et al. Transient limb ischemia induces remote ischemic preconditioning in vivo. Circulation 2002 Dec 3;106(23):2881-3.

(13) Hausenloy DJ, Mwamure PK, Venugopal V, Harris J, Barnard M, Grundy E, et al. Effect of remote ischaemic preconditioning on myocardial injury in patients undergoing coronary artery bypass graft surgery: a randomised controlled trial. Lancet 2007 Aug 18;370(9587):575-9.

(14) Cheung MM, Kharbanda RK, Konstantinov IE, Shimizu M, Frndova H, Li J, et al. Randomized controlled trial of the effects of remote ischemic preconditioning on children undergoing cardiac surgery: first clinical application in humans. J Am Coll Cardiol 2006 Jun 6;47(11):2277-82.

(15) Hoole SP, Heck PM, Sharples L, Khan SN, Duehmke R, Densem CG, et al. Cardiac Remote Ischemic Preconditioning in Coronary Stenting (CRISP Stent) Study: a prospective, randomized control trial. Circulation 2009 Feb 17;119(6):820-7.

(16) Zalewski J, Nycz K, Przewlocki T, Durak M, Cul M, Zajdel W, et al. Evolution of myocardial perfusion during primary angioplasty in spontaneously reperfused infarct-related artery: impact on long-term clinical outcomes and left ventricular function recovery. Int J Cardiol 2011 Feb 17;147(1):25-31.

(17) Ndrepepa G, Tiroch K, Keta D, Fusaro M, Seyfarth M, Pache J, et al. Predictive factors and impact of no reflow after primary percutaneous coronary intervention in patients with acute myocardial infarction. Circ Cardiovasc Interv 2010 Feb 1;3(1):27-33.

(18) Kloner RA, Forman MB, Gibbons RJ, Ross AM, Alexander RW, Stone GW. Impact of time to therapy and reperfusion modality on the efficacy of adenosine in acute myocardial infarction: the AMISTAD-2 trial. Eur Heart J 2006 Oct;27(20):2400-5.

Summary of Changes in the Condi 2 protocol Version 4; May 27, 2013 original approved in 2013 to the current applicable Version 10.6 January 2017

- The study period started in 2013 and was to complete in 2016 but got prolonged with termination in 2019

- The number of patients to include was extended from 2300 – first to 4300 then to 5700. Due to a lower than expected event rate

  First:

  To detect a 25% reduction in CV mortality in the RIC-treated group (from 17.4% to 13.0%), with a power of 80% and a significance level of 5%, a sample size of 1044 patients will be required for each trial arm (2088 in total). To account for and failure of fulfilling entry criteria on arrival we plan to include 2300 patients.

  Second

  In 2015: To demonstrate a 25% reduction in the primary composite endpoint in the RIC-treated group (from 11.0% to 8.25%), with 80% power and at the 5% significance level, would require 1,805 patients per treatment arm which equates to 3,610 patients in total. Therefore, we will need to recruit 4,300 all-comer STEMI patients

  Finally

  In 2016: To detect a 25% reduction in cardiac mortality in the RIC-treated group (from 8% to 6%%), with a power of 80% and a significance level of 5%, a sample size of 2,553 patients will be required for each trial arm (5106 in total). To account for and failure of fulfilling entry criteria on arrival we plan to include 5,700 patients.

- The primary endpoint was detailed as
  - Cardiovascular mortality and hospitalisation for heart failure at one year.

  But was in 2015 specified as

  - Cardiac mortality and hospitalisation for heart failure at one year

- The follow up echo was scheduled for 3 months but the period was expanded to include the period between 3 to 6 months

CONDI 2

Effect of Remote Ischaemic Conditioning on clinical outcomes in ST-elevation myocardial infarction patients undergoing Primary Percutaneous Coronary Intervention

ERIC-PPCI

Effect of Remote Ischaemic Conditioning on clinical outcomes in ST segment elevation myocardial infarction patients undergoing Primary Percutaneous Coronary Intervention

COMBINED STATISTICAL ANALYSIS PLAN

FOR THE CONDI-2 AND ERIC-PPCI TRIALS

SAP Authors: Tim Clayton, Henrik Toft Sørensen

SAP Version: 1.5

SAP date: 17th May 2018

FINAL VERSION APPROVED BY:

Name	Signature	Date
Prof Derek Hausenloy		**03/07/2018**

Contents

1 1 .... Introduction 125

2 2 .... Study Objectives and Sample Size 125

2.1 Primary Objective 125

2.2 Secondary Objectives 126

2.3 Sample Size 126

3 3 .... Data Provision 126

4 4 .... Analysis Populations 126

4.1 Definition of Populations for Analysis 126

4.2 Intent-to-treat (ITT) 127

4.3 Per Protocol (PP) 127

5 5 .... Statistical Analysis 127

5.1 General 127

5.2 Methods for Handling Missing Data 127

5.3 Analysis of Primary Endpoint – cardiac death and hospitalisation for heart failure 12 months after randomisation. 127

5.4 Analysis of Secondary, and Other Efficacy Endpoints 128

5.4.1 Cardiac death and HHF at 30 days 128

5.4.2 Individual components of cardiac death and HHF at 30 days and 12 months 128

5.4.3 All cause death, repeat coronary revascularisation, re-infarction, stroke at 30 days and 12 months 128

5.4.4 Repeat HHF and cardiac death 129

5.5 Subgroup analysis 129

6 6 .... References 130

Appendix: Figures 131

Figure 1: Trial flow diagram ERIC-PPCI 131

Figure 2: Trial flow diagram CONDI 2 132

• Introduction

This Statistical Analysis Plan (SAP) describes the planned analysis for the CONDI-2 and ERIC-PPCI trials titled: Effect of Remote Ischaemic Conditioning on clinical outcomes in ST-segment elevation myocardial infarction patients undergoing Primary Percutaneous Coronary Intervention. Procedures for combining and reporting the relevant data from the two trials are not covered in this document. However, the specific data to be shared and made available to both study groups will be discussed and agreed between the CONDI-2 and ERIC-PPCI trial teams. It is expected that the relevant, cleaned data from each trial will be supplied as Excel spreadsheets.

Detailed descriptions of each trial can be found in the respective protocols and SAPs for each trial. This document should be read in conjunction with the Research Agreement between CONDI-2 and ERIC-PPCI version 3.0, dated 25th January 2016 together with the 1st Amendment dated 6th March 2017. In brief, the intention and purpose of the collaboration is that main trial outcomes and anonymised patient level data from each study will be merged and analysed although the primary publication will also provide results broken down by trial.

This SAP describes the data and analyses for the primary publication and not for the sub-study data. The trial statisticians from CONDI-2 and ERIC-PPCI will undertake the analyses, and discuss and resolve any discrepancies with the wider trial groups if necessary to ensure the integrity and accuracy of the results. In order for the data to be analysed as accurately and efficiently as possible a preliminary transfer of the agreed data (excluding the randomised treatment) before the trial end will occur. The timelines for the transfer of the preliminary and final data will be discussed and agreed between the trial teams.

• Study Objectives and Sample Size

The objectives for the combined analysis are as follows:

○ Primary Objective

To determine the effect of Remote ischaemic conditioning (RIC) on the combined endpoint of cardiac death and/or hospitalisation for heart failure (HHF) at 12

months in STEMI patients undergoing primary percutaneous coronary intervention (PPCI).

○ Secondary Objectives

To determine the effects of RIC on:

- Rates of the individual components of the primary endpoint at 12 months
- Rates of the combined endpoint as well the individual components of cardiac death and/or HHF at 30 days.
- Rates of the individual endpoints of all-cause death, repeat coronary revascularisation, reinfarction, stroke at 30 days and 12 months.

○ Sample Size

The primary combined endpoint will be cardiac death and HHF at 12 months. As a conservative estimate a combined cardiac death and HHF event rate of 8.5% at 12 months in the conservative arm for all-comer STEMI patients will be used. To demonstrate a 25% reduction in the primary composite endpoint in the RIC-treated group (from 8.5% to 6.4%), with 80% power and at the 5% significance level, 2395 patients per treatment arm are required which equates to 4790 patients in total. Therefore, 5400 STEMI patients will be recruited (allowing for a 10% drop out rate at 12 months) between Denmark and the UK (2600 STEMI patients in Denmark and 2800 STEMI patients in the UK). If the event rate in the control arm is higher than the estimated 8.5% or the losses less than 10% then the power will increase.

• DATA PROVISION

Detail of the variables to be combined and analysed will be provided in the parallel document detailing the logistics and timeframe for combining and analysing the data from CONDI-2 and ERIC-PPCI.

• ANALYSIS POPULATIONS

○ Definition of Populations for Analysis

The primary analysis will be performed on an intention to treat basis, by including all patients where possible according to the group to which they were randomised irrespective of whether they received the intervention as allocated.

A secondary per protocol analysis will be undertaken including only patients who receive the allocated intervention as intended.

Intent-to-treat (ITT)

The intent-to-treat analysis population will include all patients randomised to CONDI-2 and ERIC-PPCI regardless of whether they received their randomised intervention as specified (i.e. including all patients whether or not all cycles of RIC or sham RIC were fully completed according to protocol and whether or not the patient received PPCI).

Per Protocol (PP)

The per protocol analysis population will include confirmed STEMI patients randomised to CONDI-2 and ERIC-PPCI who received their randomised intervention as specified (i.e. including patients where all four cycles of RIC or sham RIC were fully completed according to protocol and the patient received PPCI).

Statistical Analysis

General

The final combined statistical analysis will be performed as pre-specified in this SAP. Any, additional, post-hoc, exploratory analyses completed to support these planned analyses which are presented in the trial reports but not included in this SAP, will be clearly identified as such. Analyses will adjust for trial (i.e. CONDI-2 or ERIC-PPCI) where indicated below.

Methods for Handling Missing Data

For the outcomes considered in this analysis, missing data are expected to be minimal. However, multiple imputation methods may be used depending on the scale and pattern of the missing data.

Analysis of Primary Endpoint – cardiac death and hospitalisation for heart failure 12 months after randomisation.

The primary combined endpoint is defined as the first occurrence of HHF or cardiac death within 12 months of randomisation. The primary analysis will be a

comparison of the rate of the first occurrence of cardiac death or HHF between the RIC and control arms of the trial. Hazard ratios and 95% confidence intervals will be calculated using Cox proportional hazards modelling, stratified by trial, and Kaplan-Meier curves will be produced. The time scale used for survival analysis will be time to the primary endpoint since randomisation. Participants will be censored if they die of a non-cardiac cause, are lost to follow-up, withdraw from the study, or at 12 months after randomisation. The assumptions underlying the Cox model will be assessed and if there is clear non-proportionality hazard ratios will be presented separately for the relevant time periods.

In addition risk differences at one year, again stratified by trial, will also be calculated together with 95% confidence intervals.

Analysis of Secondary, and Other Efficacy Endpoints

Cardiac death and HHF at 30 days

The RIC and control arms of the trial year will be compared on rate of the first occurrence of cardiac death or HHF up to 30 days following randomisation. Hazard ratios and 95% confidence intervals will be calculated using Cox proportional hazards modelling, stratified by trial, and Kaplan-Meier curves will be produced. The time scale for analysis will be time since randomisation. Participants will be censored if they die, are lost to follow-up, withdraw from the study, or at 30 days after randomisation. The risk difference stratified by trial together with 95% confidence intervals will also be calculated.

Individual components of cardiac death and HHF at 30 days and 12 months

Survival analysis will be used to examine the individual components of the composite outcome, using the same methods as outlined above.

All cause death, repeat coronary revascularisation, re-infarction, stroke at 30 days and 12 months

Survival analysis will be used to examine each of these clinical outcomes, using the same methods as outlined above.

Repeat HHF and cardiac death

In order to take account of recurrent HHFs in patients while also accounting for the competing mortality, a parametric joint frailty model will be used for outcomes up to 12 months adjusted for trial. The aim is to analyse repeat HHFs while accounting for their associated mortality risk by specifying distributions for recurrent HHFs and for time to cardiac mortality, and including individual-specific latent variables to allow for an association between the two event processes. This results in a hazard ratio and 95% CI for recurrent HHF which takes into account mortality as informative censoring as well as a hazard ratio and 95% CI for cardiac death. If there are any problems encountered with model development a negative binomial model will be used.(1-3)

- ### Subgroup analysis

A limited number of pre-specified subgroup analyses are planned on the primary outcome. These will be limited to age, diabetic status, LAD vs non-LAD STEMI, TIMI flow (0-1 and 2-3) pre-angioplasty and time of first medical contact to PPCI (i.e. balloon time). The subgroups will be analysed by inclusion of an interaction term between treatment group and the subgroup in the Cox regression model.

• References

1. Rogers JK, Pocock SJ, McMurray JJ, et al. Analysing recurrent hospitalizations in heart failure: a review of statistical methodology, with application to CHARM-Preserved. European Journal of Heart Failure. 2014;16(1):33-40.

2. Pocock SJ, Clayton TC, Stone GW. Design of Major Randomized Trials: Part 3 of a 4-Part Series on Statistics for Clinical Trials. J Am Coll Cardiol. 2015;66(24):2757-66.

3. Rogers JK, Yaroshinsky A, Pocock SJ, et al. Analysis of recurrent events with an associated informative dropout time: Application of the joint frailty model. Statistics in Medicine. 2016;35(13):2195-205.

Appendix: Figures

Figure 1: Trial flow diagram ERIC-PPCI

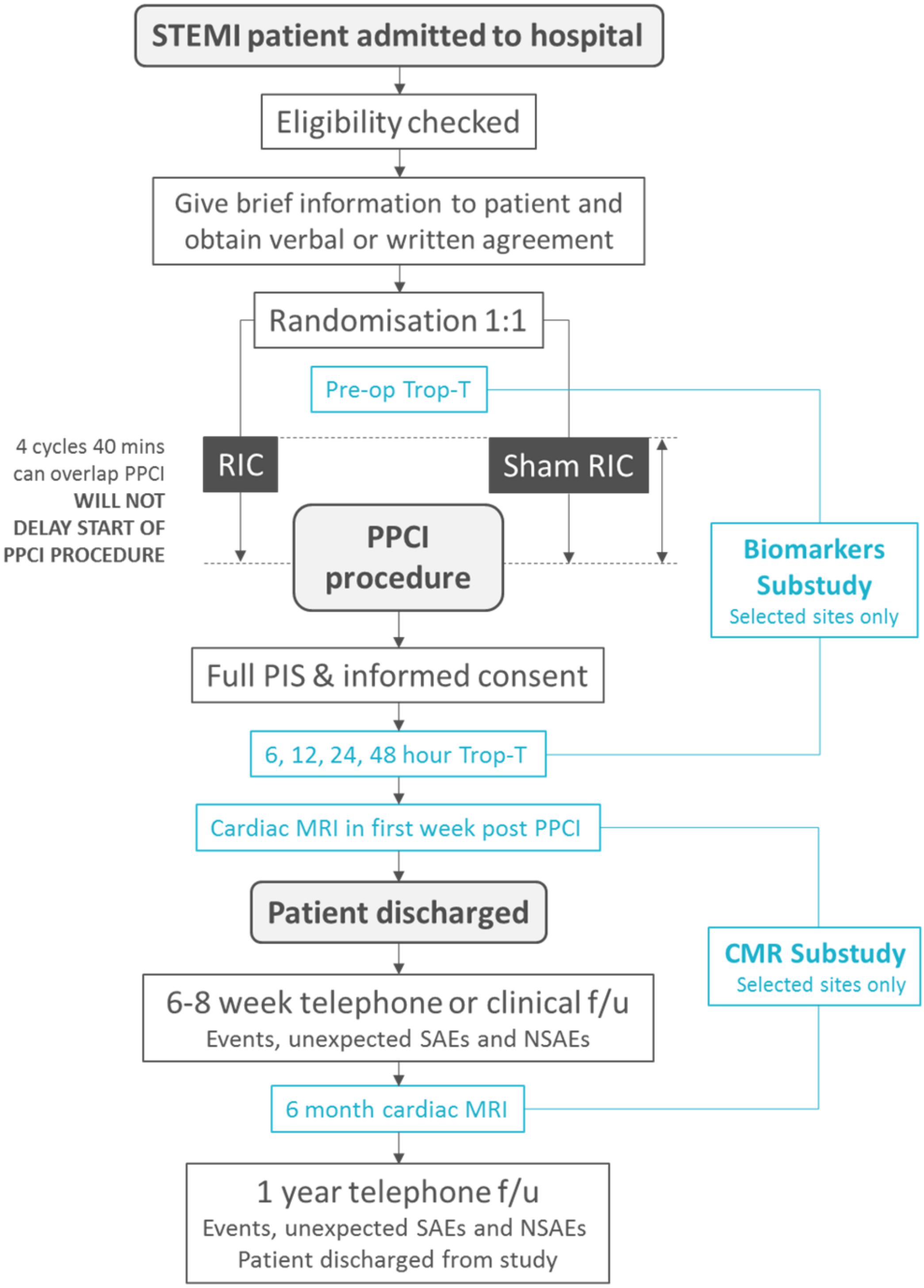

Figure 2: Trial flow diagram CONDI 2

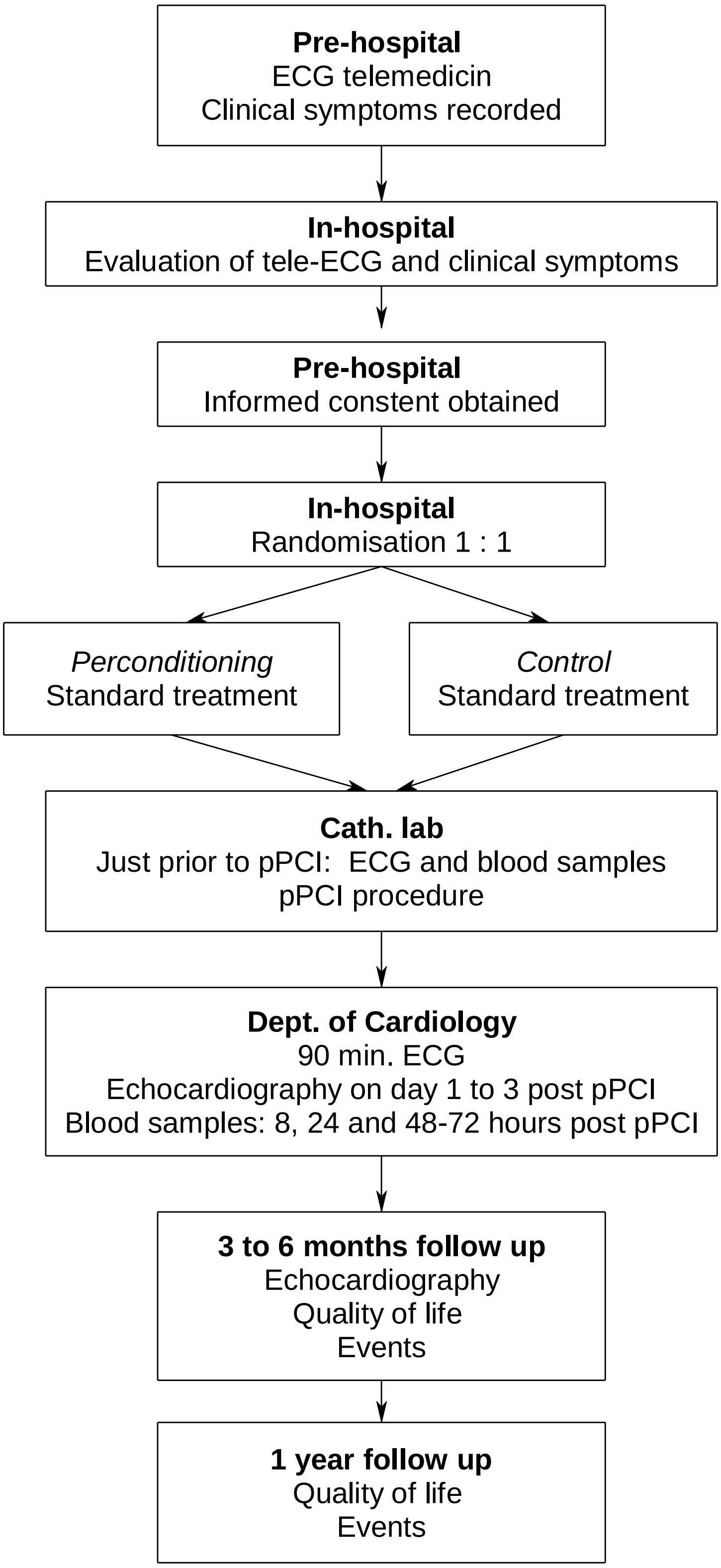

CONDI 2

Effect of Remote Ischaemic Conditioning on clinical outcomes in ST-elevation myocardial infarction patients undergoing Primary Percutaneous Coronary Intervention

ERIC-PPCI

Effect of Remote Ischaemic Conditioning on clinical outcomes in ST segment elevation myocardial infarction patients undergoing Primary Percutaneous Coronary Intervention

COMBINED STATISTICAL ANALYSIS PLAN

FOR THE CONDI-2 AND ERIC-PPCI TRIALS

SAP Authors: Tim Clayton, Henrik Toft Sørensen

SAP Version: 3.0

SAP date: 12th April 2019 (Updated by Matt Dodd)

Signature Page

FINAL VERSION APPROVED BY:

Name	Signature	Date
Derek Hausenloy		

Name	Signature	Date
Hans Erik Bøtker		

Name	Signature	Date
Henrik Toft Sørensen		

Name	Signature	Date
Tim Clayton		

Contents

7 Signature Page 134

8 1 Introduction 137

9 2 Study Objectives and Sample Size 137

2.1 Primary Objective 137

2.2 Secondary Objectives 138

2.3 Sample Size 138

10 3 Data Provision 138

11 4 Analysis Populations 138

4.1 Definition of Populations for Analysis 138

4.2 Intent-to-treat (ITT) 139

4.3 Per Protocol (PP) 139

12 5 Statistical Analysis 139

5.1 General 139

5.2 Methods for Handling Missing Data 139

5.3 Analysis of Primary Endpoint – cardiac death and hospitalisation for heart failure 12 months after randomisation. 139

5.4 Analysis of Secondary, and Other Efficacy Endpoints 140

5.4.1 Cardiac death and HHF at 30 days 140

5.4.2 Individual components of cardiac death and HHF at 30 days and 12 months 140

5.4.3 All cause death, repeat coronary revascularisation, reinfarction, stroke at 30 days and 12 months 140

5.4.4 Repeat HHF and cardiac death 140

5.4.5 Implantable cardioverter-defibrillators (ICD), biventricular pacemakers (BiV) and single/dual chamber pacemakers at 12 months 141

5.5 Subgroup analysis 141

5.6 Cardiac Biomarkers Substudy 141

13 6 .... References 143
Appendix: Figures 144
Figure 1: Trial flow diagram ERIC-PPCI 144
Figure 2: Trial flow diagram CONDI 2 145

• Introduction

This Statistical Analysis Plan (SAP) describes the planned analysis for the CONDI-2 and ERIC-PPCI trials titled: Effect of Remote Ischaemic Conditioning on clinical outcomes in ST-segment elevation myocardial infarction patients undergoing Primary Percutaneous Coronary Intervention. Procedures for combining and reporting the relevant data from the two trials are not covered in this document. However, the specific data to be shared and made available to both study groups will be discussed and agreed between the CONDI-2 and ERIC-PPCI trial teams. It is expected that the relevant, cleaned data from each trial will be supplied as Stata data files (version 13).

Detailed descriptions of each trial can be found in the respective protocols and SAPs for each trial. This document should be read in conjunction with the Research Agreement between CONDI-2 and ERIC-PPCI version 3.0, dated 25th January 2016 together with the 1st Amendment dated 6th March 2017. In brief, the intention and purpose of the collaboration is that main trial outcomes and anonymised patient level data from each study will be merged and analysed although the primary publication will also provide results broken down by trial.

The trial statisticians from CONDI-2 and ERIC-PPCI will undertake the analyses, and discuss and resolve any discrepancies with the wider trial groups if necessary to ensure the integrity and accuracy of the results. In order for the data to be analysed as accurately and efficiently as possible a preliminary transfer of the agreed data (excluding the randomised treatment) before the trial end will occur. The timelines for the transfer of the preliminary and final data will be discussed and agreed between the trial teams.

• Study Objectives and Sample Size

The objectives for the combined analysis are as follows:

○ Primary Objective

To determine the effect of Remote ischaemic conditioning (RIC) on the combined endpoint of cardiac death and/or hospitalisation for heart failure (HHF) at 12 months in STEMI patients undergoing primary percutaneous coronary intervention (PPCI).

Secondary Objectives

To determine the effects of RIC on:

- Rates of the individual components of the primary endpoint at 12 months
- Rates of the combined endpoint as well the individual components of cardiac death and/or HHF at 30 days.
- Rates of the individual endpoints of all-cause death, repeat coronary revascularisation, reinfarction, stroke at 30 days and 12 months.

Sample Size

The primary combined endpoint will be cardiac death and HHF at 12 months. As a conservative estimate a combined cardiac death and HHF event rate of 8.5% at 12 months in the conservative arm for all-comer STEMI patients will be used. To demonstrate a 25% reduction in the primary composite endpoint in the RIC-treated group (from 8.5% to 6.4%), with 80% power and at the 5% significance level, 2395 patients per treatment arm are required which equates to 4790 patients in total. Therefore, 5400 STEMI patients will be recruited (allowing for a 10% drop out rate at 12 months) between Denmark and the UK (2600 STEMI patients in Denmark and 2800 STEMI patients in the UK). If the event rate in the control arm is higher than the estimated 8.5% or the losses less than 10% then the power will increase.

DATA PROVISION

Detail of the variables to be combined and analysed will be provided in the parallel document detailing the logistics and timeframe for combining and analysing the data from CONDI-2 and ERIC-PPCI.

ANALYSIS POPULATIONS

Definition of Populations for Analysis

The primary analysis will be performed on an intention to treat basis, by including all patients where possible according to the group to which they were randomised irrespective of whether they received the intervention as allocated. A secondary per protocol analysis will be undertaken including only patients who receive the allocated intervention as intended.

○ Intent-to-treat (ITT)

The intent-to-treat analysis population will include all patients randomised to CONDI-2 and ERIC-PPCI regardless of whether they received their randomised intervention as specified (i.e. including all patients whether or not all cycles of RIC or sham RIC were fully completed according to protocol and whether or not the patient received PPCI).

○ Per Protocol (PP)

The per protocol analysis population will include confirmed STEMI patients randomised to CONDI-2 and ERIC-PPCI who received their randomised intervention as specified (i.e. including patients where all four cycles of RIC or sham RIC were fully completed according to protocol and the patient received PPCI).

• Statistical Analysis

○ General

The final combined statistical analysis will be performed as pre-specified in this SAP. Any, additional, post-hoc, exploratory analyses completed to support these planned analyses which are presented in the trial reports but not included in this SAP, will be clearly identified as such. Analyses will be stratified by trial (i.e. CONDI-2 or ERIC-PPCI) where indicated below.

○ Methods for Handling Missing Data

For the outcomes considered in this analysis, missing data are expected to be minimal. However, multiple imputation methods may be used depending on the scale and pattern of the missing data.

○ Analysis of Primary Endpoint – cardiac death and hospitalisation for heart failure 12 months after randomisation.

The primary combined endpoint is defined as the first occurrence of HHF or cardiac death within 12 months of randomisation. The primary analysis will be a comparison of the rate of the first occurrence of cardiac death or HHF between the RIC and control arms of the trial. Hazard ratios and 95% confidence intervals will be calculated using Cox proportional hazards modelling, stratified by trial,

and Kaplan-Meier curves will be produced. The time scale used for survival analysis will be time to the primary endpoint since randomisation. Participants will be censored if they die of a non-cardiac cause, are lost to follow-up, withdraw from the study, or at 12 months after randomisation. The assumptions underlying the Cox model will be assessed and if there is clear non-proportionality hazard ratios will be presented separately for the relevant time periods.

In addition risk differences at one year, again stratified by trial, will also be calculated together with 95% confidence intervals.

- ### Analysis of Secondary, and Other Efficacy Endpoints

Cardiac death and HHF at 30 days

The RIC and control arms of the trial year will be compared on rate of the first occurrence of cardiac death or HHF up to 30 days following randomisation. Hazard ratios and 95% confidence intervals will be calculated using Cox proportional hazards modelling, stratified by trial, and Kaplan-Meier curves will be produced. The time scale for analysis will be time since randomisation. Participants will be censored if they die, are lost to follow-up, withdraw from the study, or at 30 days after randomisation. The risk difference stratified by trial together with 95% confidence intervals will also be calculated.

Individual components of cardiac death and HHF at 30 days and 12 months

Survival analysis will be used to examine the individual components of the composite outcome, using the same methods as outlined above.

All cause death, repeat coronary revascularisation, reinfarction, stroke at 30 days and 12 months

Survival analysis will be used to examine each of these clinical outcomes and MACCE (all-cause death, repeat coronary revascularisation, reinfarction, or stroke) using the same methods as outlined above.

Repeat HHF and cardiac death

In order to take account of recurrent HHFs in patients while also accounting for the competing mortality, a parametric joint frailty model will be used for

outcomes up to 12 months adjusted for trial. The aim is to analyse repeat HHFs while accounting for their associated mortality risk by specifying distributions for recurrent HHFs and for time to cardiac mortality, and including individual-specific latent variables to allow for an association between the two event processes. This results in a hazard ratio and 95% CI for recurrent HHF which takes into account mortality as informative censoring as well as a hazard ratio and 95% CI for cardiac death. If there are any problems encountered with model development a negative binomial model will be used.(1-3) Repeat HHFs alone will be analysed using a negative binomial model.

Implantable cardioverter-defibrillators (ICD), biventricular pacemakers (BiV) and single/dual chamber pacemakers at 12 months

The proportions with an implantable cardioverter-defibrillator (ICD), biventricular pacemaker (BiV) or single/dual chamber pacemaker at 12 months will be compared using a generalised linear model. A risk ratio and corresponding 95% confidence interval will be calculated.

○ Subgroup analysis

A limited number of pre-specified subgroup analyses are planned on the primary outcome. These will be limited to age, diabetic status, LAD vs non-LAD STEMI, TIMI flow (0-1 and 2-3) pre-angioplasty and time of first medical contact to PPCI (i.e. balloon time). The subgroups will be analysed by inclusion of an interaction term between treatment group and the subgroup in the Cox regression model.

○ Cardiac Biomarkers Substudy

Values of high-sensitivity troponin T will be converted into a 48 hour area under the curve (AUC) summary measure for analysis. Differences in the mean AUC together with 95% confidence intervals will be calculated using a linear regression model. Troponin T will be collected at admission and 6-8, 24, and 48-72 hours post-PPCI in CONDI-2, and at admission and 6, 12, 24 and 48 hours post-PPCI in ERIC-PPCI. Where necessary, multiple imputation by chained equations will be used to account for missing information. Separate imputation models will be used for each trial. In CONDI-2, the troponin T measurement at 48-72 hours post-PPCI will be split in to those occurring at 48 hours (between 36 and 60 hours after the admission measurement) and those occurring at 72 hours (between 60 and 84 hours after the admission measurement) before multiple

imputation on all measurements up to 72 hours post-PPCI is performed. In ERIC-PPCI, multiple imputation on all measurements up to 48 hours post-PPCI will be performed. Twenty imputed datasets will be generated separately for each trial before being combined. The estimated difference in mean 48 hour AUC between the two treatment groups will be calculated using Rubin’s rules. The distribution of the outcome variable will be investigated for non-normality and if necessary a data transformation will be made or a non-parametric statistical analysis will be conducted.

The following variables will be included in the multiple imputation models: troponin-T at each time point; treatment group; country (CONDI-2 only); gender; baseline age, diabetes, hypertension, systolic blood pressure, Killip class, and body weight; use of asprin, β-blockers, nitrates, diuretics, Clopidogrel, Ticagrelor/Brillique, and metformin at admission for PPCI; previous myocardial infarction; peripheral vascular disease and hypercholesterolaemia at admission for PPCI; call to balloon time; pre/peri-procedural use of nitrates; length of index hospitalisation; and cardiac death within 12 months.

CK-MB will be analysed using data from the CONDI-2 trial only. Values of CK-MB will be converted into a 48 hour AUC summary measure for analysis. Where necessary, multiple imputation by chained equations will be used to account for missing information (as described for troponin T). The estimated difference in mean 48 hour AUC between the two treatment groups will be calculated using Rubin’s rules. The distribution of the outcome variable will be investigated for non-normality and if necessary a data transformation will be made or a non-parametric statistical analysis will be conducted.

• References

1. Rogers JK, Pocock SJ, McMurray JJ, et al. Analysing recurrent hospitalizations in heart failure: a review of statistical methodology, with application to CHARM-Preserved. European Journal of Heart Failure. 2014;16(1):33-40.

2. Pocock SJ, Clayton TC, Stone GW. Design of Major Randomized Trials: Part 3 of a 4-Part Series on Statistics for Clinical Trials. J Am Coll Cardiol. 2015;66(24):2757-66.

3. Rogers JK, Yaroshinsky A, Pocock SJ, et al. Analysis of recurrent events with an associated informative dropout time: Application of the joint frailty model. Statistics in Medicine. 2016;35(13):2195-205.

Appendix: Figures

Figure 1: Trial flow diagram ERIC-PPCI

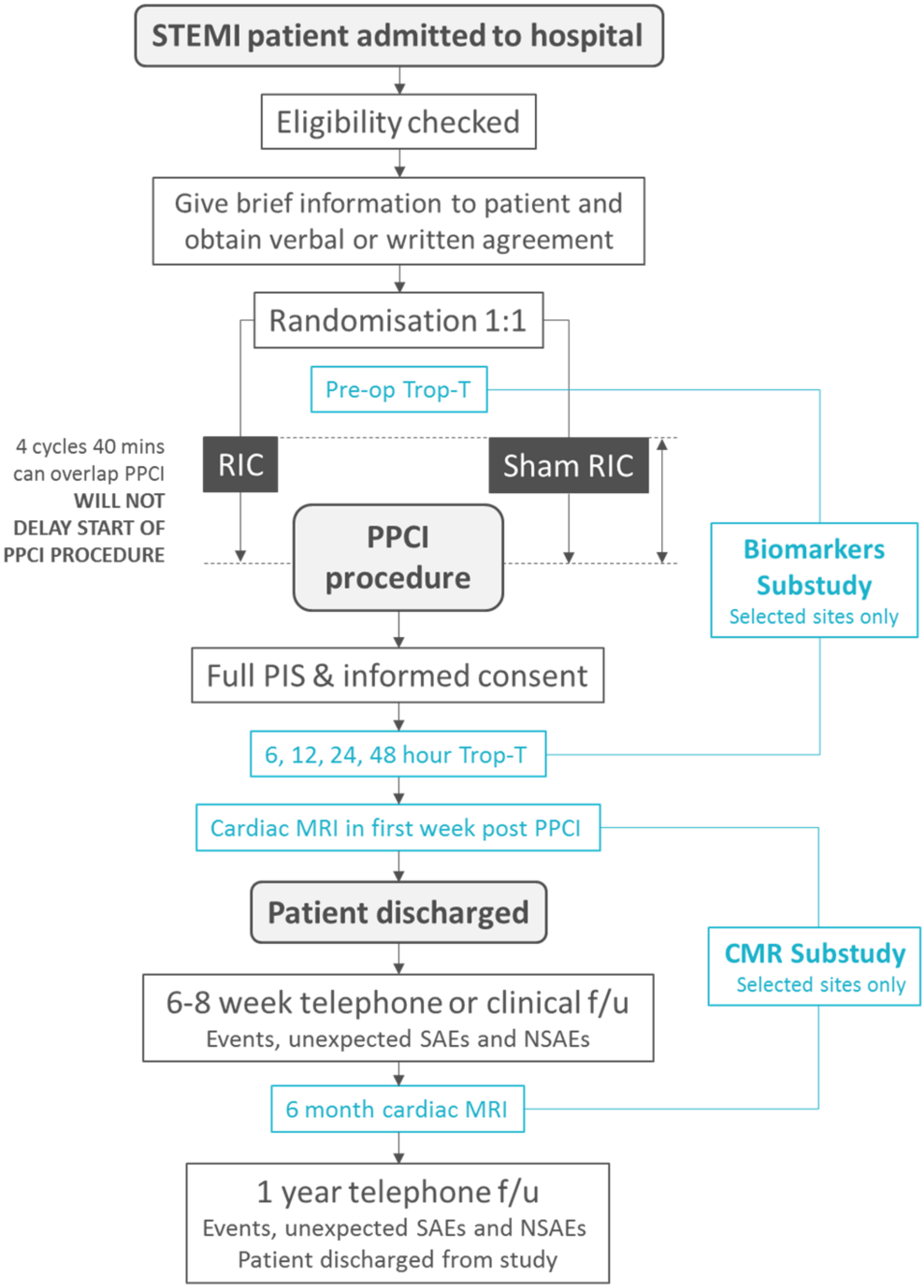

Figure 2: Trial flow diagram CONDI 2

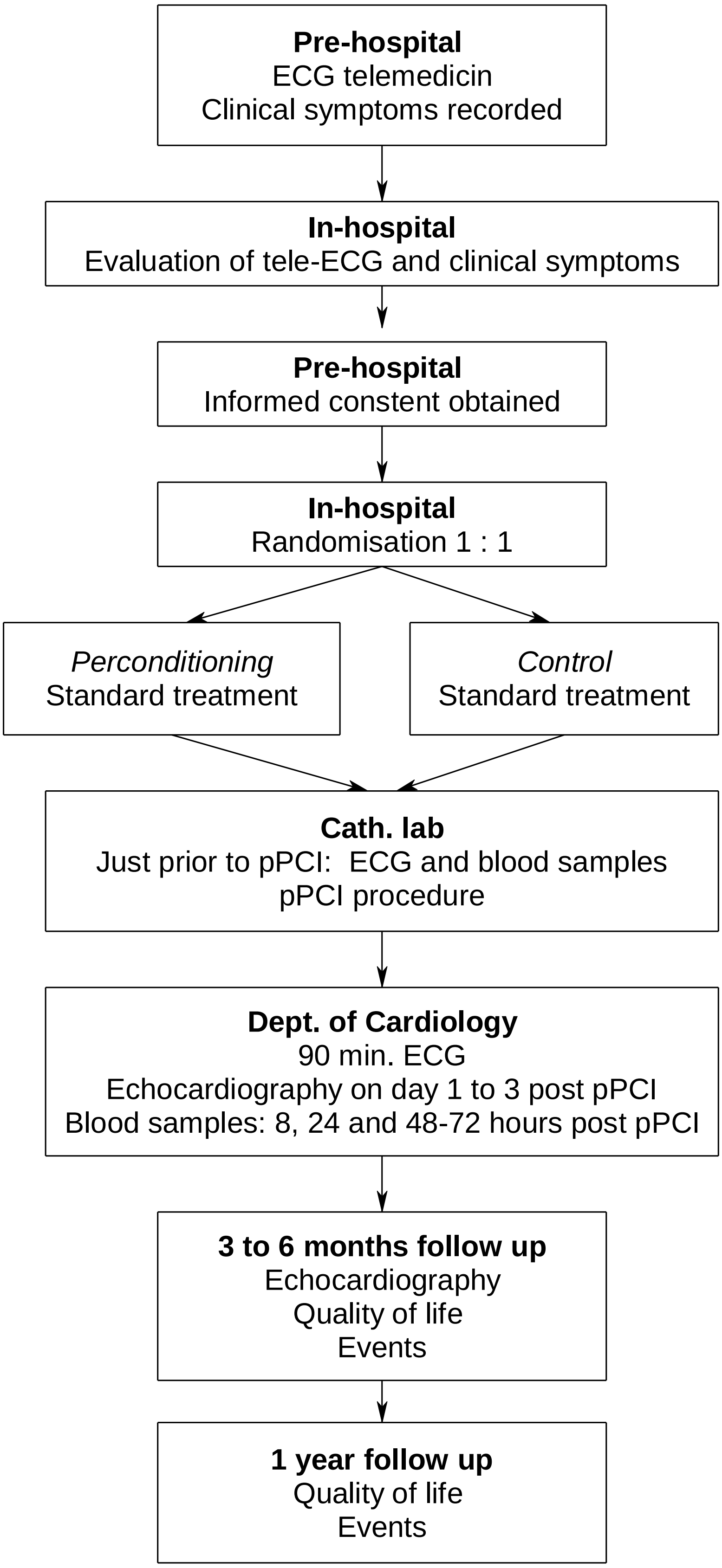

CONDI-2/ERIC-PPCI Summary of Statistical Analysis Plan Changes

Version number	SAP Data	Approved date	Changes from previous version
1.5	17 May 2018	10 August 2018	Initial approved version of the SAP. Version 1 was never moved out of draft.
2	9 April 2019	10 April 2019	The following changes were made to the SAP:<br>- Clarified that data will be supplied as STATA files<br>- Was expanded to include substudy analysis.<br>- Clarified that the analysis would be stratified by trial.<br>- Added details for the analysis of the cardiac biomarkers substudy.
3	12 April 2019	15 April 2019	The following changes were made to the SAP:<br>- It was clarified that MACCE was included in the survival analysis of the clinical outcomes.<br>- Repeat HHFs alone will be analysed.<br>- The analysis of device implantation was detailed.
